# Desmethyl SuFEx-IT: SO_2_F_2_-Free Synthesis and Evaluation as a Fluorosulfurylating Agent

**DOI:** 10.1021/acs.joc.3c02643

**Published:** 2024-02-22

**Authors:** Jan Bertram, Felix Neumaier, Boris D. Zlatopolskiy, Bernd Neumaier

**Affiliations:** † Forschungszentrum Jülich GmbH, Institute of Neuroscience and Medicine, Nuclear Chemistry (INM-5), Wilhelm-Johnen-Straße, Jülich 52425, Germany; ‡ Faculty of Medicine and Cologne University Hospital, Institute of Radiochemistry and Experimental Molecular Imaging, University of Cologne, Kerpener Straße 62, Cologne 50937, Germany

## Abstract

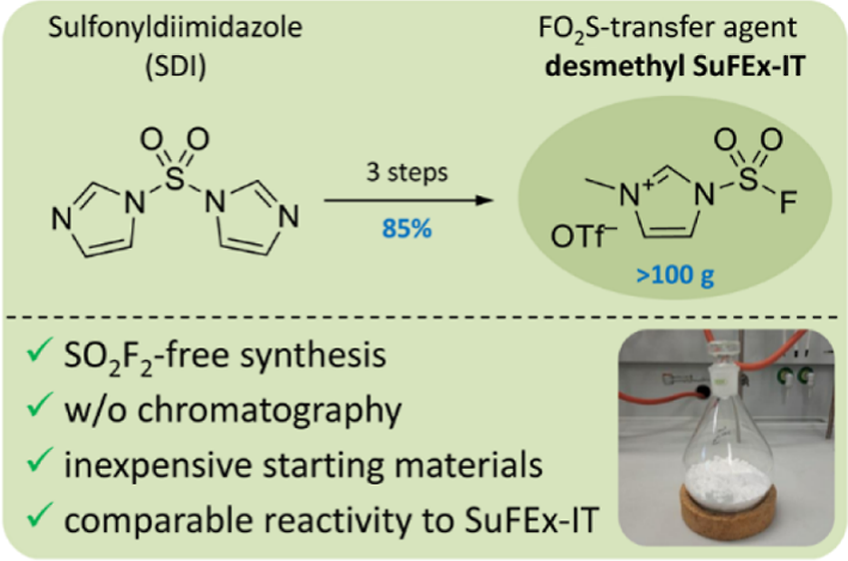

Access to SuFExable compounds was remarkably simplified by introduction of the solid FO_2_S-donor SuFEx-IT. However, the published process for preparation of this reagent relies on the use of sulfuryl fluoride (SO_2_F_2_), which is difficult to obtain and highly toxic. Herein, we disclose a simple protocol for SO_2_F_2_-free, hectogram-scale preparation of the analogous desmethyl SuFEx-IT from inexpensive starting materials. The reagent was prepared in a high (85%) total yield and without chromatographic purification steps. In addition, we demonstrate the utility of desmethyl SuFEx-IT by successful preparation of a series of fluorosulfates and sulfamoyl fluorides in high to excellent yields. As such, our work recognizes desmethyl SuFEx-IT as a valuable alternative to common FO_2_S-donors and enables cost-efficient access to substrates for SuFEx click chemistry.

## Introduction

The unique chemistry of the fluorosulfuryl (FO_2_S-) group has intrigued researchers since the early 20th century.^1,2^ Introduction of sulfur(VI)-fluoride exchange (SuFEx) as a new class of click reactions in 2014^3^ fostered scientific efforts by chemists from all over the world to exploit the exceptional properties of this functional group. The S^VI^–F bond in the FO_2_S-group is generally highly stable and can tolerate unusually harsh conditions. However, it demonstrates a latent reactivity with various nucleophiles that can be triggered under specific conditions. In addition, the facile introduction of the FO_2_S-group into target molecules using SuFEx hub-reagents has rendered FO_2_S-substituted compounds valuable building blocks across diverse applications in organic synthesis, material sciences, drug discovery, and even radiochemistry.^4^

The prototypical SuFEx hub for introduction of FO_2_S-groups into phenols or secondary amines is gaseous sulfuryl fluoride (SO_2_F_2_), which is typically obtained from pressurized lecture bottles. However, while SO_2_F_2_ shows ideal reactivity for fluorosulfurylation of phenols, it exhibits sluggish reactivity with secondary amines and has proven to be unsuitable for conversion of primary amines to the corresponding sulfamoyl fluorides. Moreover, despite the apparent simplicity of SO_2_F_2_-based fluorosulfurylation methods, their routine application is hampered by the neurotoxic nature of SO_2_F_2_, which has led to several fatalities beyond laboratory environments.^5,6^ As a consequence, the availability of SO_2_F_2_ is often restricted by regulations, and the need for specialized equipment to handle toxic gases has further impeded broad adoption of this reagent as a SuFEx hub.

To overcome these limitations, de Borggraeve and co-workers introduced a fluorosulfurylation method based on *ex situ* generation of SO_2_F_2_ from 1,1′-sulfonyldiimidazole (SDI) in a two-chamber reactor.^7^ Although this method simplifies conversion of phenols into the corresponding aryl fluorosulfates, the requirement for specialized glassware, limited scalability, and the formation of HF gas as a side product represent obvious disadvantages.

**Scheme 1:**
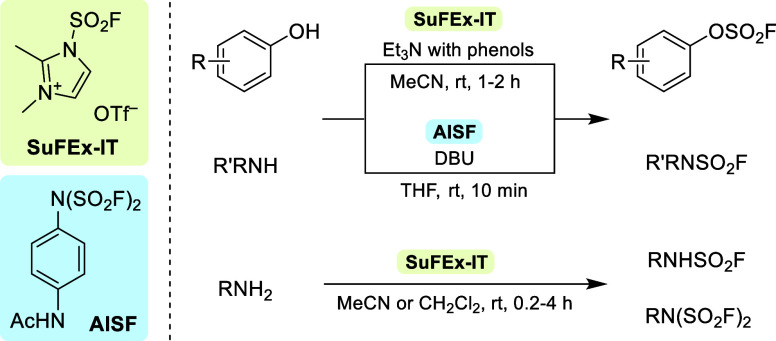
Preparation of Fluorosulfates and Sulfamoyl Fluorides with SuFEx-IT^8^ and AISF^9^

**Scheme 2:**
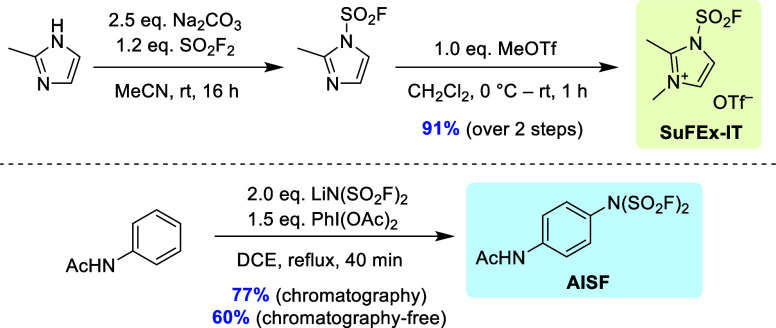
Literature Procedures for the Preparation of SuFEx-IT^8^ and AISF^9^

Accordingly, development of a fluorosulfuryl imidazolium triflate salt (termed SuFEx-IT) as a solid equivalent for SO_2_F_2_ by Guo and co-workers greatly improved access to SuFExable compounds.^8^ Thus, using SuFEx-IT as a FO_2_S-donor, the group was able to prepare a wide range of fluorosulfates and sulfamoyl fluorides from the corresponding alcohols and amines. Remarkably, SuFEx-IT showed better reactivity/chemoselectivity than SO_2_F_2_ and enabled fluorosulfurylation of primary amines, providing access to the corresponding sulfamoyl fluorides and bis(fluorosulfuryl)imides (Scheme 1). In addition, SuFEx-IT proved to be sufficiently stable for several months when stored at 4 °C or in a desiccator and could be synthesized on a multigram scale in two steps from 2-methylimidazole via fluorosulfurylation with SO_2_F_2_ followed by quaternization of the resulting intermediate with methyl triflate (MeOTf) (Scheme 2).

Another solid and bench-stable SuFEx hub developed by Zhou et al. is [4-(acetylamino)phenyl]imidodisulfuryl difluoride (AISF).^9^ This FO_2_S-donor could be prepared in a single step by oxidative C–H functionalization of acetanilide with bis(fluorosulfonyl)imide (Scheme 2). In addition, the authors demonstrated the utility of AISF for the synthesis of various aryl fluorosulfates and sulfamoyl fluorides. However, mono- or bifunctionalization of primary amines with the FO_2_S-moiety using this reagent has proven to be challenging, indicating an inferior reactivity compared to SuFEx-IT.^10^

As such, SuFEx-IT remains the most widely used reagent for facile production of sulfamoyl fluorides or fluorosulfates for SuFEx click chemistry. Nevertheless, the reagent is rather expensive and its preparation still relies on the use of toxic and hardly available SO_2_F_2_, which is associated with the aforementioned handling and regulatory issues.

For our ongoing studies on the use of SuFEx ^18^F-fluorination^11^ for the preparation of PET-tracers,^12^ a series of aryl fluorosulfates and sulfamoyl fluorides had to be prepared. Therefore, the aim of the present work was to simplify access to SuFEx-IT or alternative solid fluorosulfurylating agents by development of a simple and efficient production route that utilizes inexpensive starting materials and obviates the need for SO_2_F_2_.

## Results and Discussion

**Scheme 3:**
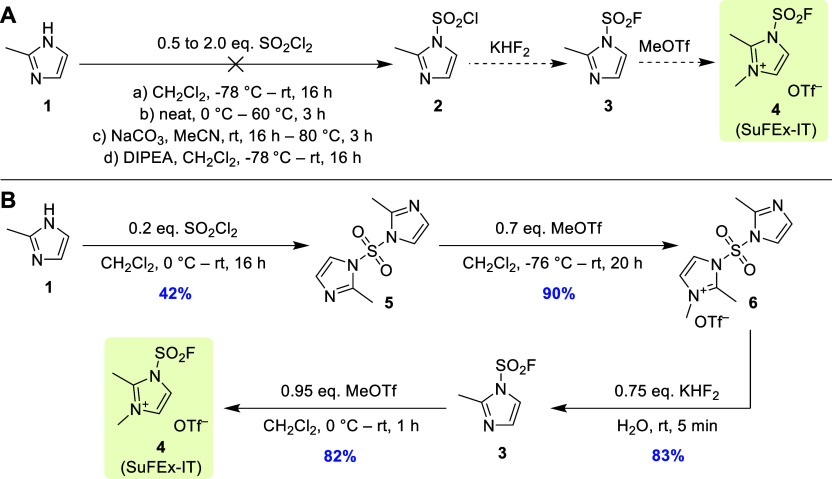
Attempted Preparation of SuFEx-IT via Sulfamoyl Chloride **2** (A) and SO_2_F_2_-Free Synthesis of SuFEx-IT (B)

Initially, we hypothesized that application of sulfuryl chloride (SO_2_Cl_2_) as an inexpensive and liquid substitute for SO_2_F_2_ could be used to improve the synthesis of SuFEx-IT (**4**). In particular, it was envisioned that reaction of 2-methylimidazole (**1**) with SO_2_Cl_2_ should afford the corresponding sulfamoyl chloride **2**, which could in turn be converted to sulfamoyl fluoride **3** using an adequate fluoride source (Scheme 3A). However, no formation of the desired sulfamoyl chloride was observed under various reaction conditions (see a–d in Scheme 3A). Therefore, this approach was abandoned.

Next, we turned our attention to the imidazolium salt **6** as an alternative precursor for sulfamoyl fluoride **3** (Scheme 3B). This compound bears the quaternized imidazolium moiety that serves as the leaving group in SuFEx-IT and could be prepared from **1** via 1,1′-sulfonylbis(2-methylimidazole) (**5**) using procedures described in the literature. To our delight, fluorination of **6** in aqueous solution proceeded efficiently and afforded sulfamoyl fluoride **3** in 83% yield. Subsequent methylation of **3** with MeOTf yielded the desired SuFEx-IT on a 3 g scale. However, a moderate overall yield of 26% (which was mainly attributable to the rather inefficient preparation of **5**) limited the practical utility of this production route.

Therefore, our interest shifted to desmethyl SuFEx-IT (**11**), which should represent a more accessible alternative to SuFExIT. Although the preparation of **11** has been described in the patent literature,^13^ neither its suitability as a FO_2_S-transfer agent nor its storage stability have been evaluated so far. In addition, the reported procedure for preparation of desmethyl SuFEx-IT is essentially the same as for the production SuFEx-IT and thus suffers from the same drawbacks.

**Scheme 4:**
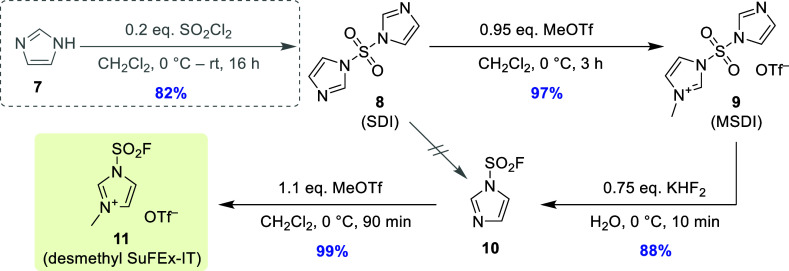
SO_2_F_2_-Free Synthesis of Desmethyl SuFEx-IT (**11**)

When the above synthetic strategy was applied to this target compound (Scheme 4), the first step could be omitted by directly starting from inexpensive SDI (**8**; available from numerous providers for 0.5–1 €/g in 25–500 g packages). Alternatively, decagram quantities of **8**(7) could be easily prepared from imidazole (**7**) and SO_2_Cl_2_ in >80% yield. Subsequent quaternization of **8** with MeOTf provided the corresponding monomethylated sulfonyldiimidazolium salt (MSDI, **9**),^14^ which precipitated from the solution and could be readily isolated in 97% yield after a total reaction time of 3 h. Thereafter, **9** was dissolved in ice-cooled water and treated with KHF_2_ to produce sulfamoyl fluoride **10**(15) within 10 min. Purification of the crude product by distillation afforded **10** in 88% yield when the reaction was performed on a decagram scale (we efficiently prepared up to ∼60 g product). On a smaller scale, the yield was ∼10% lower, presumably due to increased loss of the volatile product (see the Supporting Information). Finally, methylation of **10** with MeOTf provided, after simple crystallization, desmethyl SuFEx-IT (**11**) as a colorless solid in almost quantitative yields. This route enabled preparation of **11** on a hectogram scale in 85% total yield and without any chromatographic purifications within 2 days. Attempts to further shorten the procedure by direct fluorination of SDI were unsuccessful due to low conversion of **8** to **10**.

**Scheme 5:**
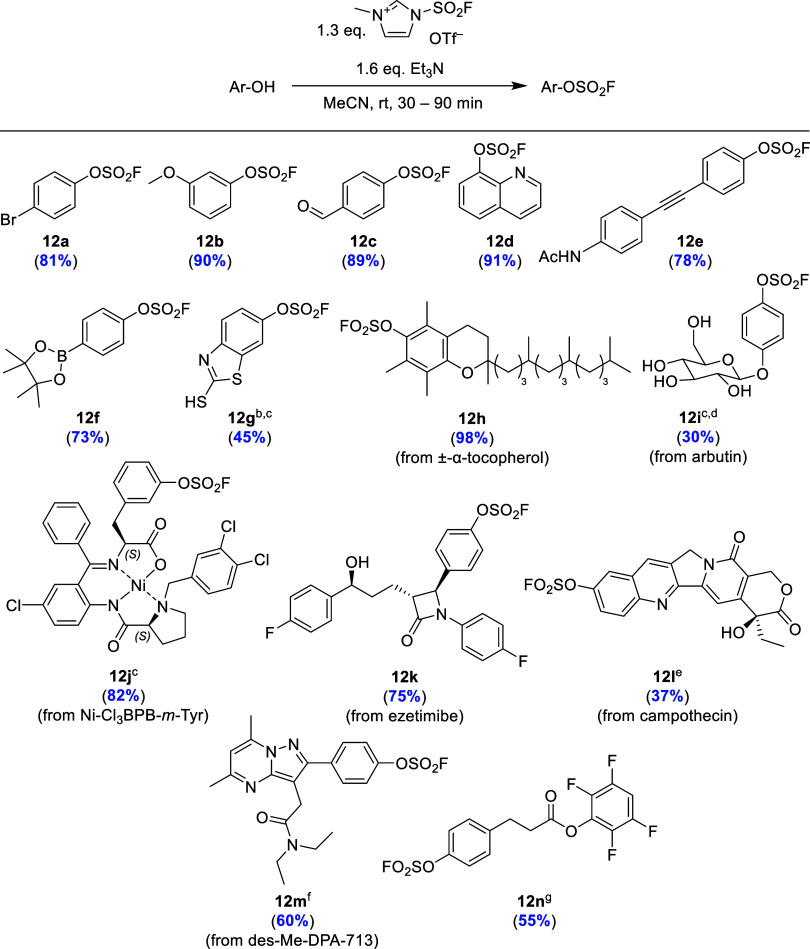
Synthesis of Fluorosulfates from the Corresponding Phenols Using Desmethyl SuFEx-IT (**11**) as a FO_2_S-Donor Indicated yields refer to isolated products. Et_3_N (2.6 equiv). Additional Et_3_N (1.0 equiv) and **11** (1.0 equiv) after 1 h. In DMF. Et_3_N (6.4 equiv) and **11** (5.2 equiv). In DMF/MeCN (1:1). Gram scale, **11** (1.4 equiv) and Et_3_N (1.7 equiv), 16 h.

Next, we investigated the reactivity and chemoselectivity of desmethyl SuFEx-IT as a FO_2_S-donor by preparation of several fluorosulfates and sulfamoyl fluorides. The results confirmed that **11** reacts readily with various simple phenols to form the corresponding fluorosulfates **12a**–**e** in yields of 78–91% within 30–90 min (Scheme 5). The reaction was unaffected by the presence of electron-withdrawing or electron-donating groups. Substrates containing a Bpin or unprotected thiol group could also be fluorosulfurylated in 73 and 45% yields, respectively (Scheme 5, **12f** and **12g**, respectively). In addition, reaction of desmethyl SuFEx-IT with more complex and/or sensitive substrates like ± -α-tocopherol (vitamin D), the skin-lightning glycoside arbutin, the Ni-complex Ni-Cl_3_BPB-*m*-Tyr, the cholesterol lowering drug ezetimibe, the topoisomerase inhibitor camptothecin or the precursor for the ^11^C-labeled TSPO-specific ligand [^11^C]DPA-713^16^ afforded the desired fluorosulfurylated products in 30–98% yields (Scheme 5, **12h**–**m**). Scalability of the procedure was confirmed by the preparation of base sensitive active ester **12n** on a gram scale in 55% yield.

**Scheme 6:**
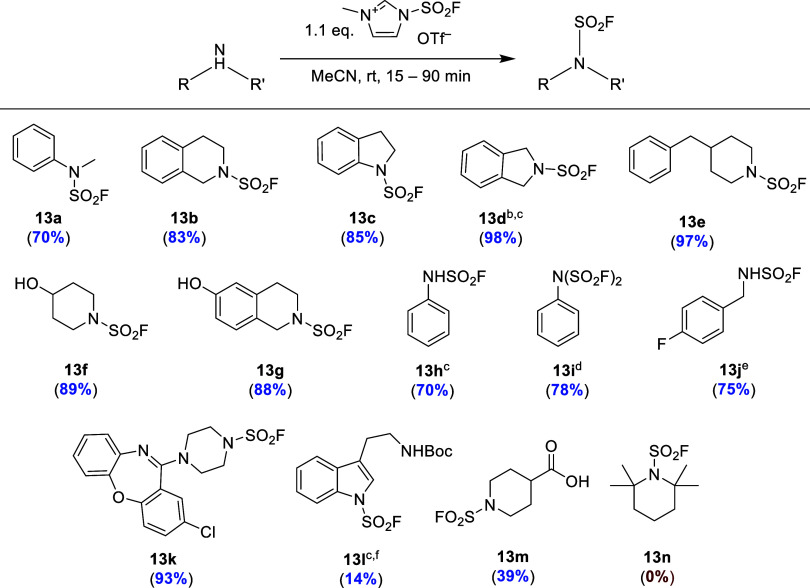
Synthesis of Sulfamoyl Fluorides from the Corresponding Amines Using Desmethyl SuFEx-IT (**11**) as a FO_2_S-Donor Indicated yields refer to isolated products. **11** (1.6 equiv) and Et_3_N (1.0 equiv). In CH_2_Cl_2_. **11** (2.5 equiv) and Et_3_N (0.5 equiv; added after 10 min), 10 min at 0 °C, then 30 min at rt. **11** (1.05 equiv). DBU (2.2 equiv).

Fluorosulfurylation of both aliphatic and aromatic secondary amines with **11** afforded the corresponding sulfamoyl fluorides **13a**–**g** in good to excellent yields (Scheme 6). Noteworthy, 4-hydroxypiperidine and 6-hydroxy-1,2,3,4-tetrahydroisoquinoline were mono-fluorosulfurylated at the nitrogen with excellent selectivity to furnish the corresponding sulfamoyl fluorides as single products in 88–89% yields (Scheme 6, **13f** and **13g**). The observed chemoselectivity for fluorosulfurylation of the secondary amino over the hydroxy groups in these substrates can most likely be attributed to its higher nucleophilicity. **11** was also successfully applied for the mono- and bi-fluorosulfurylation of aniline and mono-fluorosulfurylation of 4-fluorobenzylamine (Scheme 6, **13h**–**j**). A fluorosulfurylated derivative of the antidepressant amoxapine was prepared in 93% yield (Scheme 6, **13k**). Fluorosulfurylation of an indole nitrogen, e.g., in *N*_α_-Boc-protected tryptamine, was also possible. In this case, application of 1,8-diazabicyclo[5.4.0]undec-7-ene (DBU) as a base was necessary to prepare the desired product in a fair yield (Scheme 6, **13l**). Notably, the preparation of *N*-fluorosulfurylated indoles using SuFEx-IT or AISF has not been described so far. Finally, desmethyl SuFEx-IT enabled installation of a FO_2_S group in the presence of an unprotected carboxylic acid function, as exemplified by transformation of the GABA_A_ receptor partial agonist isonipecotic acid into the corresponding sulfamoyl fluoride in 39% yield (Scheme 6, **13m**). Generally, desmethyl SuFEx-IT demonstrated a reactivity comparable to that of SuFEx-IT.^8^ Thus, fluorosulfurylation of sterically hindered 2,2,6,6-tetramethylpiperidine, which could not be fluorosulfurylated using SuFEx-It, AISF, or SO_2_F_2_, was also impossible using desmethyl SuFEx-IT (Scheme 6, **13n**).

**Figure 1:**
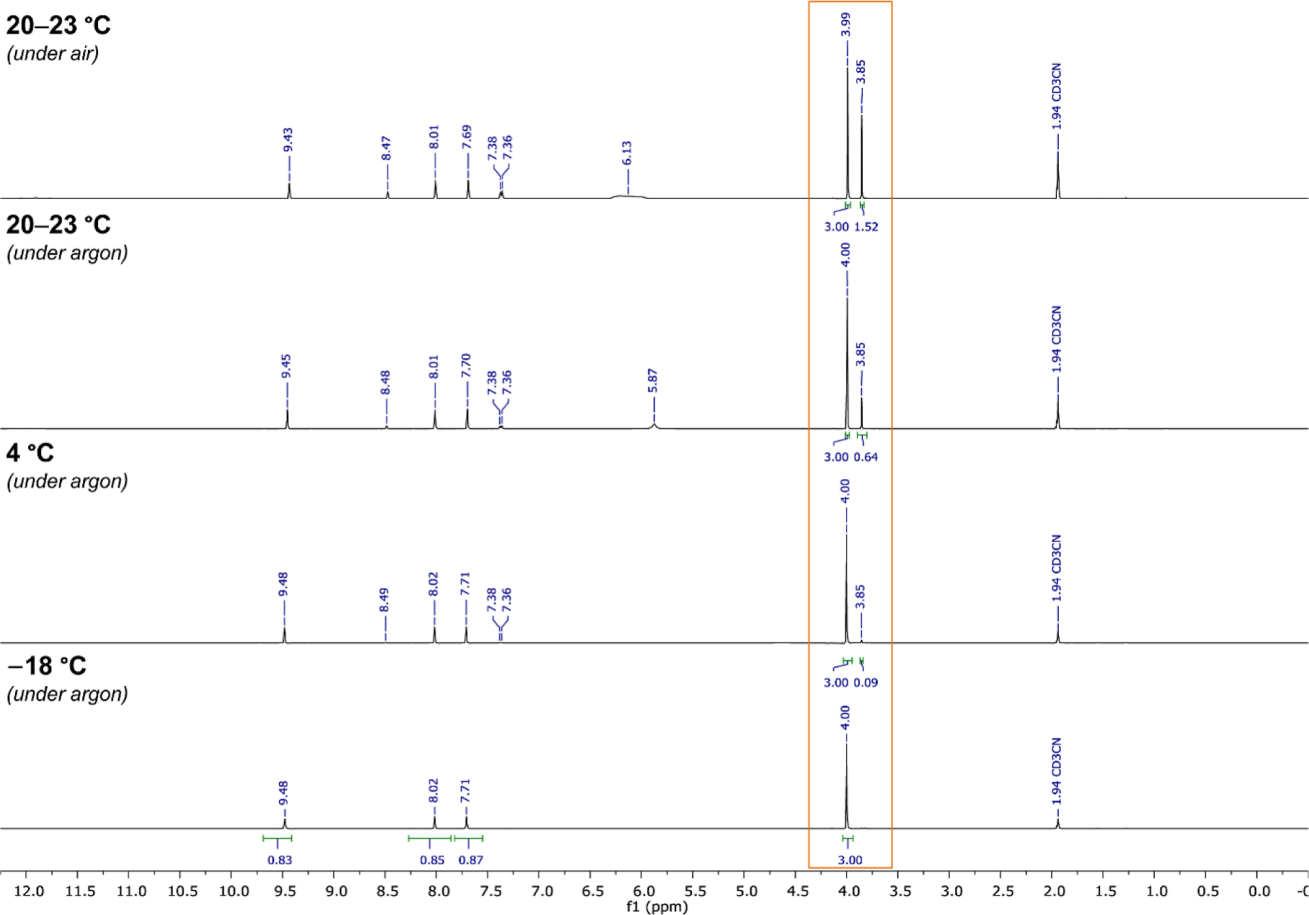
^1^H NMR of desmethyl SuFEx-IT (**11**) after storage for 288 days at the indicated temperatures under air or argon.

Finally, the long-term stability of **11** during storage under different conditions was investigated by nuclear magnetic resonance (NMR) analysis (Figure 1). Bench storage under air for 288 days at ambient temperature (20–23 °C) resulted in decomposition by 34%, which could be reduced to 18% by storing the compound under argon. In contrast, minimal (<3%) or no signs of decomposition were observed after 288 days at 4 °C or −18 °C under argon, respectively, demonstrating an excellent shelf life of **11** under these conditions.

## Conclusions

We have developed a convenient three-step procedure for the SO_2_F_2_-free, hectogram-scale preparation of desmethyl SuFEx-IT. This process affords the compound in 85% total yield from inexpensive starting materials without chromatographic purification steps within only 2 days. The utility of the reagent was demonstrated by the preparation of a series of fluorosulfates and sulfamoyl fluorides in good to excellent yields. Furthermore, desmethyl SuFEx-IT could be applied for *N*-selective mono-fluorosulfurylation of secondary amines containing aliphatic or aromatic hydroxyl groups. As such, our results identify desmethyl SuFEx-IT as a valuable alternative to common FO_2_S-donors, which offers safe and cost-efficient access to substrates for SuFEx click chemistry.

## Experimental Section

Unless noted otherwise, all chemicals and solvents were purchased from VWR International GmbH (Darmstadt, Germany), Sigma-Aldrich Chemie GmbH (Steinheim, Germany), ABCR GmbH (Karlsruhe, Germany), Apollo Scientific Ltd. (Bredbury, United Kingdom) or BLD Pharmatech GmbH (Kaiserslautern, Germany) and used without further purification.

If not stated otherwise, all reactions were carried out with magnetic stirring. Organic extracts were dried over anhydrous MgSO_4_. Air- or moisture-sensitive reagents were handled under argon (>99.999%, Air Liquide GmbH, Düsseldorf, Germany). CH_2_Cl_2_ [HPLC grade (GC: 99.8%), <0.01% H_2_O] was stored under argon and was used with moisture sensitive reagents (like MeOTf or SO_2_Cl_2_). Solutions were concentrated under reduced pressure (1–900 mbar) at 40–50 °C using a rotary evaporator (Heidolph GmbH & Co. KG, Schwabach, Germany).

### Nuclear Magnetic Resonance Spectroscopy

NMR spectra were measured at ambient temperature in deuterochloroform (CDCl_3_), trideuteroacetonitrile (CD_3_CN), hexadeuterodimethyl sulfoxide [(CD_3_)_2_SO] or octadeuterotetrahydrofuran (THF-*d*_8_) as indicated using a Bruker Ascend 400 (^1^H: 400 MHz; ^13^C{^1^H}: 101 MHz; ^19^F: 376 MHz; Bruker Biospin GmbH, Rheinstetten, Germany). The measured chemical shifts are reported in δ [ppm] relative to residual peaks of nondeuterated solvents. Higher order NMR spectra were approximately interpreted as first-order spectra if possible. The observed signal multiplicities are characterized as follows: s = singlet, bs = broad singlet, d = doublet, t = triplet, q = quartet, sep = septet, m = multiplet, dd = doublet of doublets, ddd = doublet of doublets of doublets, dt = doublet of triplets, ddt = doublet of doublets of triplets, td = triplet of doublets, tt = triplet of triplets, qd = quartet of doublets, and pt = pentet of triplets. Coupling constants *J* are reported in hertz (Hz).

### Mass Spectrometry

Low-resolution electrospray ionization mass spectrometry (LR-ESI-MS) was performed with an MSQ PlusTM mass spectrometer (Thermo Electron Corporation, San Jose, USA). High-resolution electrospray ionization mass spectrometry (HR-ESI-MS) was performed with an LTQ XL Orbitrap mass spectrometer (Thermo Fisher Scientific Inc., Bremen, Germany). Low-resolution electron ionization mass spectrometry (LR-EI-MS) was performed with an ISQ EC Single Quadrupole mass spectrometer (Thermo Fisher Scientific Inc., Bremen, Germany). High-resolution electron ionization mass spectrometry (HR-EI-MS) was performed with an Exactive GC Orbitrap mass spectrometer (Thermo Fisher Scientific Inc., Bremen, Germany).

### Column Chromatography

Manual column chromatography was performed with silica gel, 60 Å, 230–400 mesh particle size from VWR International GmbH (Darmstadt, Germany) or silica gel (w/0.1% Ca), 60 Å, 230–400 mesh particle size from Sigma-Aldrich GmbH (Steinheim, Germany). Automated column chromatography was performed on a Büchi Pure C-815 flash system (Büchi Labortechnik GmbH, Essen, Germany) using Reveleris C_18_ reversed phase cartridges (Büchi Labortechnik GmbH, Essen, Germany).

### Thin-Layer Chromatography

Thin-layer chromatography (TLC) was performed using aluminum sheets coated with silica gel 0.25 mm SIL G/UV 254 (Merck KGaA, Darmstadt, Germany). Chromatograms were inspected under UV light (λ = 254 nm) and/or stained with phosphomolybdic acid (4% in EtOH), ninhydrin (0.5% in 1-butanol), or potassium permanganate solution (0.75% KMnO_4_, 5% K_2_CO_3_, and 0.07% NaOH in H_2_O).

### Elemental Analysis

Elemental analyses were conducted by HEKAtech GmbH (Wegberg, Germany).

### Chemistry

#### 1,1′-Sulfonylbis(2-methyl-1*H*-imidazole) (**5**)^14^

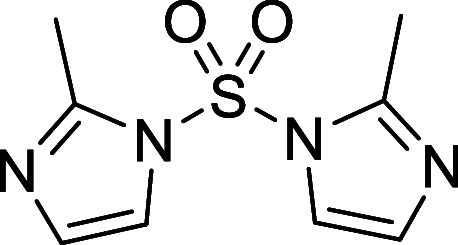



A solution of SO_2_Cl_2_ (29.99 g, 222 mmol, 1.0 equiv) in CH_2_Cl_2_ (20 mL) was added over 30 min to an ice-cooled suspension of 2-methylimidazole (82.1 g, 1.00 mol, 4.5 equiv) in CH_2_Cl_2_. The reaction mixture was stirred overnight under argon, after which H_2_O (350 mL) was added and the aqueous phase was extracted with CH_2_Cl_2_ (2 × 100 mL). The combined organic phases were washed with brine, dried, and the solvent was removed under reduced pressure. The residue was recrystallized from *i*PrOH to afford the title compound **5** (21.0 g, 93.0 mmol, 42%) as an off-white solid. Molecular formula (molecular mass): C_8_H_10_N_4_O_2_S (226.25 g/mol). ^1^H NMR (400 MHz, CDCl_3_): δ 7.37 (s, 2H), 6.94 (s, 2H), 2.51 (s, 6H). ^13^C{^1^H} NMR (101 MHz, CDCl_3_): δ 146.1, 128.6, 120.1, 15.1. HR-ESI-MS *m*/*z*: [M + H]^+^ calcd for C_8_H_11_N_4_O_2_S 227.0597; found, 227.0598.

#### 2,3-Dimethyl-1-[(2-methyl-1*H*-imidazole-1-yl)sulfonyl]-1*H*-imidazole-3-ium Trifluoromethanesulfonate (**6**)^14,17^

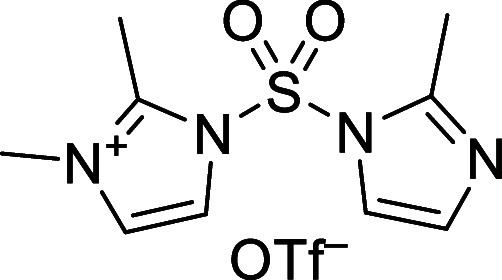



A solution of MeOTf (2.00 mL, 17.7 mmol, 0.7 equiv) in CH_2_Cl_2_ (20 mL) was added over 3.5 h to a cooled solution (−78 °C) of **5** (5.72 g, 25.3 mmol, 1.0 equiv) in CH_2_Cl_2_ (200 mL) and the reaction mixture was allowed to warm to ambient temperature within 16 h. The resulting precipitate was filtered under argon and washed with ice-cold CH_2_Cl_2_ to afford the title compound **6** (6.21 g, 15.9 mmol, 90%) as a colorless solid. Molecular formula (molecular mass): C_10_H_13_F_3_N_4_O_5_S_2_ (390.35 g/mol). ^1^H NMR (400 MHz, CD_3_CN): δ 7.89 (d, *J* = 2.5 Hz, 1H), 7.66 (d, *J* = 2.0 Hz, 1H), 7.48 (d, *J* = 2.5 Hz, 1H), 7.03 (d, *J* = 2.0 Hz, 1H), 3.75 (s, 3H), 2.76 (s, 3H), 2.56 (s, 3H). ^13^C{^1^H} NMR (101 MHz, CD_3_CN): δ 148.0, 130.0, 124.7, 121.9, 121.6, 37.0, 15.7, 12.6. ^19^F NMR (376 MHz, CD_3_CN): δ −79.29.

#### 2-Methyl-1*H*-imidazole-1-sulfonyl Fluoride (**3**)^8^

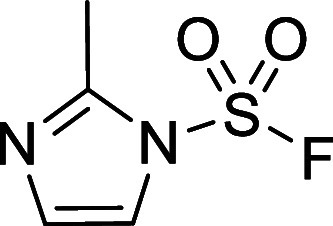



4.5 m KHF_2_* (5.56 mL, 25.0 mmol, 0.75 equiv) was added to a solution of **6** (13.0 g, 33.3 mmol, 1.0 equiv) in H_2_O (20 mL, HPLC grade) and the mixture was stirred for 5 min. The aqueous solution was extracted with Et_2_O (3 × 10 mL) and the combined organic phases were dried, filtered, and concentrated under reduced pressure at room temperature (due to the volatility of the product, the pressure was kept above 300 mbar). The raw product was purified by Kugelrohr distillation with acetone/dry ice-cooling of the receiver bulb to afford the title compound **3** (4.53 g, 27.6 mmol, 83%) as a colorless liquid. Molecular formula (molecular mass): C_4_H_5_FN_2_O_2_S (164.15 g/mol). ^1^H NMR (400 MHz, CDCl_3_): δ 7.33 (d, *J* = 1.9 Hz, 1H), 6.99 (d, *J* = 1.9 Hz, 1H), 2.66 (s, 3H). ^13^C{^1^H} NMR (101 MHz, CDCl_3_): δ 146.9, 129.1, 120.1, 15.1. ^19^F NMR (376 MHz, CDCl_3_): δ 58.16. LR-EI-MS *m*/*z*: [M]^•+^ calcd for C_4_H_5_FN_2_O_2_S 164.01; found, 164.00.

* Safety Precaution: it is strongly recommended to ensure the availability of 2.5% calcium gluconate gel as a safety measure when handling KHF_2_.

#### 1-(Fluorosulfonyl)-2,3-dimethyl-1*H*-imidazole-3-ium Trifluoromethanesulfonate (SuFEx-IT, **4**)^8^

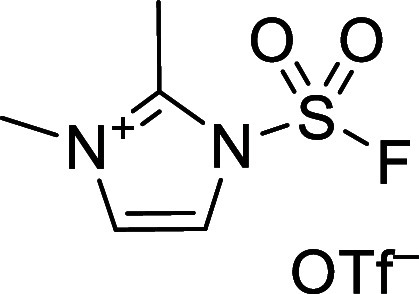



MeOTf (1.31 mL, 11.6 mmol, 0.95 equiv) was added dropwise under argon to an ice-cooled solution of **3** (2.00 g, 12.2 mmol, 1.00 equiv) in CH_2_Cl_2_. The reaction mixture was allowed to warm to ambient temperature and stirred for 1 h. The solution was then concentrated under reduced pressure, *t*BuOMe (30 mL) was added and solidification of the residual oil was induced by addition of a seed crystal and ultrasonication. The resulting solid was crushed with a spatula, filtered off, and washed with *t*BuOMe (30 mL) to afford SuFEx-IT (3.29 g, 10.0 mmol, 82%) as a colorless solid. Molecular formula (molecular mass): C_6_H_8_F_4_N_2_O_5_S_2_ (328.25 g/mol). ^1^H NMR (400 MHz, CD_3_CN): δ 7.88 (d, *J* = 2.5 Hz, 1H), 7.57 (d, *J* = 2.5 Hz, 1H), 3.86 (s, 3H), 2.86 (s, 3H). ^13^C{^1^H} NMR (101 MHz, CD_3_CN): δ 151.3, 125.5, 122.2, 122.0 (q, *J* = 320.1 Hz), 37.6, 12.9. ^19^F NMR (376 MHz, CD_3_CN): δ 60.13, −79.34.

#### 1,1′-Sulfonyldiimidazole (SDI, **8**)^7^

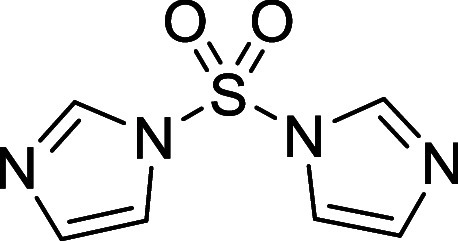



SO_2_Cl_2_ (19.4 mL, 240 mmol, 1.0 equiv) in CH_2_Cl_2_ (20 mL) was added dropwise to an ice-cooled solution of imidazole (75.0 g, 1.10 mol, 4.6 equiv) in CH_2_Cl_2_ (450 mL). The yellow suspension was stirred overnight and filtered on the next day. The solvent was removed and the crude product was recrystallized from *i*PrOH to afford the title compound **8** (38.97 g, 197 mmol, 82%) as a colorless solid. Molecular formula (molecular mass): C_6_H_6_N_4_O_2_S (198.20 g/mol). ^1^H NMR (400 MHz, CDCl_3_): δ 8.09–7.96 (m, 1H), 7.32–7.27 (m, 1H), 7.19–7.09 (m, 1H). ^13^C{^1^H} NMR (101 MHz, CDCl_3_): δ 136.6,* 132.5, 117.5. LR-ESI-MS *m*/*z*: [M + H]^+^ calcd for C_6_H_7_N_4_O_2_S 199.03; found, 199.20.

* Note that phase inversion of the C-2 signal in the APT spectrum of imidazole derivatives has been described in the literature as a “regular feature” of these compounds (for details see^18^). HSQC data further confirmed the corresponding ^1^J_CH_-coupling at C-2 (see the Supporting Information).

#### 3-(Imidazole-1-sulfonyl)-1-methyl-3*H*-imidazole-1-ium Trifluoromethanesulfonate (MSDI, **9**)^14^

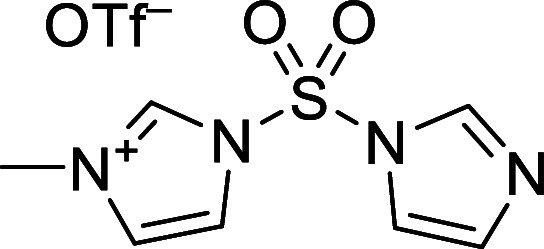



A solution of MeOTf (54.3 mL, 480 mmol, 0.95 equiv) in CH_2_Cl_2_ (80 mL) was added dropwise over the course of 30 min to an ice-cooled solution of SDI (100.0 g, 505 mmol, 1.0 equiv) in CH_2_Cl_2_ (1.5 L). The reaction mixture was stirred for 2.5 h in an ice bath, during which a colorless precipitate formed. The precipitate was allowed to settle, the supernatant solvent was decanted, and the precipitate was washed with CH_2_Cl_2_ (3 × 300 mL). The residual solvent was removed under reduced pressure to afford the title compound **9** (169.5 g, 467 mmol, 97%) as a colorless solid. Molecular formula (molecular mass): C_8_H_9_F_3_N_4_O_5_S_2_ (362.30 g/mol). ^1^H NMR (400 MHz, CD_3_CN): δ 9.37 (s, 1H), 8.27 (s, 1H), 7.93 (s, 1H), 7.68 (s, 1H), 7.55 (s, 1H), 7.23 (s, 1H), 3.90 (s, 3H). ^13^C{^1^H} NMR (101 MHz, CD_3_CN): δ 139.7, 138.9,* 133.6, 127.3, 121.5, 119.7, 38.2. ^19^F NMR (376 MHz, CD_3_CN): δ −79.33.

* Note that phase inversion of the C-2 signal in the APT spectrum of imidazole derivatives has been described in the literature as a “regular feature” of these compounds (for details see^18^).

#### 1*H*-Imidazole-1-sulfonyl Fluoride (**10**)^13^

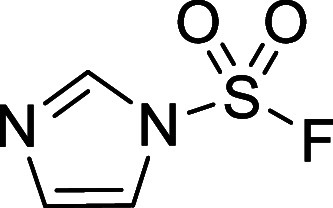



4.5 m KHF_2_* (100 mL, 341 mmol, 0.75 equiv) was added slowly to an ice-cooled solution of MSDI (164.6 g, 454 mmol, 1.0 equiv) in H_2_O (1 L, HPLC grade), and the reaction mixture was stirred for 10 min. The resulting emulsion was extracted with Et_2_O (5 × 200 mL), the combined organic phases were dried, and the solvent was removed under reduced pressure (due to the volatility of the title compound, the pressure was held above 400 mbar at 40 °C). The colorless liquid raw material thus obtained was purified by distillation (80 °C, 20 mbar, collecting flask cooled in an ice bath) to afford title compound **10** (60.1 g, 400 mmol, 88%) as a colorless liquid which crystallized on storage in the fridge. The compound should be stored at ≤ −18 °C to avoid formation of SDI over time. Molecular formula (molecular mass): C_3_H_3_FN_2_O_2_S (150.13 g/mol). ^1^H NMR (400 MHz, CDCl_3_): δ 8.05 (s, 1H), 7.42 (pt, *J* = 1.7 1H), 7.24 (dd, *J* = 1.7, 0.6 Hz, 1H). ^13^C{^1^H} NMR (101 MHz, CDCl_3_): δ 137.2,** 132.4, 118.4. ^19^F NMR (376 MHz, CDCl_3_): δ 59.59. HR-EI-MS *m*/*z*: [M]^•+^ calcd for C_3_H_3_FN_2_O_2_S 149.9894; found, 149.9893.

* Safety Precaution: it is strongly recommended to ensure the availability of 2.5% calcium gluconate gel as a safety measure when handling KHF_2_.

** Note that phase inversion of the C-2 signal in the APT spectrum of imidazole derivatives has been described in the literature as a “regular feature” of these compounds (for details see^18^).

#### 1-(Fluorosulfonyl)-3-methyl-1*H*-imidazole Trifluoromethanesulfonate (Desmethyl SuFEx-IT, **11**)^13^

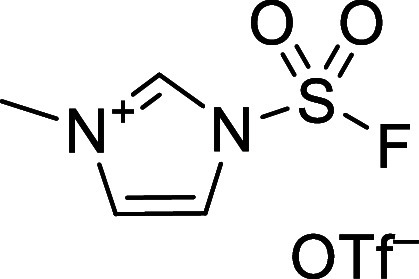



MeOTf (46.5 mL, 411 mmol, 1.1 equiv) was added dropwise over the course of 15 min to an ice-cooled solution of **10** (56.1 g, 374 mmol, 1.0 equiv) in anhydrous Et_2_O (750 mL), and the reaction mixture was stirred for 90 min. The resulting precipitate was allowed to settle, the supernatant solvent was decanted, and the precipitate was washed with Et_2_O (3 × 300 mL). The residual solvent was removed under reduced pressure and the residue dried under high vacuum overnight to afford title compound **11** (116.2 g, 370 mmol, 99%) as a colorless solid. Molecular formula (molecular mass): C_5_H_6_F_4_N_2_O_5_S_2_ (314.23 g/mol). ^1^H NMR (400 MHz, CD_3_CN): δ 9.50 (s, 1H), 8.02 (pt, *J* = 2.0 Hz, 1H), 7.72 (pt, *J* = 2.0 Hz, 1H), 4.01 (s, 3H). ^13^C{^1^H} NMR (101 MHz, CD_3_CN): δ 141.2,* 127.7, 122.8, 122.0 (q, *J* = 320.1 Hz), 38.6. ^19^F NMR (376 MHz, CD_3_CN): δ 59.89, −79.38. LR-ESI-MS *m*/*z*: [M + H]^+^ calcd for C_5_H_7_F_4_N_2_O_5_S_2_ 314.97; found, 315.06. Elemental analysis: Calcd for C_5_H_6_F_4_N_2_O_5_S_2_: C, 19.11%; H, 1.92%; N, 8.92%; S, 20.41%. Found: C, 19.06 ± 0.02%; H, 1.95 ± 0.03%; N, 8.47 ± 0.06%; S, 20.52 ± 0.08% (*n* = 2).

* Note that phase inversion of the C-2 signal in the APT spectrum of imidazole derivatives has been described in the literature as a “regular feature” of these compounds (for details see^18^).

#### General Procedure for Preparation of Fluorosulfates (GP1)

**11** (1.3 equiv) was added to a solution of the phenol substrate (1.0 equiv) and Et_3_N (1.6 equiv) in MeCN (2–5 mL), and the mixture was stirred until TLC showed full conversion (30–90 min). The products were purified by dry load column chromatography or filtration over a silica plug with an appropriate solvent unless stated otherwise.

#### General Procedure for Preparation of Sulfamoyl Fluorides (GP2)

**11** (1.1 equiv) was added to a solution of the amine substrate (1.0 equiv) in MeCN (2–5 mL), and the mixture was stirred until TLC or HPLC showed full conversion (15–90 min). The products were purified by dry load column chromatography or filtration over a silica plug with an appropriate solvent unless stated otherwise.

#### 4-Bromophenyl Sulfurofluoridate (**12a**)^19^

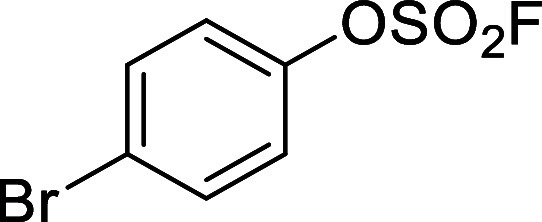



**12a** (119 mg, 467 μmol, 81%, colorless liquid) was prepared according to GP1 from 4-bromophenol (100 mg, 578 μmol) and purified by column chromatography (*R*_*f*_ = 0.38, *n*-hexane). Molecular formula (molecular mass): C_6_H_4_BrFO_3_S (255.06 g/mol). ^1^H NMR (400 MHz, CDCl_3_): δ 7.64–7.58 (m, 2H), 7.28–7.20 (m, 2H). ^13^C{^1^H} NMR (101 MHz, CDCl_3_): δ 149.1, 133.7, 122.8, 122.5. ^19^F NMR (376 MHz, CDCl_3_): δ 37.80. HR-EI-MS *m*/*z*: [M]^•+^ calcd for C_6_H_4_^79^BrFO_3_S 253.9043; found, 253.9039. HR-EI-MS *m*/*z*: [M]^•+^ calcd for C_6_H_4_^81^BrFO_3_S 255.9023; found, 255.9018.

#### 3-Methoxyphenyl Sulfurofluoridate (**12b**)^12^

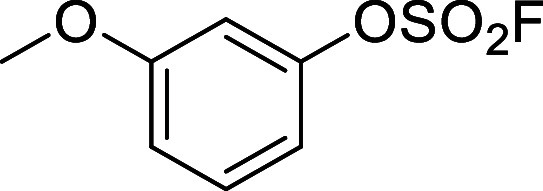



**12b** (170 mg, 824 μmol, 90%, colorless liquid) was prepared according to GP1 from 3-methoxyphenol (113 mg, 910 μmol) and purified by filtration over a silica plug (*R*_*f*_ = 0.47, EtOAc:*n*-hexane = 1:8). Molecular formula (molecular mass): C_7_H_7_FO_4_S (206.19 g/mol). ^1^H NMR (400 MHz, CDCl_3_): δ 7.37 (t, *J* = 8.3 Hz, 1H), 6.94 (td, *J* = 7.3, 6.3, 1.7 Hz, 2H), 6.87 (d, *J* = 2.2 Hz, 1H), 3.84 (s, 3H). ^13^C{^1^H} NMR (101 MHz, CDCl_3_): δ 161.1, 150.9, 130.8, 114.5, 112.8, 107.1, 55.8. ^19^F NMR (376 MHz, CDCl_3_): δ 37.70. HR-EI-MS *m*/*z*: [M]^•+^ calcd for C_7_H_7_FO_4_S 206.0044; found, 206.0038.

#### 4-Formylphenyl Sulfurofluoridate (**12c**)^20^

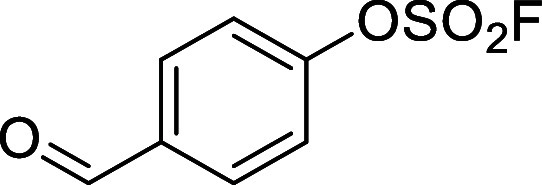



**12c** (149 mg, 730 μmol, 89%, colorless liquid) was prepared according to GP1 from 4-hydroxybenzaldehyde (100 mg, 819 μmol) and purified by column chromatography (*R*_*f*_ = 0.21, EtOAc:*n*-hexane = 1:6). Molecular formula (molecular mass): C_7_H_5_FO_4_S (204.17 g/mol). ^1^H NMR (400 MHz, CDCl_3_): δ 10.06 (s, 1H), 8.06–8.00 (m, 2H), 7.57–7.48 (m, 2H). ^13^C{^1^H} NMR (101 MHz, CDCl_3_): δ 190.2, 153.7, 136.3, 132.0, 121.9. ^19^F NMR (376 MHz, CDCl_3_): δ 39.11. HR-EI-MS *m*/*z*: [M]^•+^ calcd for C_7_H_5_FO_4_S 203.9887; found, 203.9885.

#### Quinolin-8-yl Sulfurofluoridate (**12d**)^12^

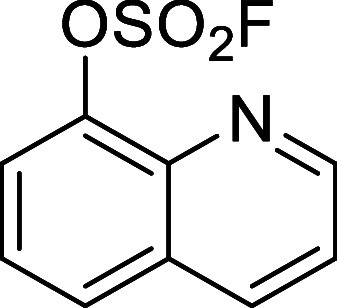



**12d** (142 mg, 627 μmol, 91%, pale yellow solid) was prepared according to GP1 from 8-hydroxyquinoline (100 mg, 689 μmol) and purified by column chromatography (*R*_*f*_ = 0.33, EtOAc:*n*-hexane = 1:6). Molecular formula (molecular mass): C_9_H_6_FNO_3_S (227.21 g/mol). ^1^H NMR (400 MHz, CDCl_3_): δ 9.06 (dd, *J* = 4.2, 1.6 Hz, 1H), 8.24 (dd, *J* = 8.4, 1.6 Hz, 1H), 7.90 (dd, *J* = 8.4, 1.1 Hz, 1H), 7.75 (dt, *J* = 7.7, 1.1 Hz, 1H), 7.66–7.47 (m, 2H). ^13^C{^1^H} NMR (101 MHz, CDCl_3_): δ 152.0, 146.0, 140.5, 136.1, 130.1, 128.8, 126.0, 122.8, 121.5. ^19^F NMR (376 MHz, CDCl_3_): δ 40.66. HR-EI-MS *m*/*z*: [M]^•+^ calcd for C_9_H_6_FNO_3_S 227.0047; found, 227.0041.

#### 4-[(4-Acetamidophenyl)ethynyl]phenyl Sulfurofluoridate (**12e**)

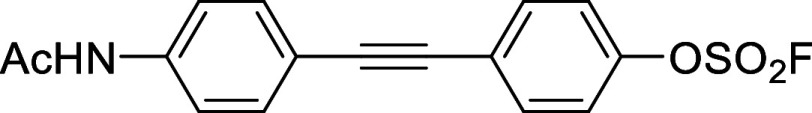



**12e** (65.0 mg, 195 μmol, 78%, yellow solid) was prepared according to GP1 from *N*-{4-[(4-hydroxyphenyl)ethynyl]phenyl}acetamide (62.8 mg, 250 μmol) and purified by column chromatography (*R*_*f*_ = 0.19, EtOAc:*n*-hexane = 1:1). Molecular formula (molecular mass): C_16_H_12_FNO_4_S (333.33 g/mol). ^1^H NMR (400 MHz, THF-*d*_8_): δ 9.22 (s, 1H), 7.72–7.57 (m, 4H), 7.52–7.45 (m, 2H), 7.45–7.38 (m, 2H), 2.04 (s, 3H). ^13^C{^1^H} NMR (101 MHz, THF-*d*_8_): δ 168.6, 150.5, 141.6, 134.3, 133.0, 125.7, 122.2, 119.5, 117.6, 92.3, 87.2, 24.2. ^19^F NMR (376 MHz, THF-*d*_8_): δ 36.77. HR-EI-MS *m*/*z*: [M]^•+^ calcd for C_16_H_12_FNO_4_S 333.0466; found, 333.0467.

#### 4-(4,4,5,5-Tetramethyl-1,3,2-dioxaborolan-2-yl)phenyl Sulfurofluoridate (**12f**)

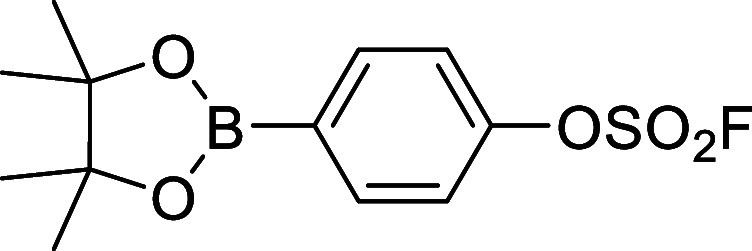



**12f** (100 mg, 330 μmol, 73%, pale yellow solid) was prepared according to GP1 from 4-(4,4,5,5-tetramethyl-1,3,2-dioxaborolan-2-yl)phenol (100 mg, 454 μmol). The reaction mixture was extracted with *n*-pentane (4 × 10 mL) and the collected phases were filtered over a silica plug (*R*_*f*_ = 0.43, *n*-pentane). Molecular formula (molecular mass): C_12_H_16_BFO_5_S (302.12 g/mol). ^1^H NMR (400 MHz, CDCl_3_): δ 7.96–7.88 (m, 2H), 7.37–7.30 (m, 2H), 1.35 (s, 12H). ^13^C{^1^H} NMR (101 MHz, CDCl_3_): δ 152.3, 137.2, 120.2, 84.5, 25.0. *C*-B was not observed. ^19^F NMR (376 MHz, CDCl_3_): δ 38.11. HR-EI-MS *m*/*z*: [M]^•+^ calcd for C_12_H_16_BFO_5_S 302.0790; found, 302.0794; [M-CH_3_]^•+^ calcd for C_11_H_13_BFO_5_S 287.0555; found, 287.0556.

#### 2-Mercaptobenzo[*d*]thiazol-6-yl Sulfurofluoridate (**12g**)

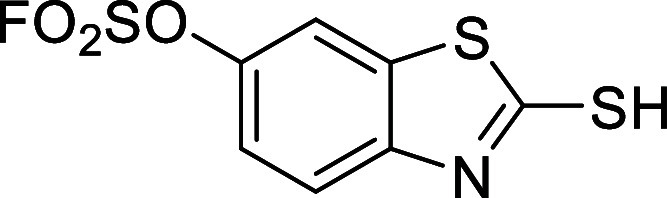



**12g** (64.9 mg, 245 μmol, 45%, colorless solid) was prepared according to a modification of GP1 as follows. 2-Mercaptobenzo[*d*]thiazol-6-ol (100 mg, 546 μmol) was dissolved (under argon) in a solution of Et_3_N (197 μL, 1.42 mmol, 2.6 equiv) in MeCN (3 mL) before **11** was added. After 1 h, a second portion of **11** and Et_3_N (1 equiv of each) was added and the reaction mixture was left to stand for another 30 min before it was diluted with CH_2_Cl_2_ (20 mL) and washed with 0.1 M HCl (2 × 10 mL). The organic phase was dried and **12g** was isolated by dry load filtration over a silica plug (*R*_*f*_ = 0.34, EtOAc:*n*-hexane = 1:3). Molecular formula (molecular mass): C_7_H_4_FNO_3_S_3_ (265.29 g/mol). ^1^H NMR [400 MHz, (CD_3_)_2_SO]: δ 14.01 (s, 1H), 8.07 (s, 1H), 7.62 (dd, *J* = 8.9, 2.1 Hz, 1H), 7.41 (d, *J* = 8.9 Hz, 1H). ^13^C{^1^H} NMR [101 MHz, (CD_3_)_2_SO]: δ 191.2, 145.9, 141.5, 131.0, 120.4, 115.1, 113.5. ^19^F NMR [376 MHz, (CD_3_)_2_SO]: δ 38.34. HR-EI-MS *m*/*z*: [M]^•+^ calcd for C_7_H_4_FNO_3_S_3_ 264.9332; found, 264.9332.

#### ±-α-Tocopherol-Derived Sulfurofluoridate (**12h**)^12^

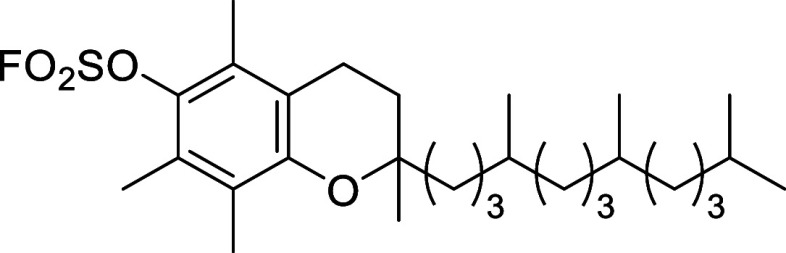



**12h** (256 mg, 499 μmol, 98%, colorless oil) was prepared according to GP1 from ±-α-tocopherol (220 mg, 511 μmol) and purified by filtration over a silica plug (*R*_*f*_ = 0.79, EtOAc:*n*-hexane = 1:9). Molecular formula (molecular mass): C_29_H_49_FO_4_S (512.77 g/mol). Mixture of diastereomers. Inequivalent signals of diastereomers in the ^13^C{^1^H} NMR spectrum are separated by forward slash symbols. ^1^H NMR (400 MHz, CDCl_3_): δ 2.60 (t, *J* = 6.8 Hz, 2H), 2.23 (s, 3H), 2.20 (s, 3H), 2.10 (s, 3H), 1.89–1.72 (m, 2H), 1.61–1.06 (m, 25H), 0.94–0.77 (m, 12H). ^13^C{^1^H} NMR (101 MHz, CDCl_3_): δ 151.2, 142.0, 127.6, 126.2, 124.5, 118.6, 75.9, 40.1/40.0, 39.5, 37.7/37.62, 37.60/37.54, 37.49/37.4, 33.0/32.9, 32.83/32.81, 31.0, 30.9, 28.1, 25.0, 24.6, 24.0, 22.9/22.8, 21.1, 20.8, 19.9/19.83, 19.82/19.7, 13.7, 12.9, 12.1. ^19^F NMR (376 MHz, CDCl_3_): δ 41.37. HR-EI-MS *m*/*z*: [M]^•+^ calcd for C_29_H_49_FO_4_S 512.3330; found, 512.3330.

#### 4-{[(2*S*,3*R*,4*S*,5*S*,6*R*)-3,4,5-Trihydroxy-6-(hydroxymethyl)tetrahydro-2*H*-pyran-2-yl]oxy}phenyl Sulfurofluoridate (**12i**)

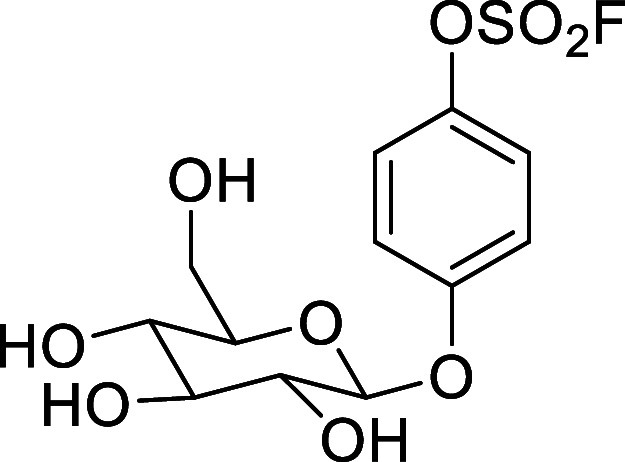



**12i** (39.5 mg, 111 μmol, 30%, colorless solid) was prepared according to a modification of GP1 from arbutin (100 mg, 367 μmol) as follows. **11** was added to a solution of the starting material and Et_3_N in DMF (3 mL) and the reaction mixture was left to stand for 1 h before another portion of Et_3_N and **11** (1 equiv of each) was added. The crude material obtained was purified by RP chromatography. Conditions: column: FlashPure Select C_18_ 12 g (Büchi Labortechnik GmbH, Essen, Germany); eluent: 0–5 min: 10% MeCN, 5–20 min: 10 → 20% MeCN, 20–25 min: 20 → 100% MeCN; flow rate: 20 mL/min; detection: UV, λ = 210 and 220 nm. Molecular formula (molecular mass): C_12_H_15_FO_9_S (354.30 g/mol). ^1^H NMR (400 MHz, CD_3_OD): δ 7.37 (d, *J* = 9.1 Hz, 2H), 7.28–7.18 (m, 2H), 5.04–4.92 (m, 1H), 3.90 (dd, *J* = 12.1, 2.0 Hz, 1H), 3.70 (dd, *J* = 12.1, 5.7 Hz, 1H), 3.55–3.33 (m, 4H). ^13^C{^1^H} NMR (101 MHz, CD_3_OD): δ 158.8, 146.2, 123.1, 119.3, 102.3, 78.3, 77.9, 74.8, 71.3, 62.4. ^19^F NMR (376 MHz, CD_3_OD): δ 34.55. HR-ESI-MS *m*/*z*: [M + Na]^+^ calcd for C_12_H_15_FO_9_SNa 377.03130; found, 377.03180.

#### (*S,S*)-Ni-Cl_3_BPB-*m*-Tyr Sulfurofluoridate (**12j**)

##### (*S,S*)-Ni-Cl_3_BPB-*m*-Tyr

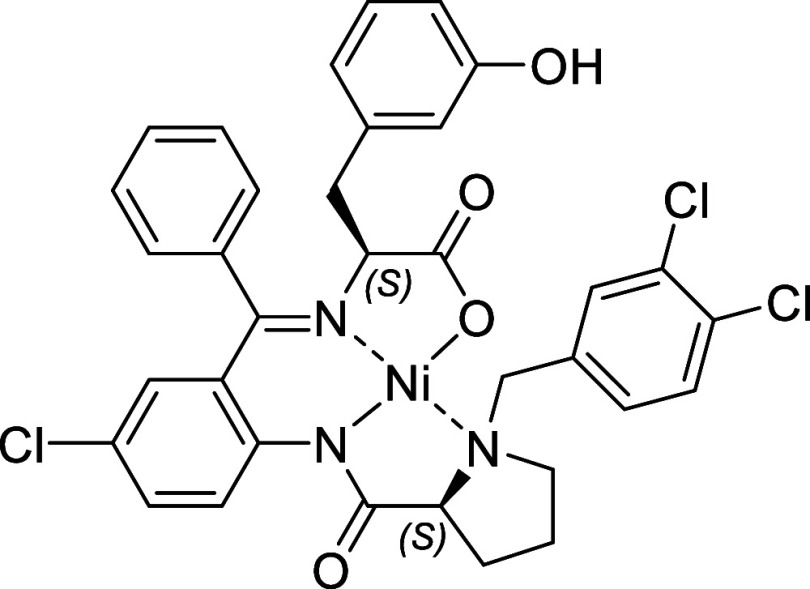



A solution of (*S*)-*N*-(2-benzoyl-4-chlorophenyl)-1-(3,4-dichlorobenzyl)pyrrolidine-2-carboxamide^21^ (8.1 g, 16.6 mmol), racemic *m*-Tyr (5.89 g, 32.5 mmol), Ni(OAc)_2_·4H_2_O (8.09 g, 32.5 mmol), and K_2_CO_3_ (20.4 g, 147.75 mmol) in anhydrous MeOH (400 mL) was stirred at 60 °C for 24 h and at ambient temperature for 72 h. The reaction mixture was poured into an ice-cold solution of AcOH (50 mL) in H_2_O (3 L) and the resulting suspension was allowed to stand at ambient temperature for 24 h, after which a fine red precipitate had formed. The precipitate was collected by filtration, washed with H_2_O (3 × 100 mL), air-dried, and dissolved in EtOAc (200 mL). The resulting solution was washed with H_2_O (3 × 50 mL) and brine (2 × 50 mL), dried, and concentrated under reduced pressure. The residue was triturated with Et_2_O to give a red precipitate, which was recrystallized from EtOAc/hexane to afford a first crop of the title compound as a red solid. The combined mother liquors (from trituration with Et_2_O and recrystallization) were concentrated under reduced pressure and the residue was purified by column chromatography (*R*_*f*_ = 0.25; CHCl_3_:acetone = 5:1; broad spot) followed by recrystallization from EtOAc/hexane to afford a second crop of the title compound (total: 10.35 g, 88%) as a red solid. Molecular formula (molecular mass): C_34_H_28_Cl_3_N_3_NiO_4_ (707.66 g/mol). ^1^H NMR (400 MHz, CDCl_3_): δ 8.90 (s, 1H), 8.30 (br s, 1H), 8.09 (d, *J* = 8.9 Hz, 1H), 7.81–7.41 (m, 4H), 7.40–7.20 (m, 3H), 7.10 (d, *J* = 8.5 Hz, 1H), 6.91 (dd, *J* = 22.3, 6.9 Hz, 2H), 6.80–6.70 (m, 1H), 6.69–6.53 (m, 2H), 4.29 (s, 1H), 4.13 (d, *J* = 12.4 Hz, 1H), 3.33–3.16 (m, 1H), 3.09 (d, *J* = 11.6 Hz, 2H), 2.96 (d, *J* = 12.4 Hz, 1H), 2.67 (d, *J* = 8.8 Hz, 1H), 2.45–2.19 (m, 3H), 2.17–1.83 (m, 2H). ^13^C{^1^H}-NMR (101 MHz, CDCl_3_): δ 180.2, 179.1, 171.0, 157.7, 140.9, 136.8, 135.0, 133.8, 133.44, 133.36, 133.3, 132.6, 132.5, 131.1, 130.5, 130.2, 129.9, 129.6, 129.4, 127.8, 127.4, 127.2, 126.0, 124.0, 122.2, 117.7, 115.3, 71.7, 71.6, 63.5, 58.8, 39.2, 30.9, 23.1. *ortho*- and *meta*-Carbons of the phenyl substituent are not equivalent owing to hindered rotation. HR-ESI-MS *m*/*z*: [M + K]^+^ calcd for C_34_H_28_Cl_3_N_3_NiO_4_K 744.01387; found, 744.01305; [M + Na]^+^ calcd for C_34_H_28_Cl_3_N_3_NiO_4_Na 728.04024; found, 728.03911; [M + H]^+^ calcd for C_34_H_29_Cl_3_N_3_NiO_4_ 706.05836; found, 706.05716. Correct isotopic pattern.

##### (*S,S*)-Ni-Cl_3_BPB-*m*-Tyr Sulfurofluoridate (**12j**)

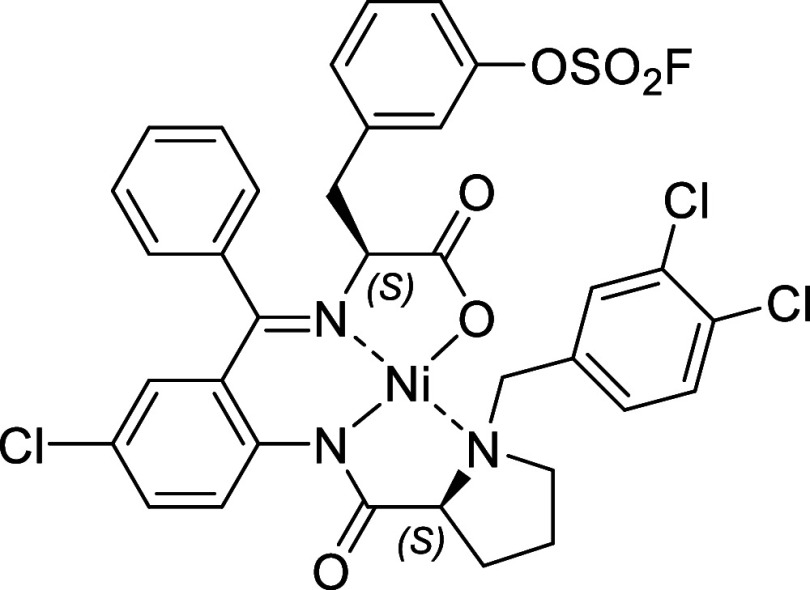



**12j** (90.8 mg, 115 μmol, 82%, red solid) was prepared according to a modification of GP1 from (*S,S*)-Ni-Cl_3_BPB-*m*-Tyr (100 mg, 141 μmol) as follows. **11** was added to a solution of the starting material and Et_3_N in MeCN (10 mL) and the reaction mixture was left to stand for 1 h before a second portion of **11** and Et_3_N (1 equiv of each) was added. TLC showed incomplete conversion of the starting material after 16 h. The crude material was purified by dry load column chromatography (*R*_*f*_ = 0.18, CH_2_Cl_2_:MeOH = 40:1). Molecular formula (molecular mass): C_34_H_27_Cl_3_FN_3_NiO_6_S (789.71 g/mol). ^1^H NMR (400 MHz, CDCl_3_): δ 8.90 (d, *J* = 2.0 Hz, 1H), 8.17 (d, *J* = 9.3 Hz, 1H), 7.70–7.56 (m, 3H), 7.53–7.43 (m, 2H), 7.40–7.30 (m, 3H), 7.16–7.10 (m, 2H), 7.00–6.95 (m, 1H), 6.82 (d, *J* = 7.7 Hz, 1H), 6.62 (d, *J* = 2.6 Hz, 1H), 4.28–4.17 (m, 2H), 3.25 (dd, *J* = 10.6, 6.5 Hz, 2H), 3.16–3.05 (m, 2H), 2.97 (dd, *J* = 13.8, 6.4 Hz, 1H), 2.70–2.55 (m, 1H), 2.53–2.31 (m, 2H), 2.03–1.88 (m, 2H). ^13^C{^1^H} NMR (101 MHz, CDCl_3_): δ 180.1, 177.6, 171.4, 150.5, 141.2, 139.0, 135.0, 133.8, 133.6, 133.5, 133.1, 132.9, 132.5, 131.2, 130.7, 130.61, 130.56, 129.90, 129.86, 129.5, 127.5, 127.4, 127.1, 126.0, 124.1, 122.8, 120.1, 71.4, 71.2, 63.3, 58.6, 40.0, 31.0, 23.4. *Ortho*- and *meta*-carbons of the phenyl substituent are not equivalent owing to hindered rotation. ^19^F NMR (376 MHz, CDCl_3_): δ 38.15. HR-ESI-MS *m*/*z*: [M + Na]^+^ calcd for C_34_H_27_Cl_3_FN_3_NiO_6_SNa 809.99158; found, 809.99107. Correct isotopic pattern.

#### 4-{(2*S*,3*R*)-1-(4-Fluorophenyl)-3-[(*S*)-3-(4-fluorophenyl)-3-hydroxypropyl]-4-oxoazetidin-2-yl}phenyl Sulfurofluoridate (**12k**)^12^

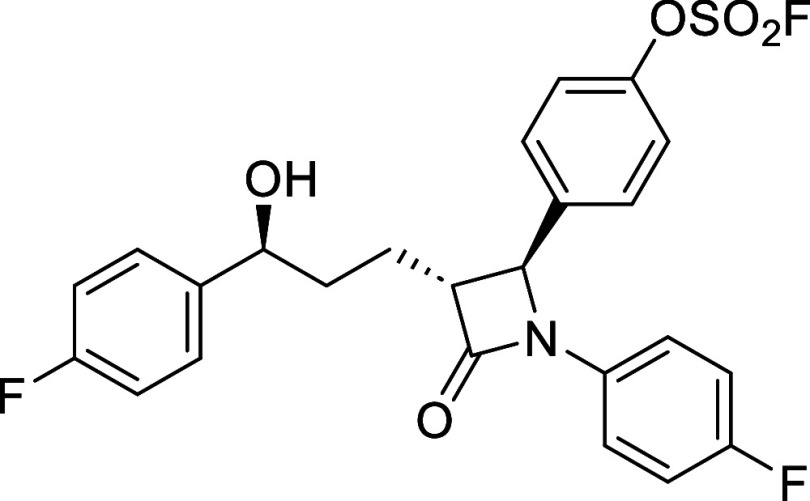



**12k** (90 mg, 183 μmol, 75%, colorless solid) was prepared according to GP 1 from ezetimibe (100 mg, 244 μmol) and purified by column chromatography (*R*_*f*_ = 0.32, EtOAc:*n*-hexane = 1:2). Molecular formula (molecular mass): C_24_H_20_F_3_NO_5_S (491.48 g/mol). ^1^H NMR (400 MHz, CDCl_3_): δ 7.49–7.40 (m, 2H), 7.36 (d, *J* = 8.5 Hz, 2H), 7.33–7.26 (m, 2H), 7.19 (ddt, *J* = 8.0, 5.6, 2.8 Hz, 2H), 7.08–6.90 (m, 4H), 4.72 (t, *J* = 5.8 Hz, 1H), 4.68 (d, *J* = 2.4 Hz, 1H), 3.07 (td, *J* = 7.5, 2.4 Hz, 1H), 2.25 (s, 1H), 2.12–1.84 (m, 4H). ^13^C{^1^H} NMR (101 MHz, CDCl_3_): δ 167.0, 162.4 (d, *J* = 245.8 Hz), 159.3 (d, *J* = 244.2 Hz), 150.0, 140.0 (d, *J* = 3.0 Hz), 138.7, 133.6 (d, *J* = 2.7 Hz), 128.0, 127.5 (d, *J* = 8.2 Hz), 122.1, 118.4 (d, *J* = 7.9 Hz), 116.2 (d, *J* = 22.7 Hz), 115.6 (d, *J* = 21.4 Hz), 73.3, 60.8, 60.5, 36.7, 25.3. ^19^F NMR (376 MHz, CDCl_3_): δ 38.03, −114.62, −117.30. HR-ESI-MS *m*/*z*: [M + Na]^+^ calcd for C_24_H_20_F_3_NO_5_SNa 514.09065; found, 514.09065.

#### (*S*)-4-Ethyl-4-hydroxy-3,14-dioxo-3,4,12,14-tetrahydro-1*H*-pyrano[3′,4’:6,7]indolizino[1,2-*b*]quinolin-9-yl Sulfurofluoridate (**12l**)^8^

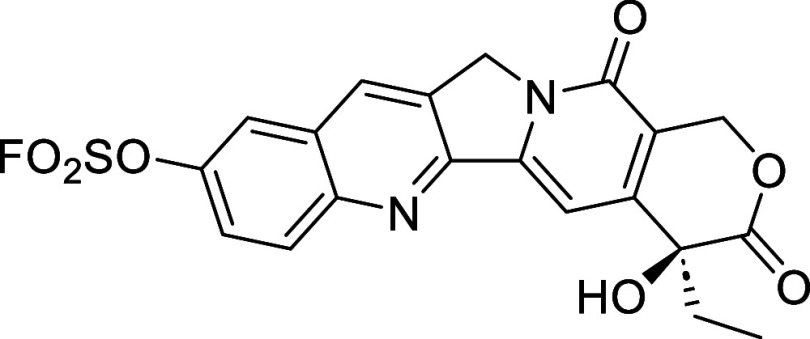



**12l** (68.9 mg, 154 μmol, 37%, green-yellow solid) was prepared according to GP1 from 10-hydroxycamptothecin (150 mg, 412 μmol) using increased amounts of **11** (5.2 equiv) and Et_3_N (6.4 equiv) and purified by column chromatography (*R*_*f*_ = 0.14, EtOAc:*n*-hexane = 2:1). Molecular formula (molecular mass): C_20_H_15_FN_2_O_7_S (446.41 g/mol). ^1^H NMR [400 MHz, (CD_3_)_2_SO]: δ 8.81 (s, 1H), 8.50 (s, 1H), 8.42–8.30 (m, 1H), 8.13–7.97 (m, 1H), 7.37 (d, *J* = 2.9 Hz, 1H), 6.56 (s, 1H), 5.44 (s, 2H), 5.32 (s, 2H), 1.87 (sep, *J* = 7.1 Hz, 2H), 0.88 (t, *J* = 7.3 Hz, 3H). ^13^C{^1^H} NMR [101 MHz, (CD_3_)_2_SO]: δ 172.4, 156.7, 154.3, 149.9, 147.5, 146.9, 144.9, 132.2, 132.0, 131.4, 128.2, 123.7, 120.1, 119.8, 97.3, 72.3, 65.2, 50.3, 30.3, 7.8. ^19^F NMR [376 MHz, (CD_3_)_2_SO]: δ 39.33. HR-ESI-MS *m*/*z*: [M + H]^+^ calcd for C_20_H_16_FN_2_O_7_S 447.06568; found, 447.06623; [M + Na]^+^ calcd for C_20_H_15_FN_2_O_7_SNa 469.04762; found, 469.04838.

#### 4-{3-[2-(Diethylamino)-2-oxoethyl]-5,7-dimethylpyrazolo[1,5-*a*]pyrimidin-2-yl}phenyl Sulfurofluoridate (**12m**)^12^

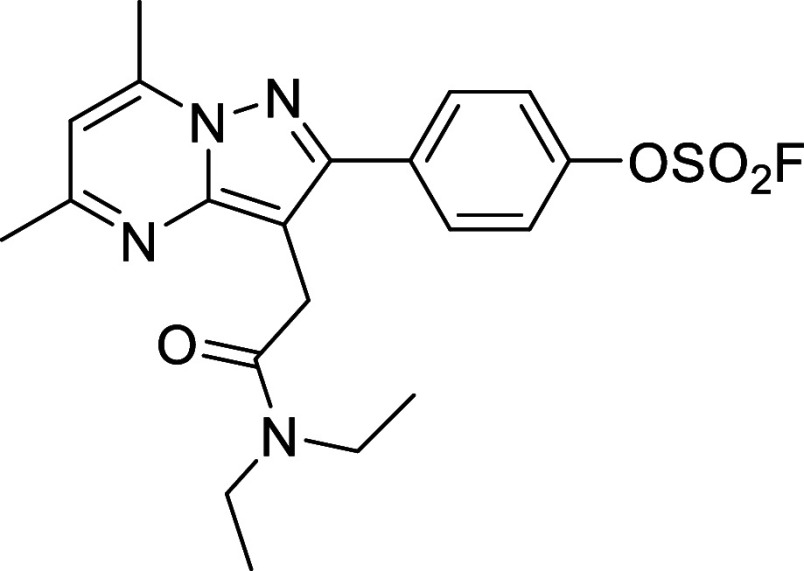



**12m** (73.9 mg, 170 μmol, 60%, colorless solid) was prepared according to GP1 from *N,N*-diethyl-2-{2-(4-hydroxyphenyl)-5,7-dimethylpyrazolo[1,5-*a*]pyrimidin-3-yl}-acetamide (des-Me-DPA-713) (100 mg, 284 μmol; prepared as described in^22^) and purified by column chromatography (*R*_*f*_ = 0.26, CHCl_3_:acetone = 5:1). The reaction was conducted in a mixture of DMF/MeCN (1:1, 6 mL). Molecular formula (molecular mass): C_20_H_23_FN_4_O_4_S (434.49 g/mol). ^1^H NMR (400 MHz, CDCl_3_): δ 8.04 (d, *J* = 8.9 Hz, 2H), 7.42 (d, *J* = 8.1 Hz, 2H), 6.56 (s, 1H), 3.93 (s, 2H), 3.56 (q, *J* = 7.1 Hz, 2H), 3.40 (q, *J* = 7.1 Hz, 2H), 2.74 (s, 3H), 2.55 (s, 3H), 1.25 (t, *J* = 7.1 Hz, 4H), 1.10 (t, *J* = 7.1 Hz, 3H). ^13^C{^1^H} NMR (101 MHz, CDCl_3_): δ 170.0, 158.1, 153.4, 150.1, 147.8, 145.0, 134.8, 130.9, 121.1, 109.0, 101.7, 42.6, 40.9, 27.9, 24.8, 17.0, 14.6, 13.2. ^19^F NMR (376 MHz, CDCl_3_): δ 37.66. HR-ESI-MS *m*/*z*: [M + Na]^+^ calcd for C_20_H_23_FN_4_O_4_SNa 457.13163; found, 457.13162; [M + H]^+^ calcd for C_20_H_24_FN_4_O_4_S 435.14968; found, 435.14983.

#### 2,3,5,6-Tetrafluorophenyl 3-{4-[(fluorosulfonyl)oxy]phenyl}propanoate (**12n**)

##### 2,3,5,6-Tetrafluorophenyl 3-(4-Hydroxyphenyl)propanoate^23^

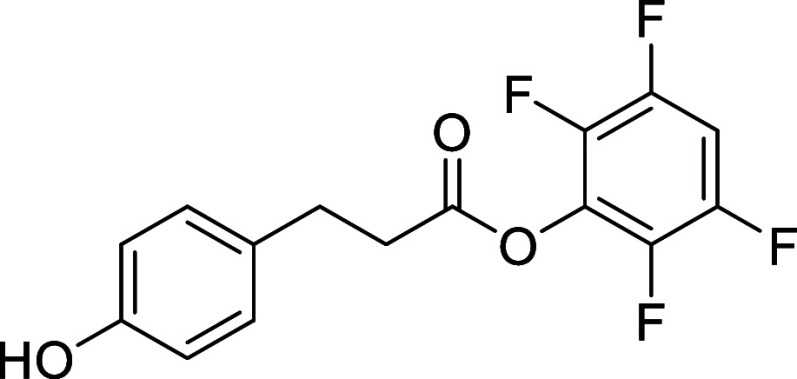



*N*-[3-(Dimethylamino)propyl]-*N*′-ethylcarbodiimide hydrochloride (1.2 g, 6.28 mmol, 1.04 equiv) was added to an ice-cold solution of (4-hydroxyphenyl)propionic acid (1.00 g, 6.02 mmol, 1.0 equiv) and 2,3,5,6-tetrafluorophenol (1.1 g, 6.02 mmol, 1.0 equiv) in CH_2_Cl_2_ (20 mL) and the reaction mixture was stirred for 15 min. The cooling bath was removed and the mixture was stirred for another 16 h. The mixture was then concentrated under reduced pressure and the residue was taken up into Et_2_O and H_2_O (50 mL of each). The organic phase was separated, washed with H_2_O (3 × 20 mL) and brine (2 × 20 mL), dried, and concentrated under reduced pressure. The resulting crude product was purified by column chromatography (*R*_*f*_ = 0.15, EtOAc:*n*-hexane = 1:2.3) to afford the title compound (1.44 g, 4.83 mmol, 76%) as a colorless liquid. Molecular formula (molecular mass): C_15_H_10_F_4_O_3_ (314.24 g/mol). ^1^H NMR (400 MHz, CDCl_3_): δ 7.16–7.08 (m, 2H), 7.04–6.93 (m, 1H), 6.82–6.73 (m, 2H), 4.87 (d, *J* = 27.4 Hz, 1H), 3.09–2.86 (m, 4H). ^13^C{^1^H} NMR (101 MHz, CDCl_3_): δ 169.0, 154.4, 146.1 (ddd, *J* = 240.5, 7.1, 4.0 Hz), 142.3–139.2 (m), 131.8, 129.6, 115.6, 103.3 (t, *J* = 22.8 Hz), 35.5, 30.0. *C*_Ar_-O was not observed. ^19^F NMR (376 MHz, CDCl_3_): δ −139.02 (dd, *J* = 21.8, 9.8 Hz), −152.84 (dd, *J* = 21.8, 9.8 Hz). LR-ESI-MS *m*/*z*: [M + NH_4_]^+^ calcd for C_15_H_14_NF_4_O_3_ 332.09; found, 332.22.

##### 2,3,5,6-Tetrafluorophenyl 3-{4-[(Fluorosulfonyl)oxy]phenyl}propanoate (**12n**)

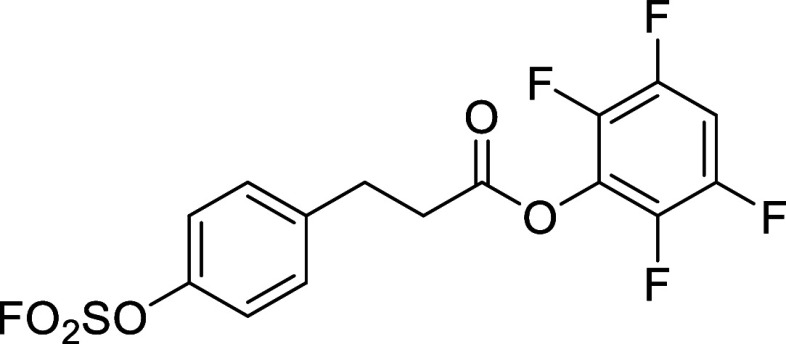



**12n** was prepared according to a modification of GP1 as follows. **11** (2.02 g, 6.40 mmol, 1.4 equiv) was added to a solution of 2,3,5,6-tetrafluorophenyl (4-hydroxyphenyl)propanoate (1.44 g, 4.58 mmol) and Et_3_N (1.09 mL, 0.79 g, 7.82 mmol, 1.7 equiv) in MeCN (20 mL) and the reaction mixture was vigorously stirred for 16 h. The mixture was then concentrated under reduced pressure, the residue was taken up into Et_2_O and H_2_O (50 mL of each), and the insoluble tar and aqueous fraction were discarded. The organic phase was washed with H_2_O (3 × 20 mL) and brine (2 × 20 mL), dried, and concentrated under reduced pressure. The resulting crude product was purified by column chromatography (*R*_*f*_ = 0.52, EtOAc:*n*-hexane = 1:15) to afford, after low-temperature (−20 °C) recrystallization from pentane, **12n** (0.99 g, 2.50 mmol, 55%) as a colorless solid. Molecular formula (molecular mass): C_15_H_9_F_5_O_5_S (396.28 g/mol). ^1^H NMR (400 MHz, CDCl_3_): δ 7.40–7.34 (m, 2H), 7.34–7.28 (m, 2H), 7.00 (tt, *J* = 9.9, 7.1 Hz, 1H), 3.14 (t, *J* = 7.4 Hz, 2H), 3.05–2.98 (m, 2H). ^13^C{^1^H} NMR (101 MHz, CDCl_3_): δ 168.4, 149.0, 146.2 (ddd, *J* = 250.5, 7.0, 4.0 Hz), 142.1–139.0 (m), 130.4, 121.3, 103.5 (t, *J* = 22.8 Hz), 34.8, 30.1. *C*_Ar_-O was not observed. ^19^F NMR (376 MHz, CDCl_3_): δ 37.42, −138.79 (dd, *J* = 21.7, 9.8 Hz), −152.93 (dd, *J* = 21.7, 9.8 Hz). Elemental analysis: Calcd for C_15_H_9_F_5_O_5_S: C, 45.46%; H, 2.29%; S, 8.09%. Found: C, 45.85 ± 0.02%; H, 2.31 ± 0.04%; S, 8.16 ± 0.00% (*n* = 2).

#### Methyl(phenyl)sulfamoyl Fluoride (**13a**)^8^

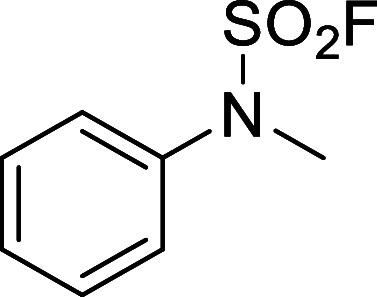



**13a** (247 mg, 1.31 mmol, 70%, yellow oil) was prepared according to GP2 from *N*-methylaniline (200 mg, 1.87 mmol). After HPLC indicated full conversion, the reaction solvent was removed and the residue was taken up into CH_2_Cl_2_. The organic phase was washed with H_2_O, dried, and the solvent was removed. The crude material thus obtained was purified by RP chromatography. Conditions: column: FlashPure Select C_18_ 12 g (Büchi Labortechnik GmbH, Essen, Germany); eluent: 0–15 min: 40% MeCN, 15–17 min: 40 → 80% MeCN, 17–22 min: 80 → 100% MeCN; flow rate: 20 mL/min; detection: UV, λ = 210, 220, 254, 280 nm. Molecular formula (molecular mass): C_7_H_8_FNO_2_S (189.20 g/mol). ^1^H NMR (400 MHz, CDCl_3_): δ = 7.50–7.33 (m, 5H), 3.44 (d, *J* = 2.2 Hz, 3H). ^13^C{^1^H} NMR (101 MHz, CDCl_3_): δ 140.0, 130.0, 129.1, 126.7, 40.8. ^19^F NMR (376 MHz, CDCl_3_): δ 42.33. HR-EI-MS *m*/*z*: [M]^•+^ calcd for C_7_H_8_FNO_2_S 189.0254; found, 189.0254.

#### 1,2,3,4-Tetrahydroisoquinoline-2-sulfonyl Fluoride (**13b**)^24^

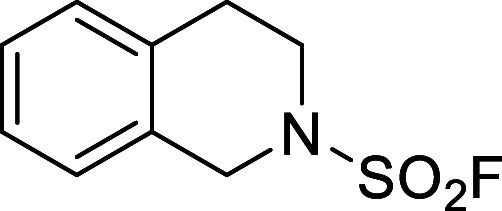



**13b** (200 mg, 929 μmol, 83%, pale rose solid) was prepared according to GP2 from 1,2,3,4-tetrahydroisoquinoline (150 mg, 1.23 mmol). After TLC indicated full conversion, the reaction solvent was removed, the residue was dissolved in Et_2_O and the organic phase was washed with H_2_O. After removal of the solvent, no further purification was required. Molecular formula (molecular mass): C_9_H_10_FNO_2_S (215.24 g/mol). ^1^H NMR (400 MHz, CDCl_3_): δ 7.31–7.24 (m, 2H), 7.24–7.18 (m, 1H), 7.17–7.10 (m, 1H), 4.67 (s, 2H), 3.78 (td, *J* = 6.0, 1.9 Hz, 2H), 3.05 (t, *J* = 6.0 Hz, 2H). ^13^C{^1^H} NMR (101 MHz, CDCl_3_): δ 132.6, 130.4, 129.2, 127.6, 127.0, 126.3, 48.3, 45.1, 28.1. ^19^F NMR (376 MHz, CDCl_3_): δ 40.96. HR-EI-MS *m*/*z*: [M]^•+^ calcd for C_9_H_10_FNO_2_S 215.0411; found, 215.0407.

#### Indoline-1-sulfonyl Fluoride (**13c**)^24^

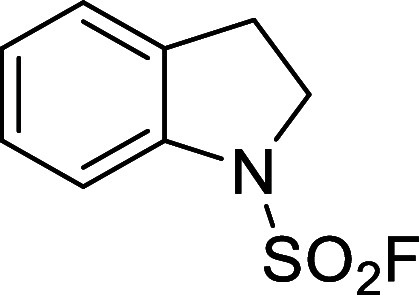



**13c** (143 mg, 711 μmol, 85%, pale rose solid) was prepared according to GP2 from indoline (100 mg, 839 μmol) and purified by filtration over a silica plug (*R*_*f*_ = 0.45, EtOAc:*n*-hexane = 1:6). Molecular formula (molecular mass): C_8_H_8_FNO_2_S (201.22 g/mol). ^1^H NMR (400 MHz, CDCl_3_): δ 7.54–7.37 (m, 1H), 7.30–7.19 (m, 2H), 7.17–7.05 (m, 1H), 4.14 (td, *J* = 8.4, 2.5 Hz, 2H), 3.21 (t, *J* = 8.4 Hz, 2H). ^13^C{^1^H} NMR (101 MHz, CDCl_3_): δ 139.9, 131.0, 128.3, 125.6, 125.3, 114.7, 51.7, 28.1. ^19^F NMR (376 MHz, CDCl_3_): δ 39.12. HR-EI-MS *m*/*z*: [M]^•+^ calcd for C_8_H_8_FNO_2_S 201.0254; found, 201.0252.

#### Isoindoline-2-sulfonyl Fluoride (**13d**)

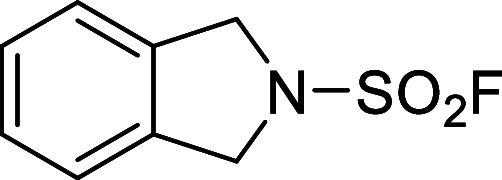



**11** (488 mg, 1.55 mmol, 1.6 equiv) was added to a suspension of isoindoline × HCl (150 mg, 0.96 mmol) in CH_2_Cl_2_ (4 mL) followed by dropwise addition of Et_3_N (134 μL, 0.96 mmol, 1.0 equiv). After TLC indicated full conversion, the reaction mixture was filtered over a plug of silica. The crude product was purified by column chromatography (*R*_*f*_ = 0.43, EtOAc:*n*-hexane = 1:10) to afford the title compound **13d** (191 mg, 0.95 mmol, 98%) as a colorless solid. Molecular formula (molecular mass): C_8_H_8_FNO_2_S (201.22 g/mol). ^1^H NMR (400 MHz, CDCl_3_): δ 7.42–7.32 (m, 2H), 7.31–7.26 (m, 2H), 4.86 (d, *J* = 2.4 Hz, 4H). ^13^C{^1^H} NMR (101 MHz, CDCl_3_): δ 134.8, 128.5, 122.9, 55.0. ^19^F NMR (376 MHz, CDCl_3_): δ 37.93. HR-EI-MS *m*/*z*: [M]^•+^ calcd for C_8_H_8_FNO_2_S 201.0254; found, 201.0251.

#### 4-Benzylpiperidine-1-sulfonyl Fluoride (**13e**)^8^

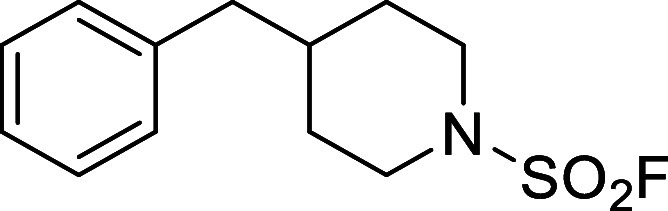



**13e** (285 mg, 1.11 mmol, 97%, colorless solid) was prepared according to GP2 from 4-benzylpiperidine (200 mg, 1.14 mmol). After TLC indicated full conversion, the reaction mixture was diluted with Et_2_O (10 mL) and washed with H_2_O (10 mL) and brine (10 mL). The organic phase was dried and concentrated under reduced pressure and the product was purified by filtration over a silica plug (*R*_*f*_ = 0.42, EtOAc:*n*-hexane = 1:6). Molecular formula (molecular mass): C_12_H_16_FNO_2_S (257.32 g/mol). ^1^H NMR (400 MHz, CDCl_3_): δ 7.37–7.27 (m, 2H), 7.25–7.19 (m, 1H), 7.17–7.09 (m, 2H), 3.91 (dt, *J* = 12.7, 2.3 Hz, 2H), 2.94 (tt, *J* = 12.7, 3.0 Hz, 2H), 2.59 (d, *J* = 7.0 Hz, 2H), 1.84–1.61 (m, 3H), 1.39 (qd, *J* = 12.8, 4.2 Hz, 2H). ^13^C{^1^H} NMR (101 MHz, CDCl_3_): δ 139.4, 129.2, 128.6, 126.4, 47.6, 42.7, 37.2, 30.8. ^19^F NMR (376 MHz, CDCl_3_): δ 40.17. HR-EI-MS *m*/*z*: [M]^•+^ calcd for C_12_H_16_FNO_2_S 257.0880; found, 257.0878.

#### 4-Hydroxypiperidyl-1-sulfonyl Fluoride (**13f**)

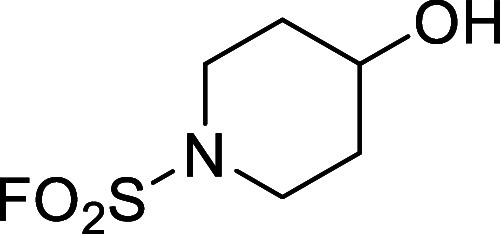



**13f** (160 mg, 876 μmol, 89%, colorless solid) was prepared according to GP2 from 4-hydroxypiperidine (100 mg, 989 μmol) and purified by filtration over a silica plug (*R*_*f*_ = 0.46, EtOAc:*n*-hexane = 2:1). Molecular formula (molecular mass): C_5_H_10_FNO_3_S (183.20 g/mol). ^1^H NMR (400 MHz, CDCl_3_): δ 4.19–3.88 (m, 1H), 3.87–3.61 (m, 2H), 3.55–3.25 (m, 2H), 2.12–1.90 (m, 2H), 1.78–1.65 (m, 2H), 1.76 (br s, 1H). ^13^C{^1^H} NMR (101 MHz, CDCl_3_): δ 65.0, 44.1, 32.6. ^19^F NMR (376 MHz, CDCl_3_): δ 41.07. HR-EI-MS *m*/*z*: [M-H_2_O]^•+^ calcd for C_5_H_8_FNO_2_S 165.0254; found, 165.0253.

#### 6-Hydroxy-1,2,3,4-tetrahydroisoquinoline-2-sulfonyl Fluoride (**13g**)

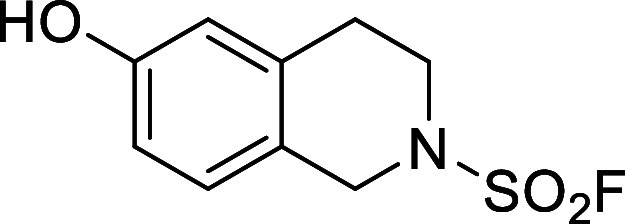



**13g** (205 mg, 887 μmol, 88%, colorless oil) was prepared according to GP2 from 1,2,3,4-tetrahydroisoquinolin-6-ol (150 mg, 1.01 mmol) and purified by filtration over a silica plug (*R*_*f*_ = 0.37, EtOAc:*n*-hexane = 1:2). Molecular formula (molecular mass): C_9_H_10_FNO_3_S (231.24 g/mol). ^1^H NMR (400 MHz, CDCl_3_): δ 6.97 (d, *J* = 8.4 Hz, 1H), 6.72 (dd, *J* = 8.3, 2.6 Hz, 1H), 6.65 (d, *J* = 2.6 Hz, 1H), 4.97 (s, 1H), 4.56 (s, 2H), 3.71 (td, *J* = 6.1, 1.9 Hz, 2H), 2.95 (t, *J* = 6.1 Hz, 2H). ^13^C{^1^H} NMR (101 MHz, CDCl_3_): δ 154.9, 134.2, 127.6, 122.6, 115.4, 114.5, 47.9, 44.9, 28.2. ^19^F NMR (376 MHz, CDCl_3_): δ 41.05. HR-EI-MS *m*/*z*: [M]^+^ calcd for C_9_H_10_FNO_3_S 231.0360; found, 231.0361.

#### Phenylsulfamoyl Fluoride (**13h**)^8^

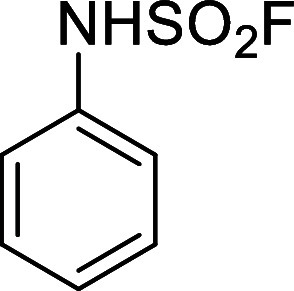



A solution of aniline (147 μL, 1.61 mmol) in CH_2_Cl_2_ (1.5 mL) was added to an ice-cooled suspension of **11** (556 mg, 1.77 mmol, 1.1 equiv) in CH_2_Cl_2_ (4 mL). After TLC indicated full conversion, CH_2_Cl_2_ (10 mL) was added and the solution was washed with 0.1 m HCl (3 × 10 mL) and brine (20 mL). The organic phase was dried and the solvent was removed under reduced pressure to afford the title compound **13h** (196 mg, 1.12 mmol, 70%) as a pale red liquid. Molecular formula (molecular mass): C_6_H_6_FNO_2_S (175.18 g/mol). ^1^H NMR (400 MHz, CDCl_3_): δ 7.42 (ddd, *J* = 8.0, 6.2, 1.8 Hz, 2H), 7.36–7.24 (m, 3H), 7.16 (br s, 1H). ^13^C{^1^H} NMR (101 MHz, CDCl_3_): δ 134.0, 129.9, 127.6, 123.1. ^19^F NMR (376 MHz, CDCl_3_): δ 50.90. HR-EI-MS *m*/*z*: [M]^•+^ calcd for C_6_H_6_FNO_2_S 175.0098; found, 175.0096.

#### Phenyliminodisulfonyl Difluoride (**13i**)^8^

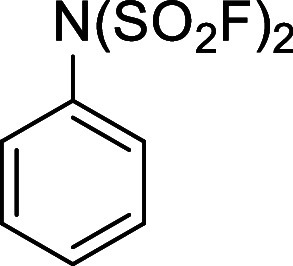



**11** (1.26 g, 4.03 mmol, 2.5 equiv) was added to an ice-cooled solution of aniline (147 μL, 1.61 mmol) in MeCN (4 mL). The mixture was stirred for 10 min before Et_3_N (112 μL, 805 μmol, 0.5 equiv) was added. After TLC indicated full conversion, silica was added, the solvent was removed, and the residue was filtered over a plug of silica (*R*_*f*_ = 0.50, EtOAc:*n*-hexane = 1:10) to afford the title compound **13i** (322 mg, 1.26 mmol, 78%) as a colorless solid. Molecular formula (molecular mass): C_6_H_5_F_2_NO_4_S_2_ (257.23 g/mol). ^1^H NMR (400 MHz, CDCl_3_): δ 7.69–7.54 (m, 3H), 7.54–7.47 (m, 2H). ^13^C{^1^H} NMR (101 MHz, CDCl_3_): δ 133.2, 132.5, 130.9, 129.3. ^19^F NMR (376 MHz, CDCl_3_): δ 56.32. HR-EI-MS *m*/*z*: [M]^•+^ calcd for C_6_H_5_F_2_NO_4_S_2_ 256.9623; found, 256.9621.

#### (4-Fluorobenzyl)sulfamoyl Fluoride (**13j**)^25^

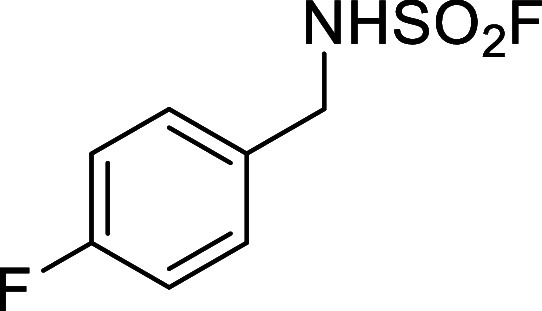



**11** (0.68 g, 2.10 mmol, 1.05 equiv) was added to a solution of 4-fluorobenzylamine (0.228 mL, 0.25 g, 2.0 mmol) in MeCN (2 mL). An exothermic reaction was observed. The reaction mixture was stirred for 40 min and taken up into Et_2_O/H_2_O (30 mL of each). The organic layer was separated, washed with H_2_O (3 × 20 mL) and brine (2 × 10 mL), dried, and successively concentrated under atmospheric pressure and at 750 mbar. The crude product was purified by fast column chromatography (*R*_*f*_ = 0.38, CH_2_Cl_2_:*n*-pentane = 1:1; **13j** is not completely stable on silica). The product containing fractions were pooled and concentrated under atmospheric pressure to afford, after drying at 100 mbar and ambient temperature, **13j** (0.31 g, 75%, 1.5 mmol) as a volatile colorless liquid. Molecular formula (molecular mass): C_7_H_7_F_2_NO_2_S (207.19 g/mol). ^1^H NMR (400 MHz, CDCl_3_): δ 7.32 (ddd, *J* = 8.1, 5.0, 2.5 Hz, 2H), 7.11–7.04 (m, 2H), 5.37–5.11 (br s, 1H), 4.42 (d, *J* = 5.9 Hz, 1H). ^13^C{^1^H} NMR (101 MHz, CDCl_3_): δ 163.1 (d, *J* = 248.2 Hz), 130.8 (dd, *J* = 2.9, 1.1 Hz), 130.2 (d, *J* = 8.5 Hz), 116.3 (d, *J* = 21.8 Hz), 48.0. ^19^F NMR (376 MHz, CDCl_3_): δ 51.25, −112.67. LR-ESI-MS *m*/*z*: [M–H]^−^ calcd for C_7_H_6_F_2_NO_2_S 206.01; found, 206.06. HR-EI-MS *m*/*z*: [M]^•+^ calcd for C_7_H_7_F_2_NO_2_S 207.0160; found, 207.0159.

#### 4-(2-Chlorodibenzo[*b*,*f*][1,4]oxazepin-11 yl)piperazine-1-sulfonyl Fluoride (**13k**)^8^

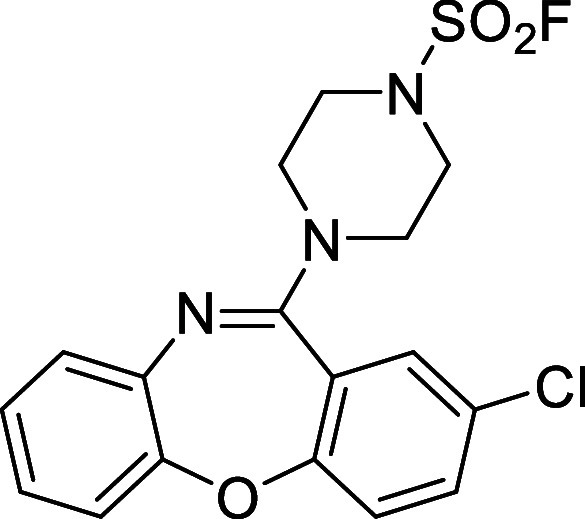



**13k** (235 mg, 594 μmol, 93%, colorless solid) was prepared according to GP2 from amoxapine (200 mg, 637 μmol) and purified by filtration over a silica plug (*R*_*f*_ = 0.38, EtOAc:*n*-hexane = 1:4). Molecular formula (molecular mass): C_17_H_15_ClFN_3_O_3_S (395.83 g/mol). ^1^H NMR (400 MHz, CDCl_3_): δ 7.44 (dd, *J* = 8.7, 2.6 Hz, 1H), 7.31 (d, *J* = 2.6 Hz, 1H), 7.22 (d, *J* = 8.7 Hz, 1H), 7.19–7.08 (m, 3H), 7.08–7.02 (m, 1H), 3.62 (d, *J* = 35.1 Hz, 8H). ^13^C{^1^H} NMR (101 MHz, CDCl_3_): δ 159.6, 158.4, 151.8, 139.5, 133.3, 130.8, 128.7, 127.3, 126.1, 125.6, 124.5, 123.1, 120.4, 46.7, 46.5. ^19^F NMR (376 MHz, CDCl_3_): δ 39.17. HR-ESI-MS *m*/*z*: [M + H]^+^ calcd for C_17_H_16_ClFN_3_O_3_S 396.0579; found, 396.0578.

#### *tert*-Butyl {2-[1-(Fluorosulfonyl)-1*H*-indol-3-yl]ethyl}carbamate (**13l**)

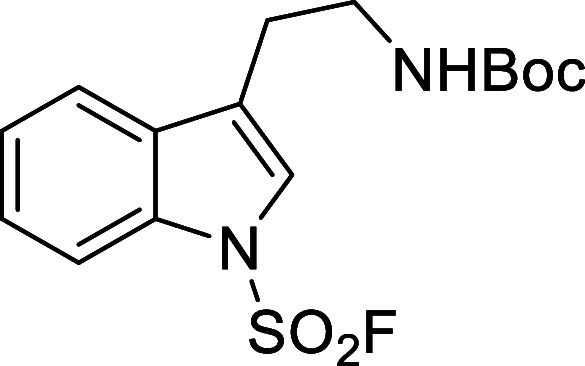



**11** (531 mg, 1.69 mmol, 1.1 equiv) was added to a solution of *tert*-butyl [2-(1*H*-indol-3-yl)ethyl]carbamate (400 mg, 1.54 mmol) and DBU (505 μL, 3.38 mmol, 2.2 equiv) in CH_2_Cl_2_. Conversion of the starting material was monitored by HPLC. After 1 h, a second portion of **11** (241 mg, 768 μmol, 0.5 equiv) was added, but no further conversion was observed after another 2 h. Therefore, silica was added, the solvent was removed, and the residue was purified by column chromatography with solid loading (*R*_*f*_ = 0.26, EtOAc:*n*-hexane = 1:4) to afford the title compound **13l** (72.0 mg, 210 μmol, 14%) as a colorless solid. Molecular formula (molecular mass): C_15_H_19_FN_2_O_4_S (342.39 g/mol). ^1^H NMR (400 MHz, CDCl_3_): δ 8.02–7.78 (m, 1H), 7.63 (d, *J* = 7.6 Hz, 1H), 7.53–7.31 (m, 2H), 7.28–7.23 (m, 1H), 4.67 (s, 1H), 3.47 (q, *J* = 6.7 Hz, 2H), 2.93 (t, *J* = 6.7 Hz, 2H), 1.44 (s, 9H). ^13^C{^1^H} NMR (101 MHz, CDCl_3_): δ 156.0, 135.4, 130.8, 126.2, 124.8, 123.0, 122.2, 120.2, 113.9, 79.7, 39.9, 28.5, 25.8. ^19^F NMR (376 MHz, CDCl_3_): δ 53.90. HR-ESI-MS *m*/*z*: [M + Na]^+^ calcd for C_15_H_19_FN_2_O_4_SNa 365.0942; found, 365.0948.

#### 1-(Fluorosulfonyl)piperidine-4-carboxylic Acid (**13m**)

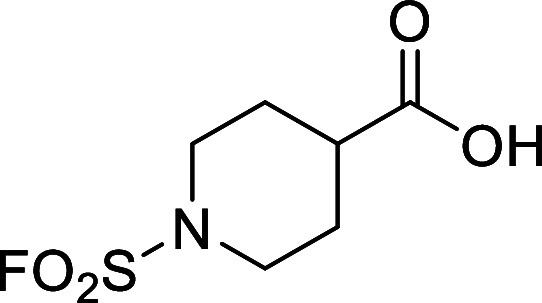



**13m** (62.6 mg, 298 μmol, 39%, colorless solid) was prepared according to GP2 from isonipecotic acid (100 mg, 774 μmol) and purified by column chromatography (*R*_*f*_ = 0.21, CH_2_Cl_2_:MeOH = 30:1). Molecular formula (molecular mass): C_6_H_10_FNO_4_S (211.21 g/mol). ^1^H NMR (400 MHz, CD_3_OD): δ 3.93–3.74 (m, 2H), 3.26–3.11 (m, 2H), 2.65–2.47 (m, 1H), 2.14–1.97 (m, 2H), 1.90–1.68 (m, 2H). ^13^C{^1^H} NMR (101 MHz, CD_3_OD): δ 177.2, 47.6, 40.5, 28.2. ^19^F NMR (376 MHz, CD_3_OD): δ 38.16. HR-EI-MS *m*/*z*: [M-CO_2_H]^•+^ calcd for C_5_H_9_FNO_2_S 166.0333; found, 166.0332.

## Data Availability

The data underlying this study are available in the published article and its Supporting Information.

## Supporting Information Available

Appearance and preparation of 1*H*-imidazole-1-sulfonyl fluoride (**10**) and desmethyl SuFEx-IT (**11**) and NMR-spectra for all prepared compounds (PDF)

## Abbreviations

AISF: [4-(acetylamino)phenyl]imidodisulfuryl difluoride
(CD_3_)_2_SO: hexadeuterodimethyl sulfoxide
CD_3_CN: trideuteroacetonitrile
CDCl_3_: deuterochloroform
DBU: 1,8-diazabicyclo[5.4.0]undec-7-ene
desmethyl SuFEx-IT: 1-(fluorosulfonyl)-3-methyl-1*H*-imidazole trifluoromethanesulfonate
FO_2_S-: fluorosulfuryl
HR-EI-MS: high-resolution electron ionization mass spectrometry
HR-ESI-MS: high-resolution electrospray ionization mass spectrometry
LR-EI-MS: low-resolution electron ionization mass spectrometry
LR-ESI-MS: low-resolution electrospray ionization mass spectrometry
MeOTf: methyl triflate
MS: mass spectrometry
MSDI: 3-(imidazole-1-sulfonyl)-1-methyl-3*H*-imidazole-1-ium trifluoromethanesulfonate
NMR: nuclear magnetic resonance
SDI: 1,1′-sulfonyldiimidazole
SO_2_Cl_2_: sulfuryl chloride
SO_2_F_2_: sulfuryl fluoride
SuFEx: sulfur(VI)-fluoride exchange
SuFEx-IT: 1-(fluorosulfonyl)-2,3-dimethyl-1*H*-imidazole-3-ium trifluoromethanesulfonate
THF-*d*_8_: octadeuterotetrahydrofuran
TLC: thin-layer chromatography
LR-EI-MS: low-resolution electron ionization mass spectrometry
