# Supplementary material for: Desmethyl SuFEx-IT: SO2F2-Free Synthesis and Evaluation as a Fluorosulfurylating Agent
Source: J Org Chem. 2024 Feb 22;89(6):3821–33. doi: 10.1021/acs.joc.3c02643 (PMC10949248; doi:10.1021/acs.joc.3c02643)

Supporting Information for

Desmethyl SuFEx-IT: SO_2F_2-free Synthesis and Evaluation as Fluorosulfurylating Agent

Jan Bertram[a], Felix Neumaier[a,b], Boris D. Zlatopolskiy[a,b,c], Bernd Neumaier[a,b,c*]

[a] Forschungszentrum Jülich GmbH, Institute of Neuroscience and Medicine, Nuclear Chemistry (INM-5), Wilhelm-Johnen-Straße, 52425 Jülich, Germany, j.bertram@fz-juelich.de (JB), f.neumaier@fz-juelich.de (FN)

[b] University of Cologne, Faculty of Medicine and Cologne University Hospital, Institute of Radiochemistry and Experimental Molecular Imaging, Kerpener Straße 62, 50937 Cologne, Germany, boris.zlatopolskiy@uk-koeln.de (BDZ)

[c] Max Planck Institute for Metabolism Research, Gleueler Straße 50, 50931 Cologne, Germany

* correspondence: b.neumaier@fz-juelich.de

Table of contents:

Appearance of **10** and desmethyl SuFEx-IT (**11**) S3
Production of 1*H*-imidazole-1-sulfonyl fluoride (**10**) S3
NMR Spectra S4
1,1'-Sulfonylbis(2-methyl-1*H*-imidazole) (**5**) S4
2,3-Dimethyl-1-[(2-methyl-1*H*-imidazol-1-yl)sulfonyl]-1*H*-imidazol-3-ium trifluoromethanesulfonate (**6**) S5
2-Methyl-1*H*-imidazole-1-sulfonyl fluoride (**3**) S7
1-(Fluorosulfonyl)-2,3-dimethyl-1*H*-imidazol-3-ium trifluoromethanesulfonate (SuFEx-IT, **4**) S9
1,1′-Sulfonyldiimidazole (SDI, **8**) S11
3-(Imidazole-1-sulfonyl)-1-methyl-3*H*-imidazol-1-ium trifluoromethanesulfonate (MSDI, **9**) S13
1*H*-Imidazole-1-sulfonyl fluoride (**10**) S15
1-(Fluorosulfonyl)-3-methyl-1*H*-imidazole trifluoromethanesulfonate (desmethyl SuFEx-IT, **11**) ... S17
4-Bromophenyl sulfurofluoridate (**12a**) S19
3-Methoxyphenyl sulfurofluoridate (**12b**) S21
4-Formylphenyl sulfurofluoridate (**12c**) S23
Quinolin-8-yl sulfurofluoridate (**12d**) S25
4-[(4-Acetamidophenyl)ethynyl]phenyl sulfurofluoridate (**12e**) S27
4-(4,4,5,5-Tetramethyl-1,3,2-dioxaborolan-2-yl)phenyl sulfurofluoridate (**12f**) S29
2-Mercaptobenzo[d]thiazol-6-yl sulfurofluoridate (**12g**) S31
±-α-Tocopherol-derived sulfurofluoridate (**12h**) S33
4-{[(2*S*,3*R*,4*S*,5*S*,6*R*)-3,4,5-Trihydroxy-6-(hydroxymethyl)tetrahydro-2*H*-pyran-2-yl]oxy}phenyl sulfurofluoridate (**12i**) S35
(*S,S*)-Ni-Cl_3BPB-*m*-Tyr S37
(*S,S*)-Ni-Cl_3BPB-*m*-Tyr sulfurofluoridate (**12j**) S38
4-{(2*S*,3*R*)-1-(4-Fluorophenyl)-3-[(*S*)-3-(4-fluorophenyl)-3-hydroxypropyl]-4-oxoazetidin-2-yl}phenyl sulfurofluoridate (**12k**) S40
(*S*)-4-Ethyl-4-hydroxy-3,14-dioxo-3,4,12,14-tetrahydro-1*H*-pyrano[3',4':6,7] indolizino[1,2-b]quinolin-9-yl sulfurofluoridate (**12l**) S42
4-{3-[2-(Diethylamino)-2-oxoethyl]-5,7-dimethylpyrazolo[1,5-a]pyrimidin-2-yl}phenyl sulfurofluoridate (**12m**) S44
2,3,5,6-Tetrafluorophenyl 3-(4-hydroxyphenyl)propanoate S46
2,3,5,6-Tetrafluorophenyl 3-{4-[(fluorosulfonyl)oxy]phenyl}propanoate (**12n**) S48
Methyl(phenyl)sulfamoyl fluoride (**13a**) S50
1,2,3,4-Tetrahydroisoquinoline-2-sulfonyl fluoride (**13b**) S52
Indoline-1-sulfonyl fluoride (**13c**) S54
Isoindoline-2-sulfonyl fluoride (**13d**) S56
4-Benzylpiperidine-1-sulfonyl fluoride (**13e**) S58
4-Hydroxypiperidyl-1-sulfonyl fluoride (**13f**) S60
6-Hydroxy-1,2,3,4-tetrahydroisoquinoline-2-sulfonyl fluoride (**13g**) S62
Phenylsulfamoyl fluoride (**13h**) S64
Phenyliminodisulfonyl difluoride (**13i**) S66
(4-Fluorobenzyl)sulfamoyl fluoride (**13j**) S68
4-(2-Chlorodibenzo[b,f][1,4]oxazepin-11-yl)piperazine-1-sulfonyl fluoride (**13k**) S70
tert-Butyl {2-[1-(fluorosulfonyl)-1*H*-indol-3-yl]ethyl}carbamate (**13l**) S72
1-(Fluorosulfonyl)piperidine-4-carboxylic acid (**13m**) S74

Appearance of 10 and desmethyl SuFEx-IT (11)

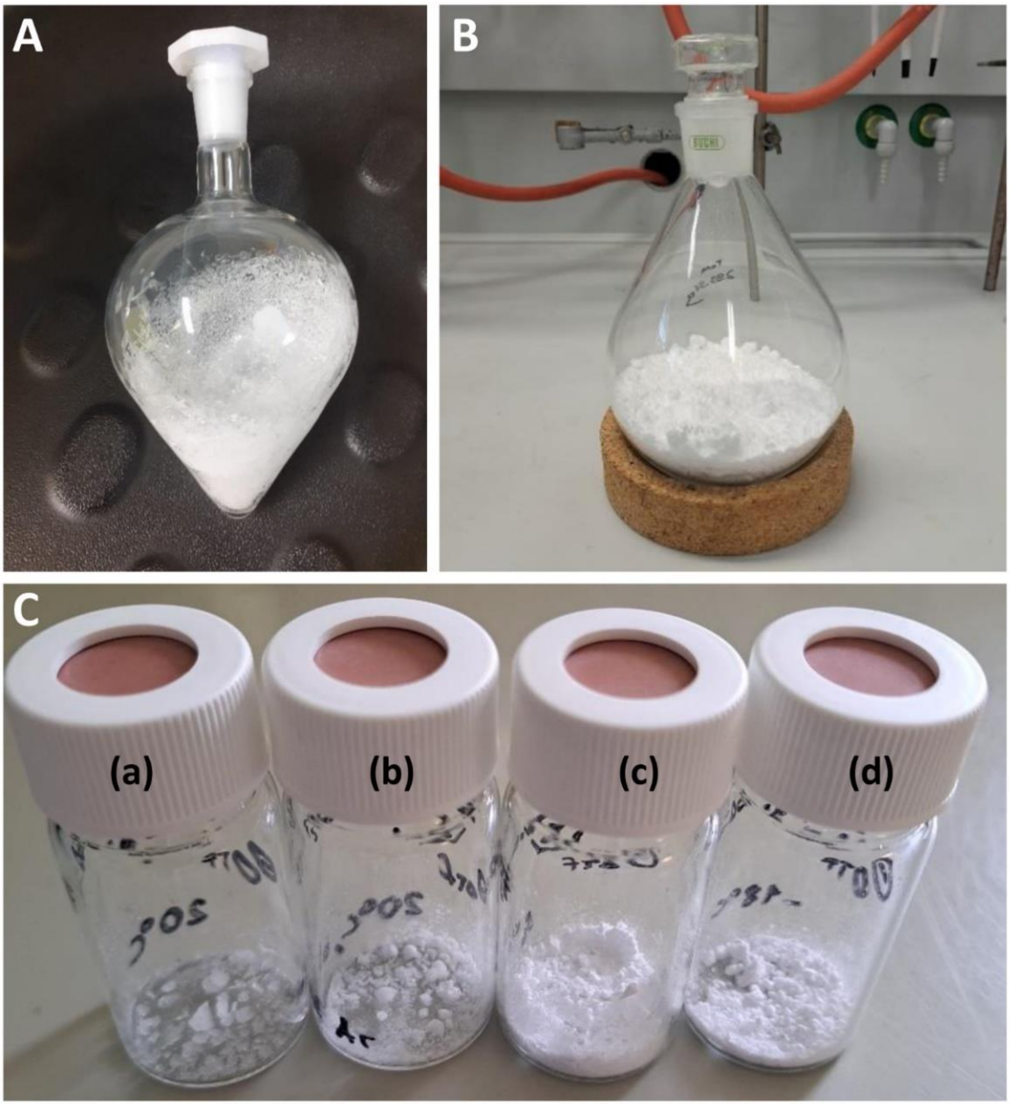

*Figure S1: A) 1H-Imidazole-1-sulfonyl fluoride (**10**). B) 116 g batch of desmethyl SuFEx-IT (**11**). C) Appearance of desmethyl SuFEx-IT after 162 days storage under different conditions: a) 23 °C under air, b) 23 °C under argon, c) 4 °C under argon, d) –18 °C under argon.*

Production of 1*H*-imidazole-1-sulfonyl fluoride (10)

Table S1: Dependency of yield on the amount of KHF_2.

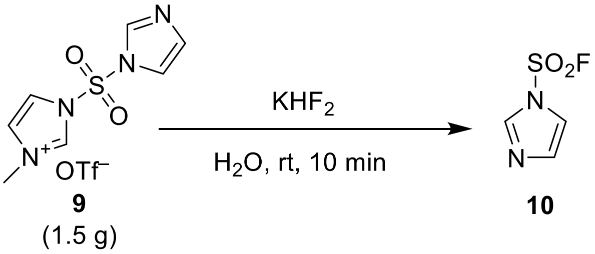

KHF_2 [eq.]	0.50	0.75	1.00
Yield [%]	66	77[a]	75

[a] Note that a roughly 10% higher yield (88%) was obtained when the same reaction conditions were applied on a decagram scale, as described in the main text.

NMR Spectra

1,1'-Sulfonylbis(2-methyl-1*H*-imidazole) (5)

^{1}H NMR (400 MHz, $CDCl_3$)

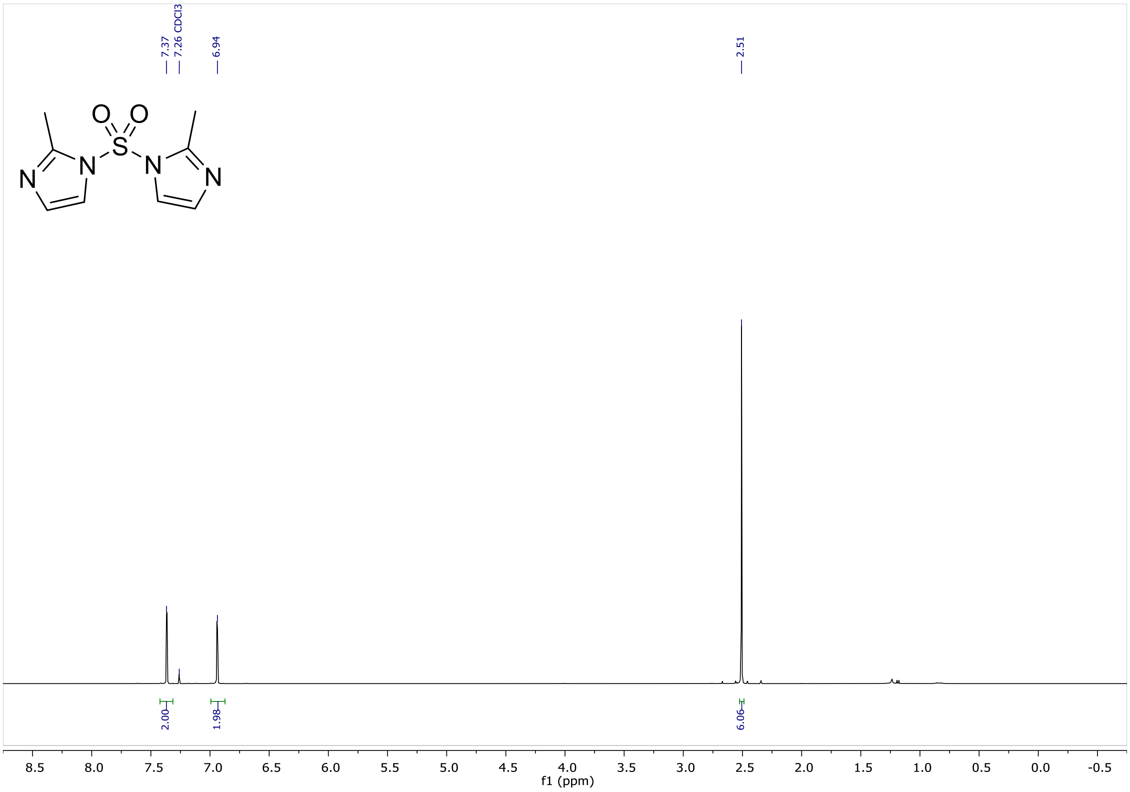

$^{13}C\{^{1}H\}$ NMR (101 MHz, $CDCl_3$)

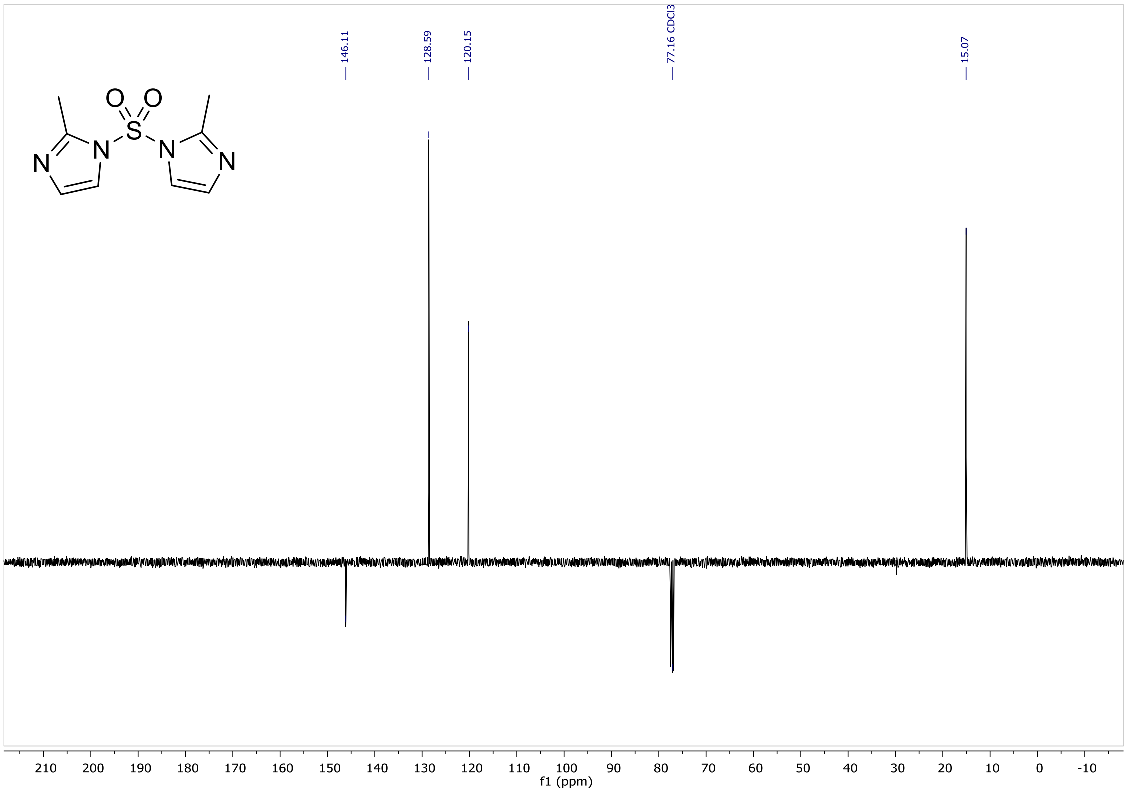

2,3-Dimethyl-1-[(2-methyl-1*H*-imidazol-1-yl)sulfonyl]-1*H*-imidazol-3-ium trifluoromethanesulfonate (6)

^{1}H NMR (400 MHz, CD_3CN)

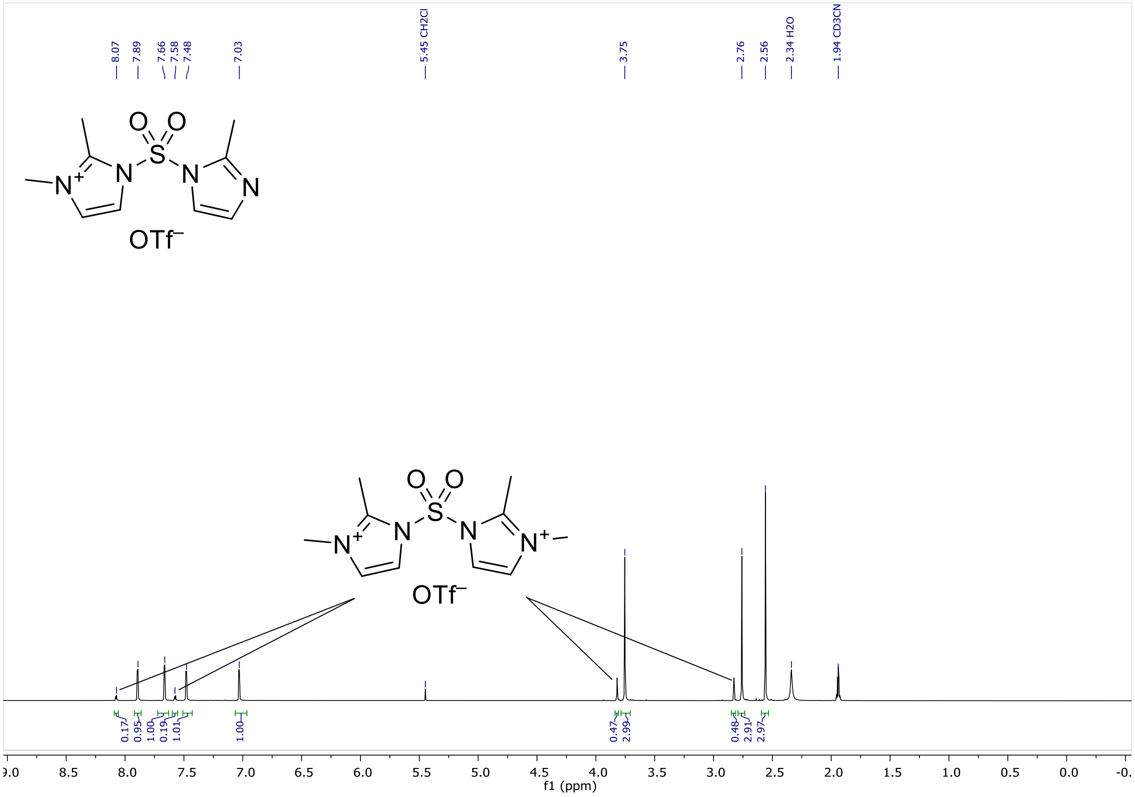

$^{13}C\{^{1}H\}$ NMR (101 MHz, CD_3CN)

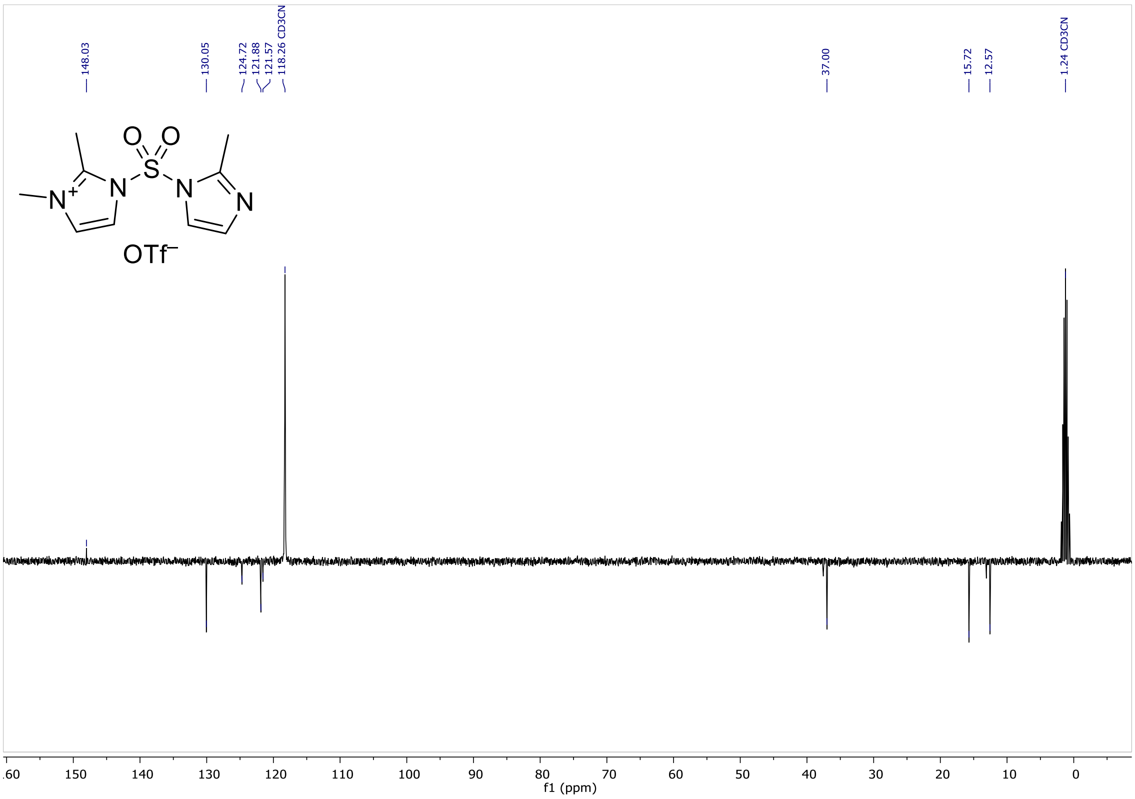

^{19}F NMR (376 MHz, CD_3CN)

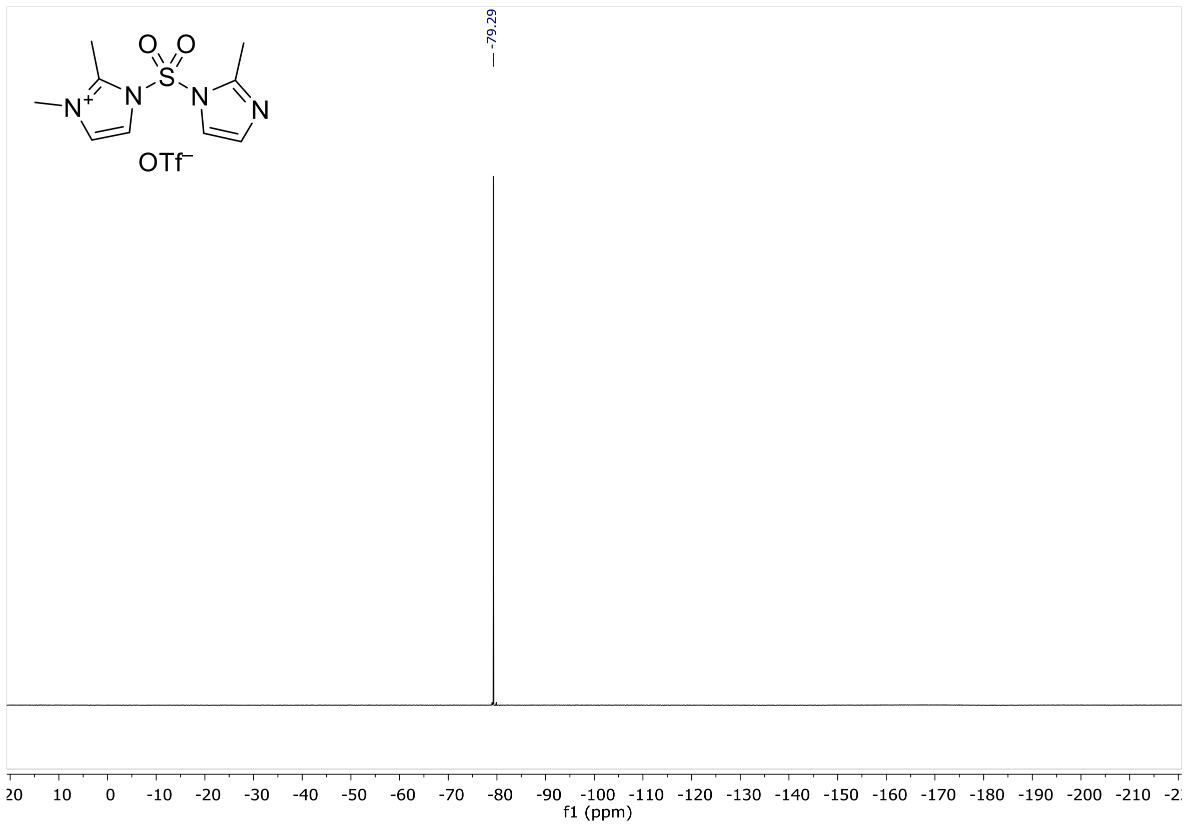

2-Methyl-1*H*-imidazole-1-sulfonyl fluoride (3)

^{1}H NMR (400 MHz, $CDCl_3$)

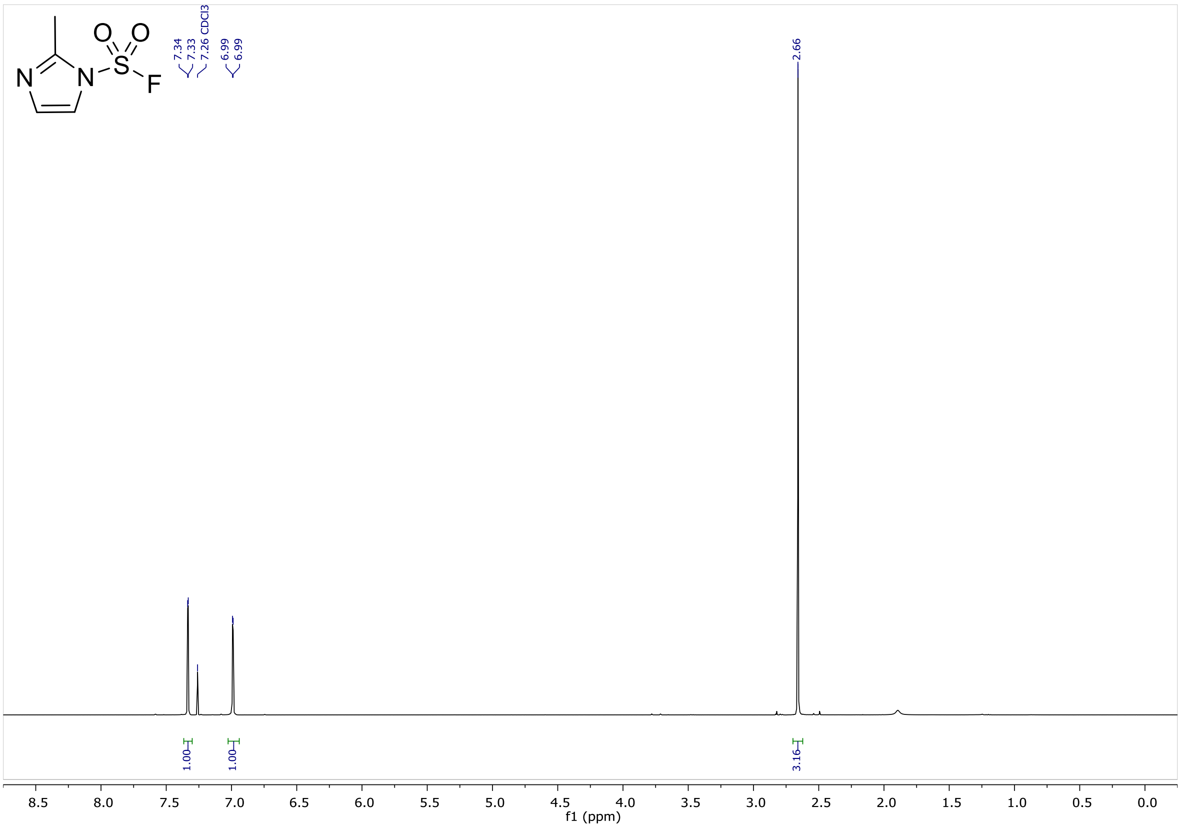

$^{13}C\{^1H\}$ NMR (101 MHz, $CDCl_3$)

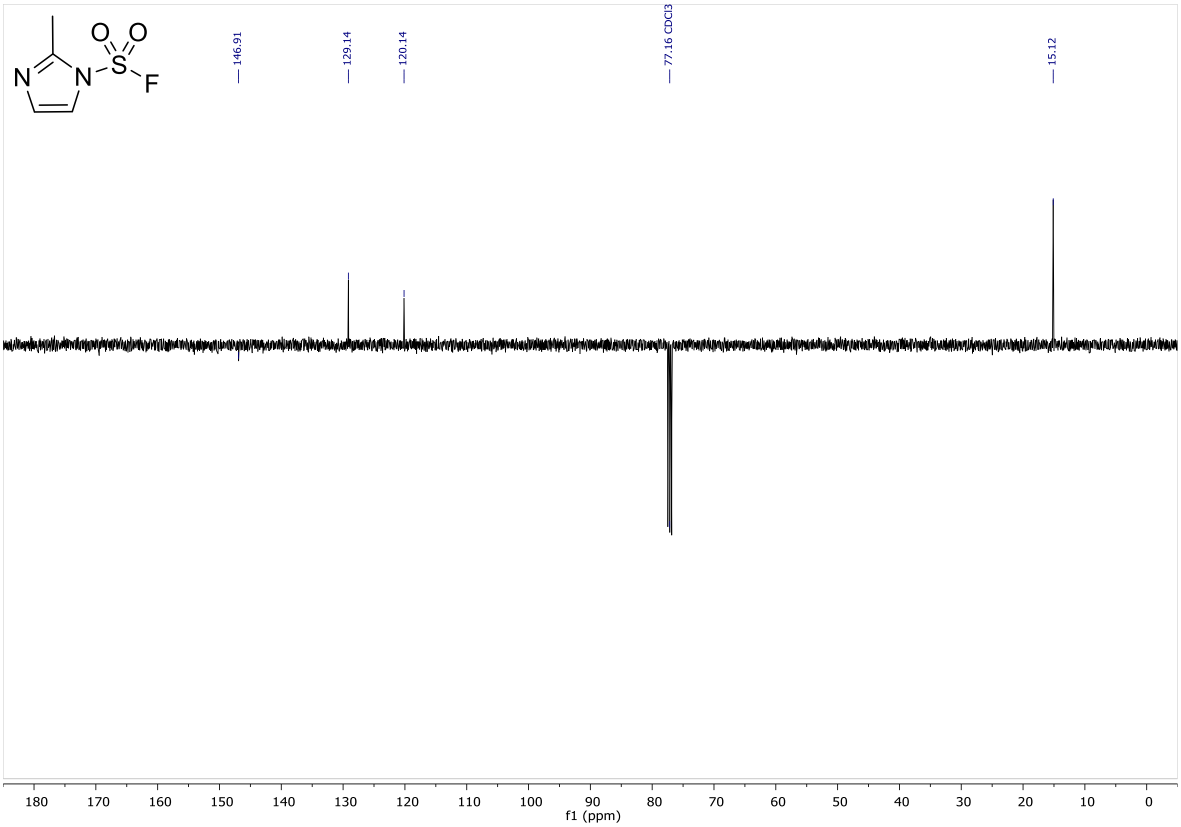

^{19}F NMR (376 MHz, $CDCl_3$)

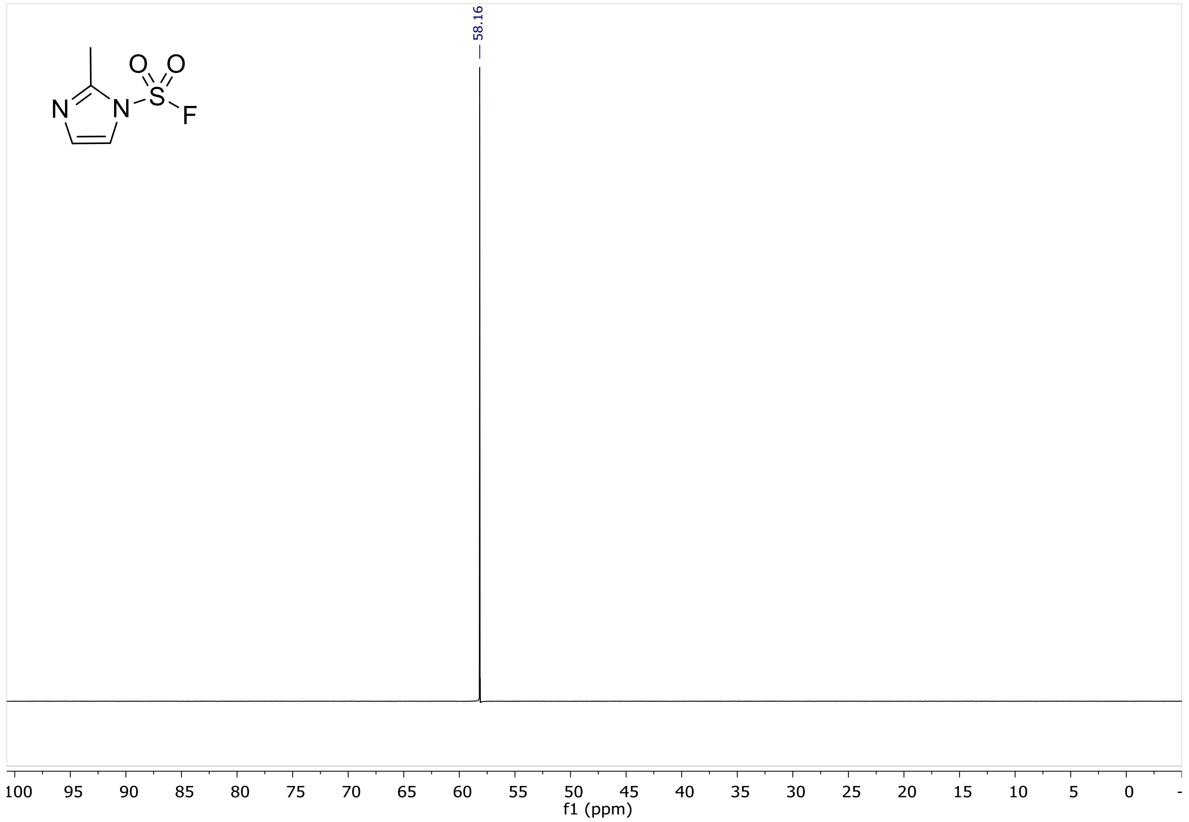

1-(Fluorosulfonyl)-2,3-dimethyl-1*H*-imidazol-3-ium trifluoromethanesulfonate (SuFEx-IT, 4)

^{1}H NMR (400 MHz, CD_3CN)

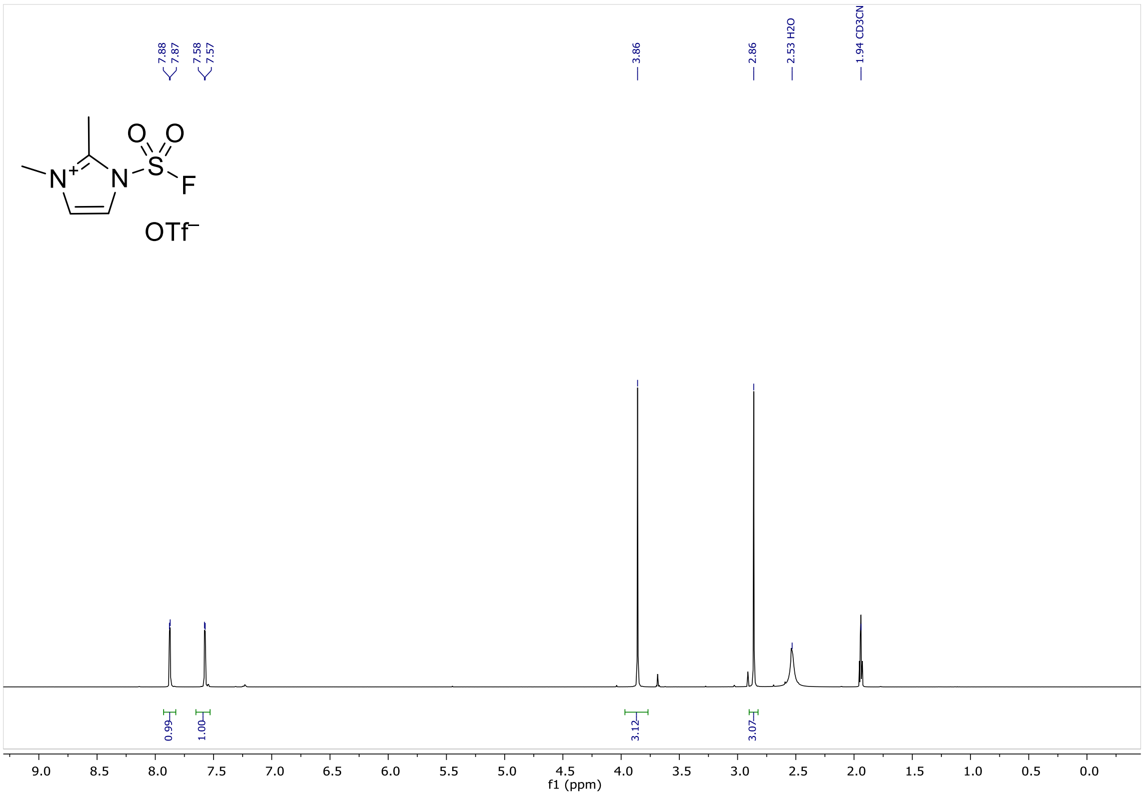

^{13}C{^{1}H} NMR (101 MHz, CD_3CN)

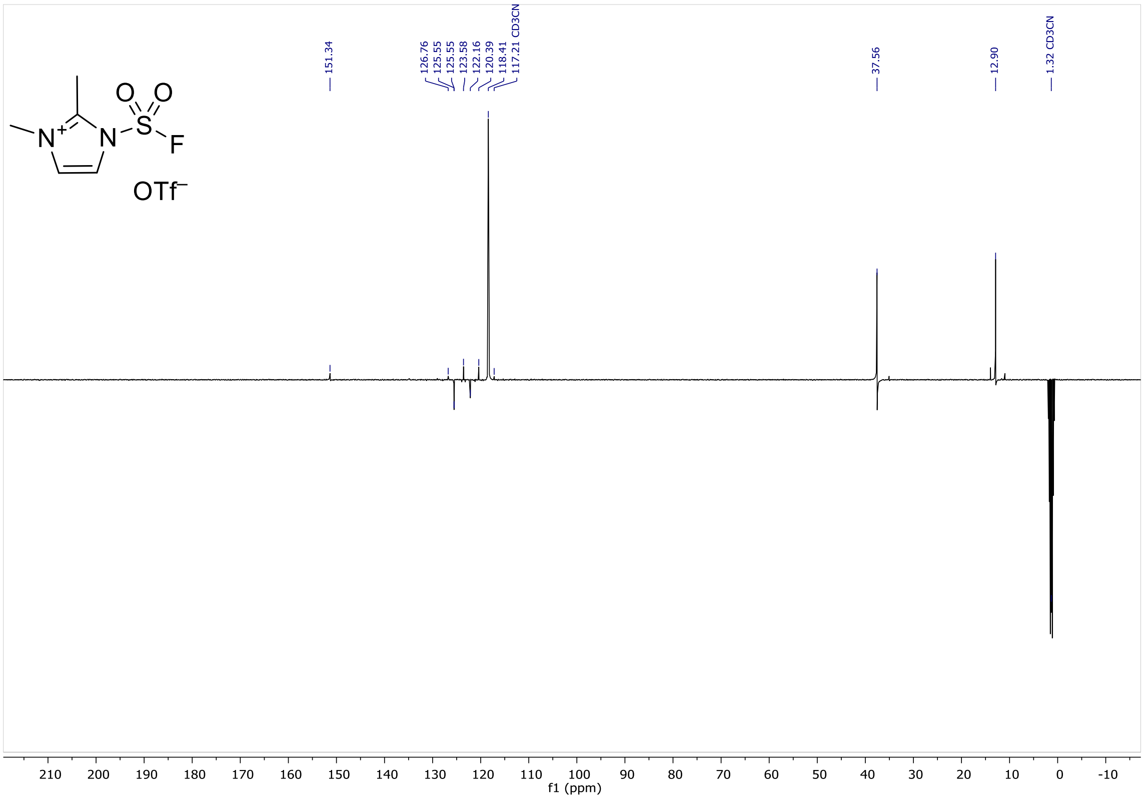

^{19}F NMR (376 MHz, CD_3CN)

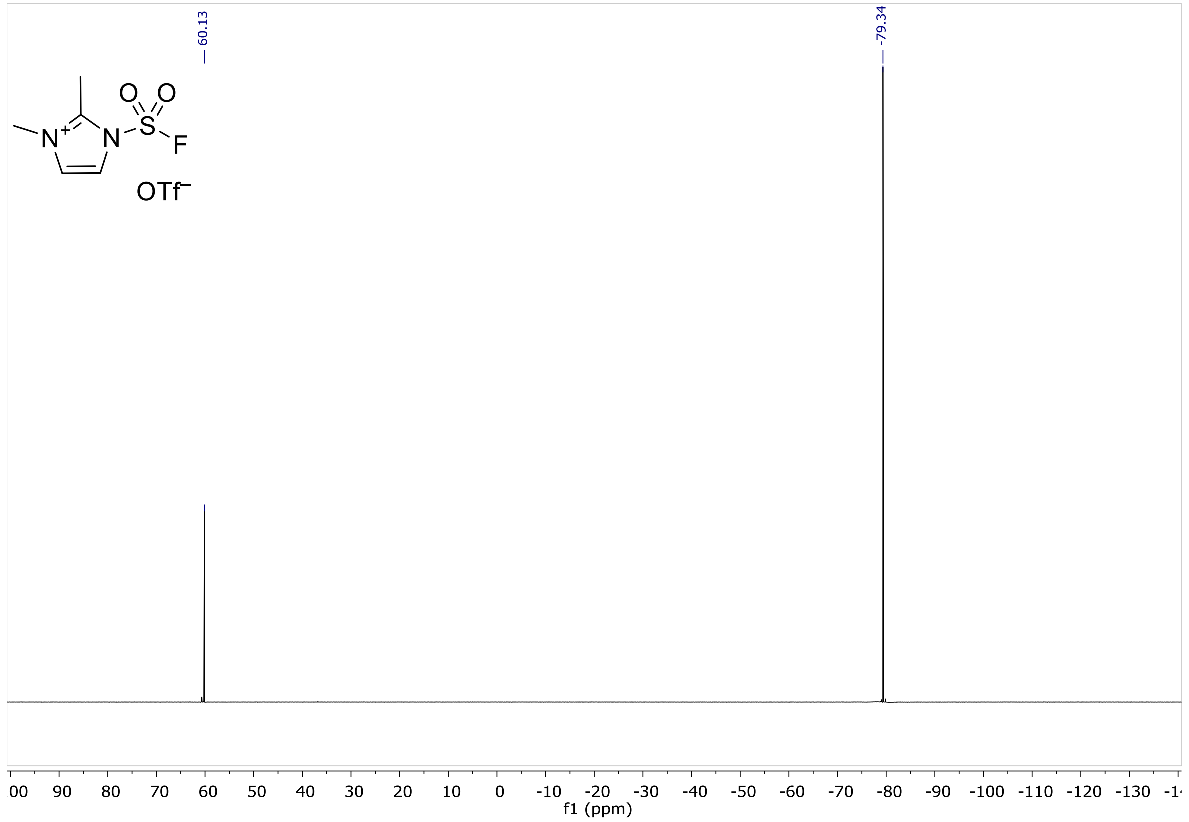

1,1′-Sulfonyldiimidazole (SDI, 8)

^{1}H NMR (400 MHz, $CDCl_3$)

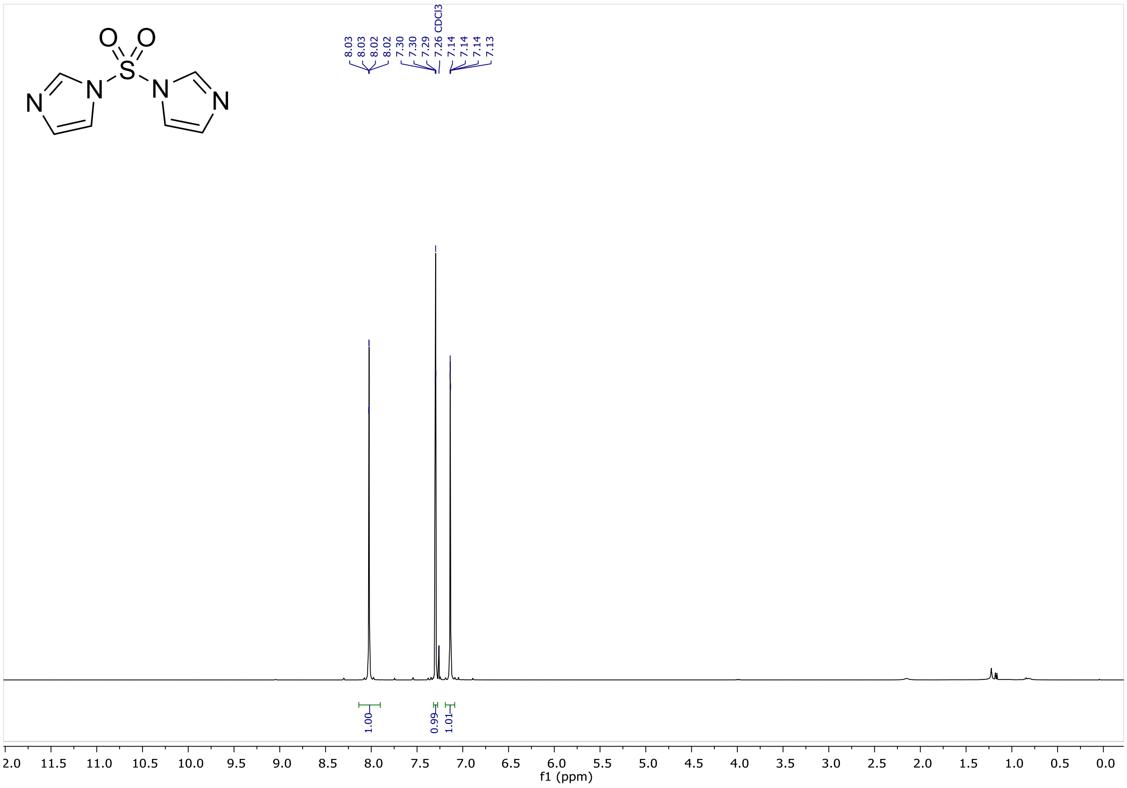

$^{13}C\{^{1}H\}$ NMR (101 MHz, $CDCl_3$)

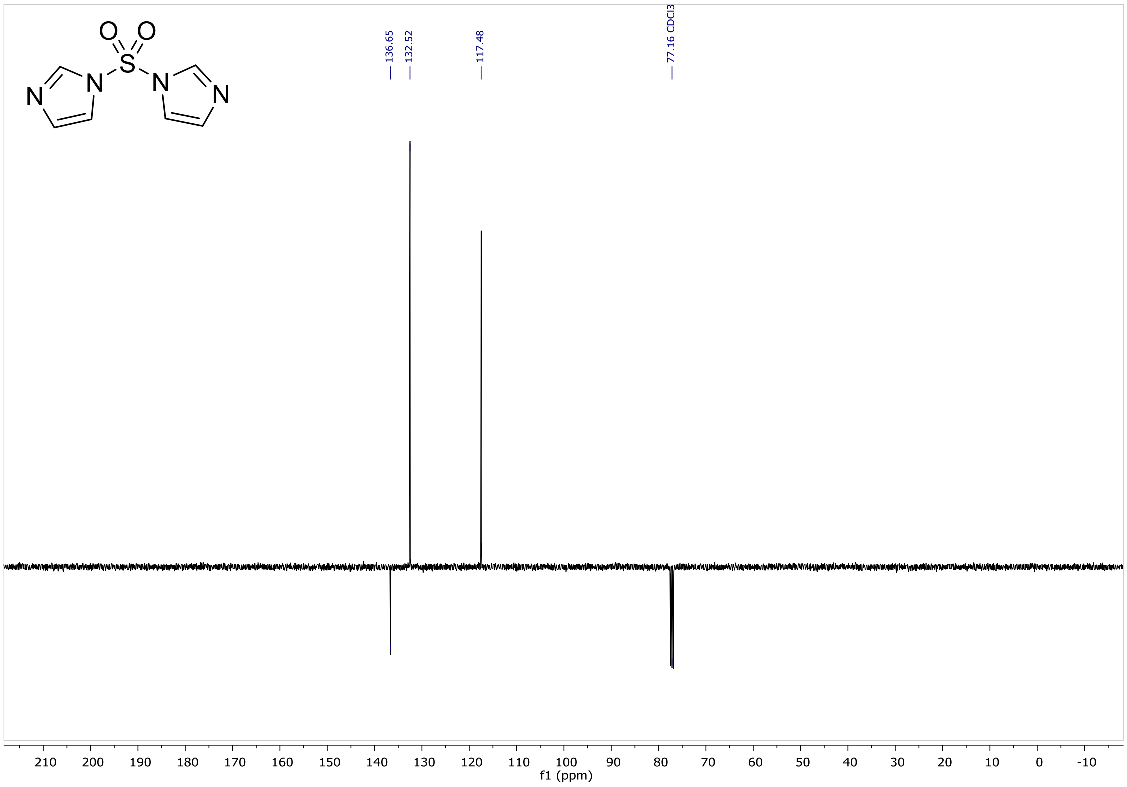

HSQC NMR (400 MHz/101 MHz, $CDCl_3$)

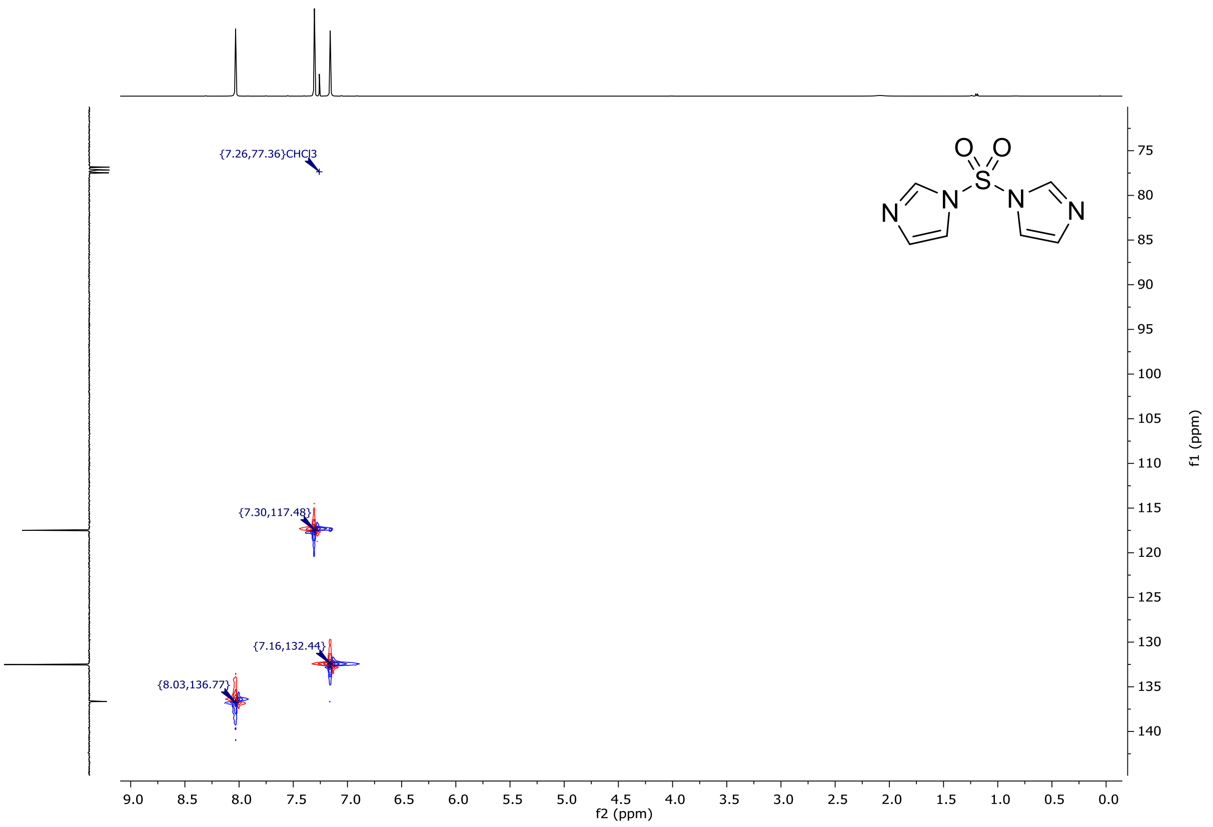

3-(Imidazole-1-sulfonyl)-1-methyl-3*H*-imidazol-1-ium trifluoromethanesulfonate (MSDI, 9)

^{1}H NMR (400 MHz, CD_3CN)

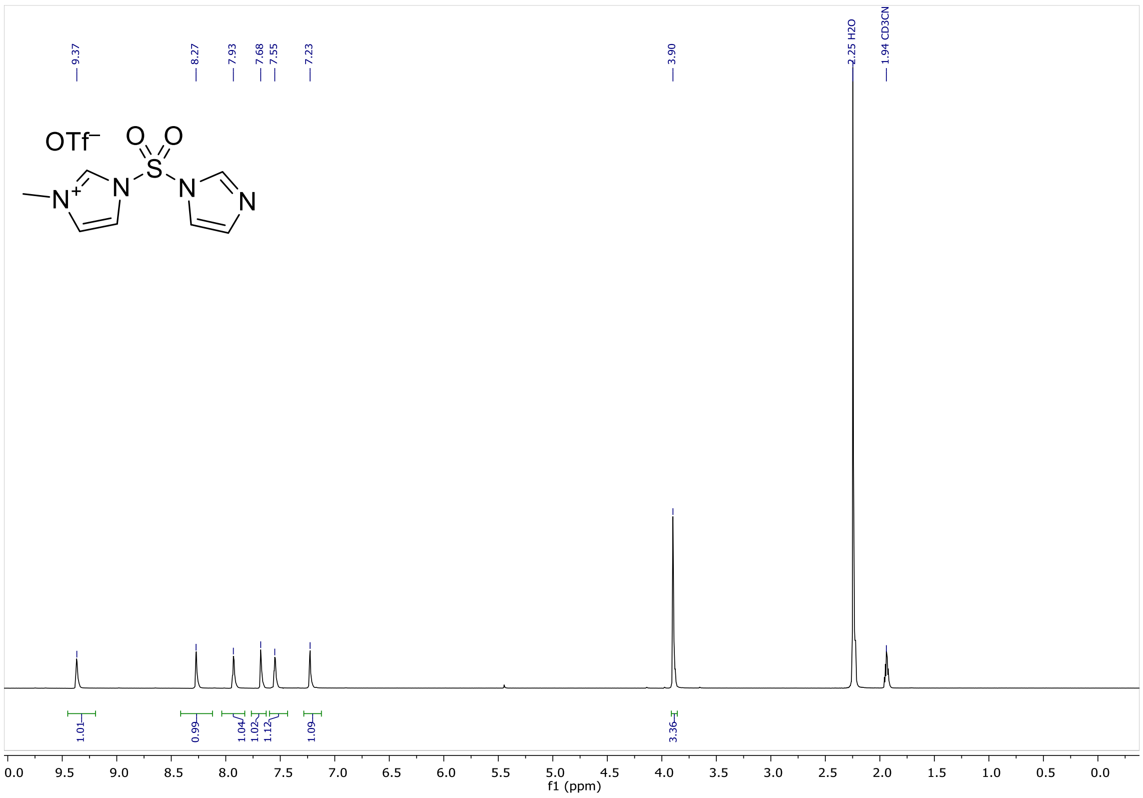

^{13}C{^{1}H} NMR (101 MHz, CD_3CN)

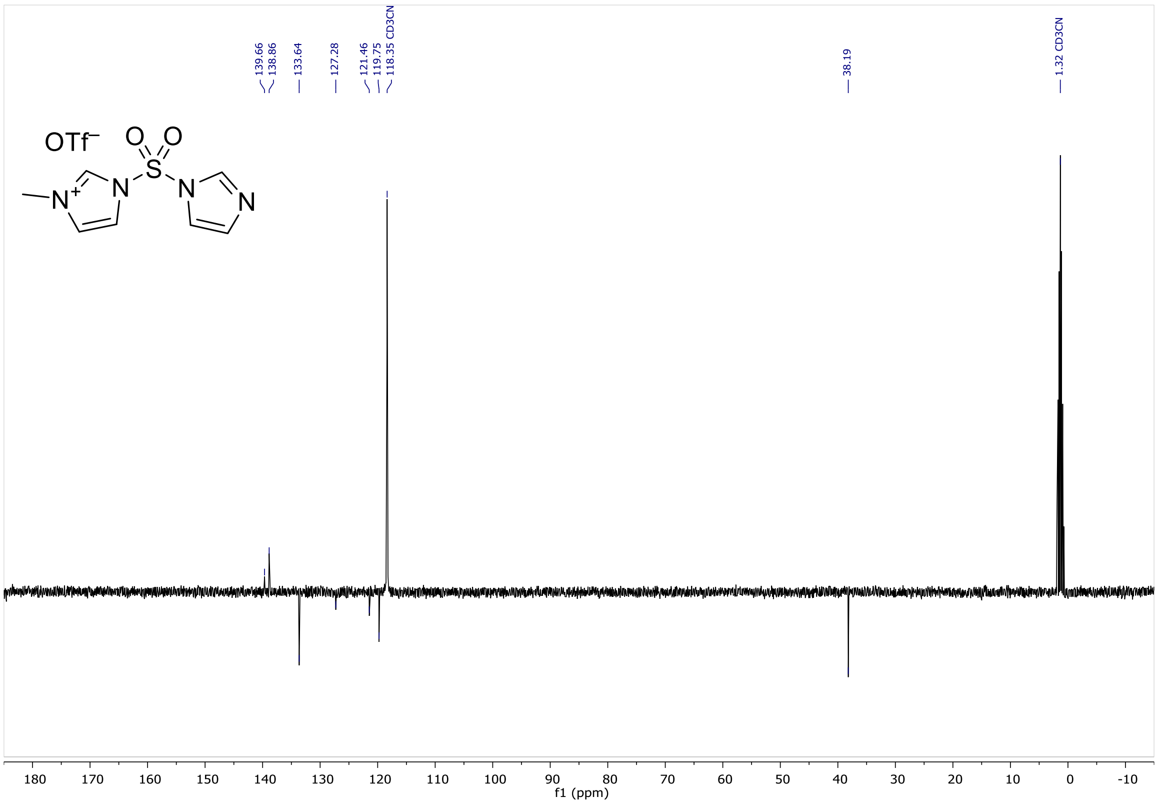

^{19}F NMR (376 MHz, CD_3CN)

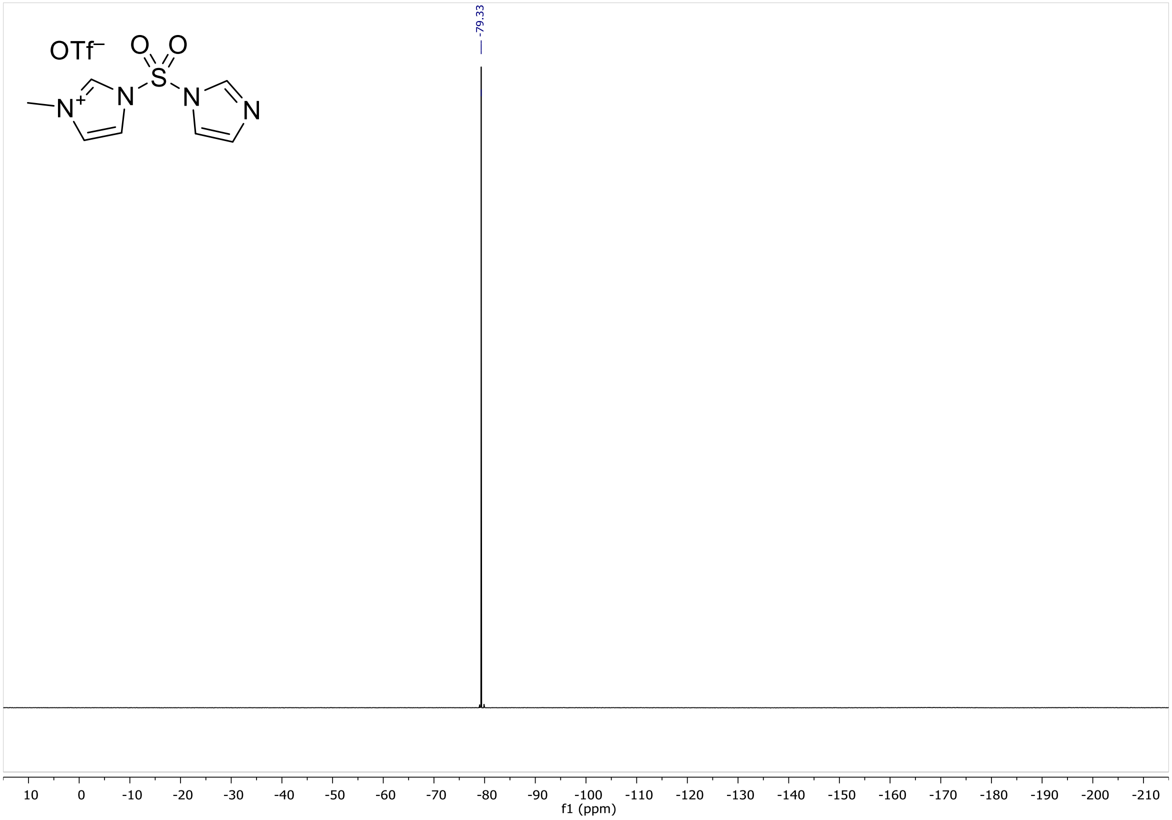

1*H*-Imidazole-1-sulfonyl fluoride (10)

^{1}H NMR (400 MHz, $CDCl_3$)

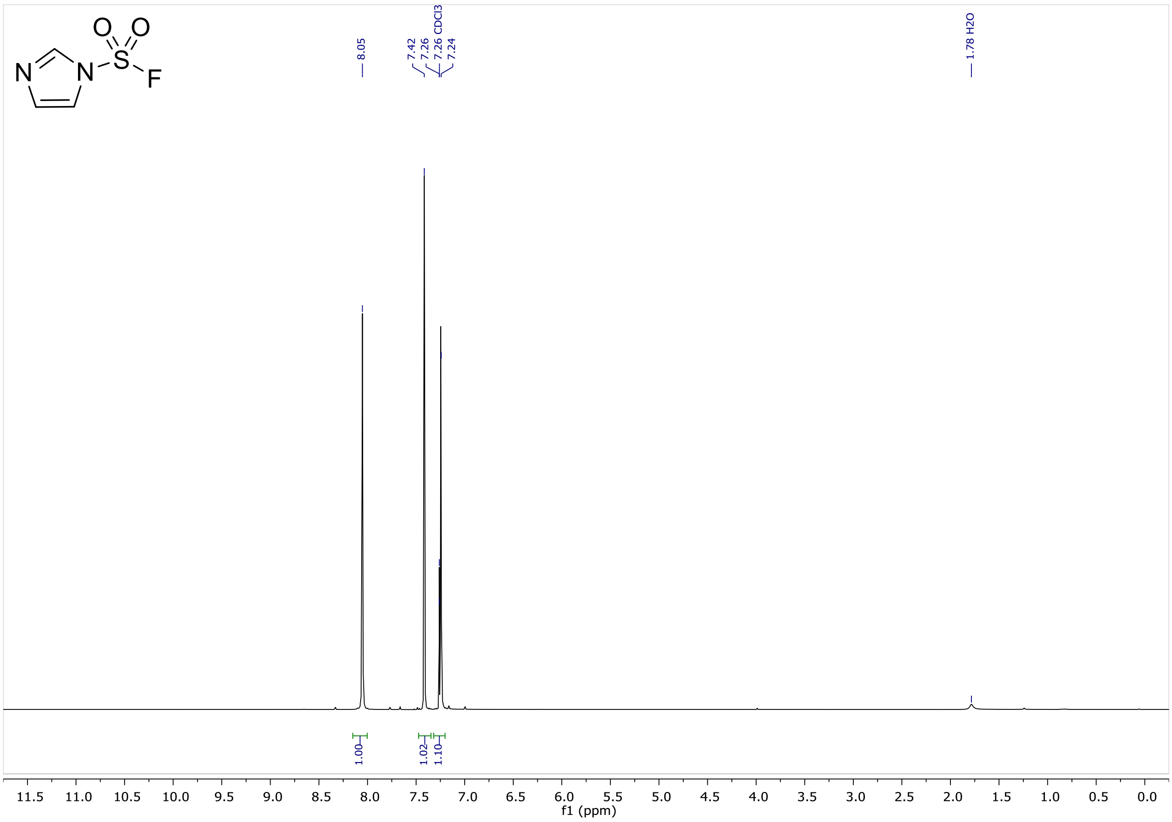

$^{13}C\{^{1}H\}$ NMR (101 MHz, $CDCl_3$)

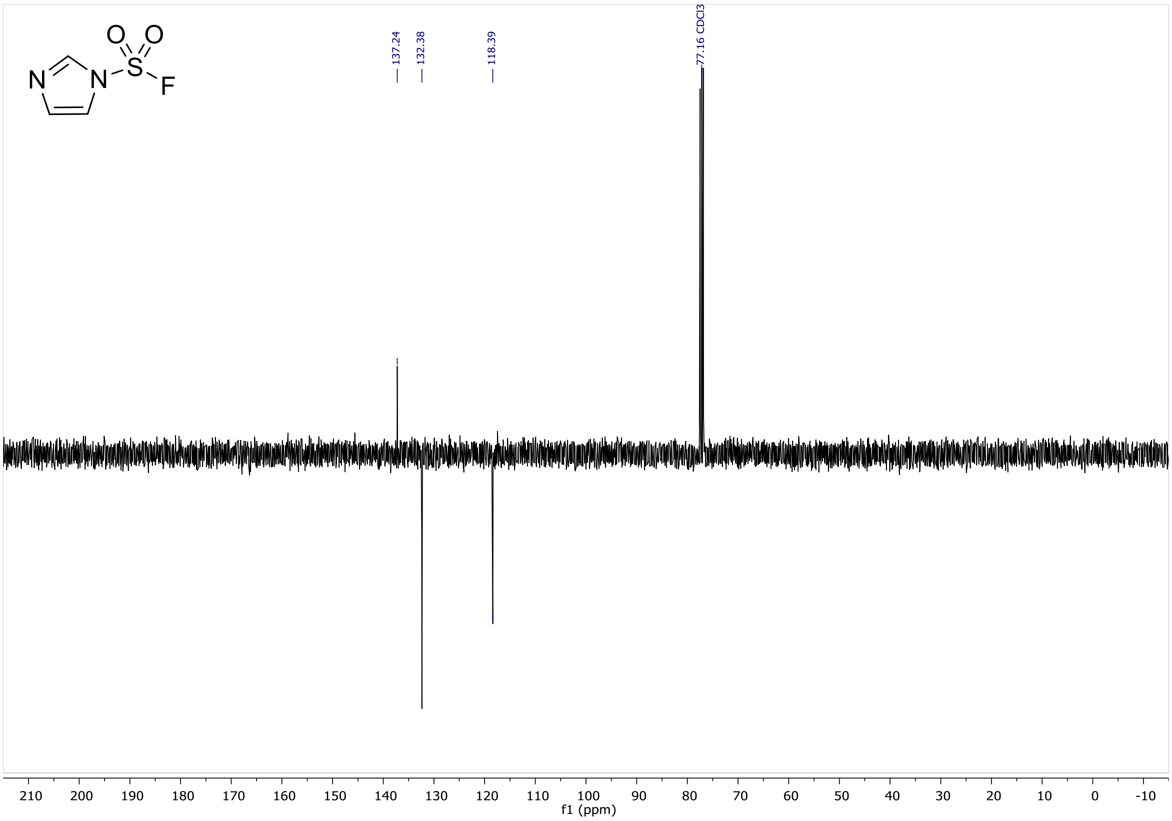

^{19}F NMR (376 MHz, $CDCl_3$)

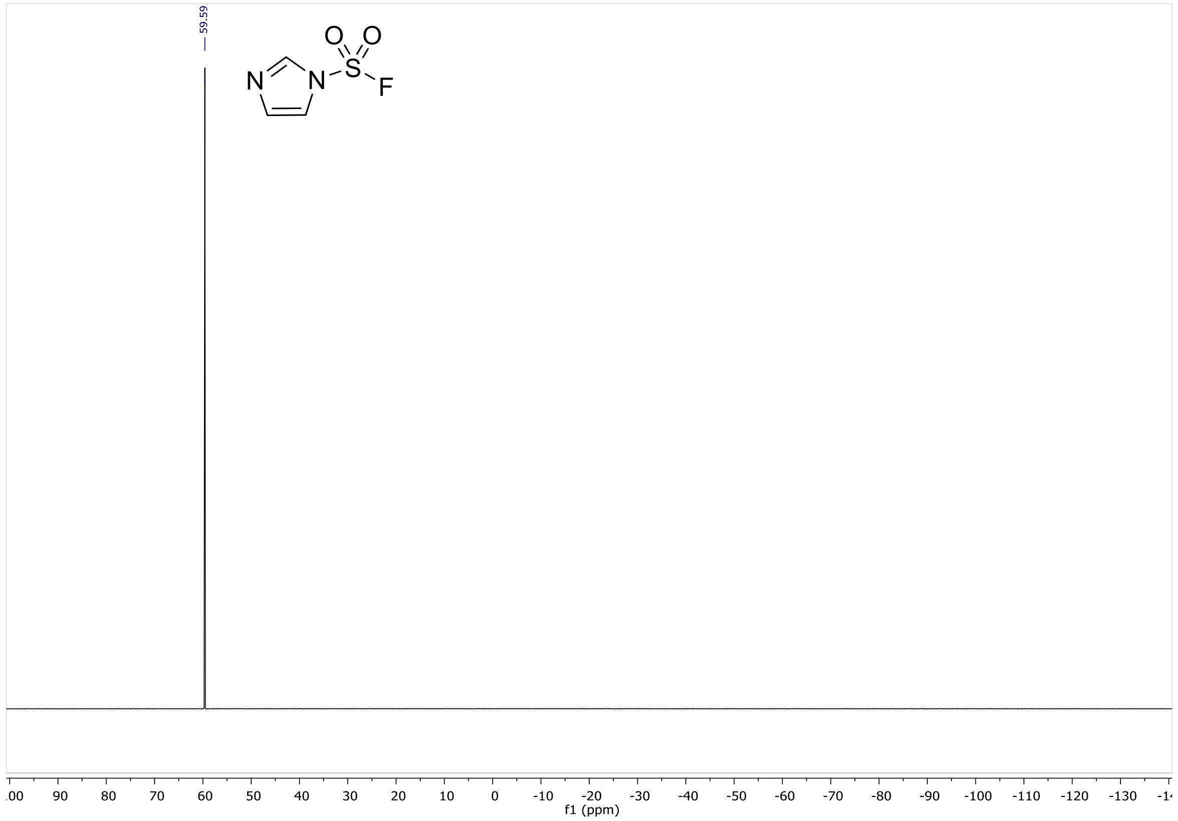

1-(Fluorosulfonyl)-3-methyl-1*H*-imidazole trifluoromethanesulfonate (desmethyl SuFEx-IT, 11)

^{1}H NMR (400 MHz, CD_3CN)

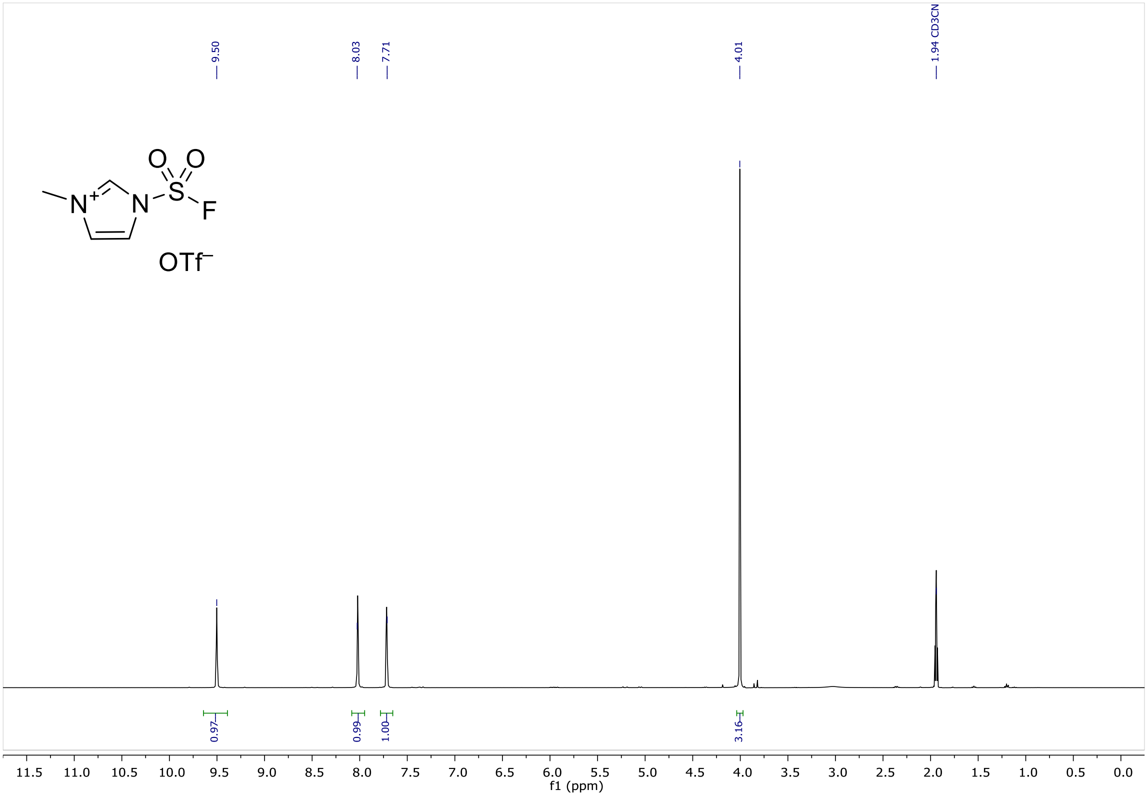

$^{13}C\{^{1}H\}$ NMR (101 MHz, CD_3CN)

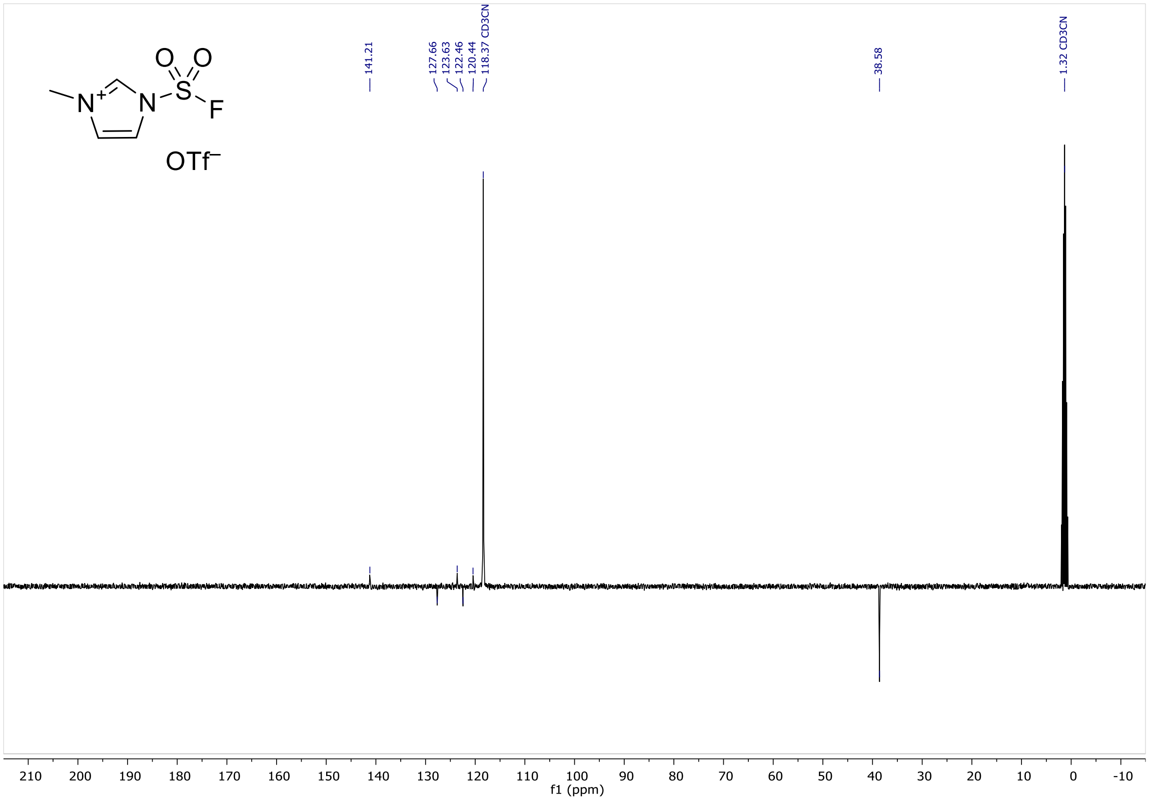

^{19}F NMR (376 MHz, CD_3CN)

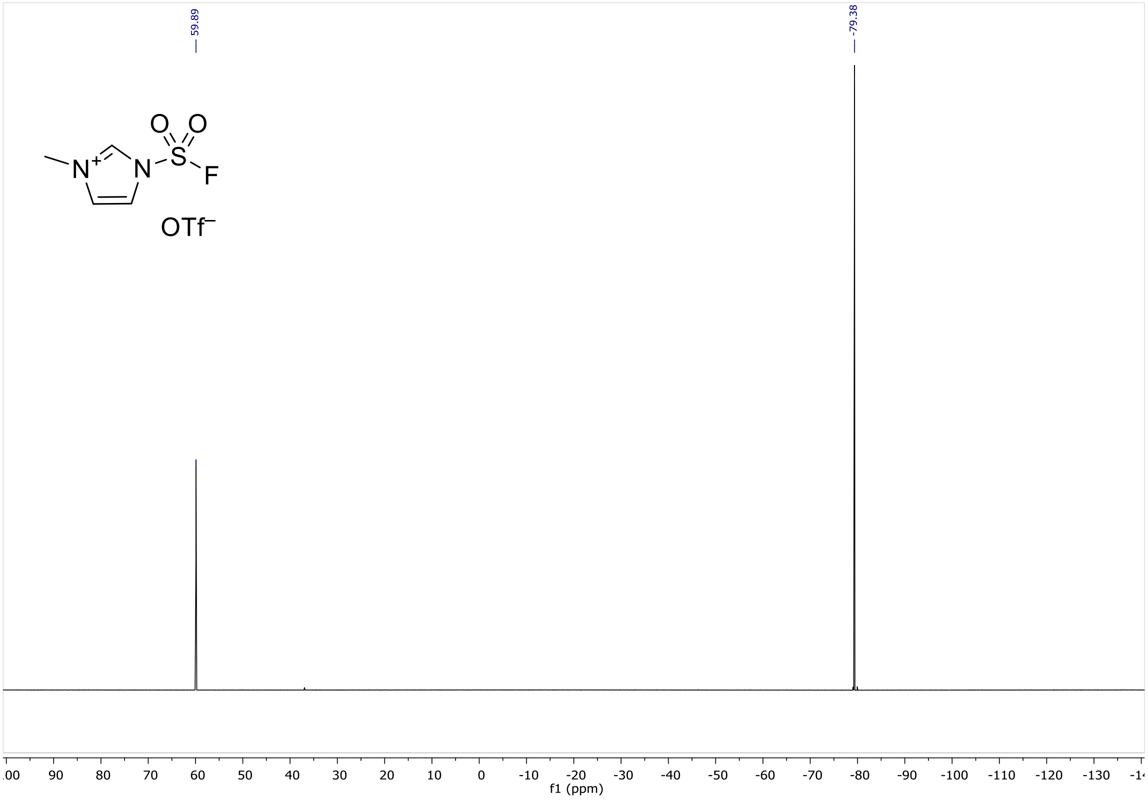

4-Bromophenyl sulfurofluoridate (12a)

^{1}H NMR (400 MHz, $CDCl_3$)

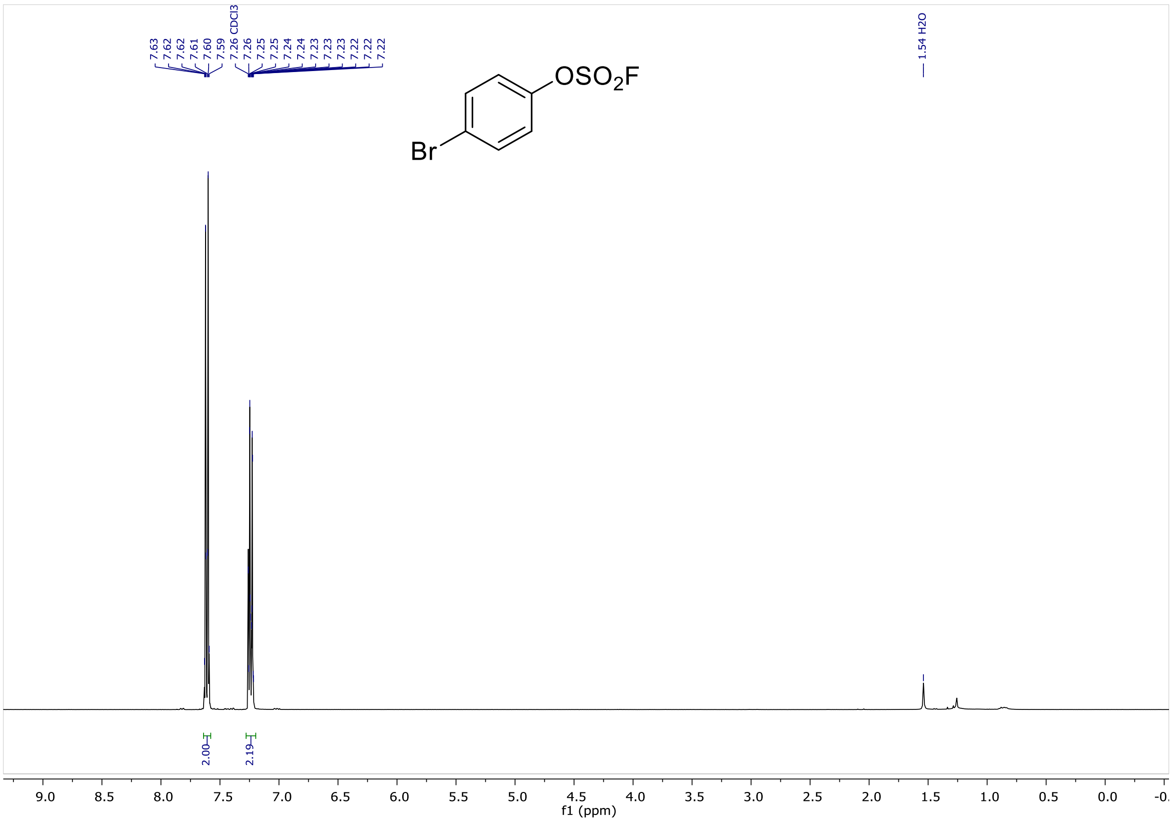

$^{13}C\{^{1}H\}$ NMR (101 MHz, $CDCl_3$)

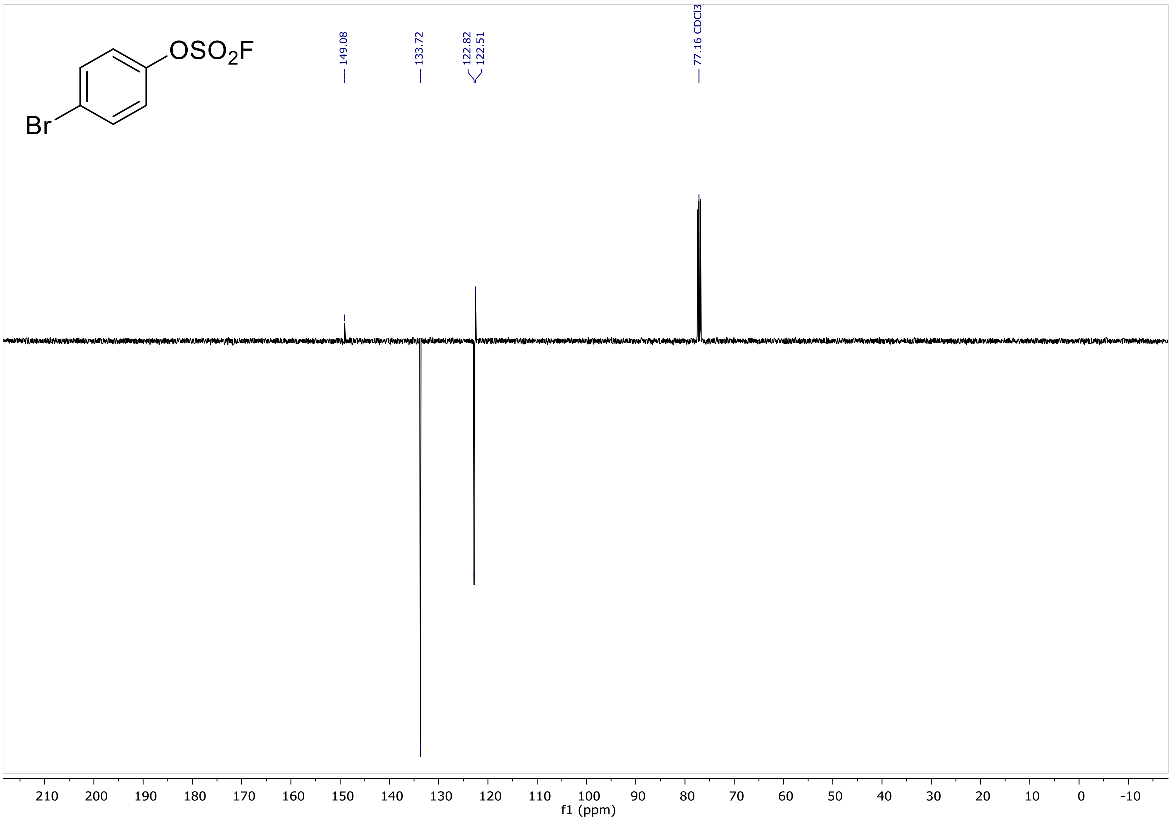

^{19}F NMR (376 MHz, $CDCl_3$)

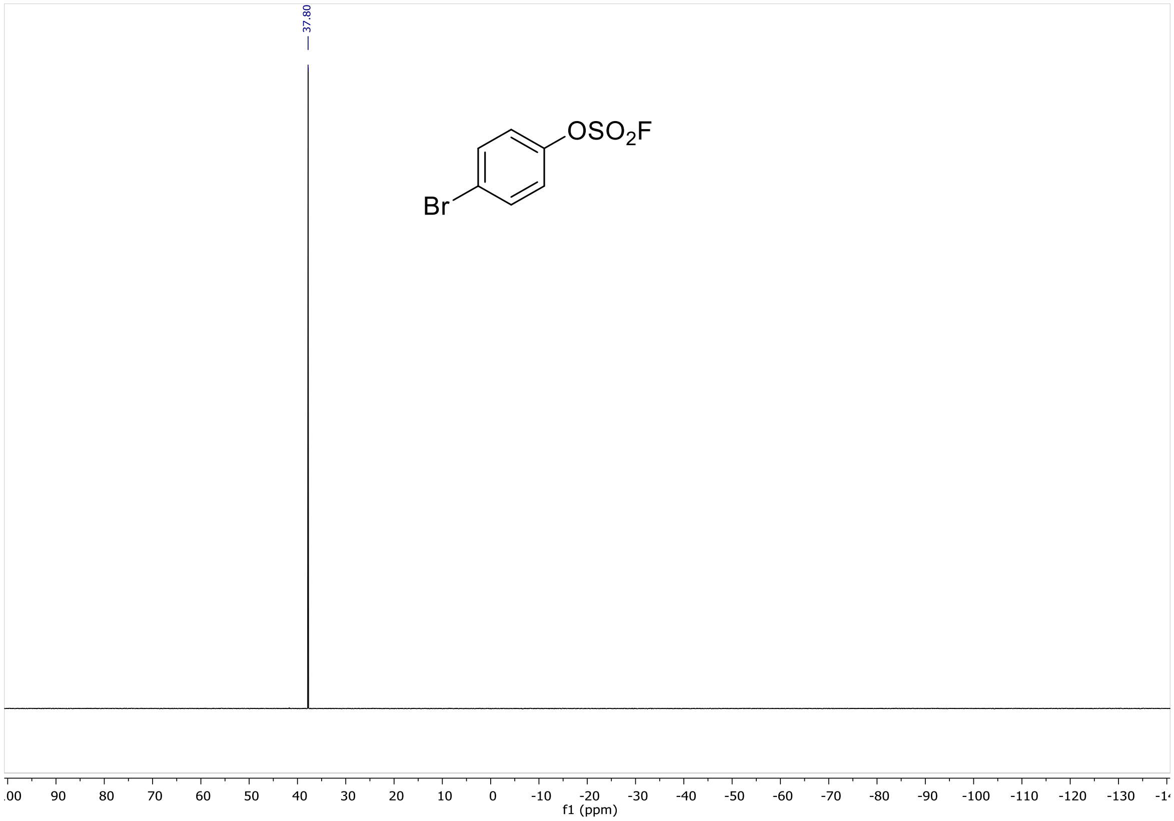

3-Methoxyphenyl sulfurofluoridate (12b)

^{1}H NMR (400 MHz, $CDCl_3$)

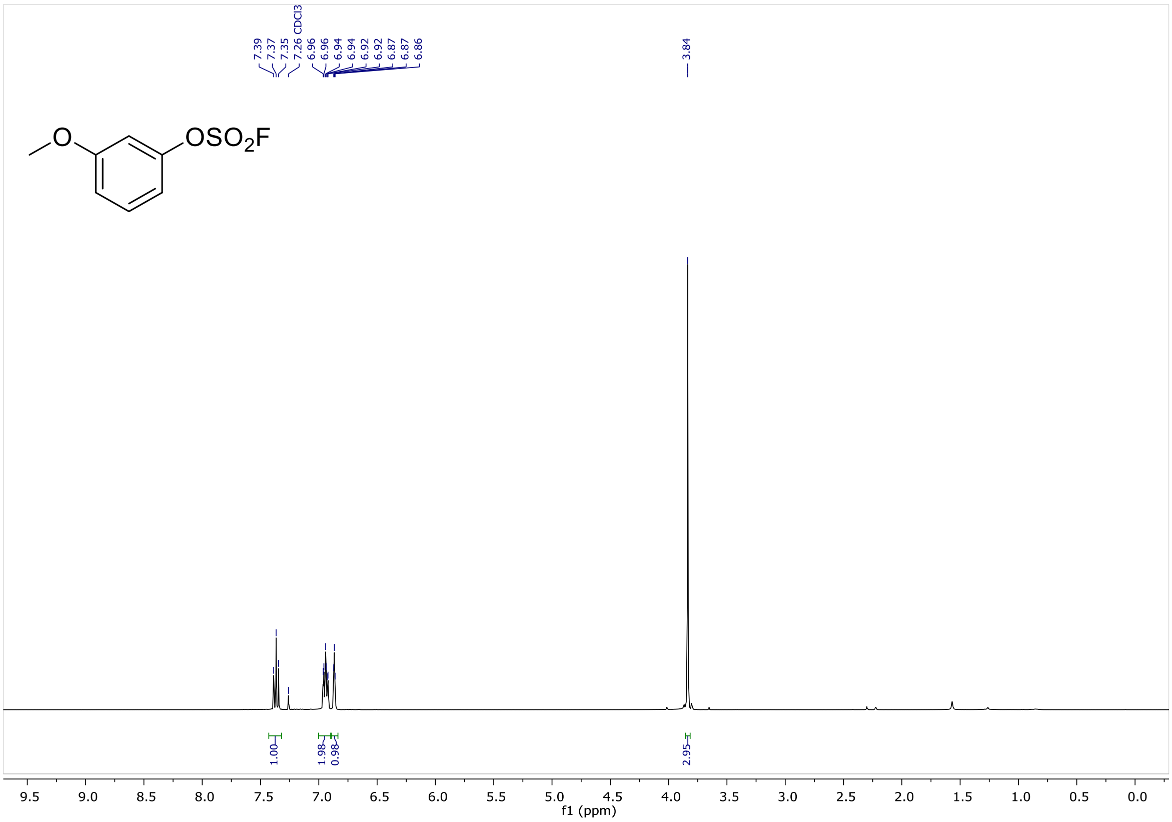

^{13}C{^{1}H} NMR (101 MHz, $CDCl_3$)

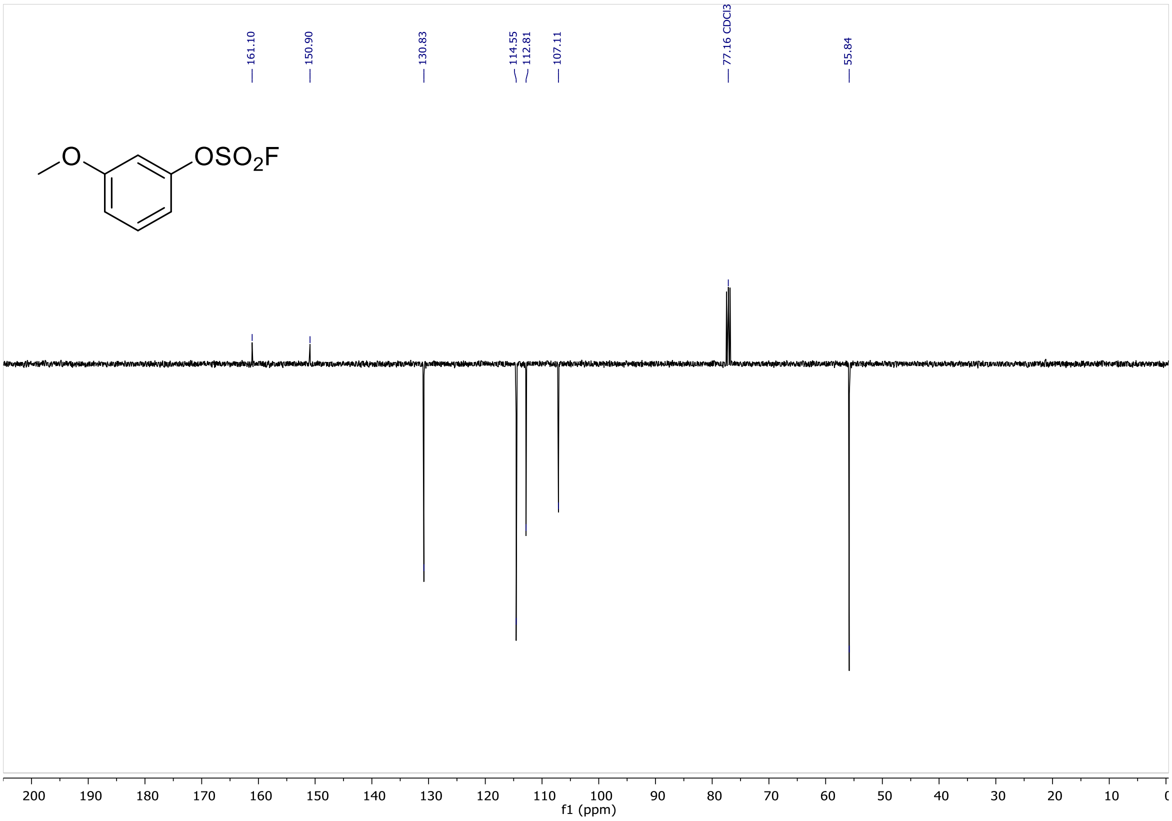

^{19}F NMR (376 MHz, $CDCl_3$)

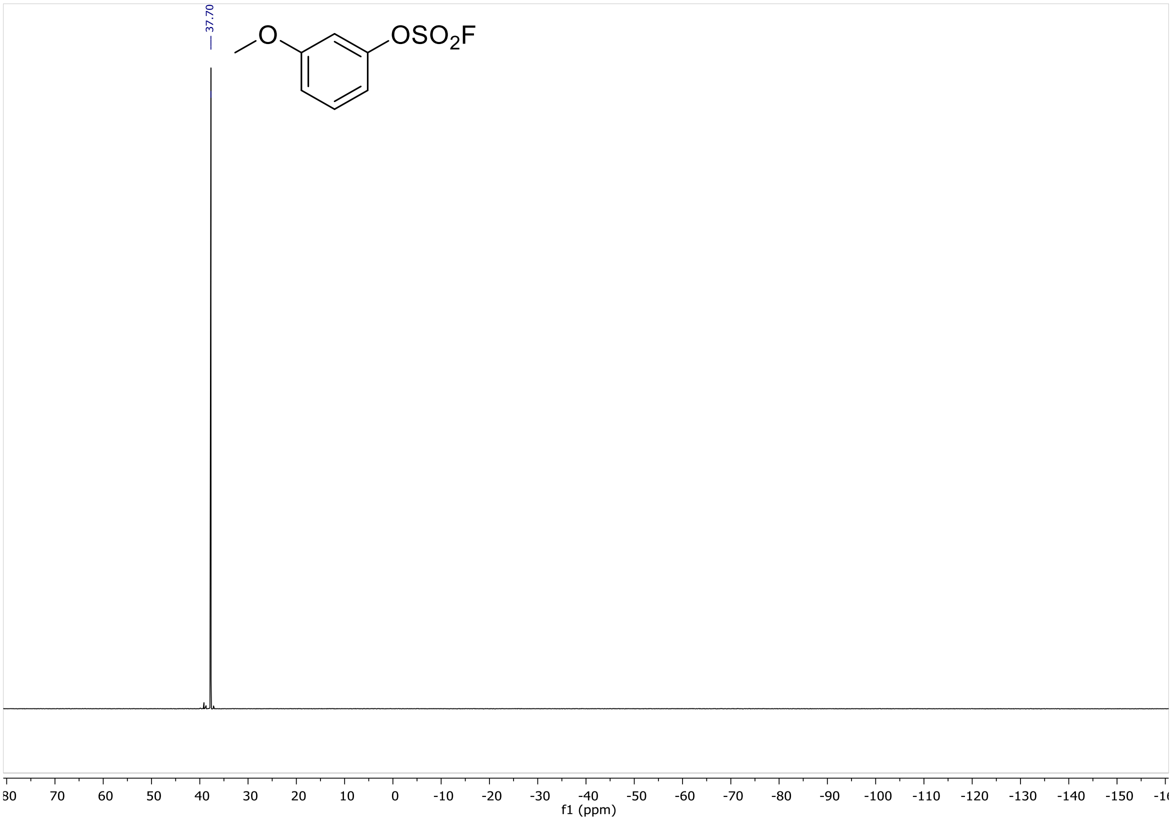

4-Formylphenyl sulfurofluoridate (12c)

^{1}H NMR (400 MHz, $CDCl_3$)

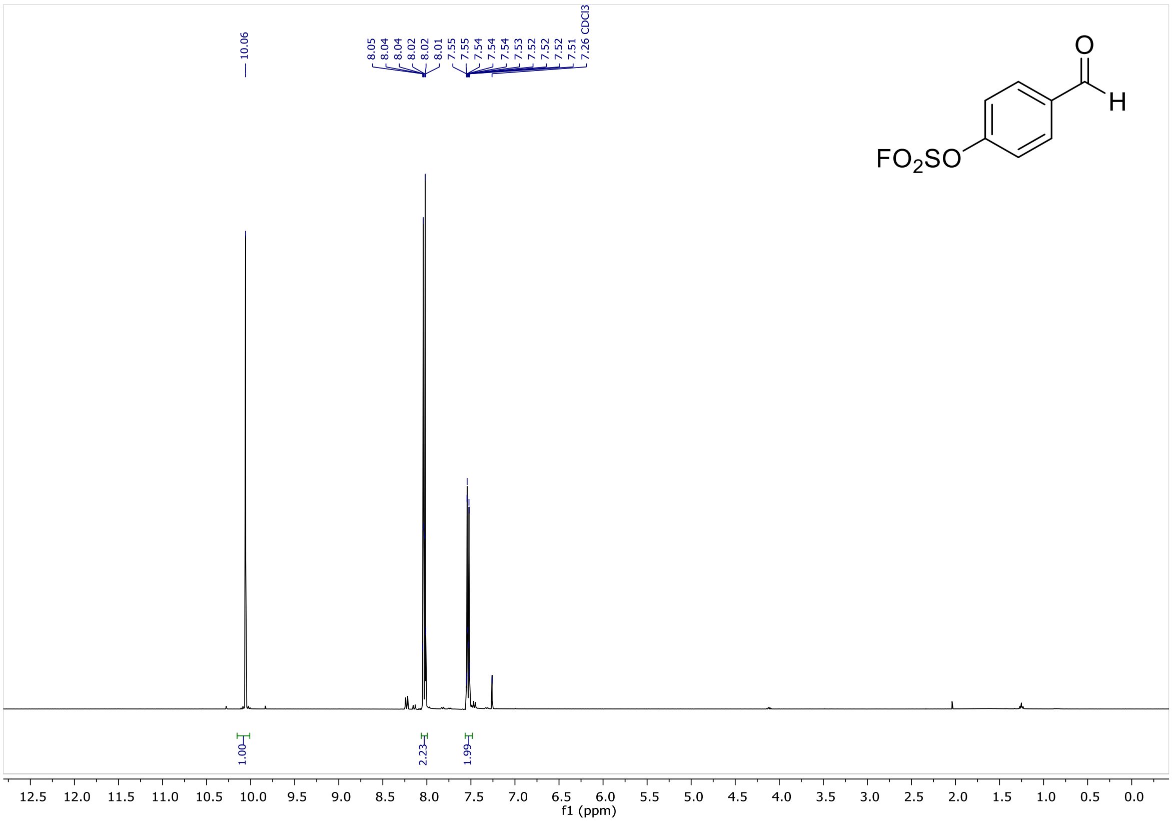

$^{13}C\{^{1}H\}$ NMR (101 MHz, $CDCl_3$)

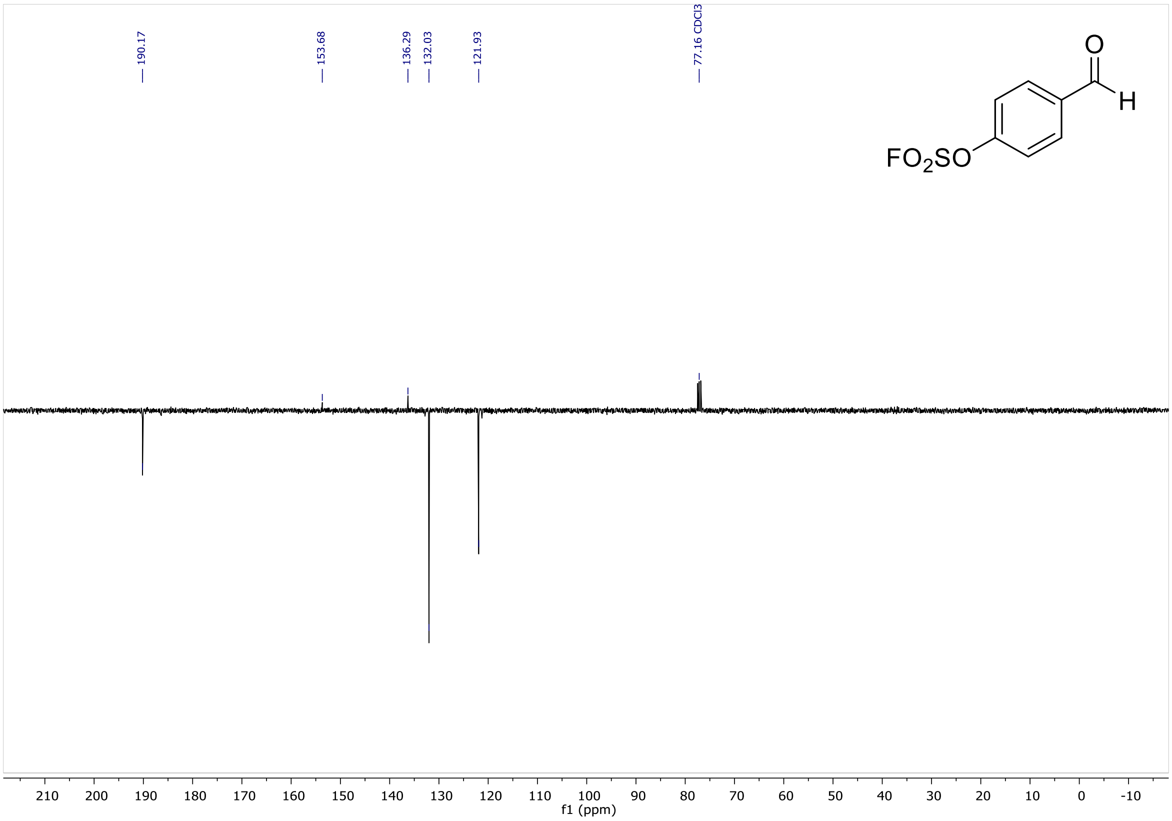

^{19}F NMR (376 MHz, $CDCl_3$)

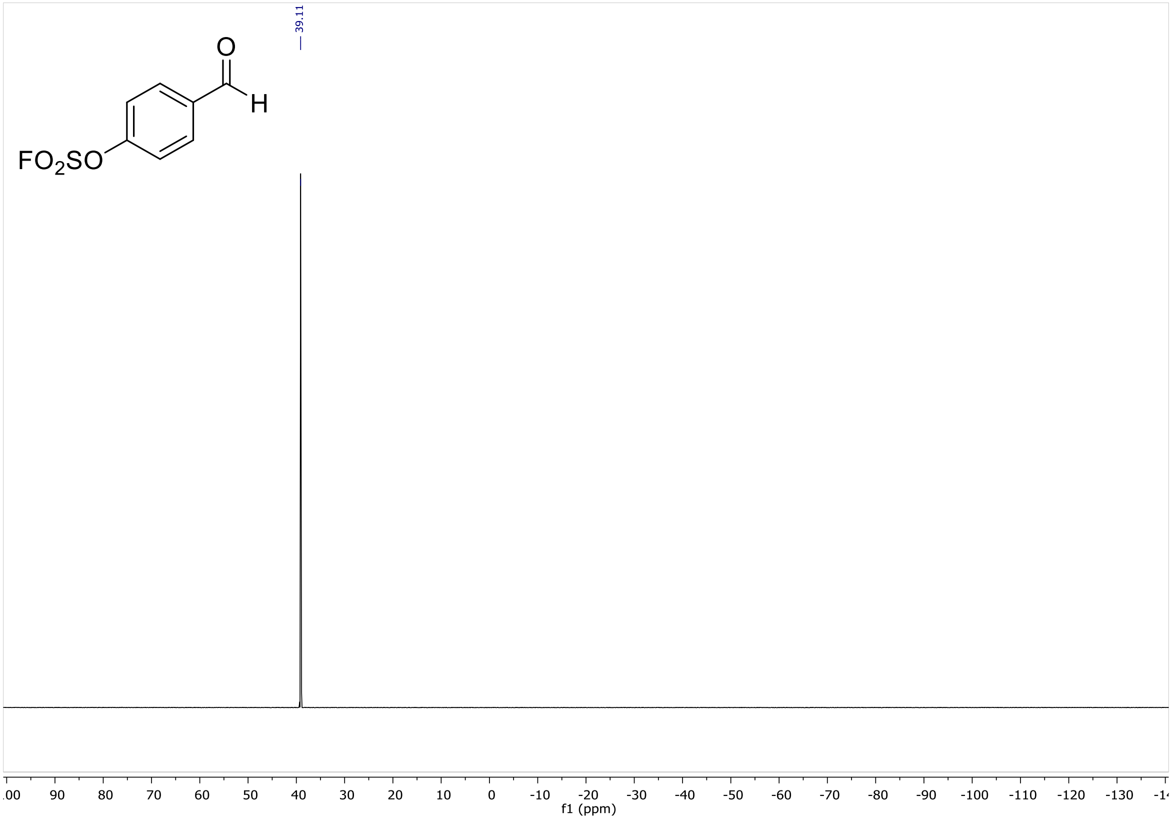

Quinolin-8-yl sulfurofluoridate (12d)

^1H NMR (400 MHz, $CDCl_3$)

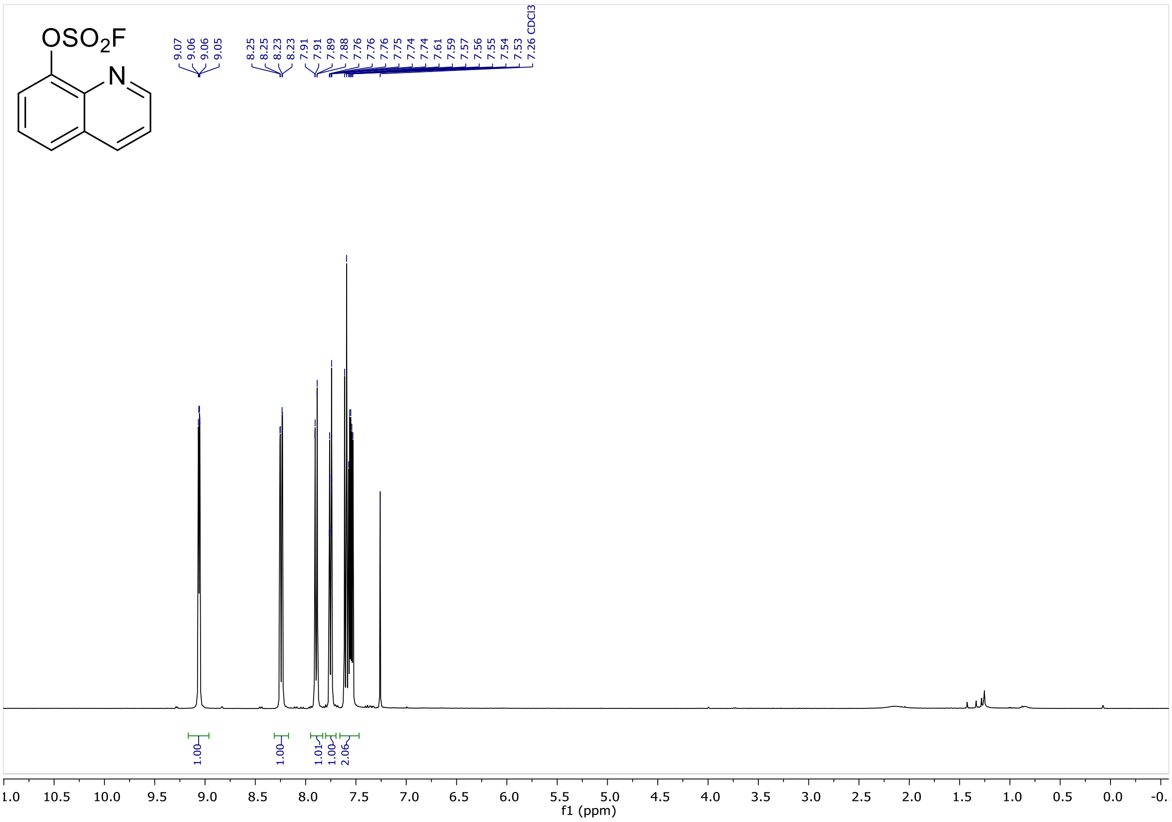

$^{13}C\{^1H\}$ NMR (101 MHz, $CDCl_3$)

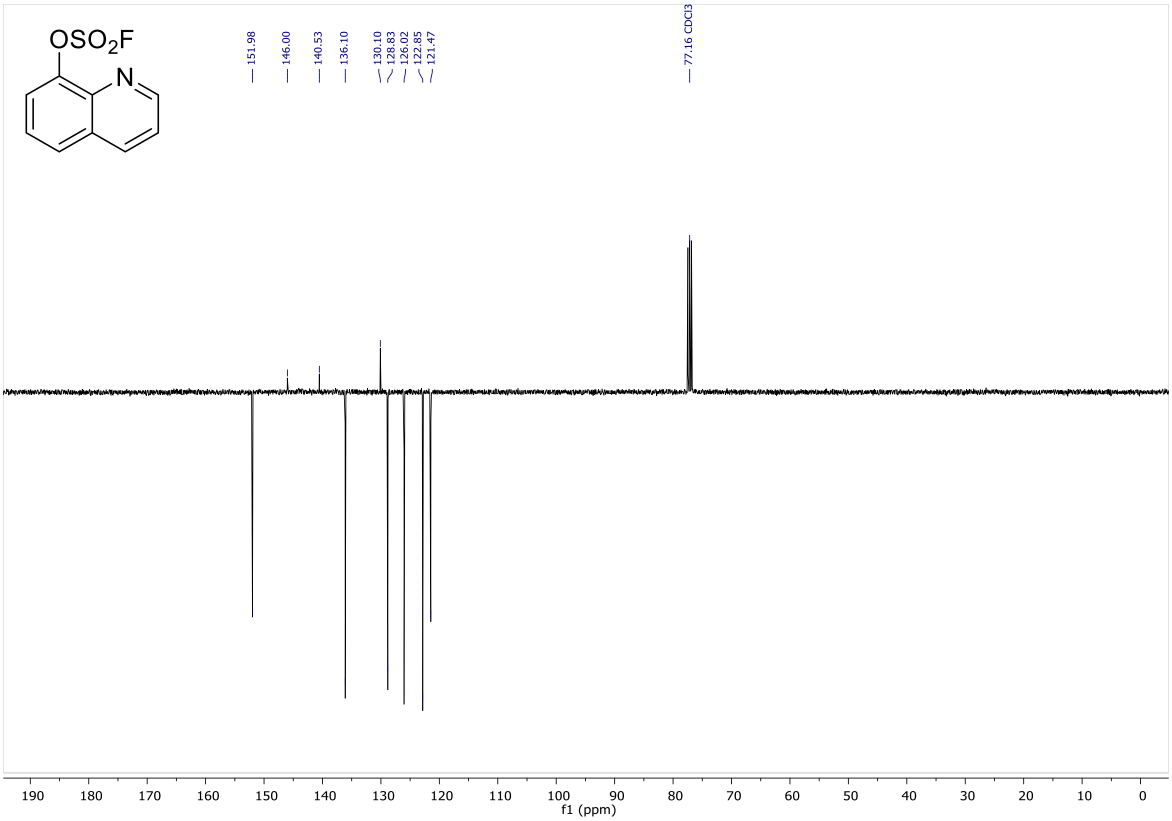

^{19}F NMR (376 MHz, $CDCl_3$)

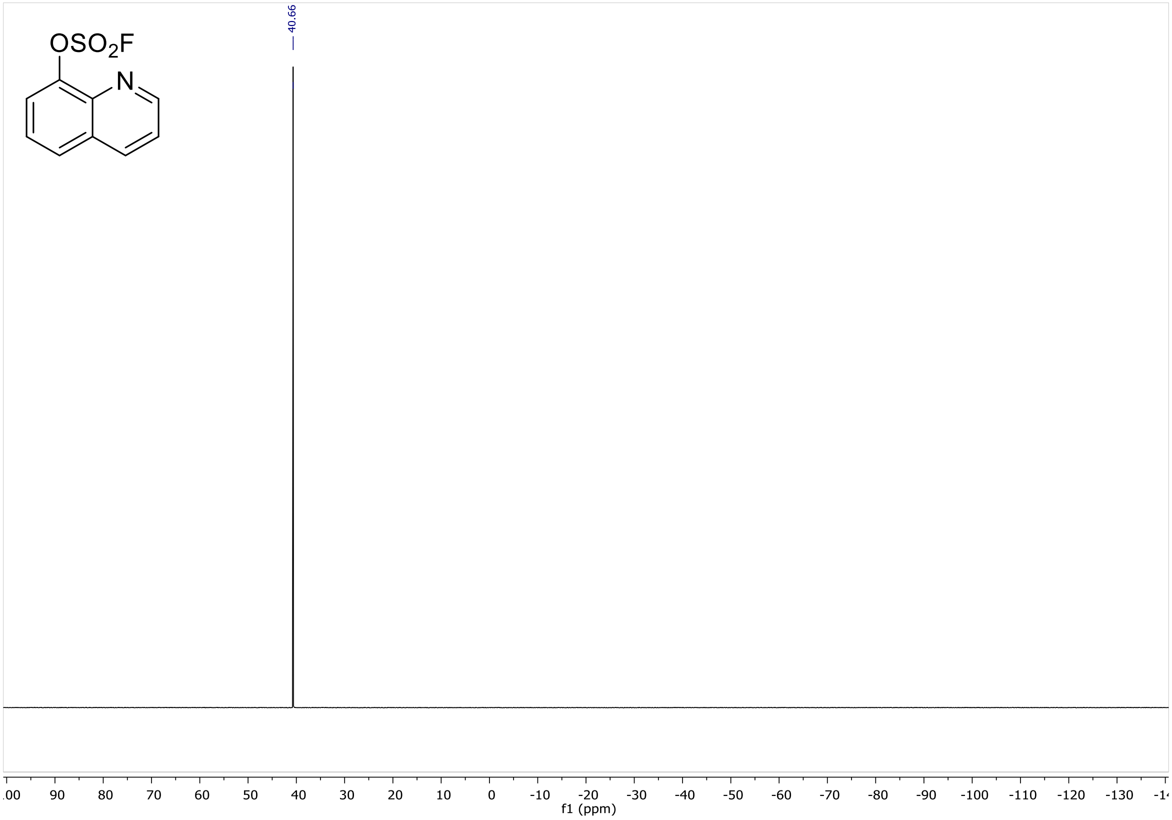

4-[(4-Acetamidophenyl)ethynyl]phenyl sulfurofluoridate (12e)

^{1}H NMR (400 MHz, THF-d8)

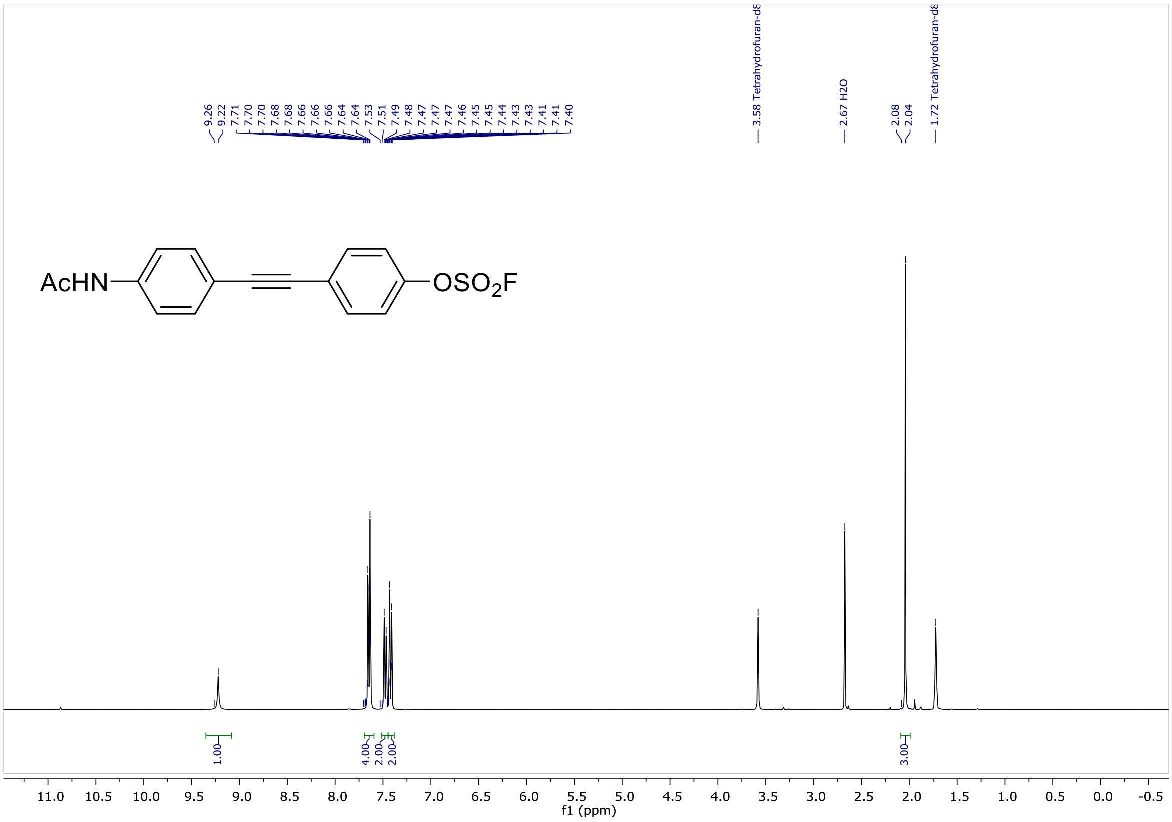

$^{13}C\{^{1}H\}$ NMR (101 MHz, THF-d8)

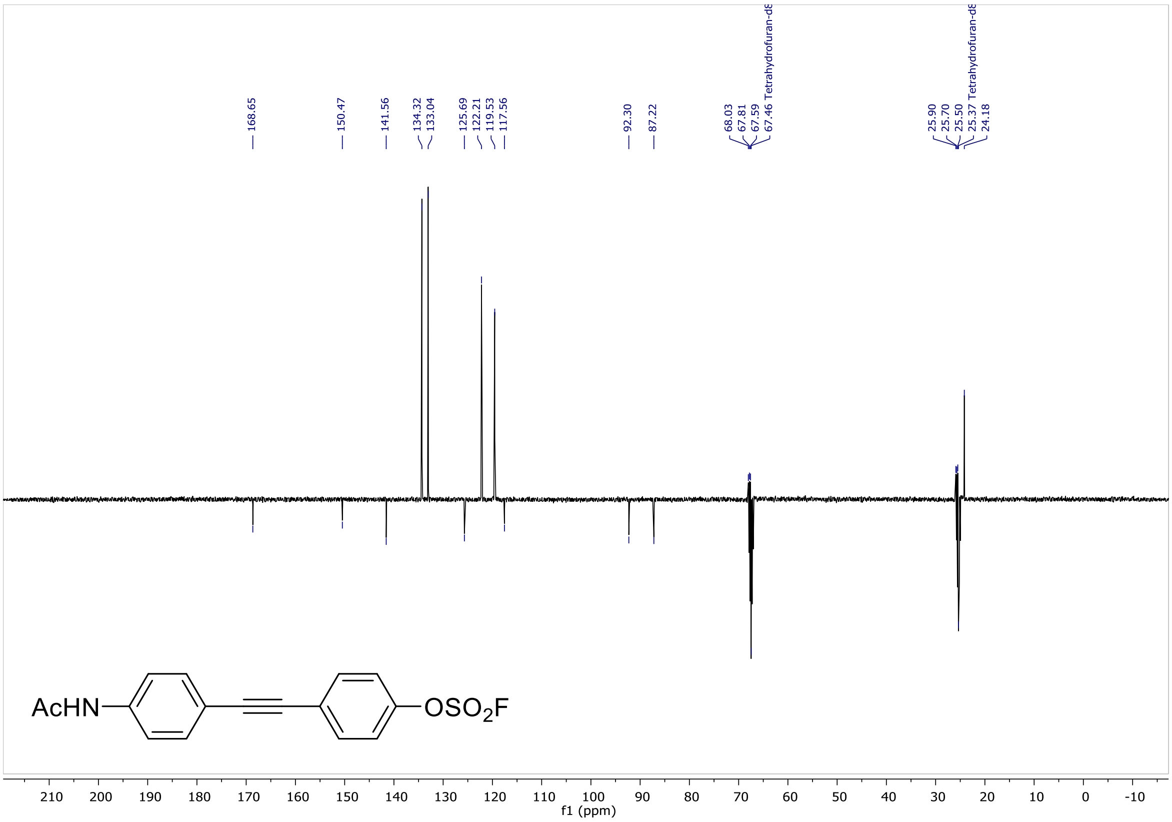

^{19}F NMR (376 MHz, THF-d8)

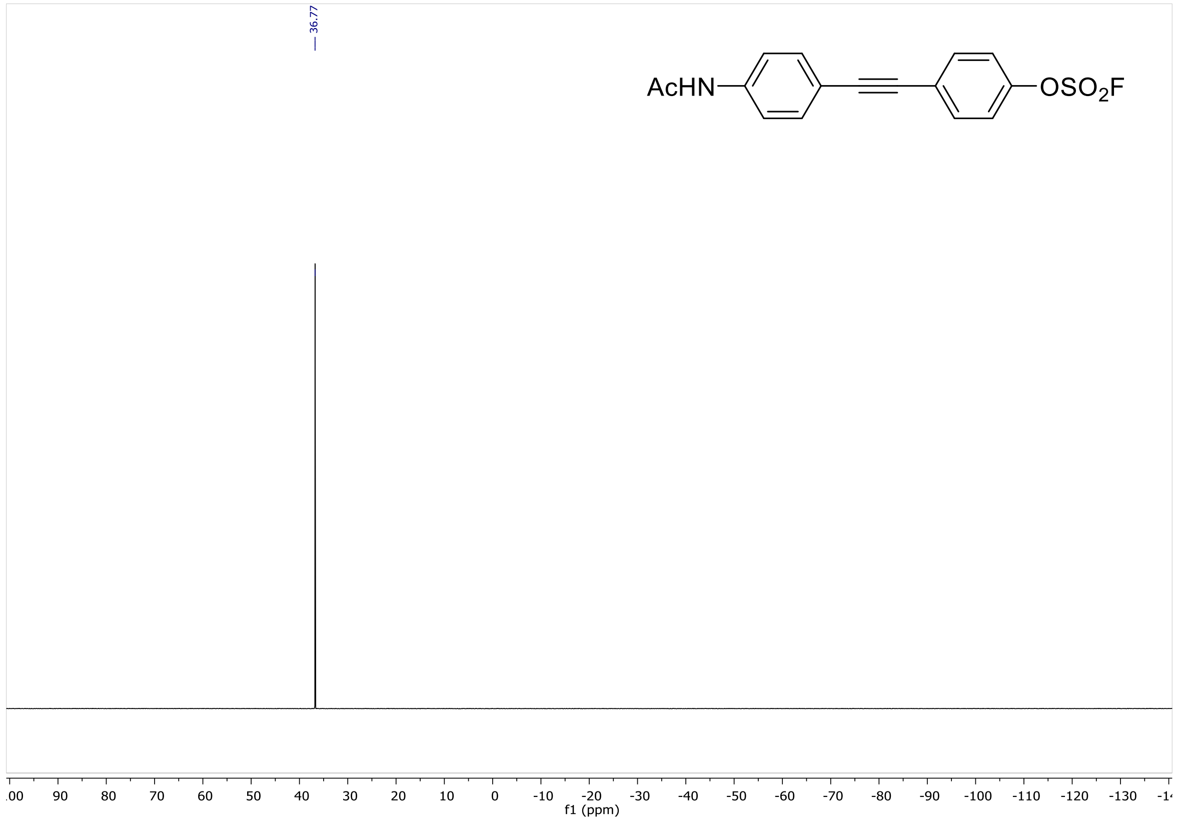

4-(4,4,5,5-Tetramethyl-1,3,2-dioxaborolan-2-yl)phenyl sulfurofluoridate (12f)

^{1}H NMR (400 MHz, $CDCl_3$)

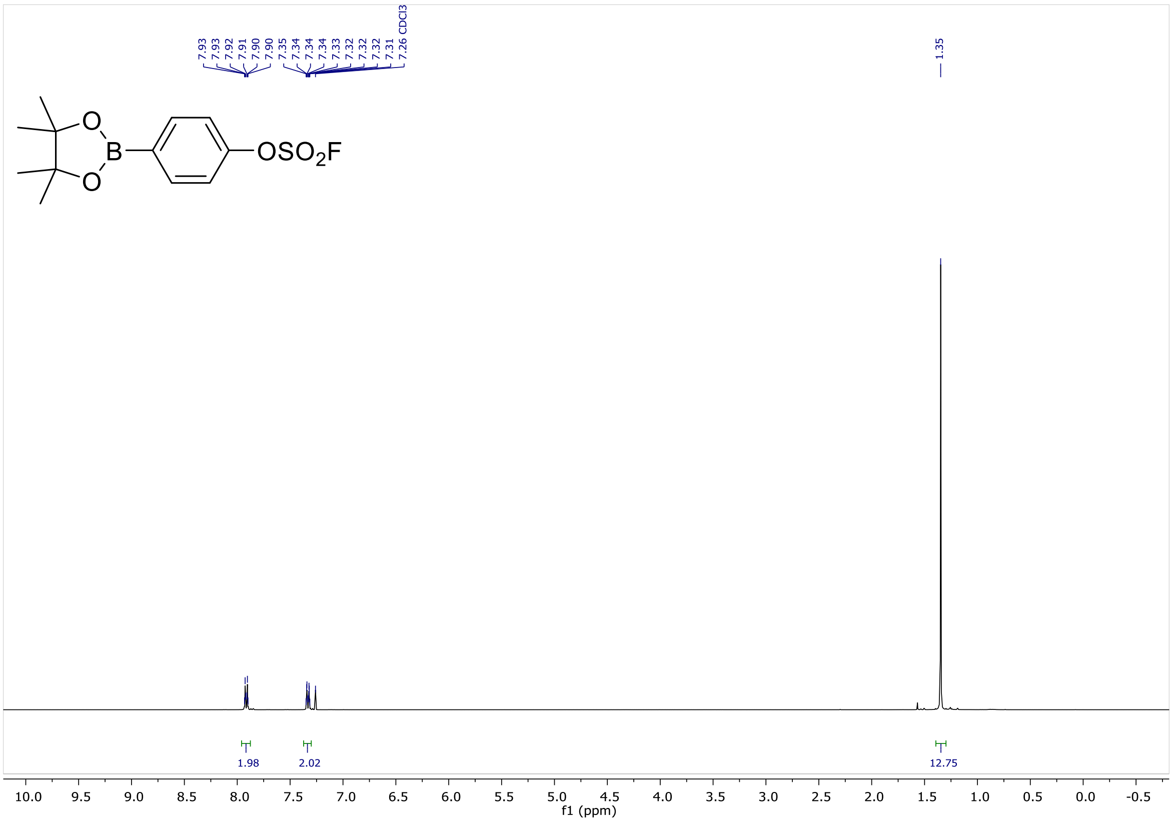

^{13}C{^{1}H} NMR (101 MHz, $CDCl_3$)

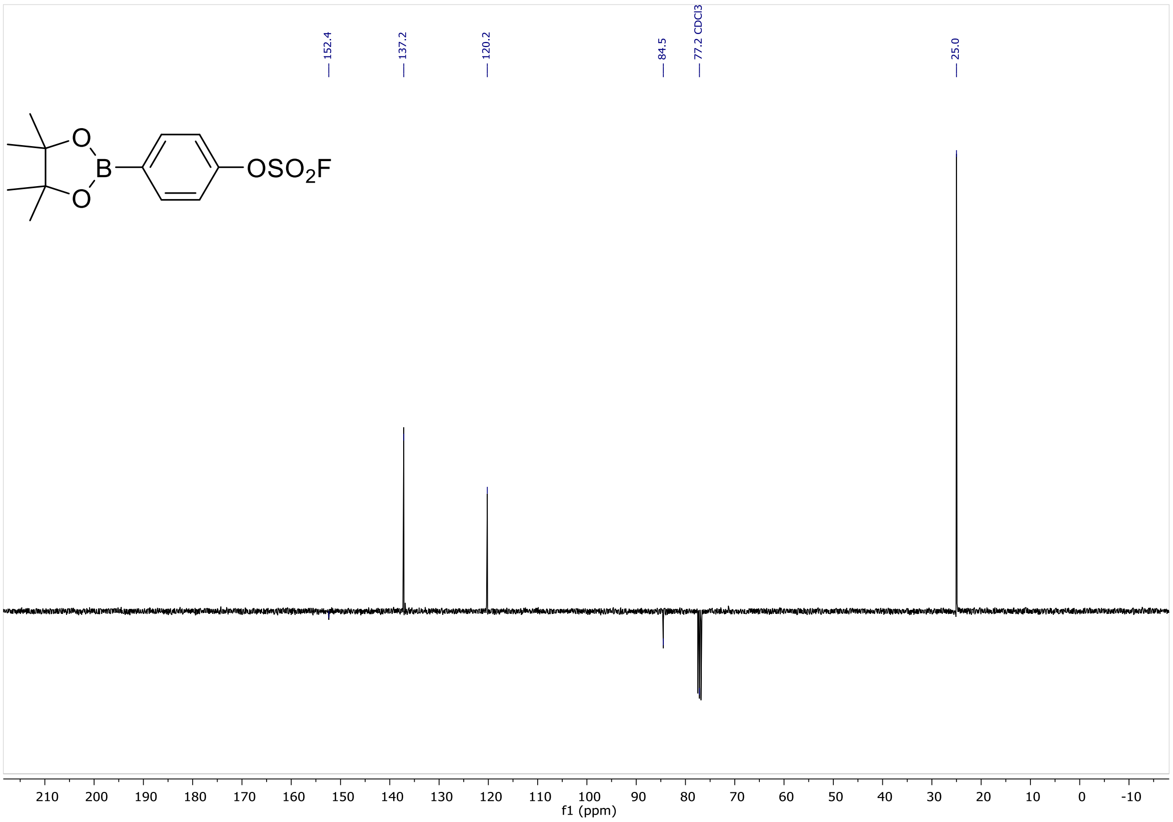

^{19}F NMR (376 MHz, $CDCl_3$)

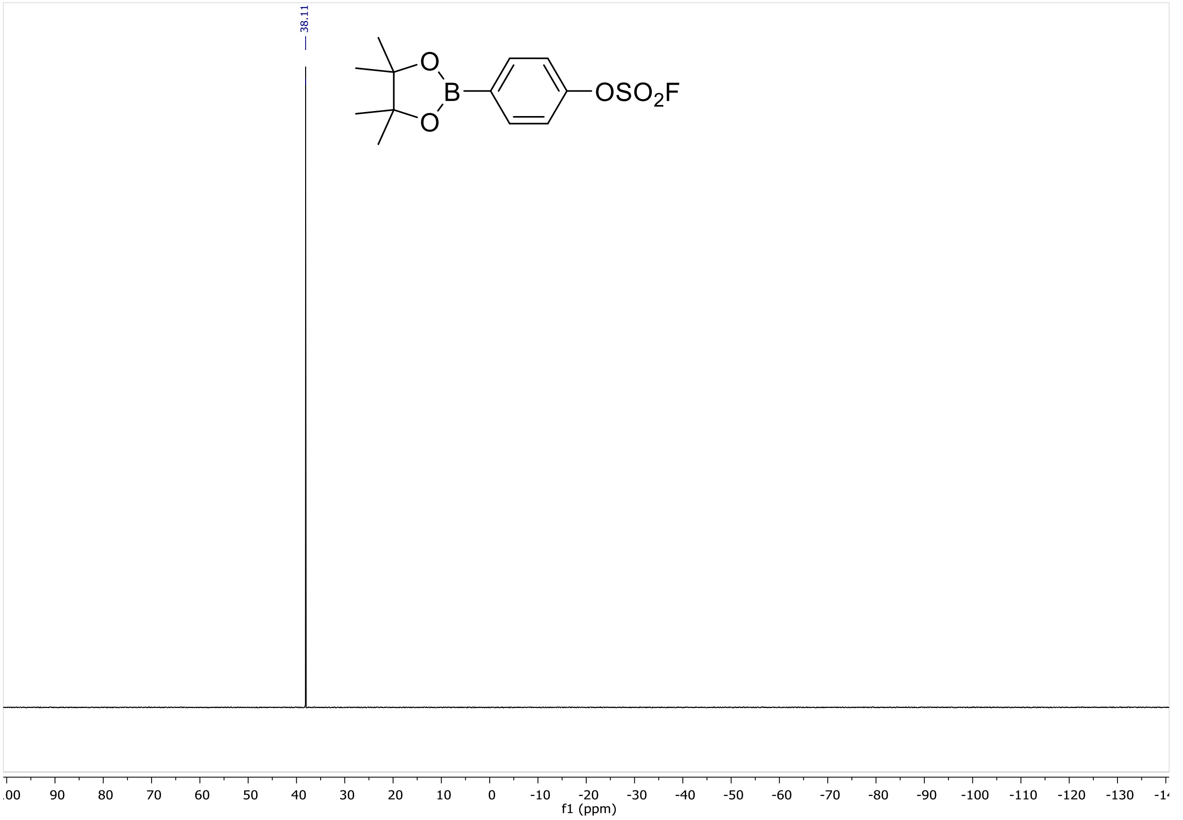

2-Mercaptobenzo[d]thiazol-6-yl sulfurofluoridate (12g)

^{1}H NMR [400 MHz, $(CD_3)_2SO$]

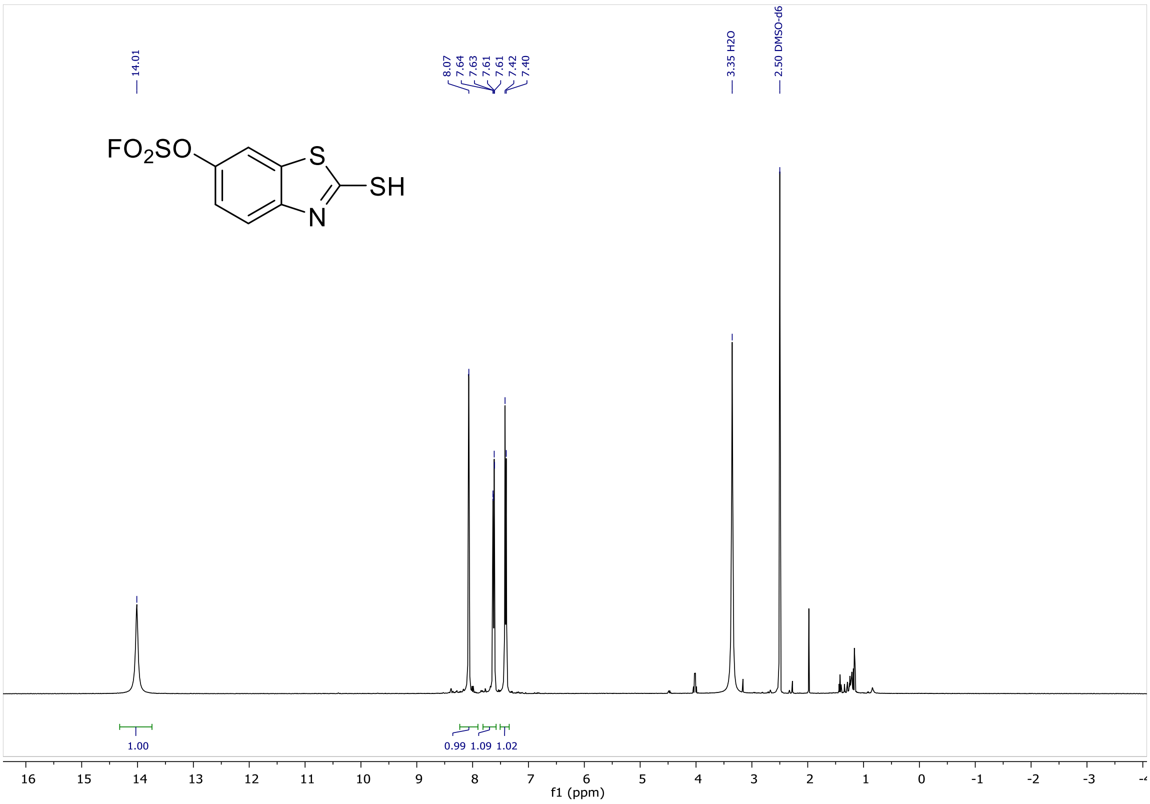

$^{13}C\{^{1}H\}$ NMR [101 MHz, $(CD_3)_2SO$]

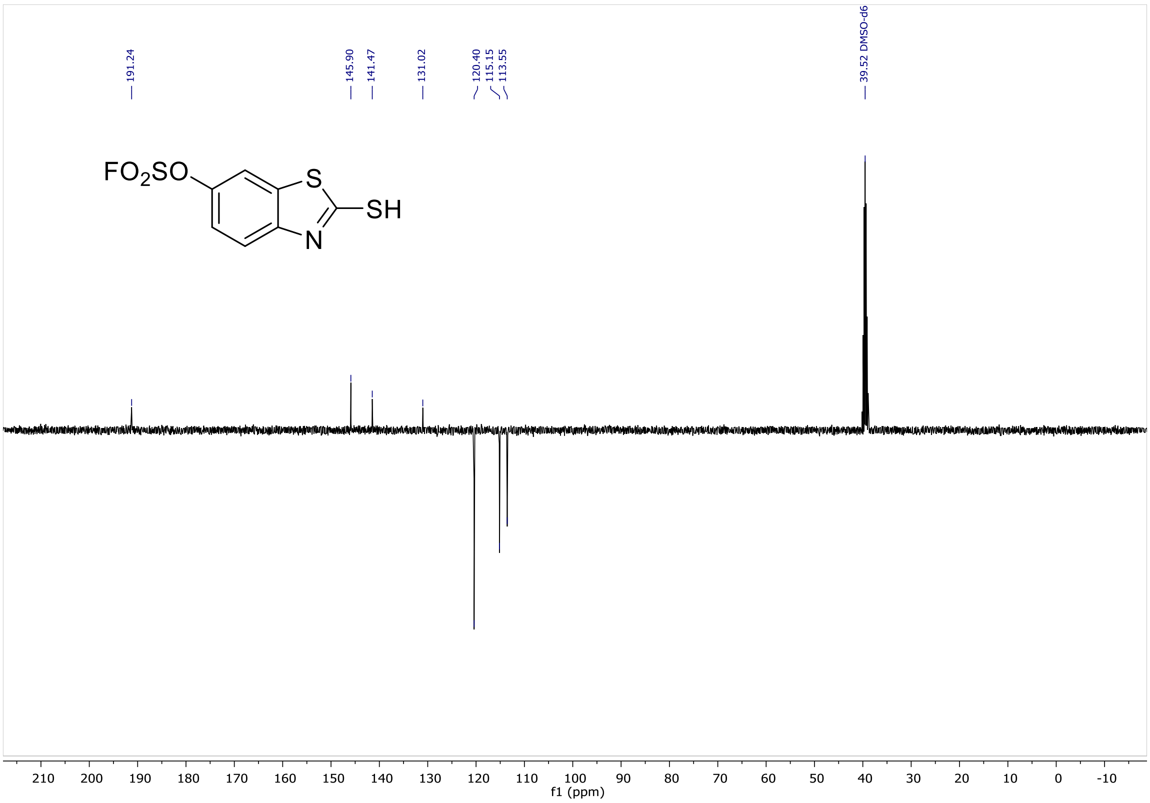

^{19}F NMR [376 MHz, $(CD_3)_2SO$]

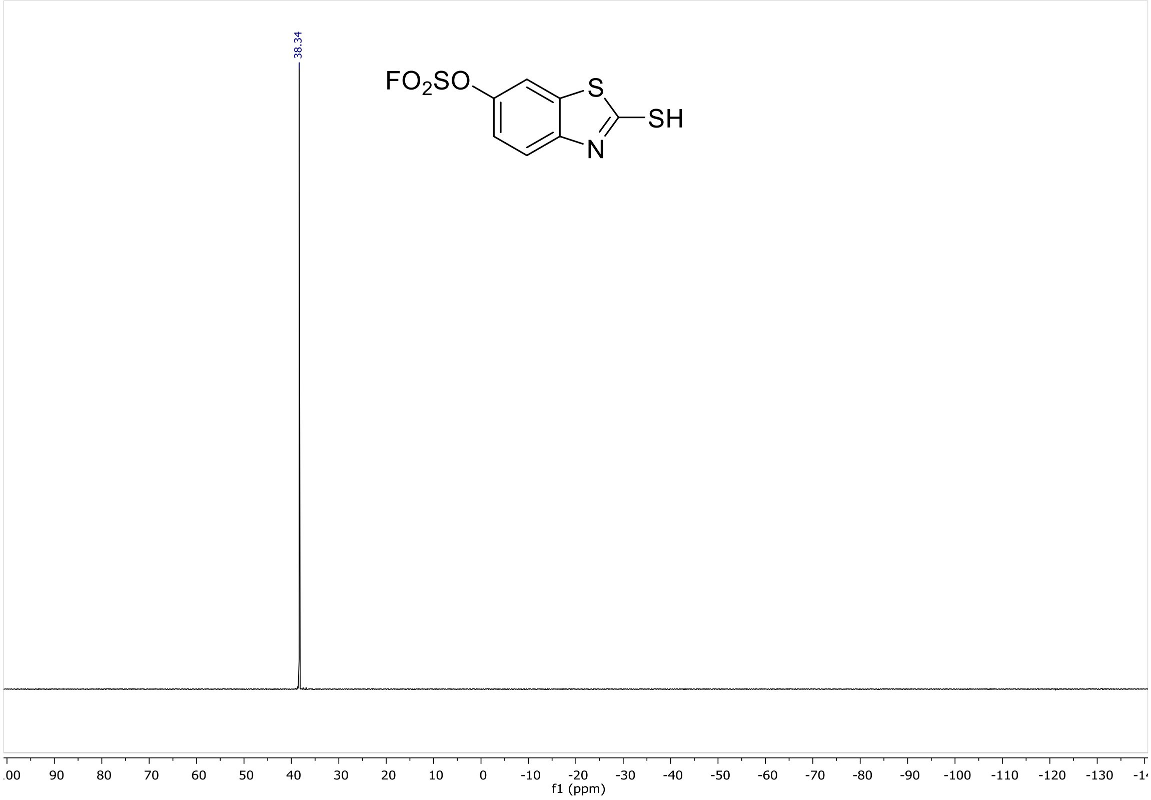

±-α-Tocopherol-derived sulfurofluoridate (12h)

^1H NMR (400 MHz, $CDCl_3$)

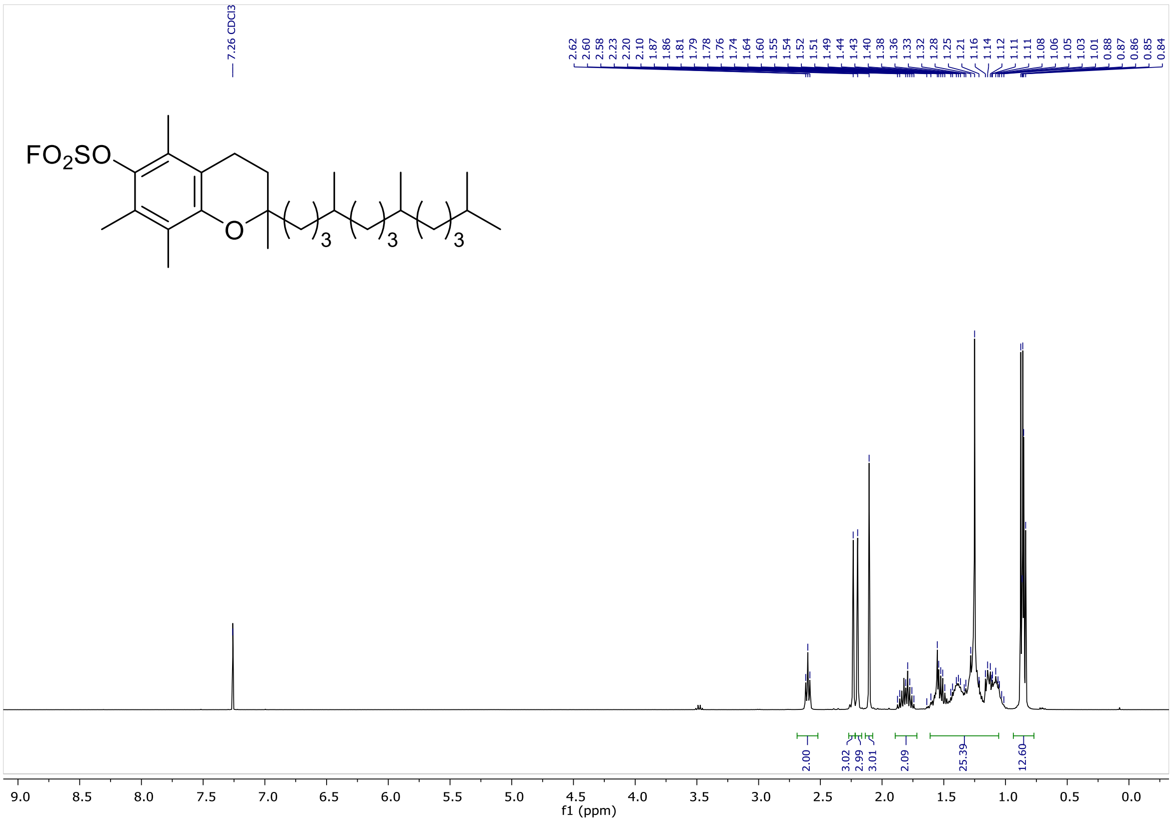

$^{13}C\{^1H\}$ NMR (101 MHz, $CDCl_3$)

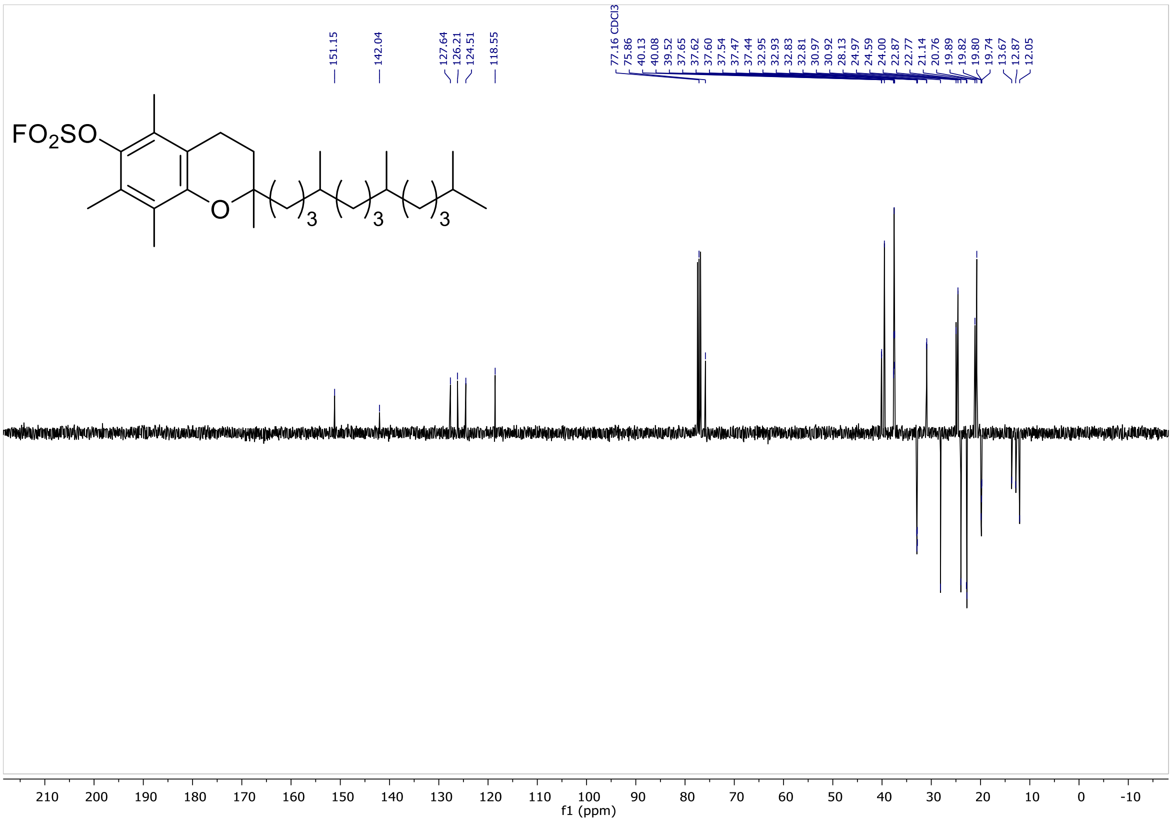

^{19}F NMR (376 MHz, $CDCl_3$)

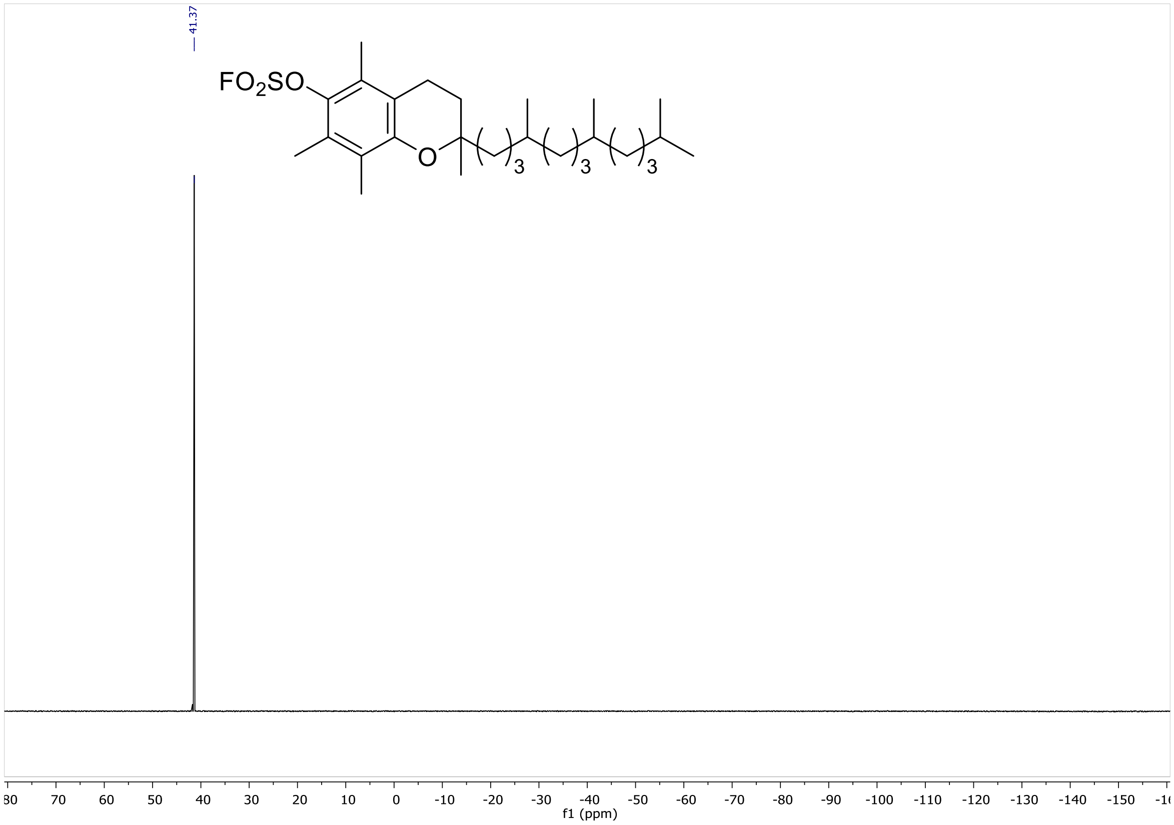

4-{[(2*S*,3*R*,4*S*,5*S*,6*R*)-3,4,5-Trihydroxy-6-(hydroxymethyl)tetrahydro-2*H*-pyran-2-yl]oxy}phenyl sulfurofluoridate (12i)

^{1}H NMR (400 MHz, CD_3OD)

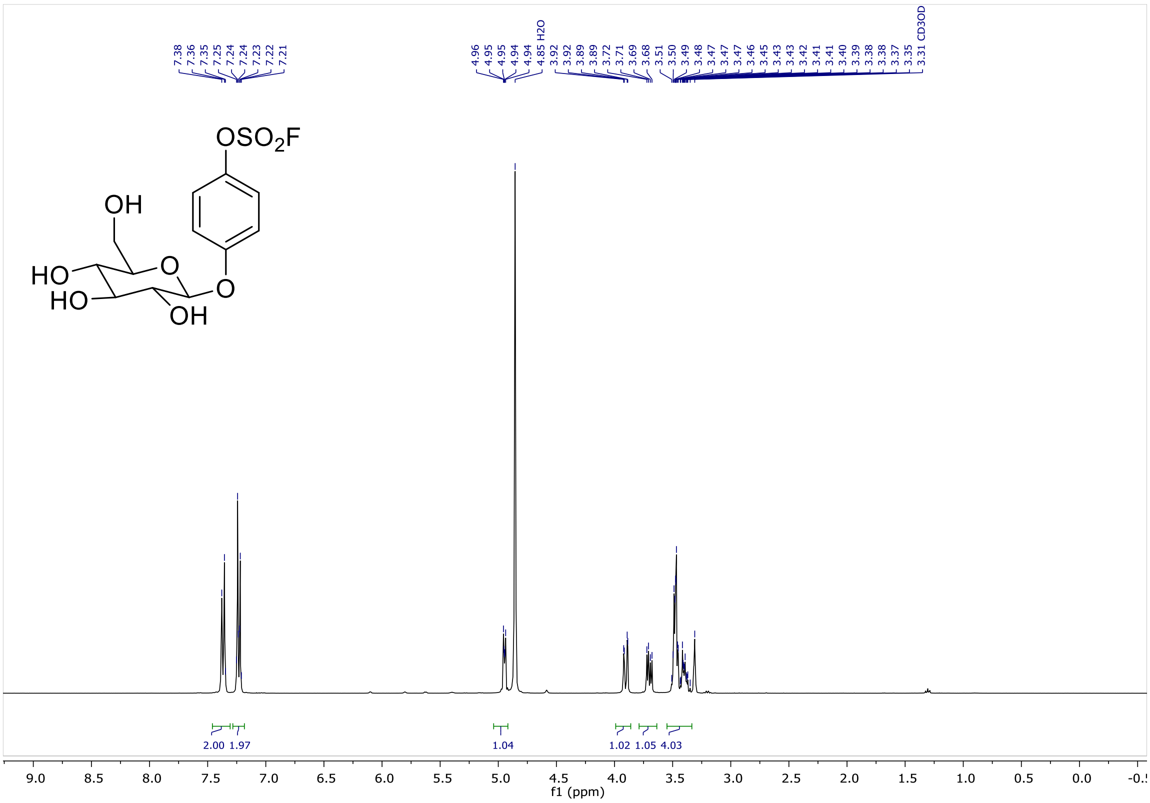

$^{13}C\{^{1}H\}$ NMR (101 MHz, CD_3OD)

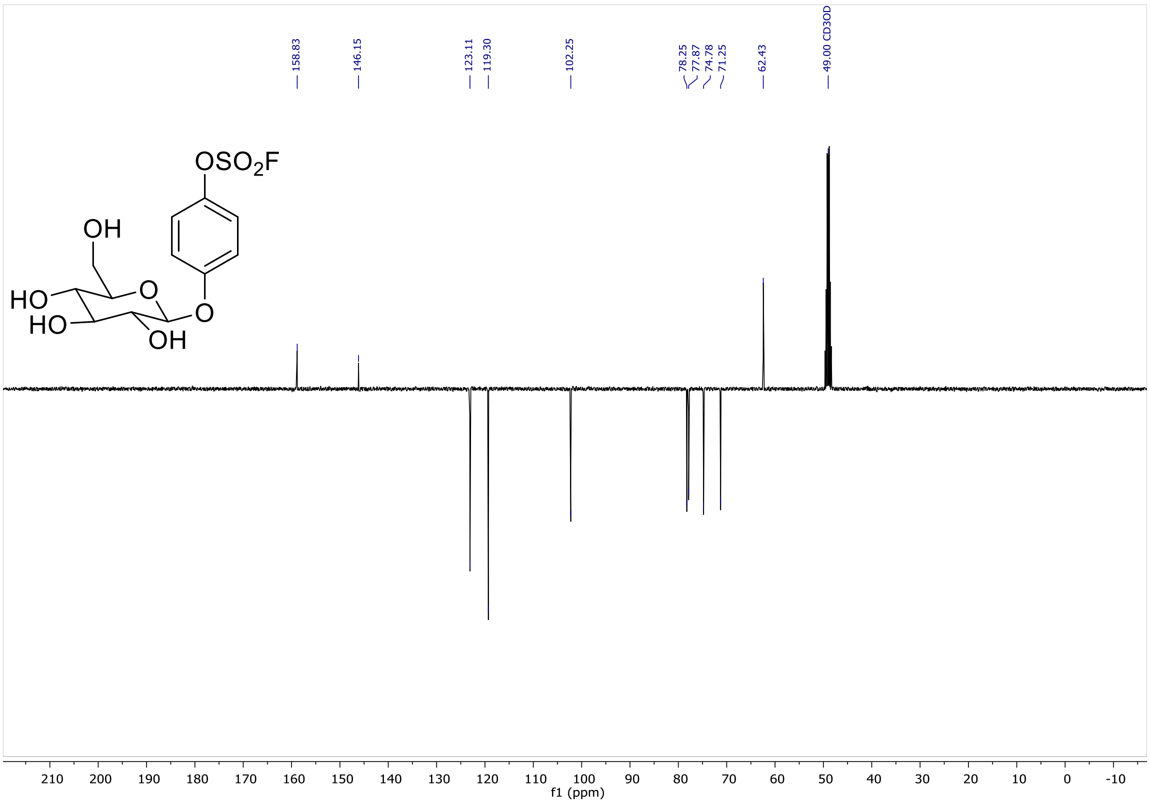

^{19}F NMR (376 MHz, CD_3OD)

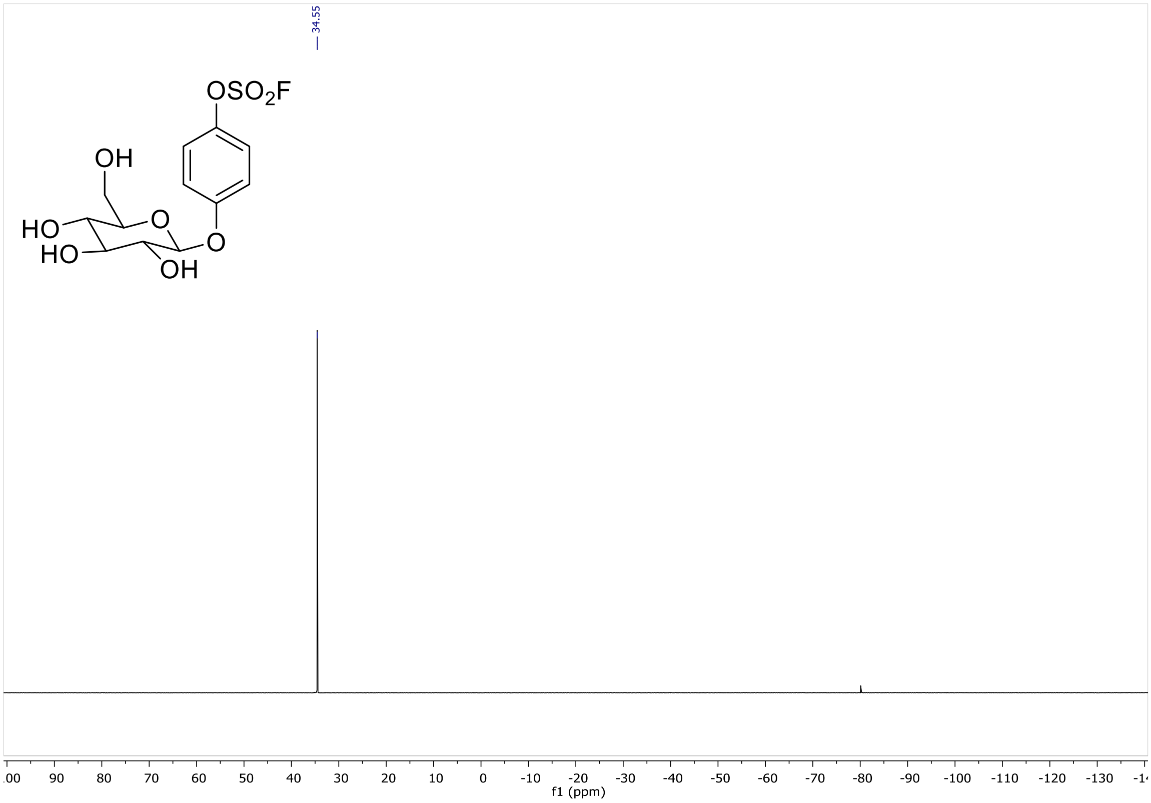

(*S,S*)-Ni-Cl3BPB-*m*-Tyr

^{1}H NMR (400 MHz, $CDCl_3$)

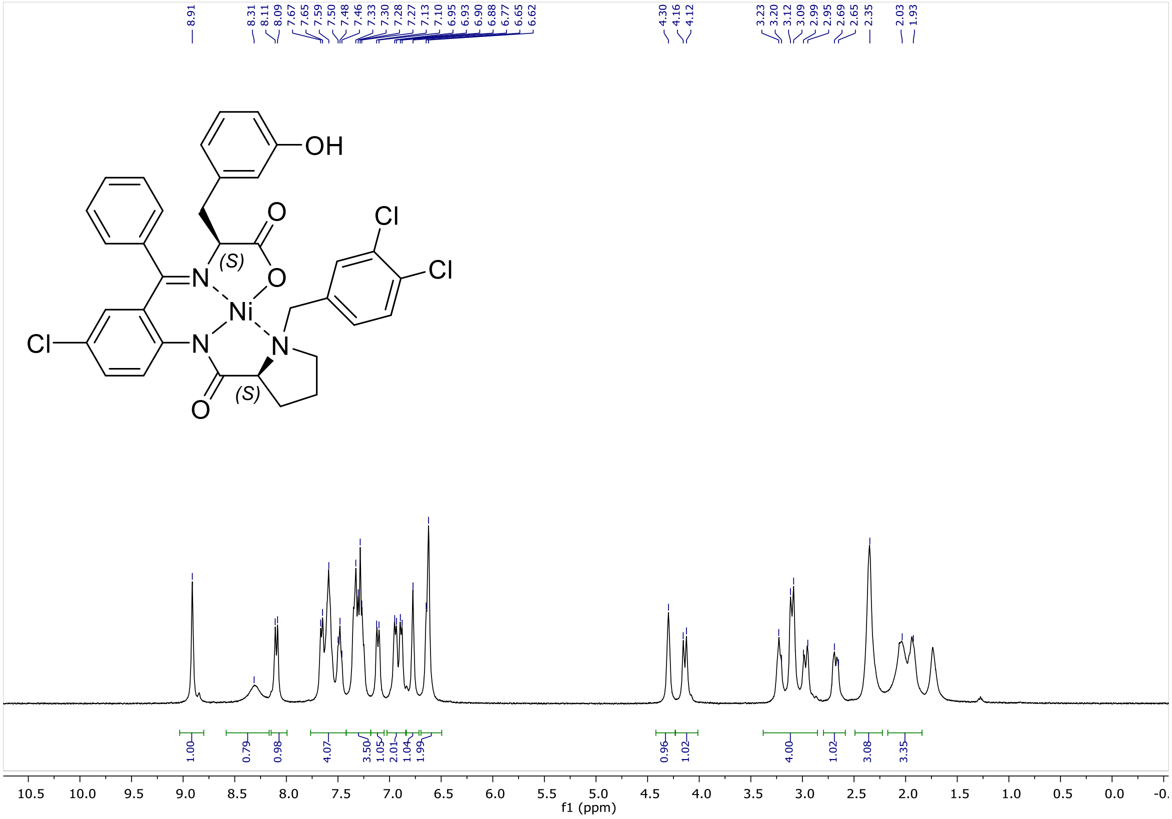

^{13}C{^{1}H} NMR (101 MHz, $CDCl_3$)

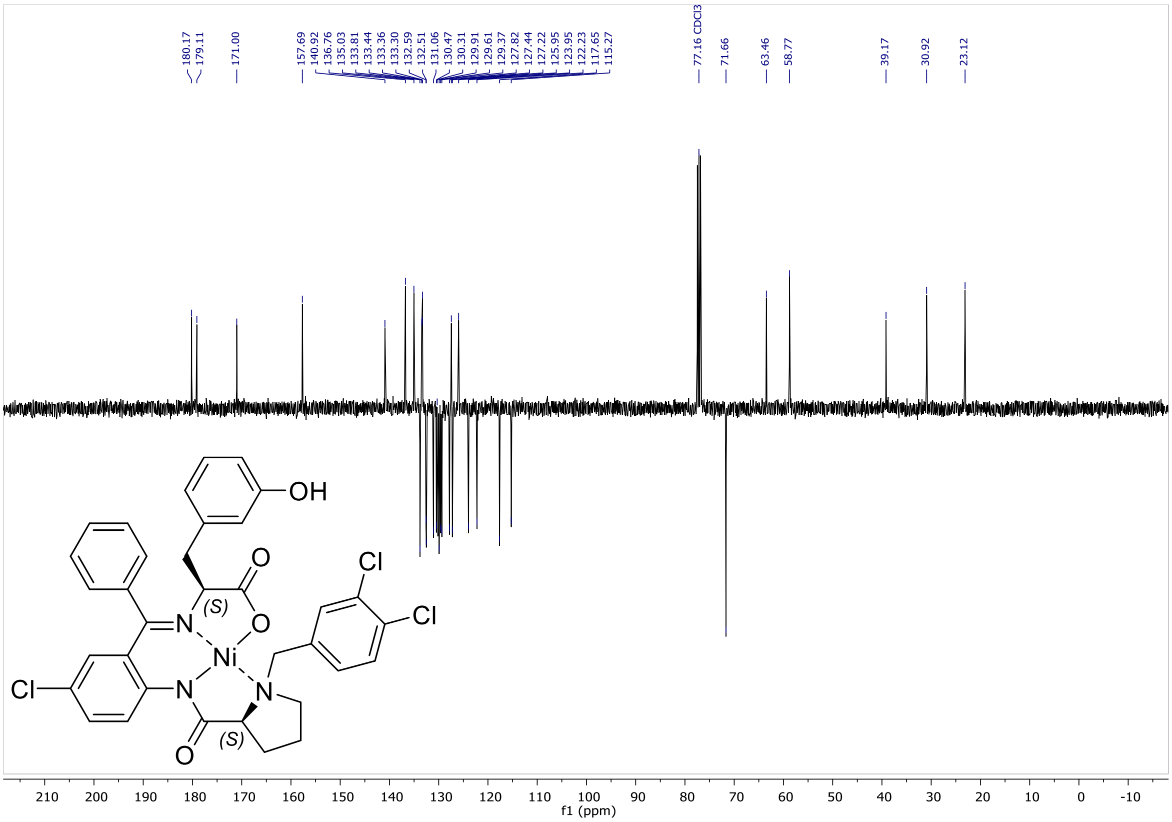

(*S,S*)-Ni-Cl3BPB-*m*-Tyr sulfurofluoridate (12j)

^{1}H NMR (400 MHz, $CDCl_3$)

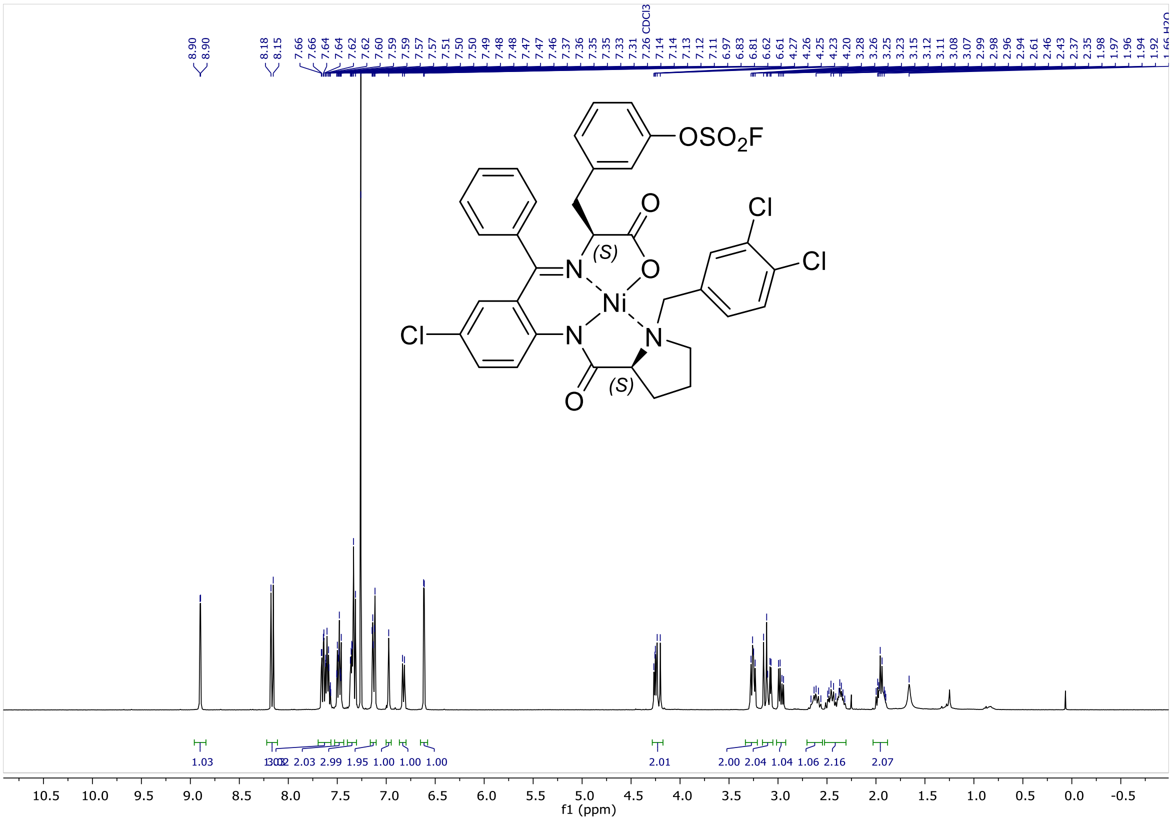

$^{13}C\{^{1}H\}$ NMR (101 MHz, $CDCl_3$)

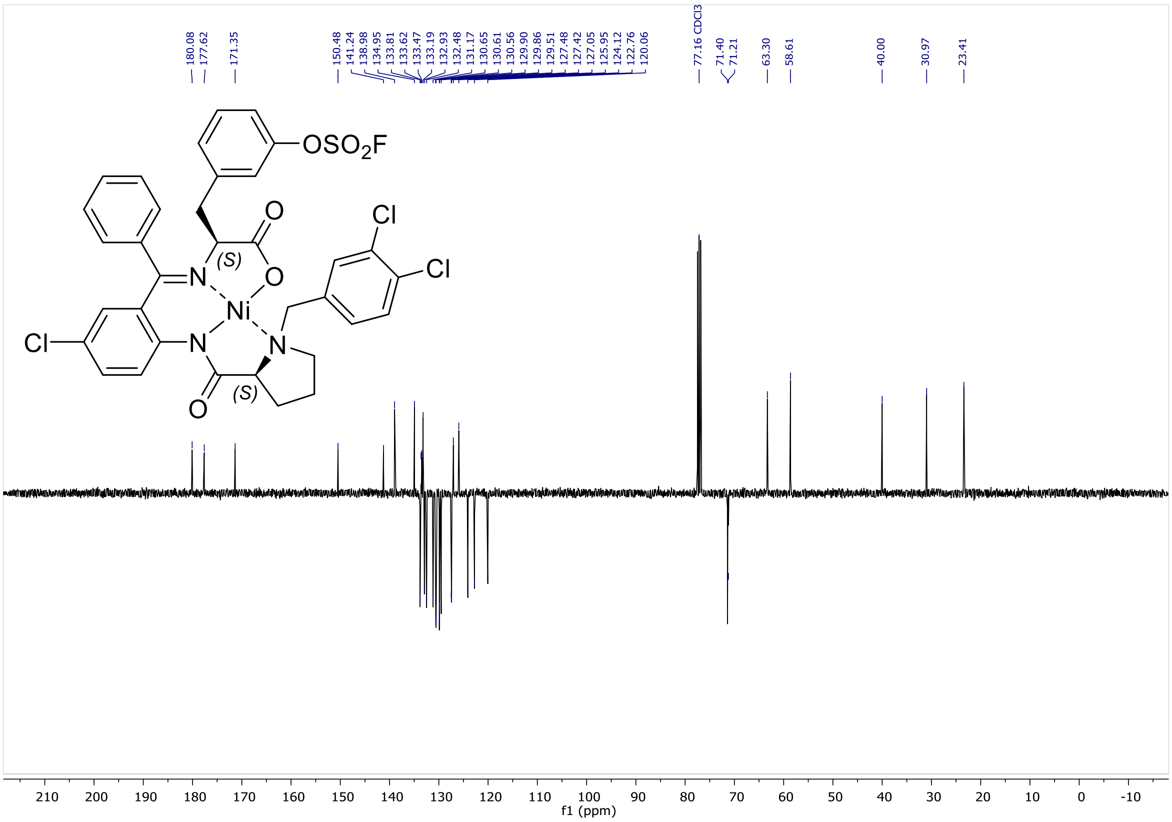

^{19}F NMR (376 MHz, $CDCl_3$)

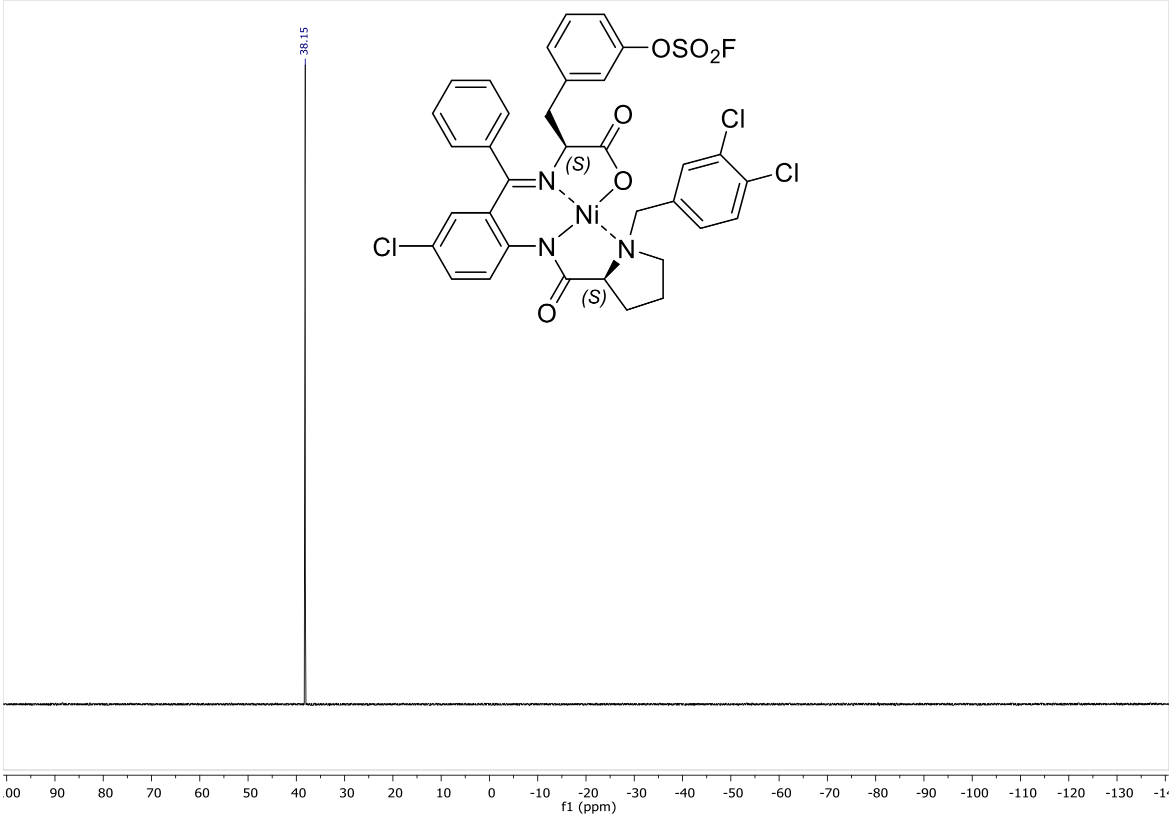

4-{(2*S*,3*R*)-1-(4-Fluorophenyl)-3-[(*S*)-3-(4-fluorophenyl)-3-hydroxypropyl]-4-oxoazetidin-2-yl}phenyl sulfurofluoridate (12k)

^{1}H NMR (400 MHz, $CDCl_3$)

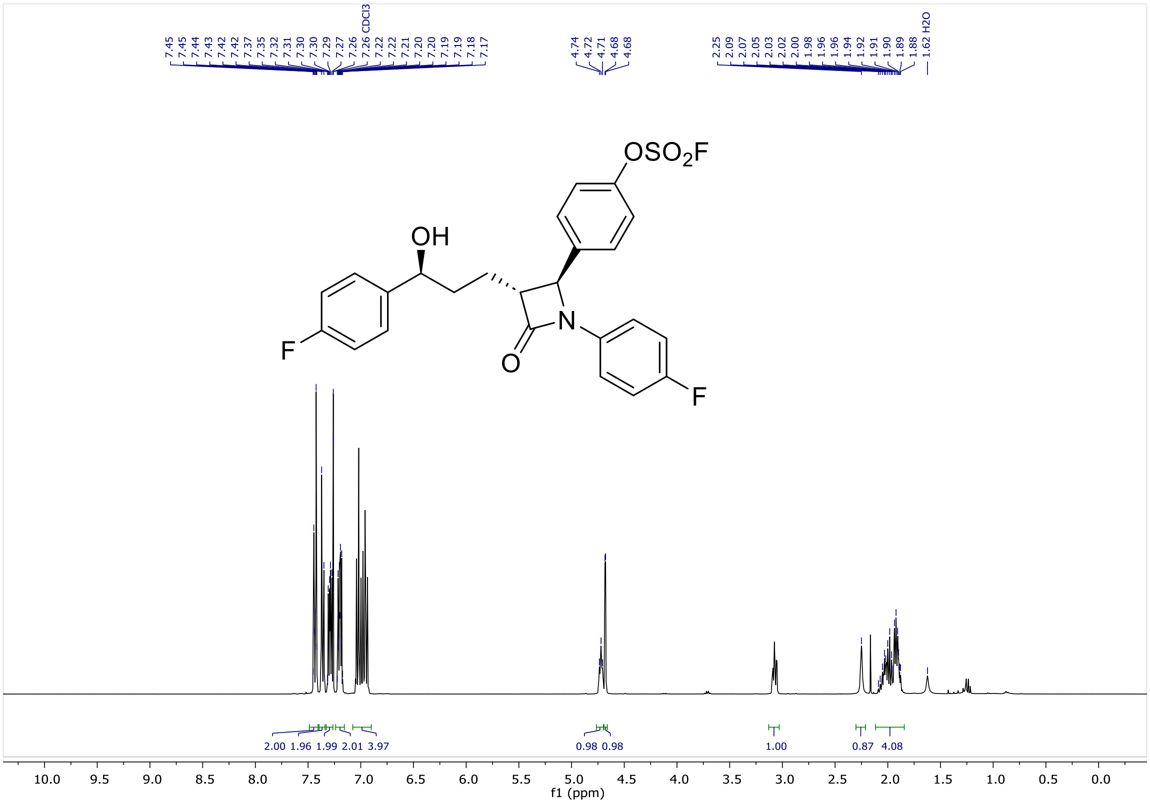

^{13}C{^{1}H} NMR (101 MHz, $CDCl_3$)

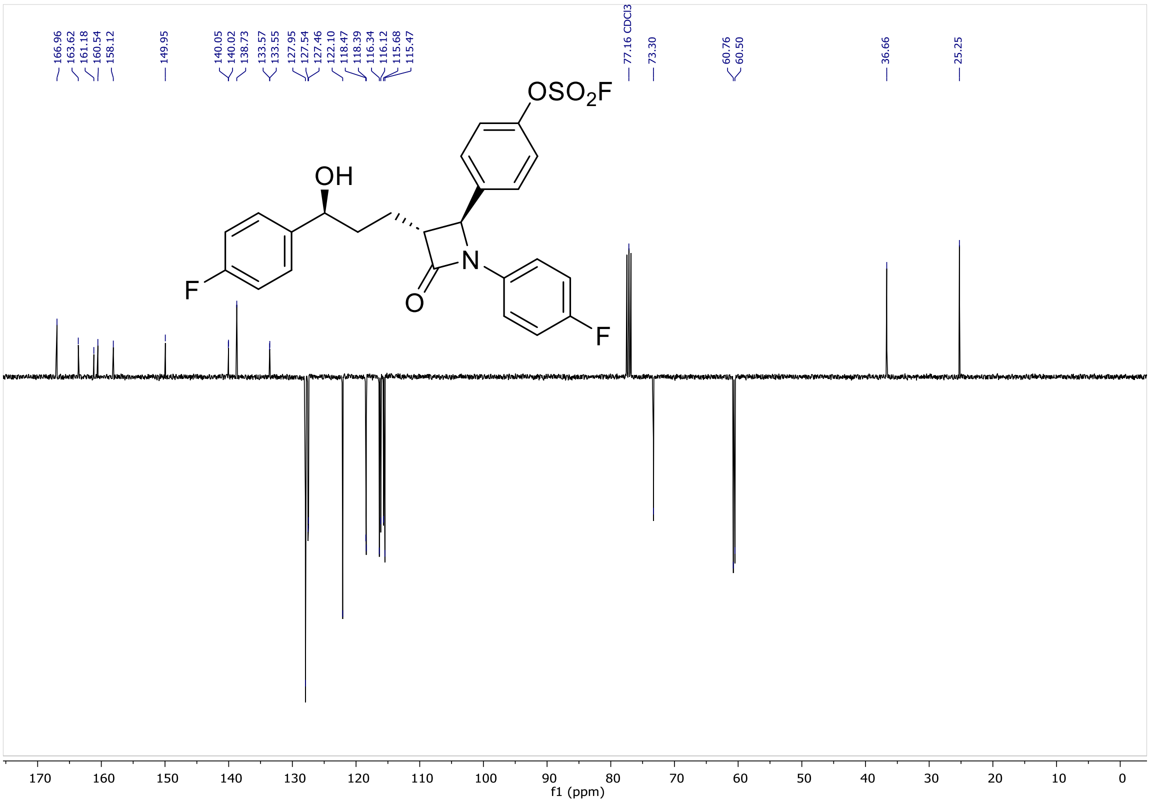

^{19}F NMR (376 MHz, $CDCl_3$)

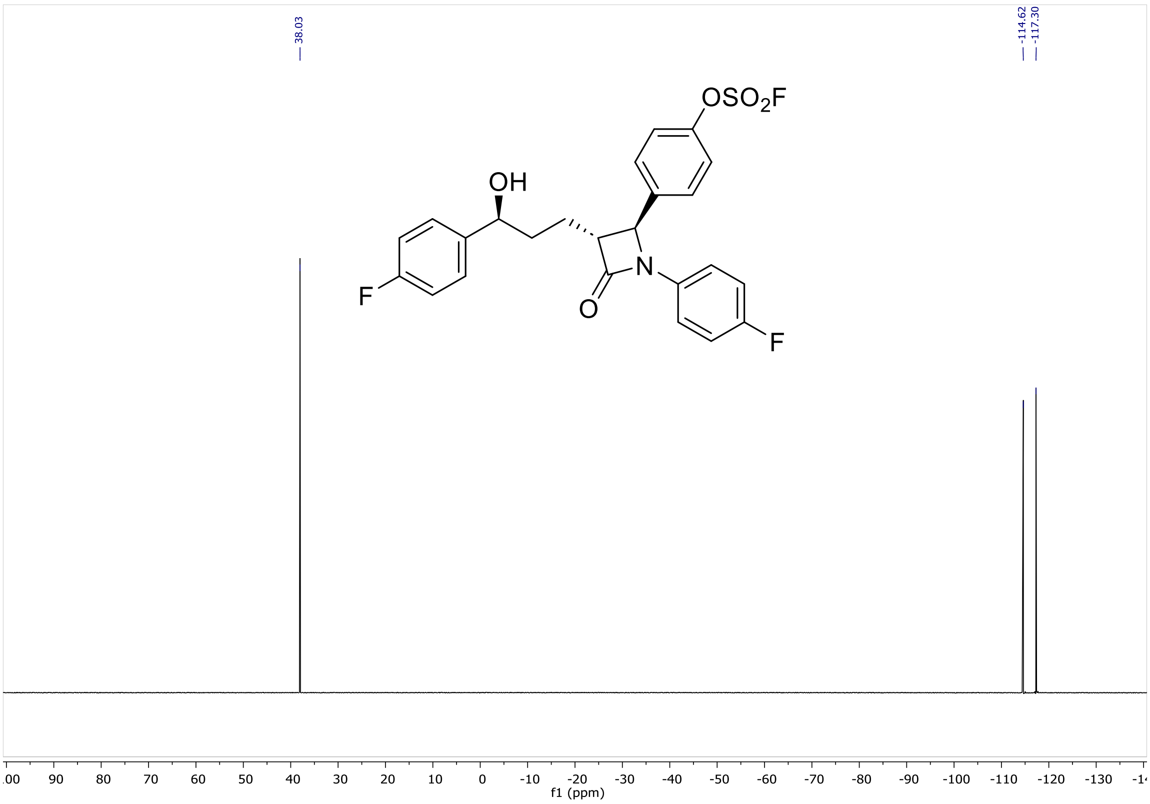

(*S*)-4-Ethyl-4-hydroxy-3,14-dioxo-3,4,12,14-tetrahydro-1*H*-pyrano[3',4':6,7] indolizino[1,2-b]quinolin-9-yl sulfurofluoridate (12l)

^{1}H NMR [400 MHz, $(CD_3)_2SO$]

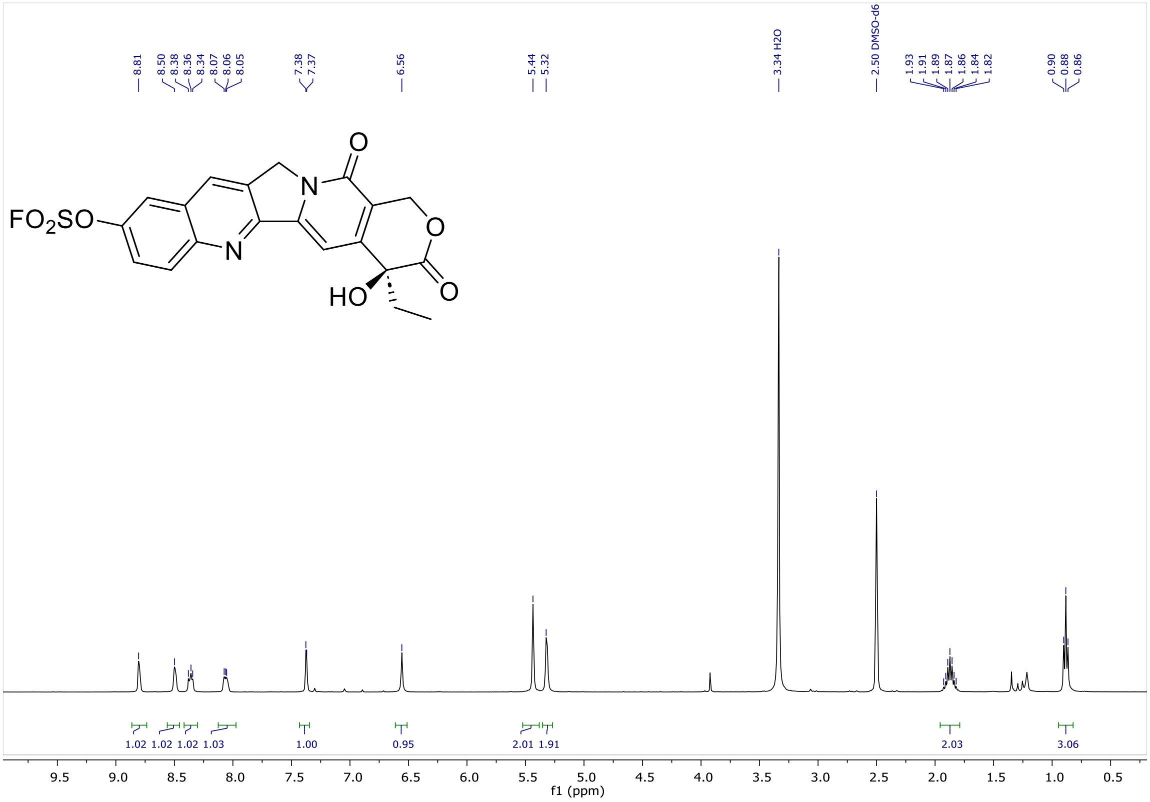

^{13}C{^{1}H} NMR [101 MHz, $(CD_3)_2SO$]

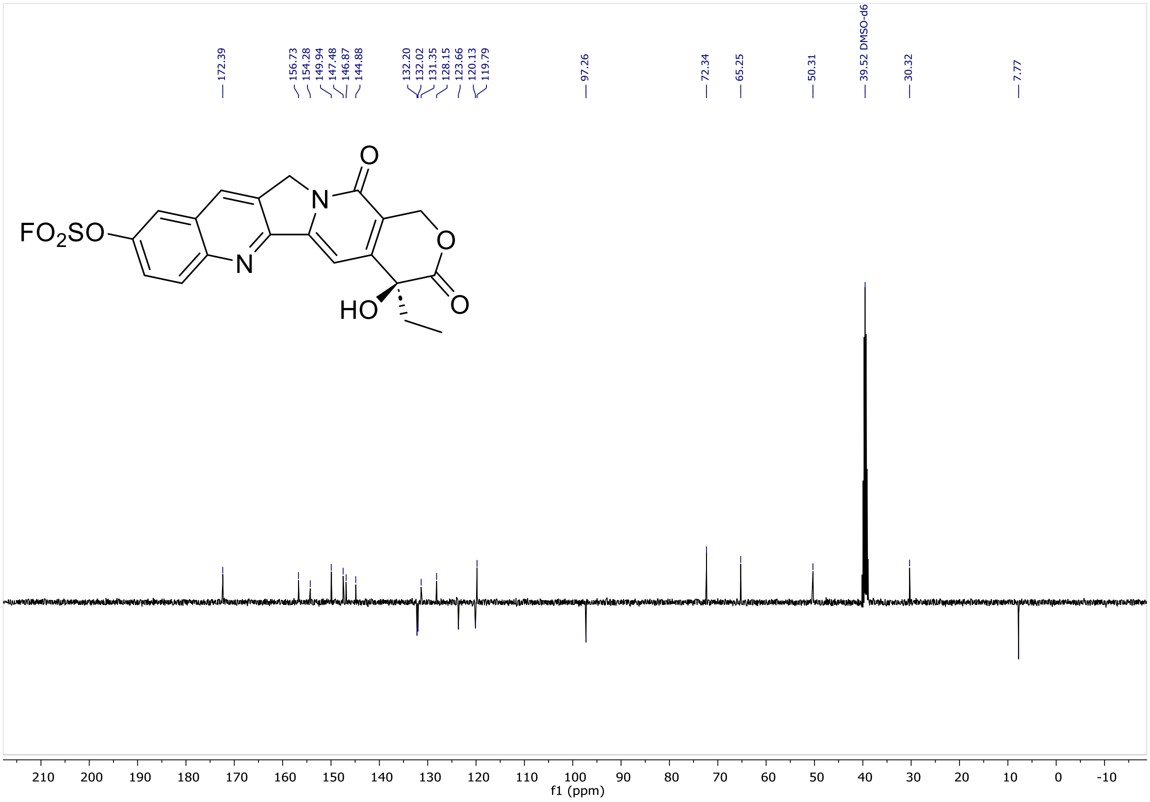

^{19}F NMR [376 MHz, $(CD_3)_2SO$]

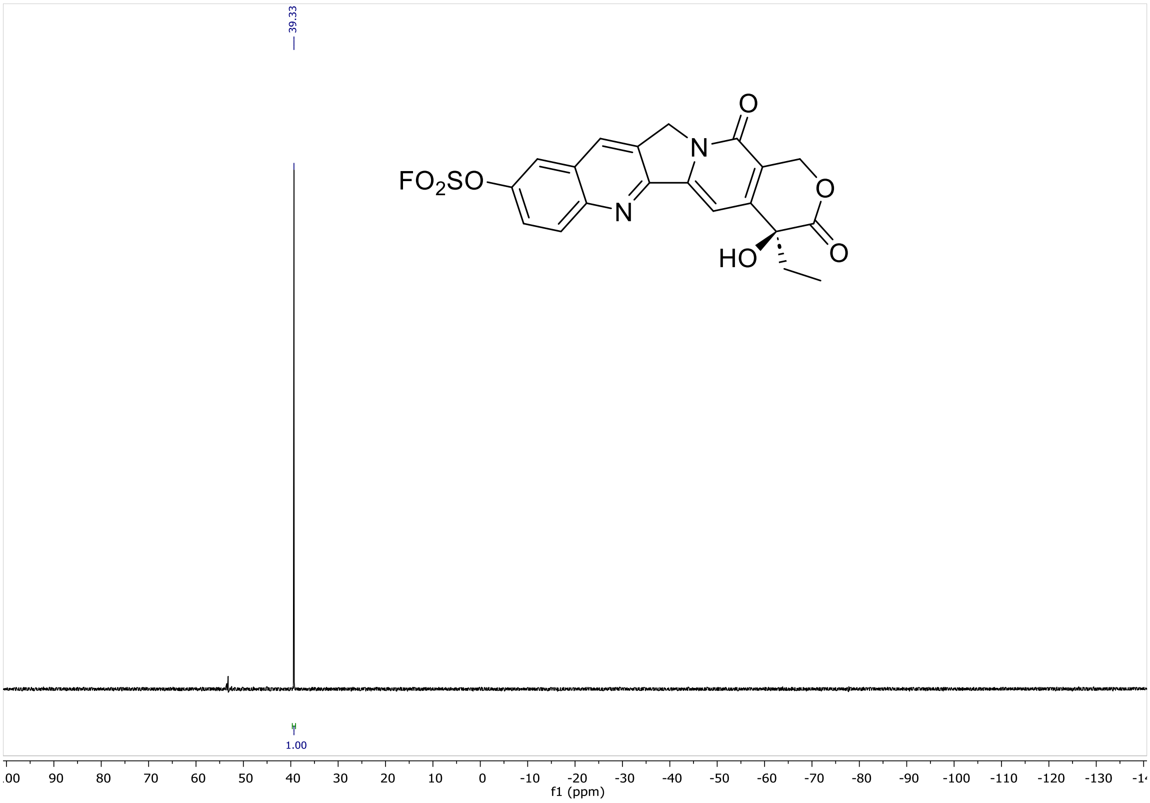

4-{3-[2-(Diethylamino)-2-oxoethyl]-5,7-dimethylpyrazolo[1,5-a]pyrimidin-2-yl}phenyl sulfurofluoridate (12m)

^{1}H NMR (400 MHz, $CDCl_3$)

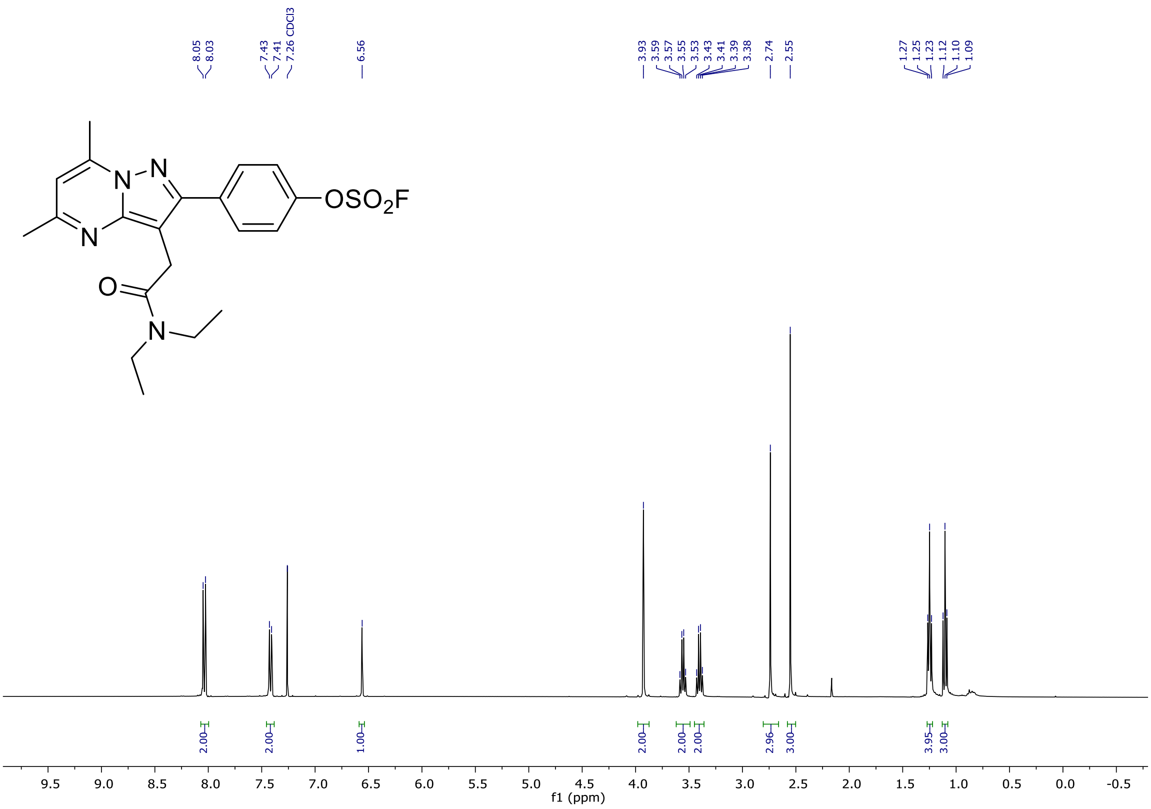

$^{13}C\{^{1}H\}$ NMR (101 MHz, $CDCl_3$)

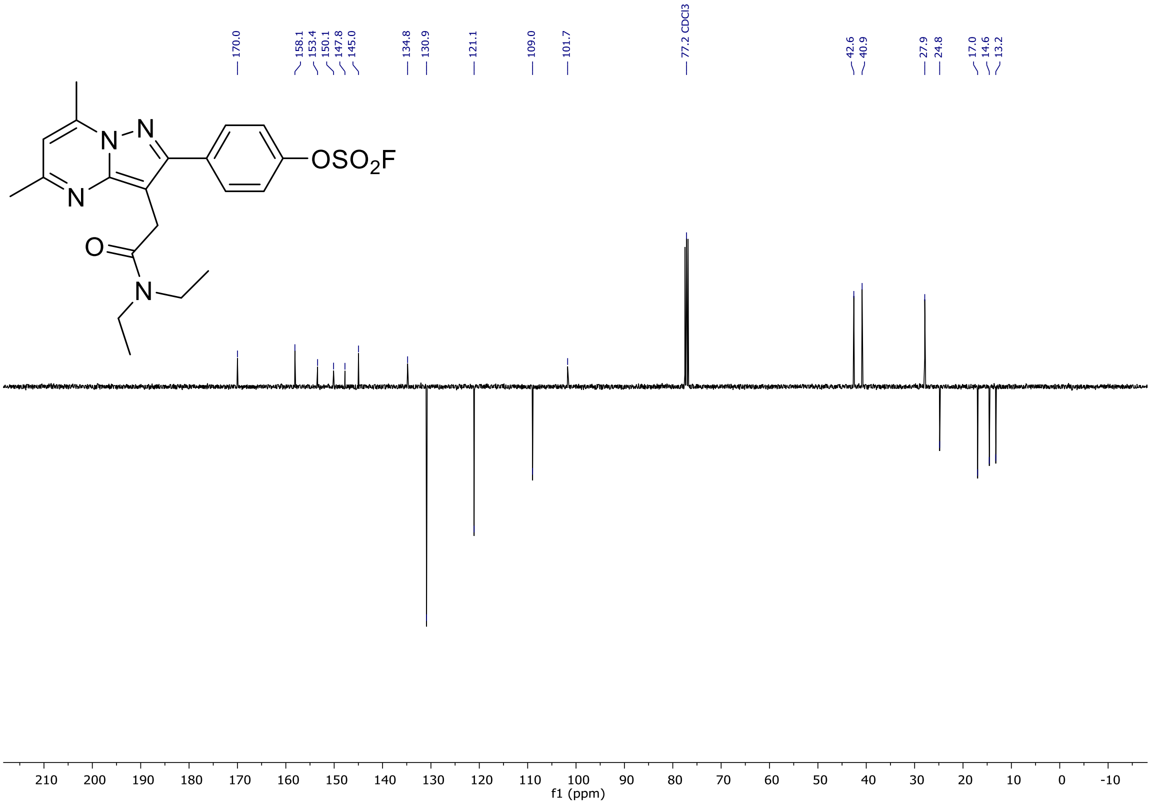

^{19}F NMR (376 MHz, $CDCl_3$)

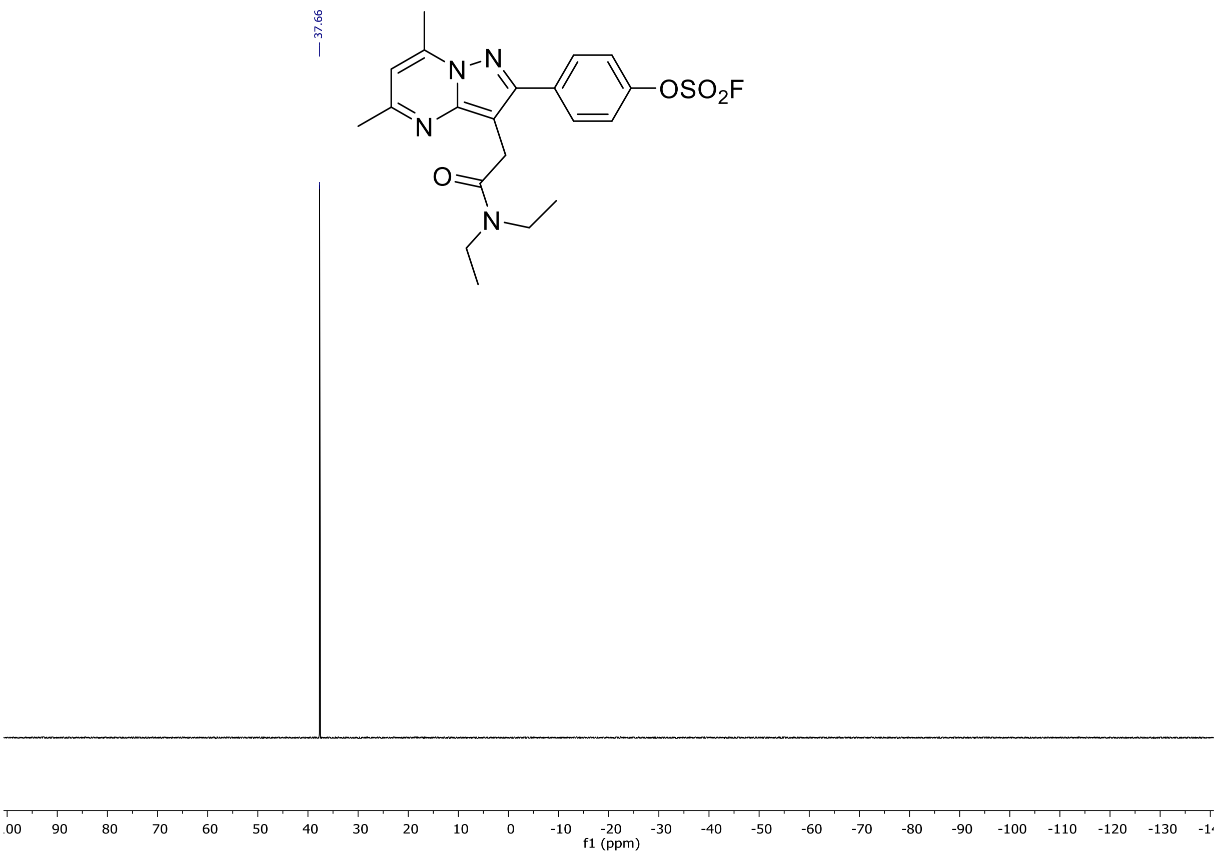

2,3,5,6-Tetrafluorophenyl 3-(4-hydroxyphenyl)propanoate

^{1}H NMR (400 MHz, $CDCl_3$)

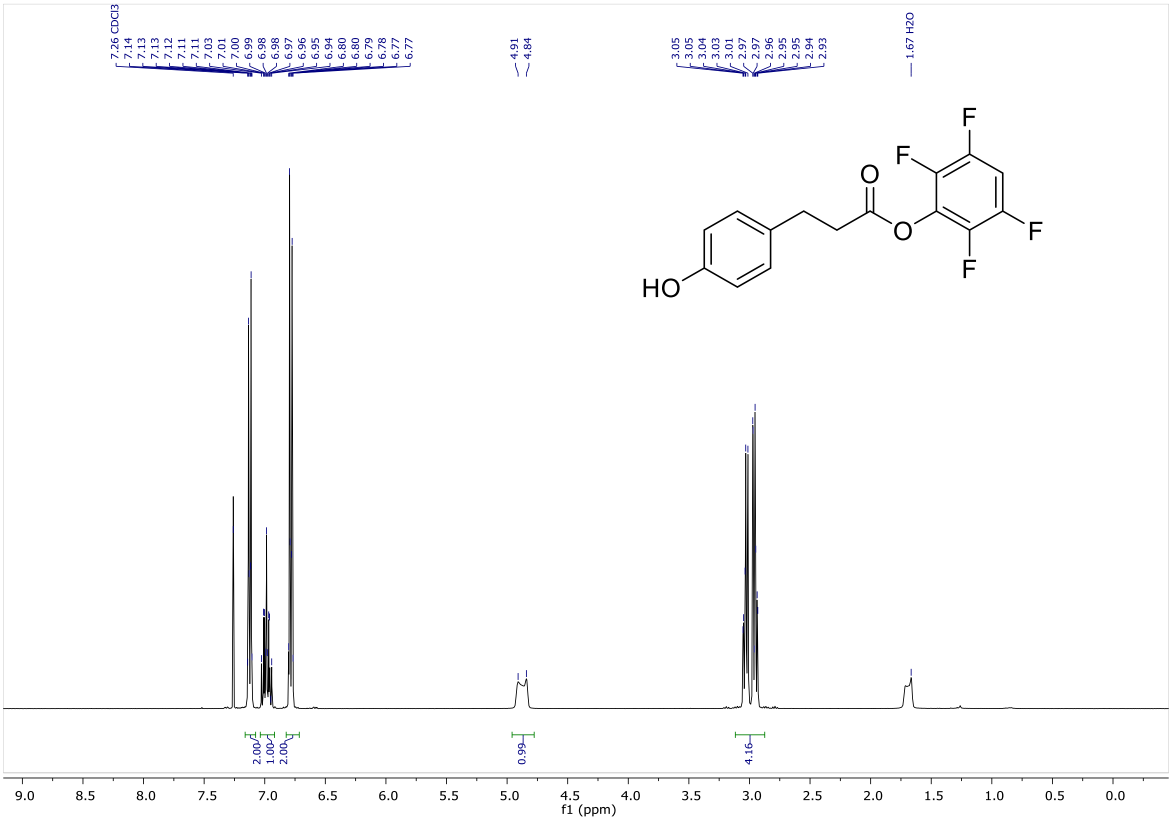

$^{13}C\{^1H\}$ NMR (101 MHz, $CDCl_3$)

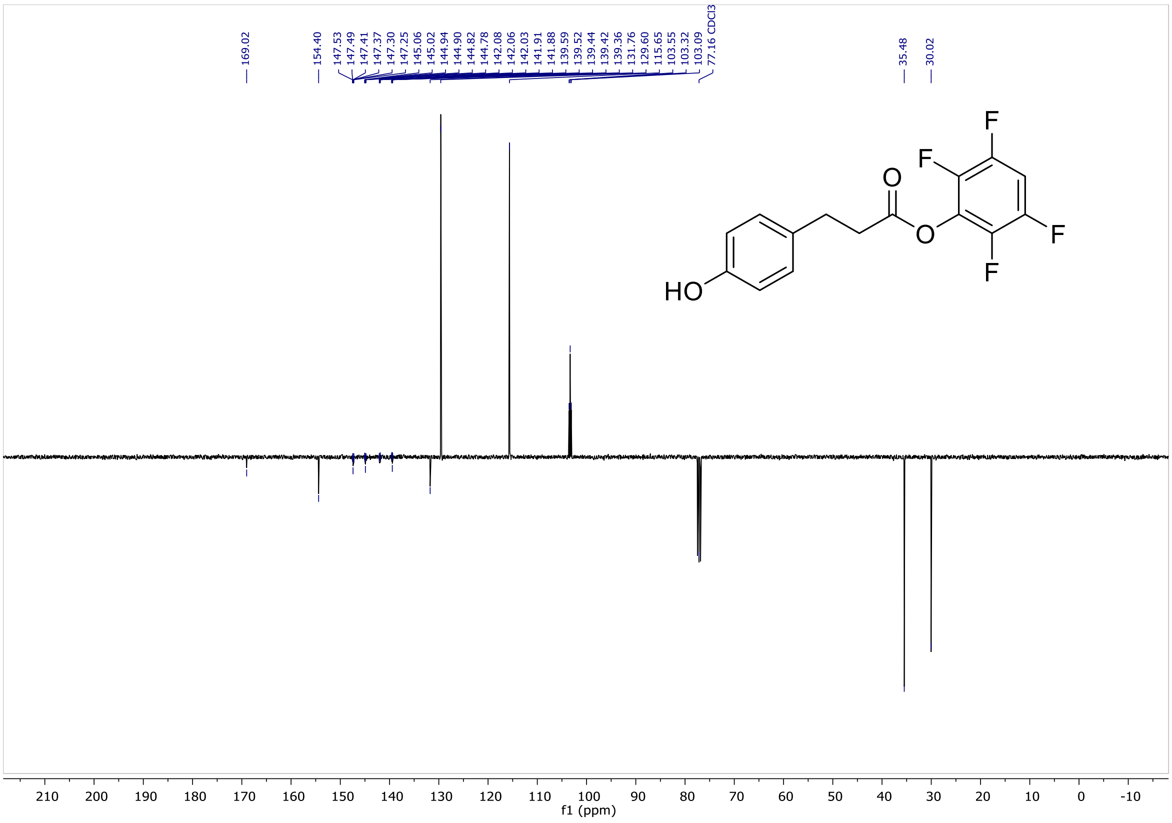

^{19}F NMR (376 MHz, $CDCl_3$)

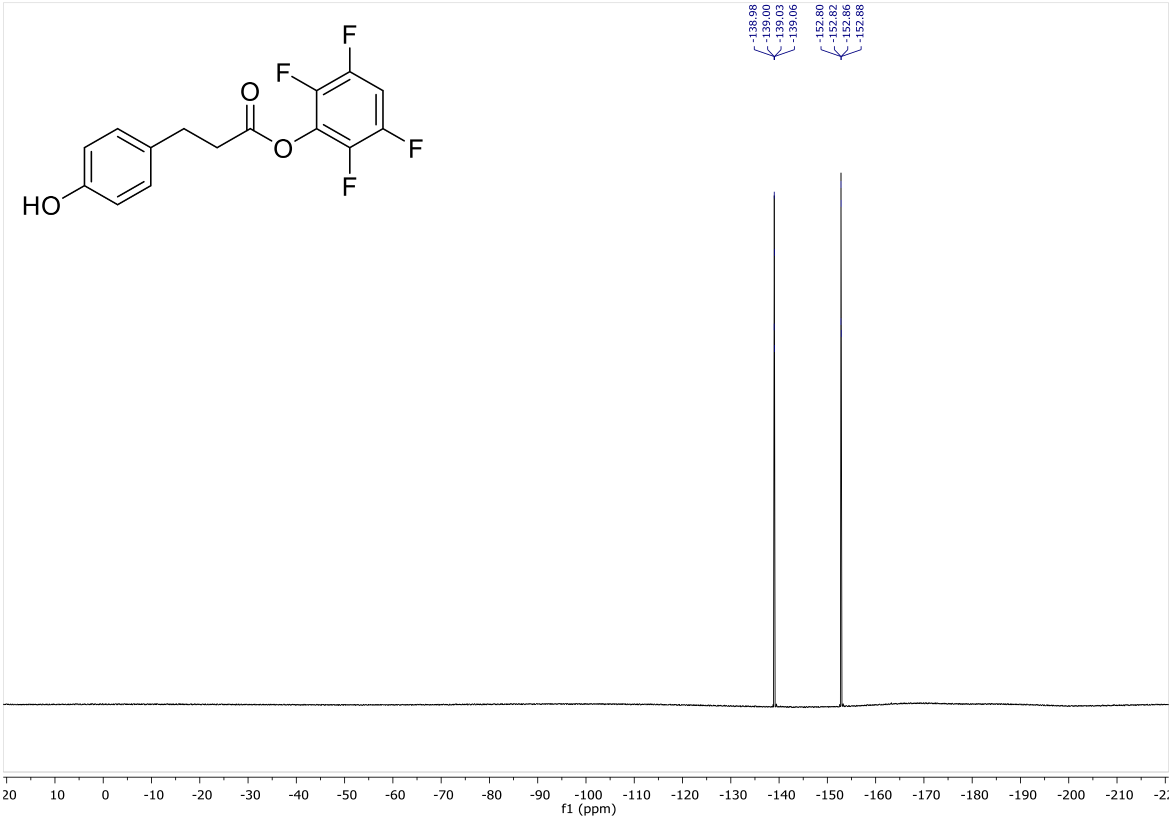

2,3,5,6-Tetrafluorophenyl 3-{4-[(fluorosulfonyl)oxy]phenyl}propanoate (12n)

^{1}H NMR (400 MHz, $CDCl_3$)

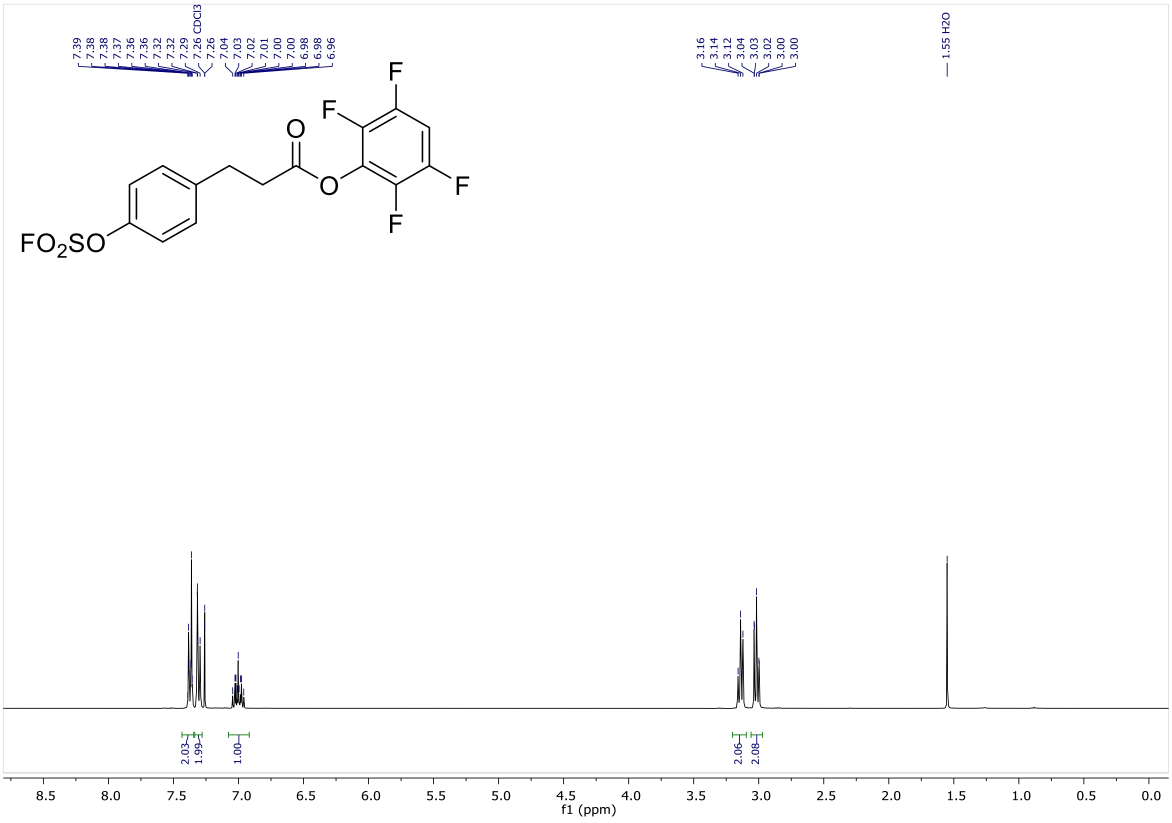

$^{13}C\{^{1}H\}$ NMR (101 MHz, $CDCl_3$)

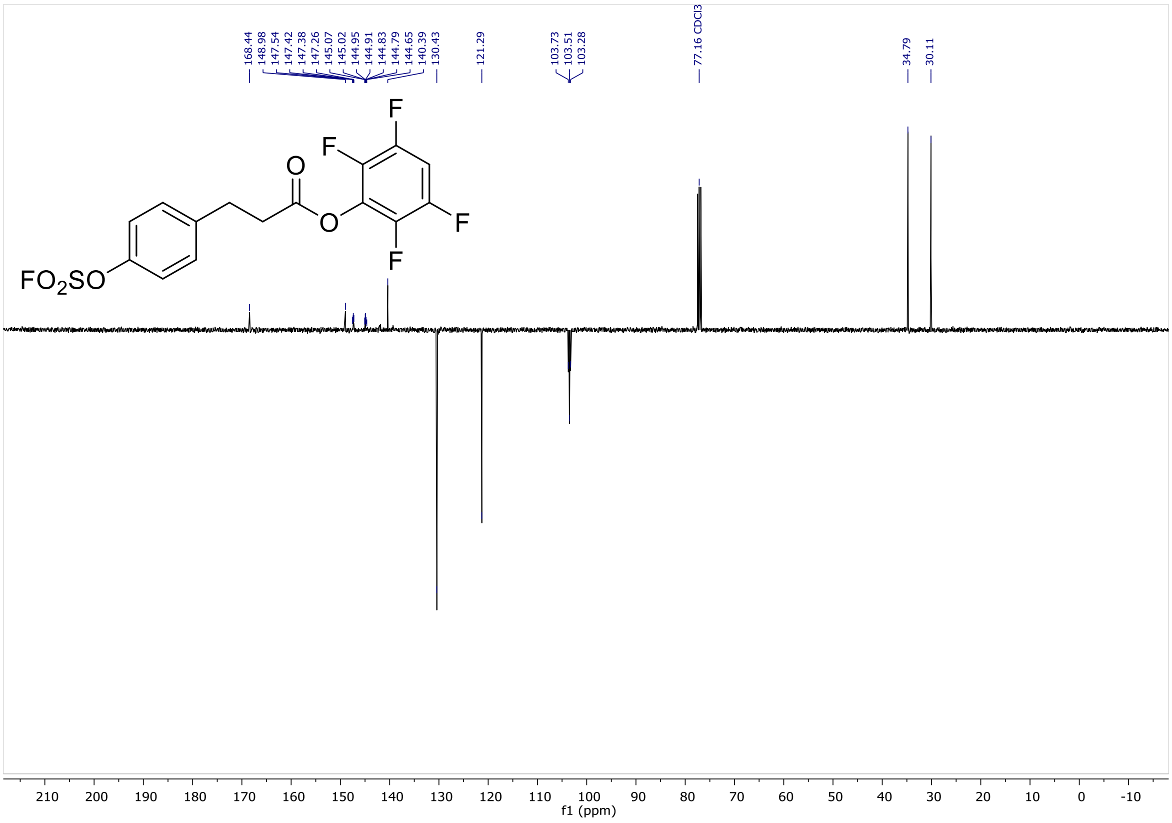

^{19}F NMR (376 MHz, $CDCl_3$)

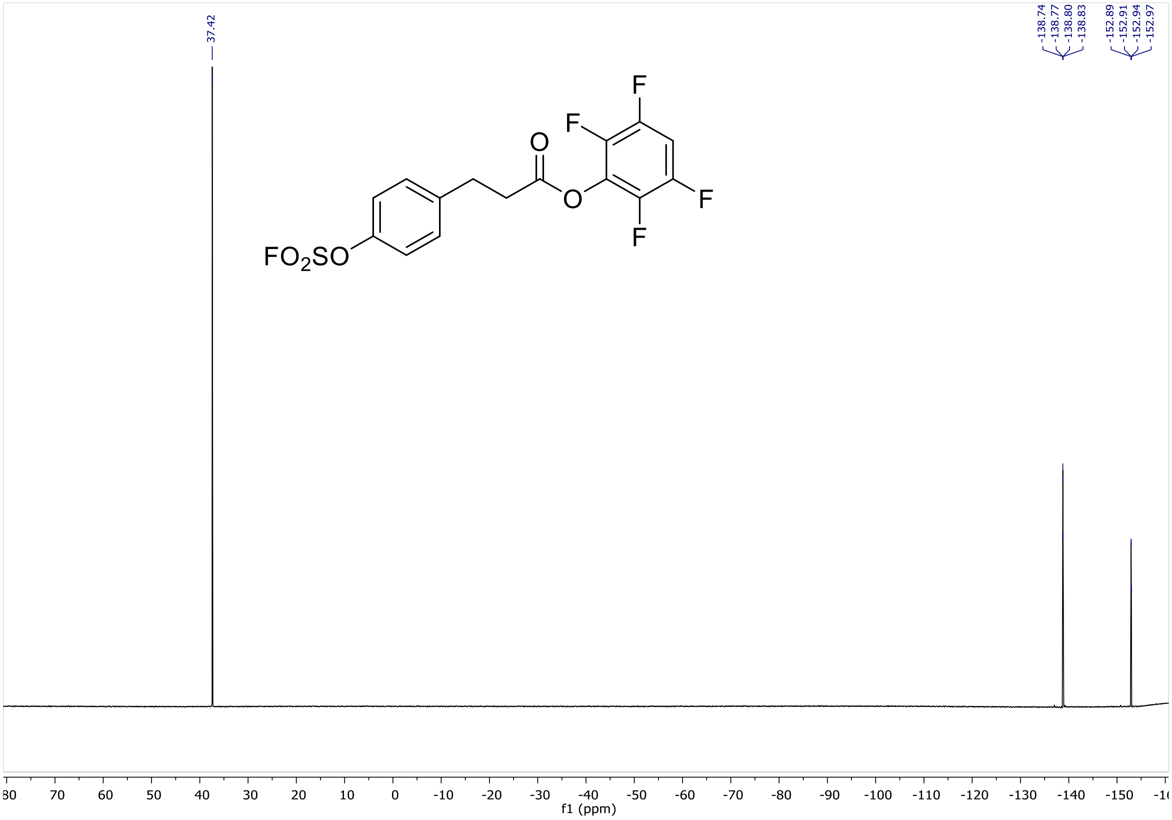

Methyl(phenyl)sulfamoyl fluoride (13a)

^{1}H NMR (400 MHz, $CDCl_3$)

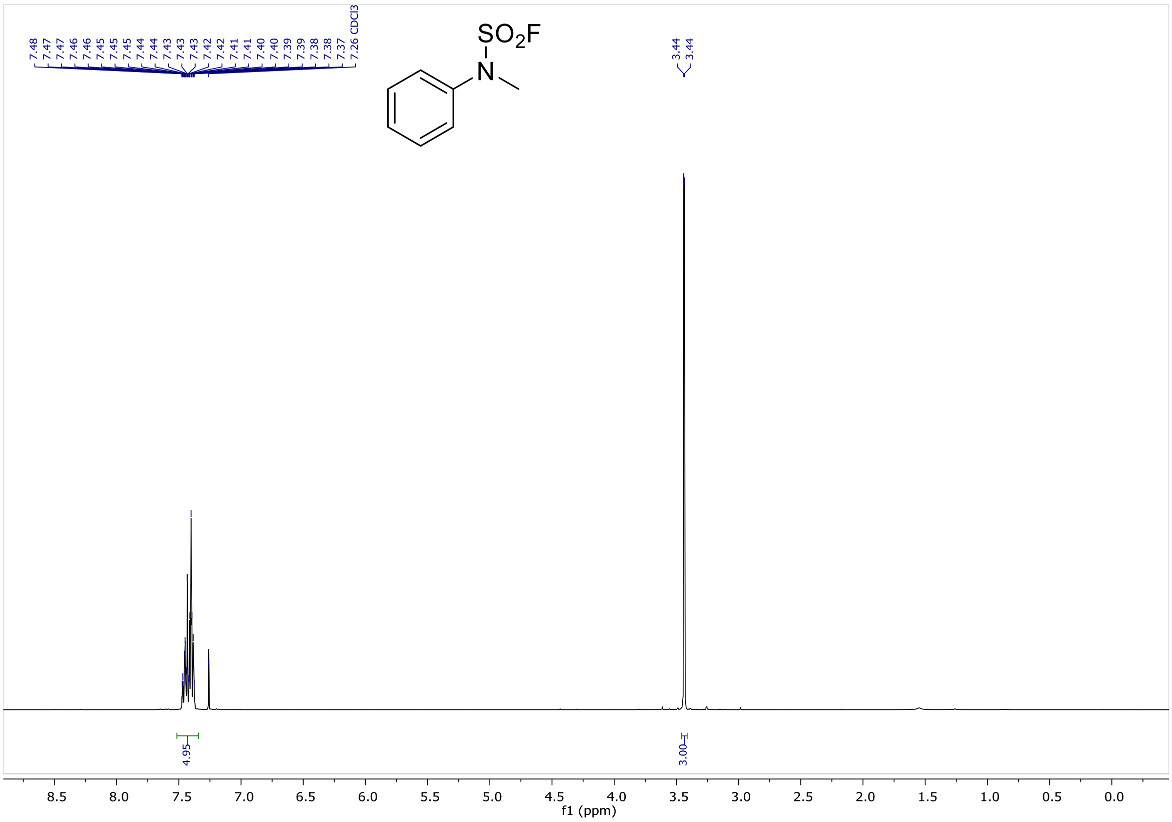

$^{13}C\{^1H\}$ NMR (101 MHz, $CDCl_3$)

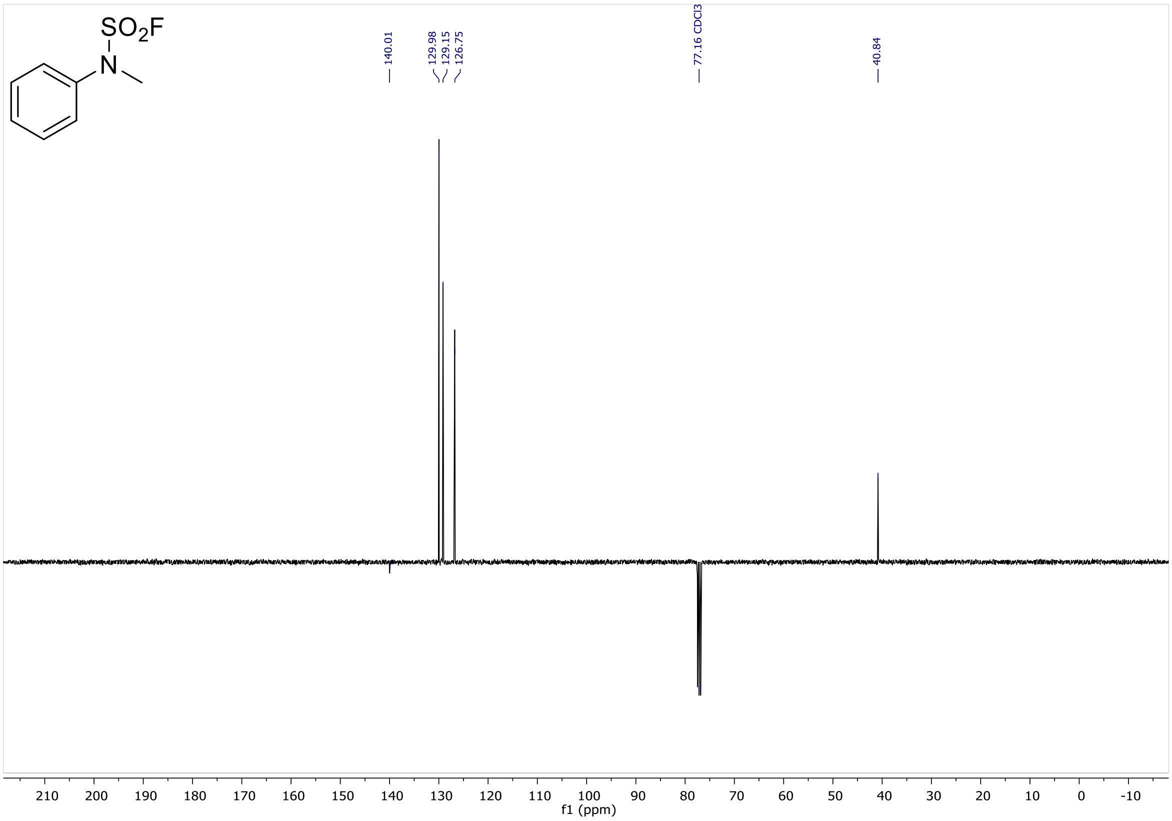

^{19}F NMR (376 MHz, $CDCl_3$)

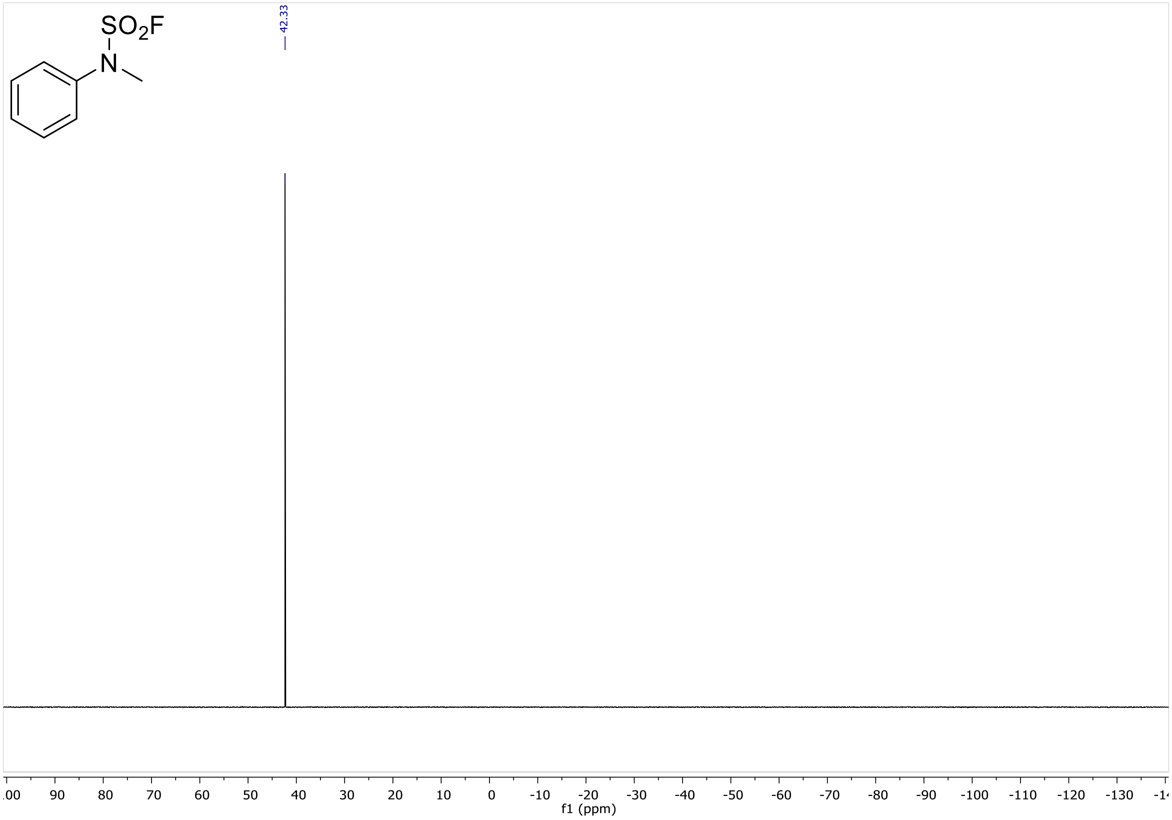

1,2,3,4-Tetrahydroisoquinoline-2-sulfonyl fluoride (13b)

^{1}H NMR (400 MHz, $CDCl_3$)

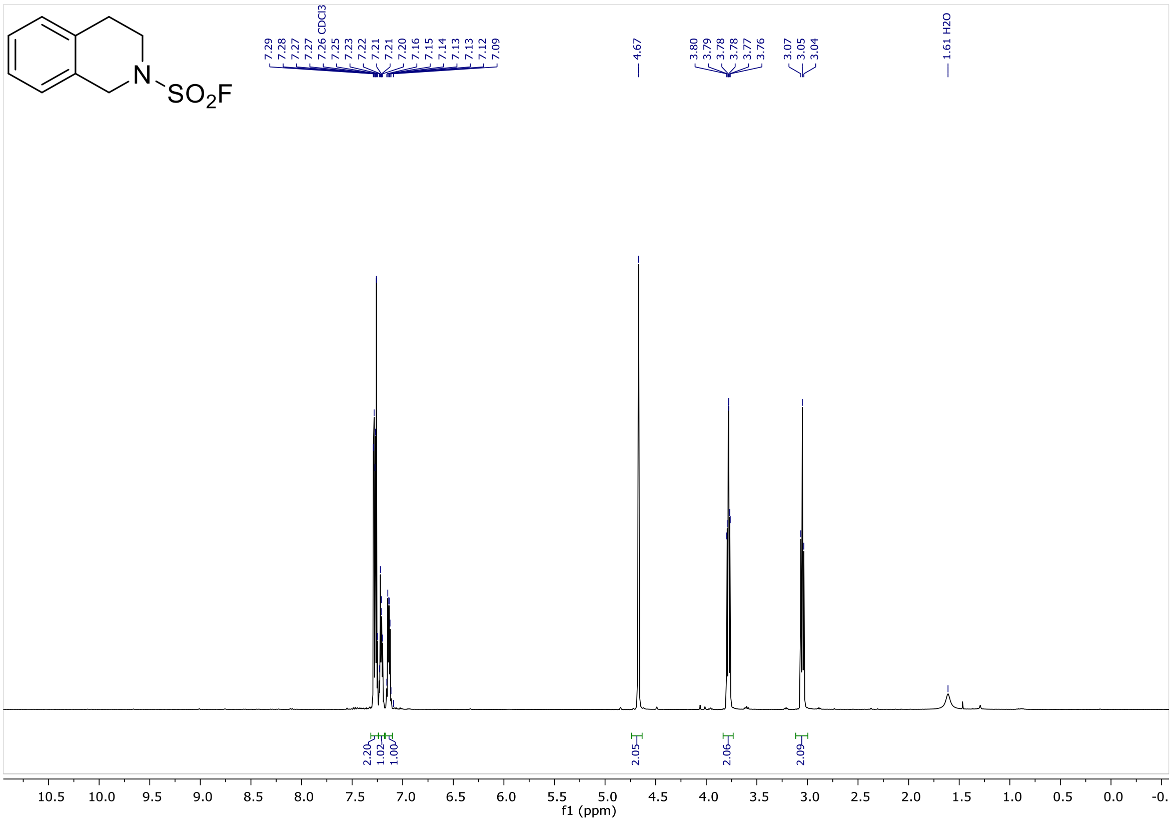

$^{13}C\{^{1}H\}$ NMR (101 MHz, $CDCl_3$)

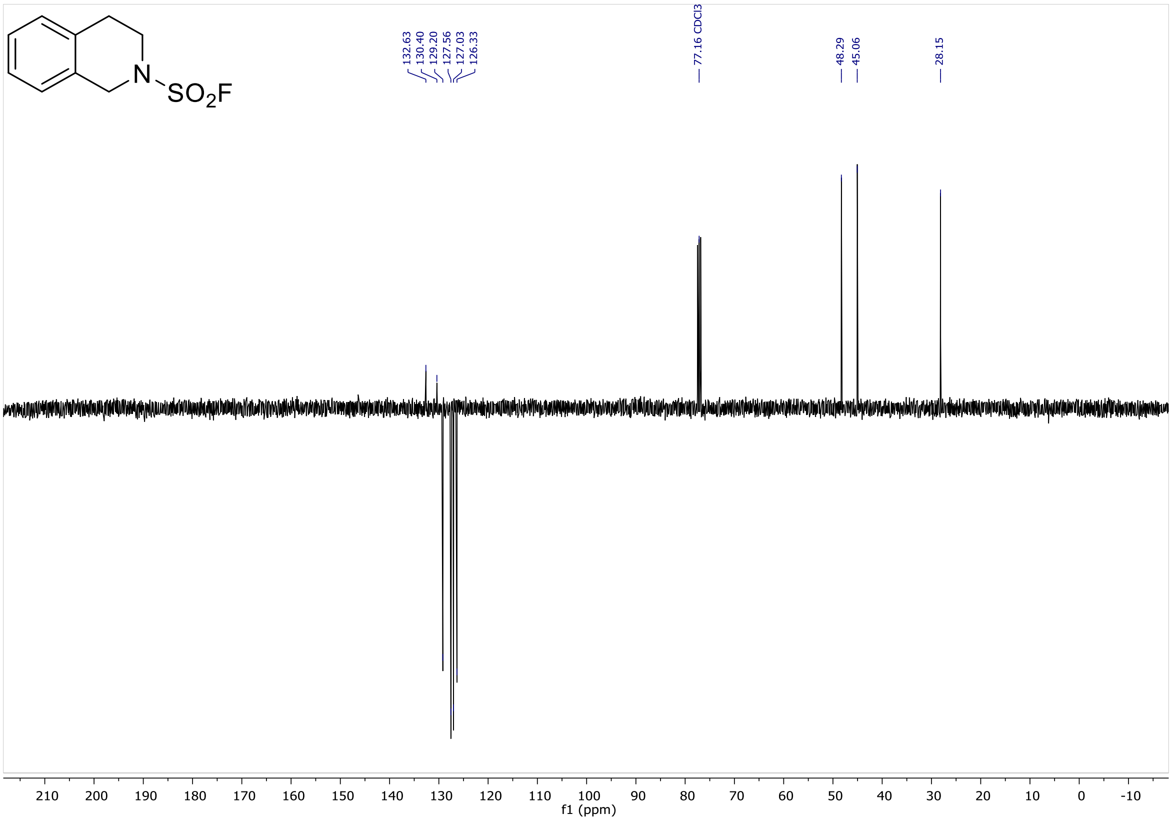

^{19}F NMR (376 MHz, $CDCl_3$)

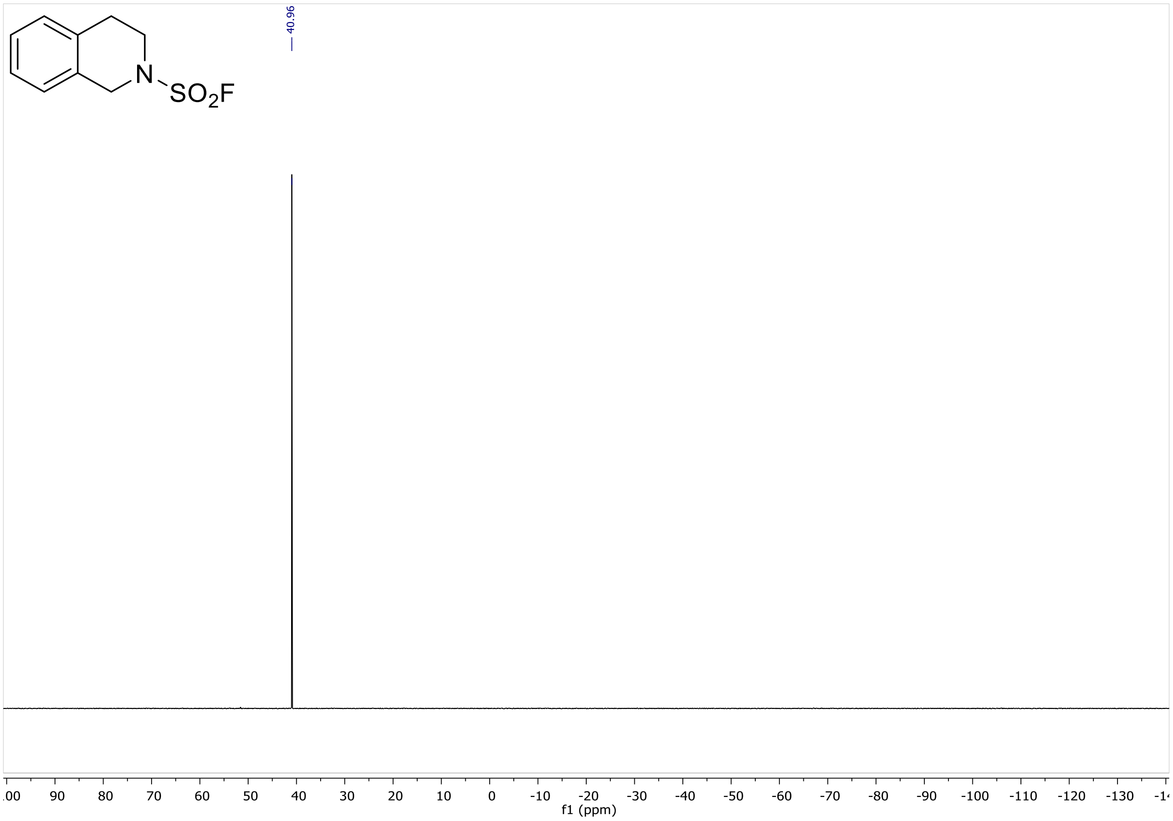

Indoline-1-sulfonyl fluoride (13c)

^{1}H NMR (400 MHz, $CDCl_3$)

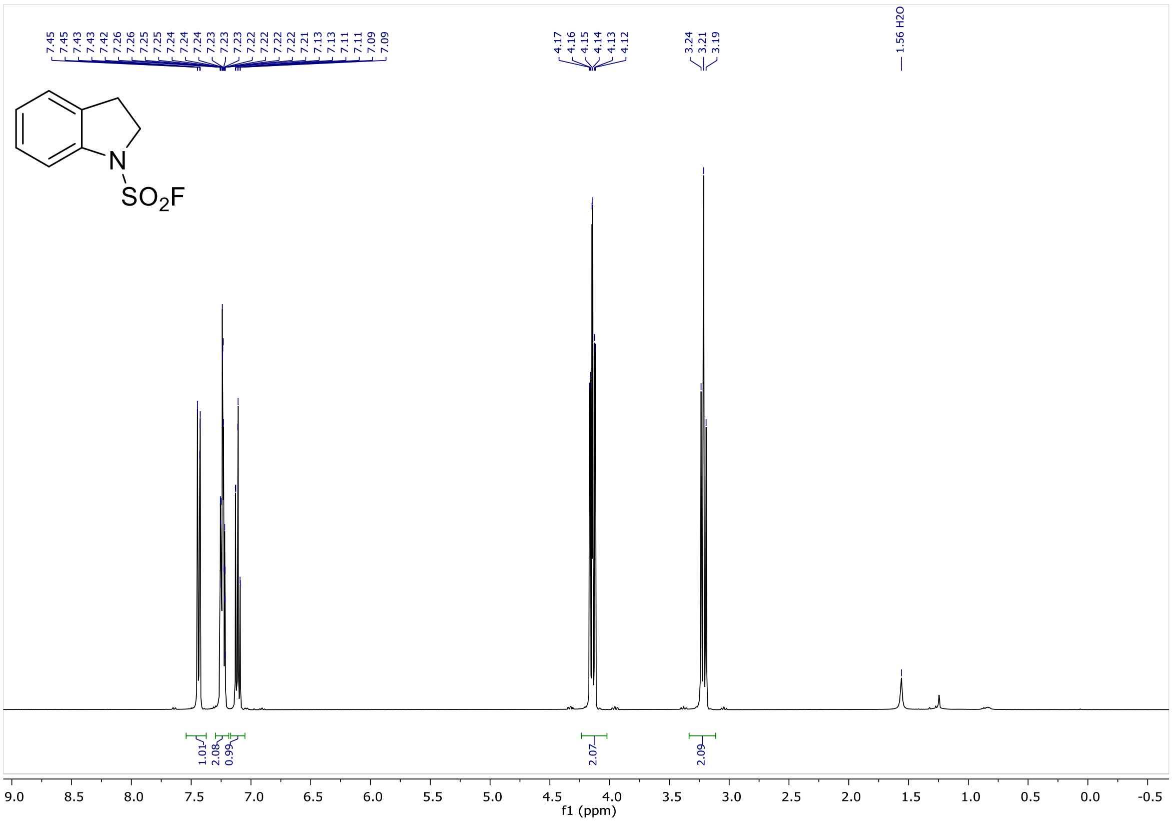

$^{13}C\{^{1}H\}$ NMR (101 MHz, $CDCl_3$)

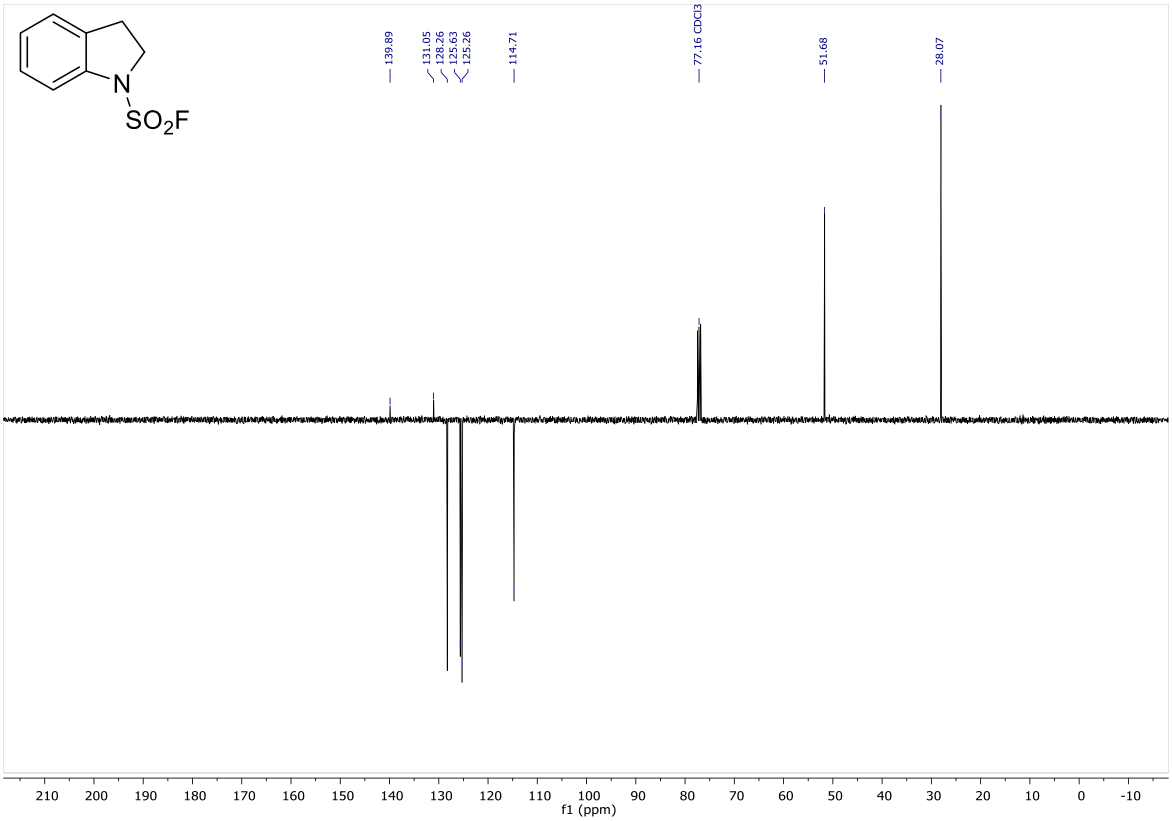

^{19}F NMR (376 MHz, $CDCl_3$)

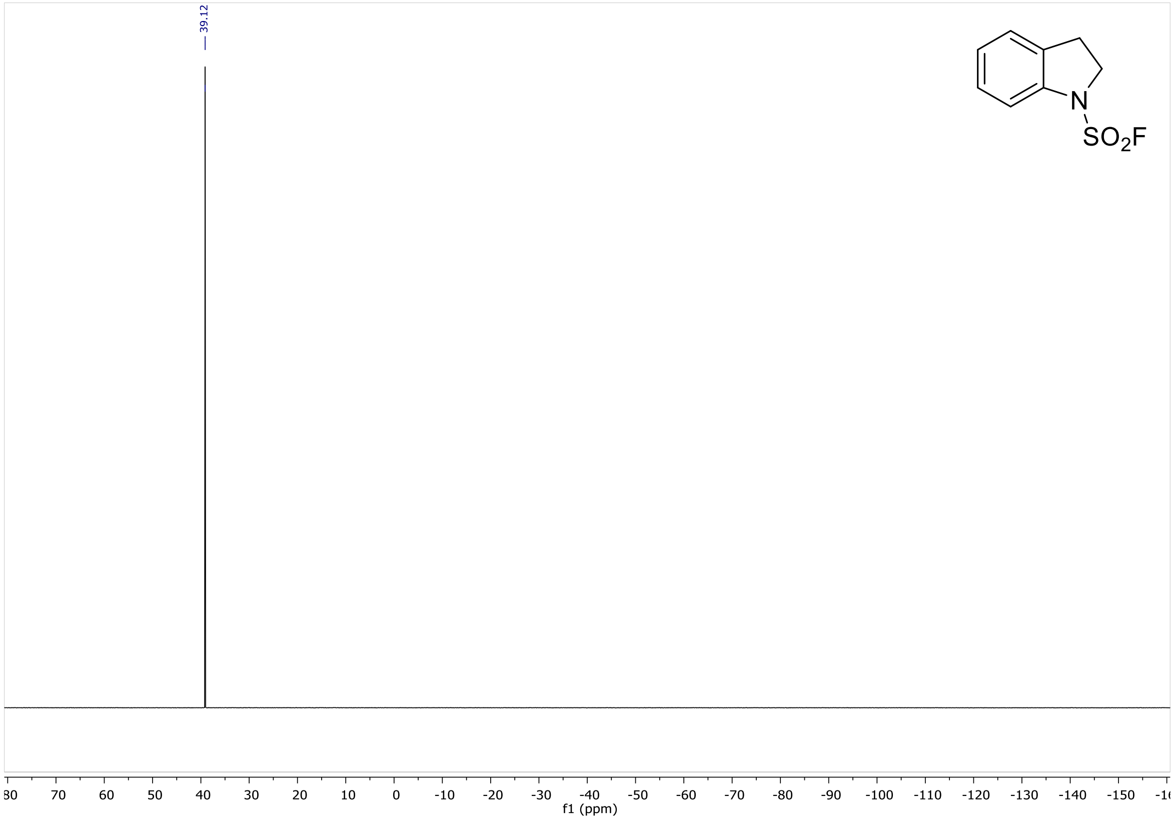

Isoindoline-2-sulfonyl fluoride (13d)

^1H NMR (400 MHz, $CDCl_3$)

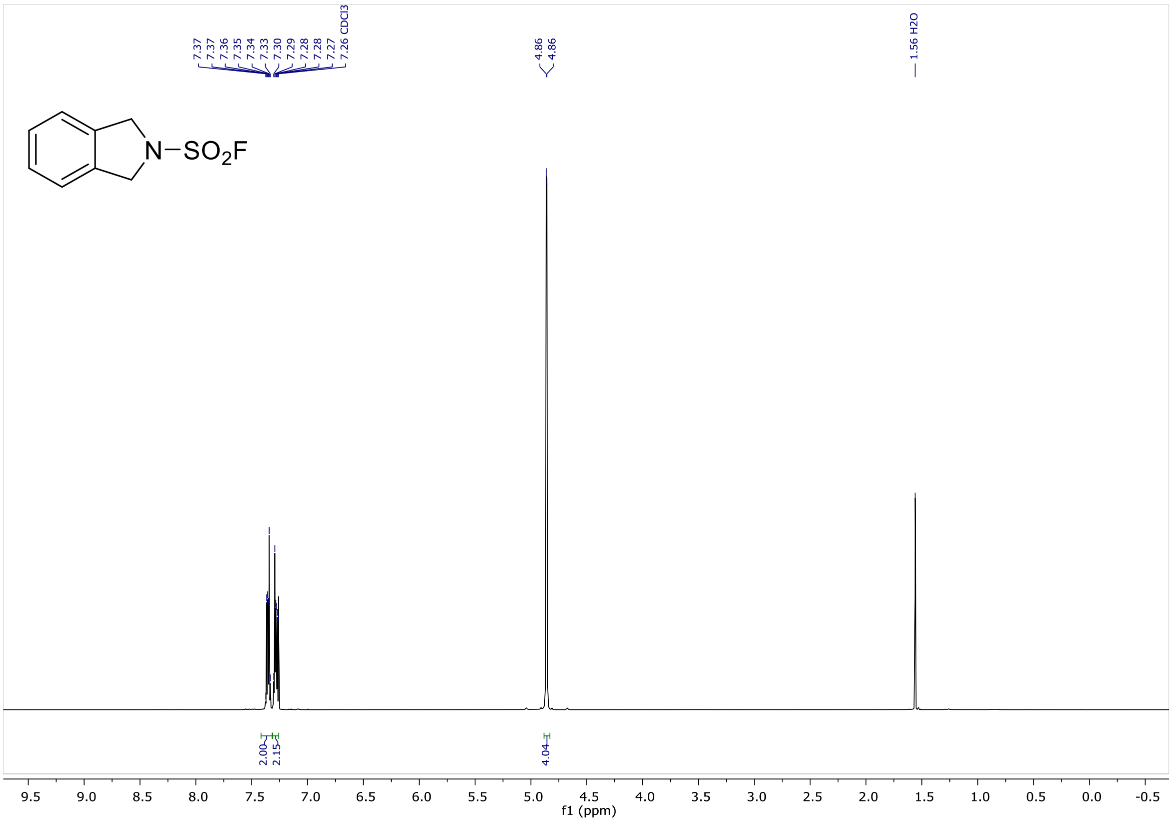

$^{13}C\{^1H\}$ NMR (101 MHz, $CDCl_3$)

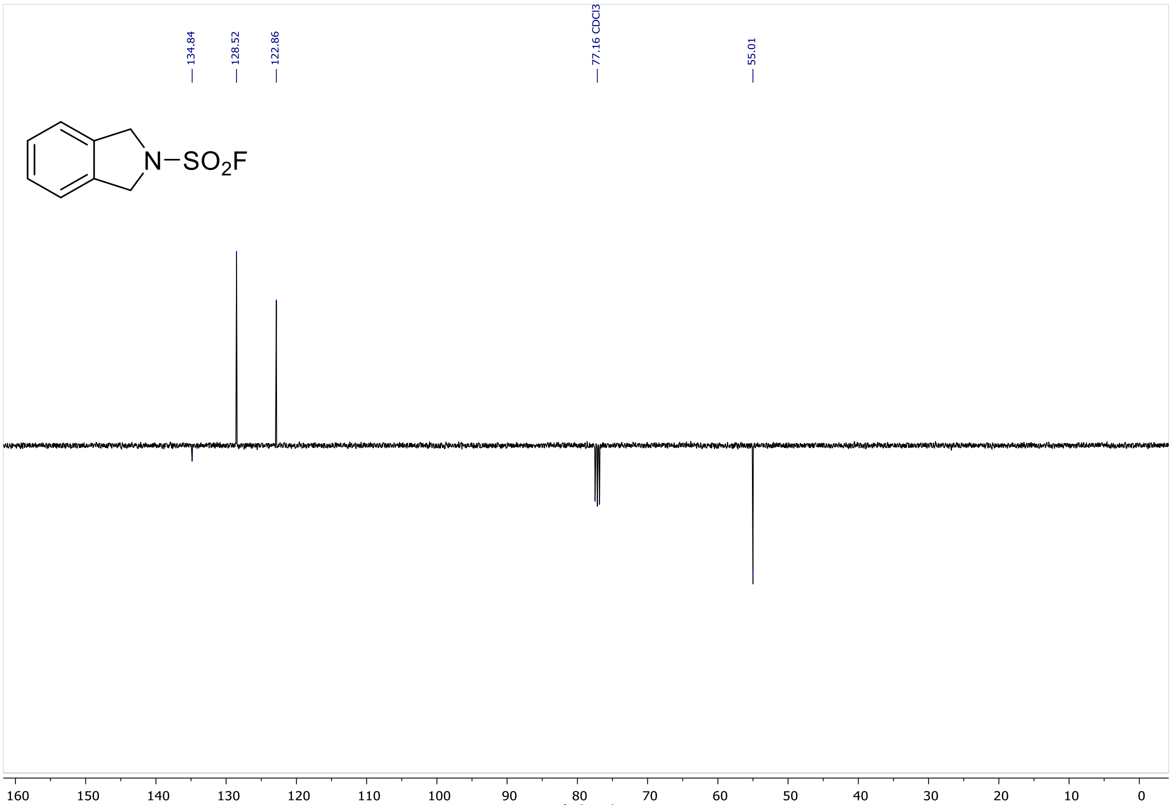

^{19}F NMR (376 MHz, $CDCl_3$)

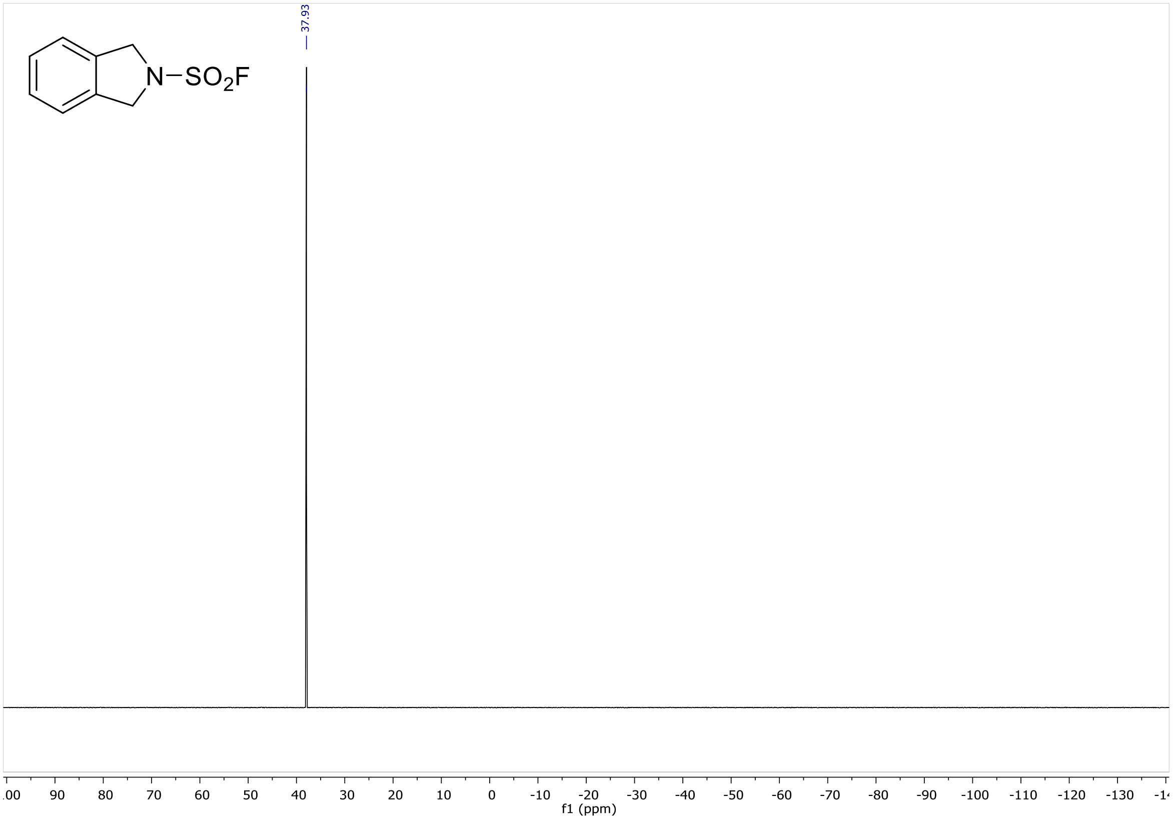

4-Benzylpiperidine-1-sulfonyl fluoride (13e)

^{1}H NMR (400 MHz, $CDCl_3$)

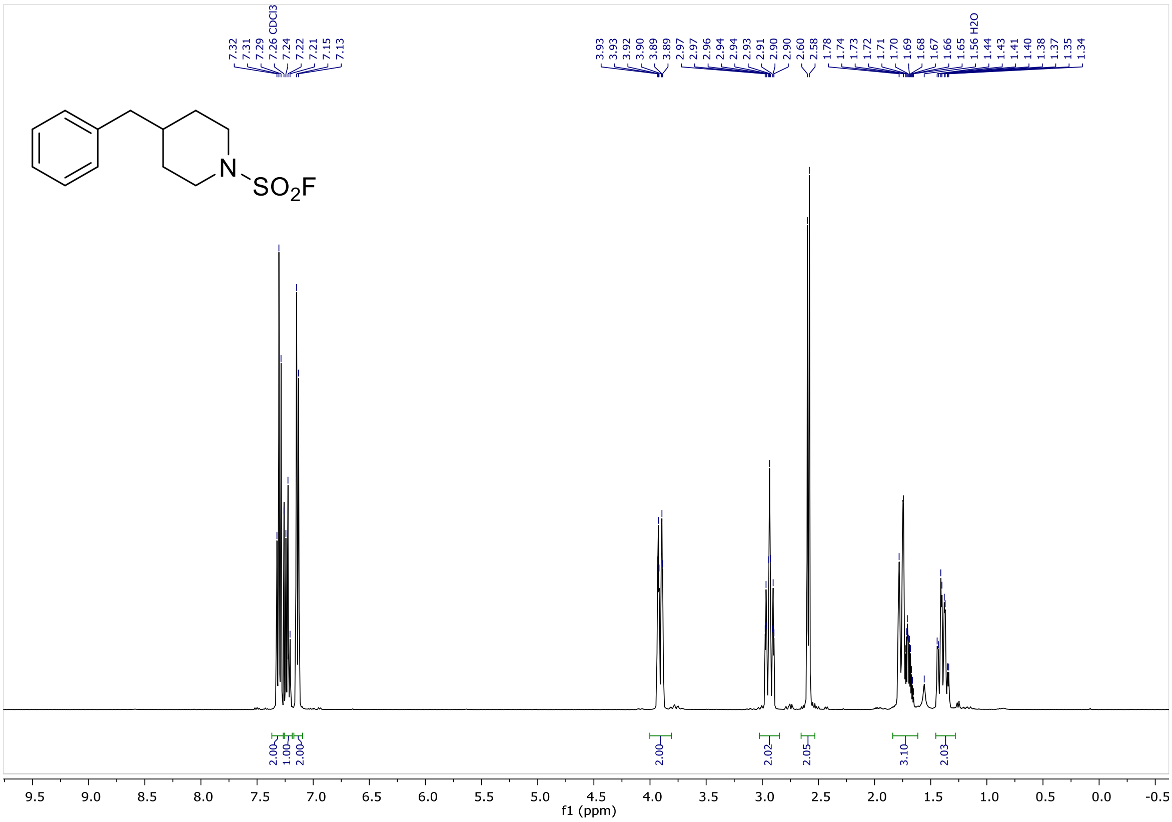

$^{13}C\{^1H\}$ NMR (101 MHz, $CDCl_3$)

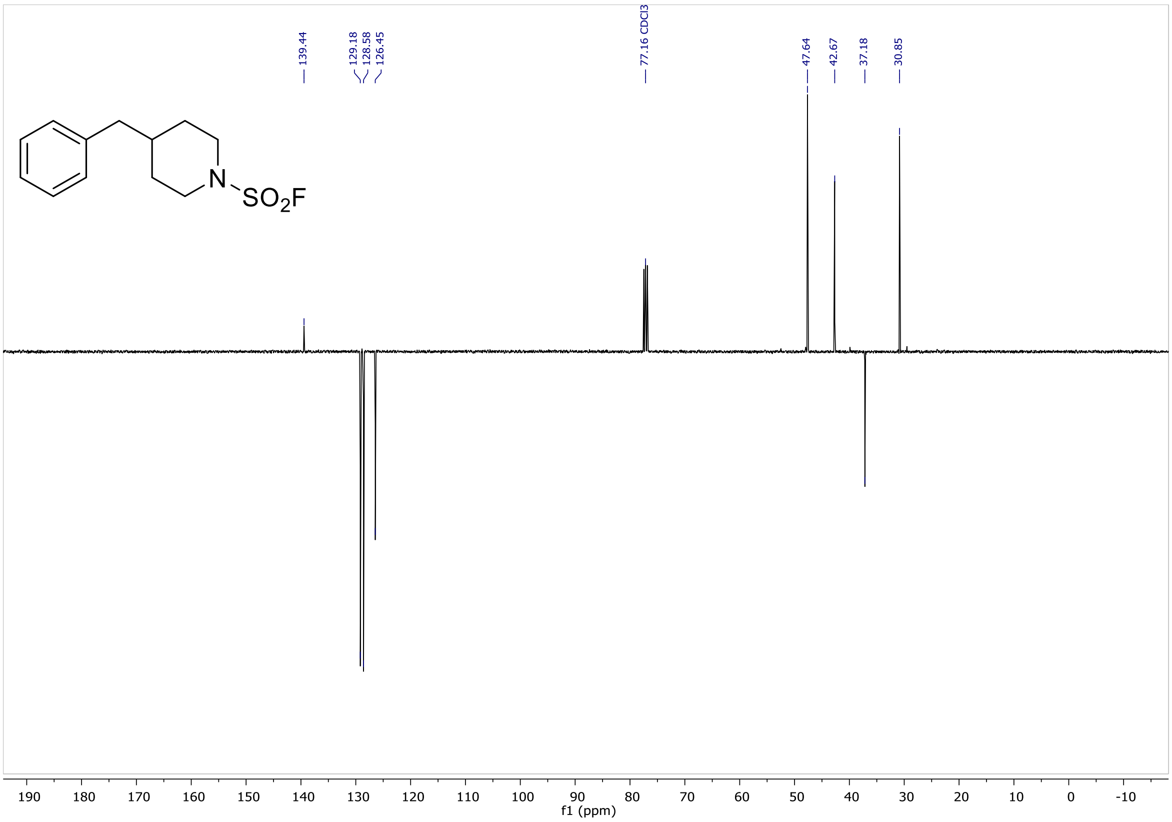

^{19}F NMR (376 MHz, $CDCl_3$)

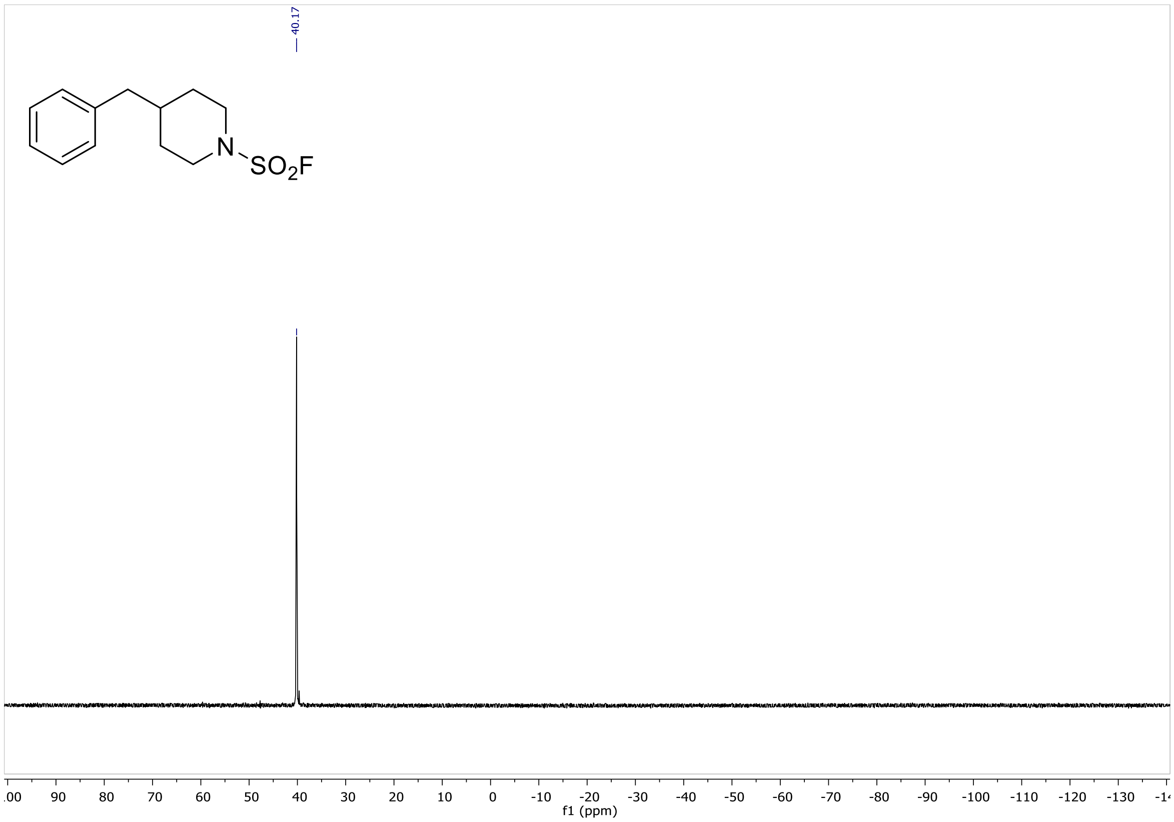

4-Hydroxypiperidyl-1-sulfonyl fluoride (13f)

^{1}H NMR (400 MHz, $CDCl_3$)

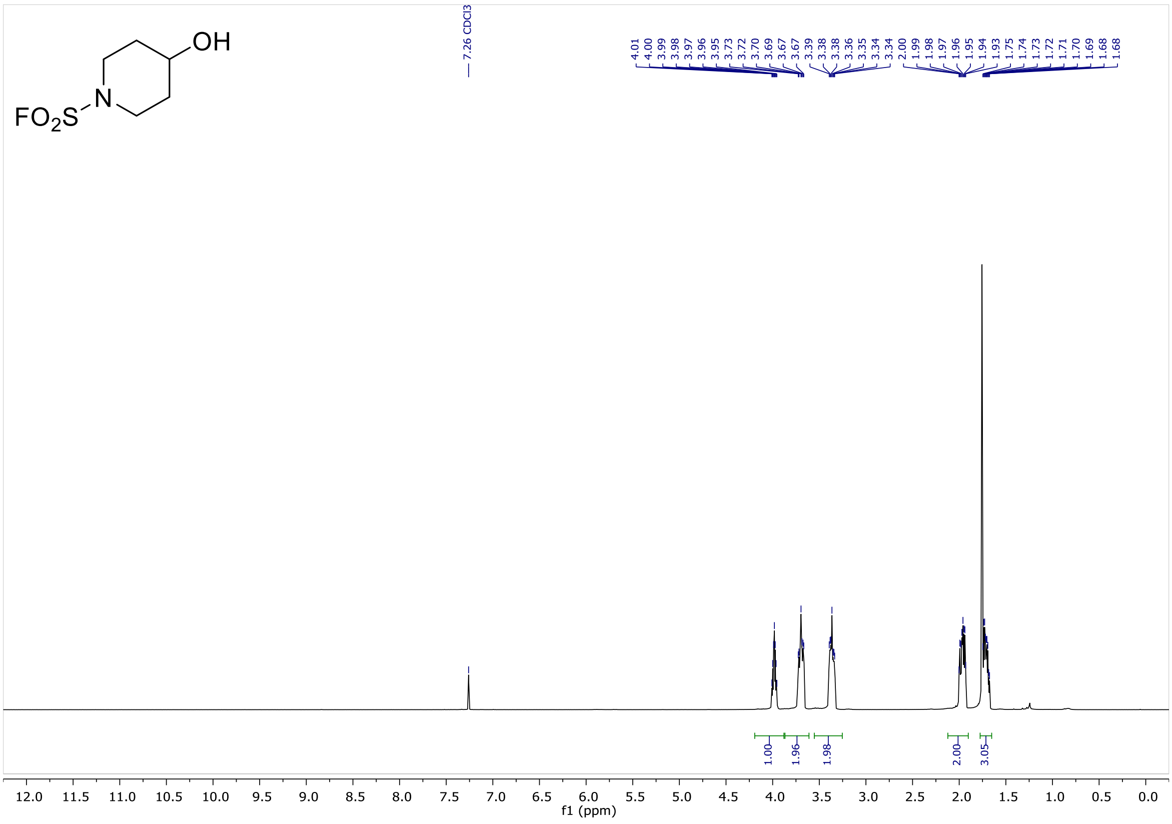

^{13}C{^{1}H} NMR (101 MHz, $CDCl_3$)

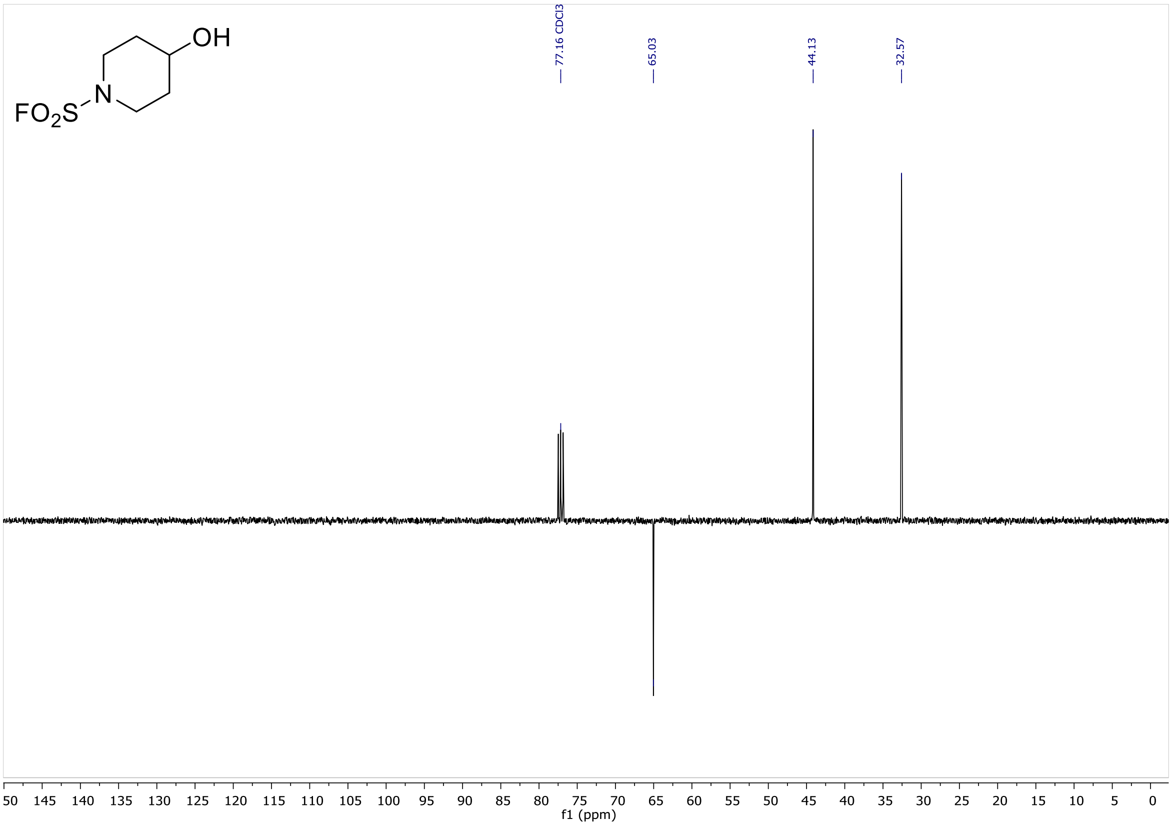

^{19}F NMR (376 MHz, $CDCl_3$)

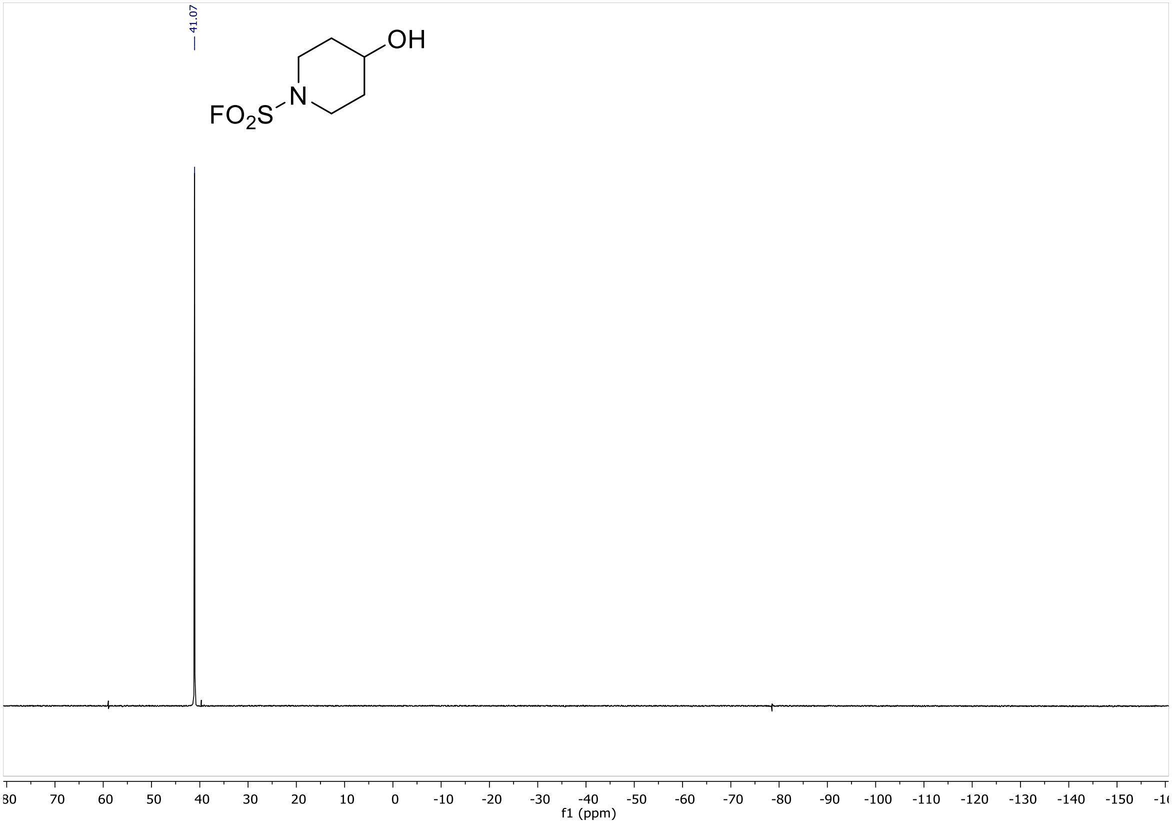

6-Hydroxy-1,2,3,4-tetrahydroisoquinoline-2-sulfonyl fluoride (13g)

^{1}H NMR (400 MHz, $CDCl_3$)

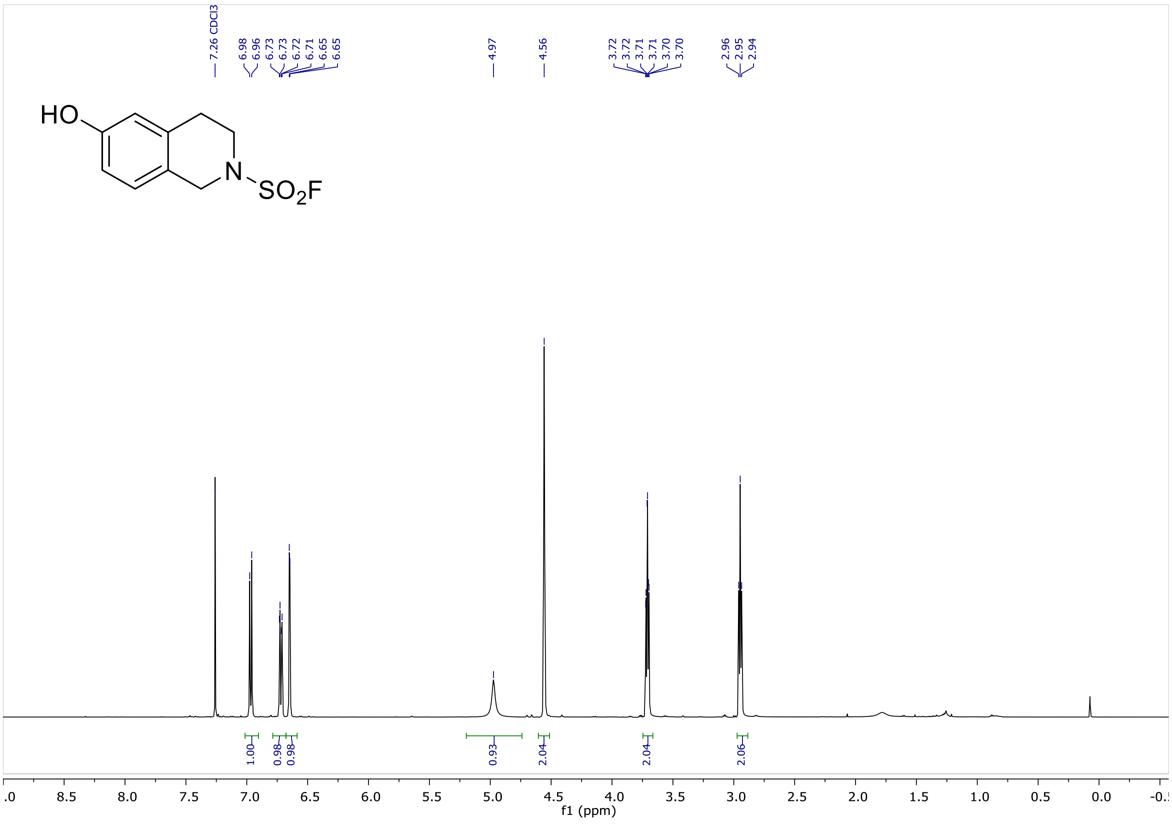

$^{13}C\{^1H\}$ NMR (101 MHz, $CDCl_3$)

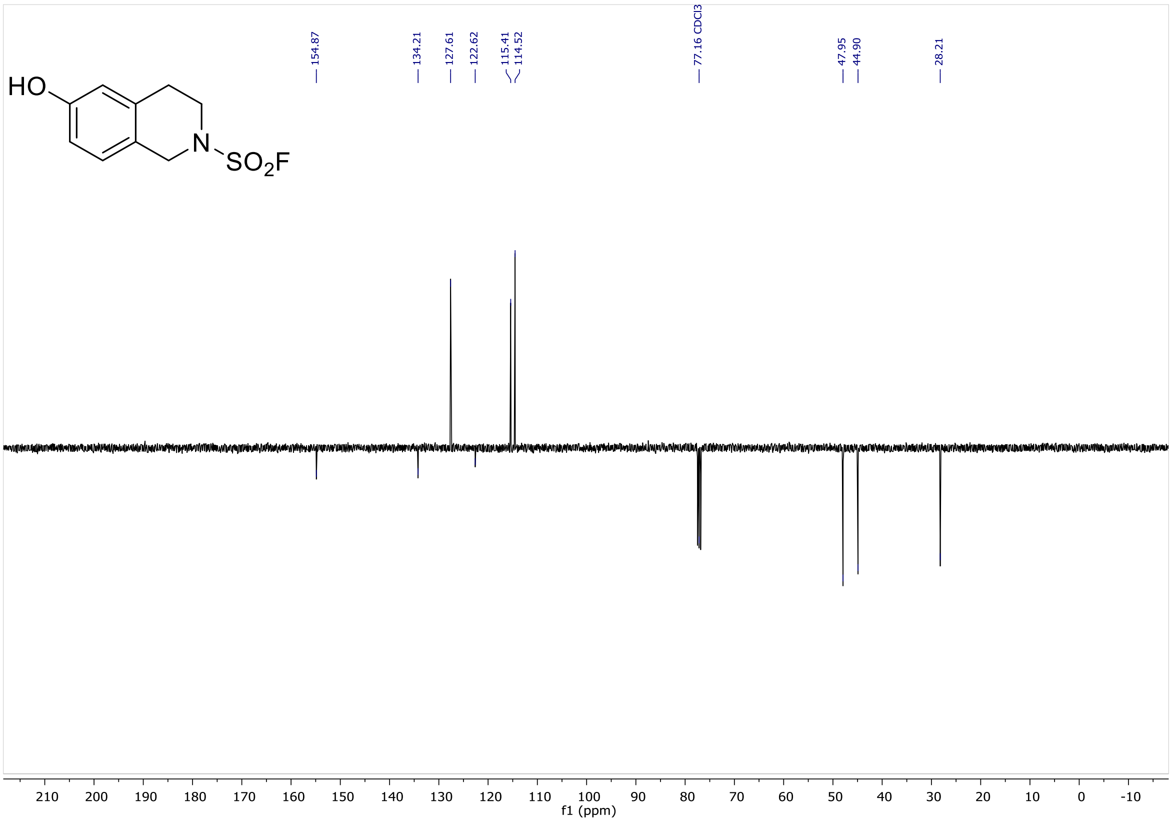

^{19}F NMR (376 MHz, $CDCl_3$)

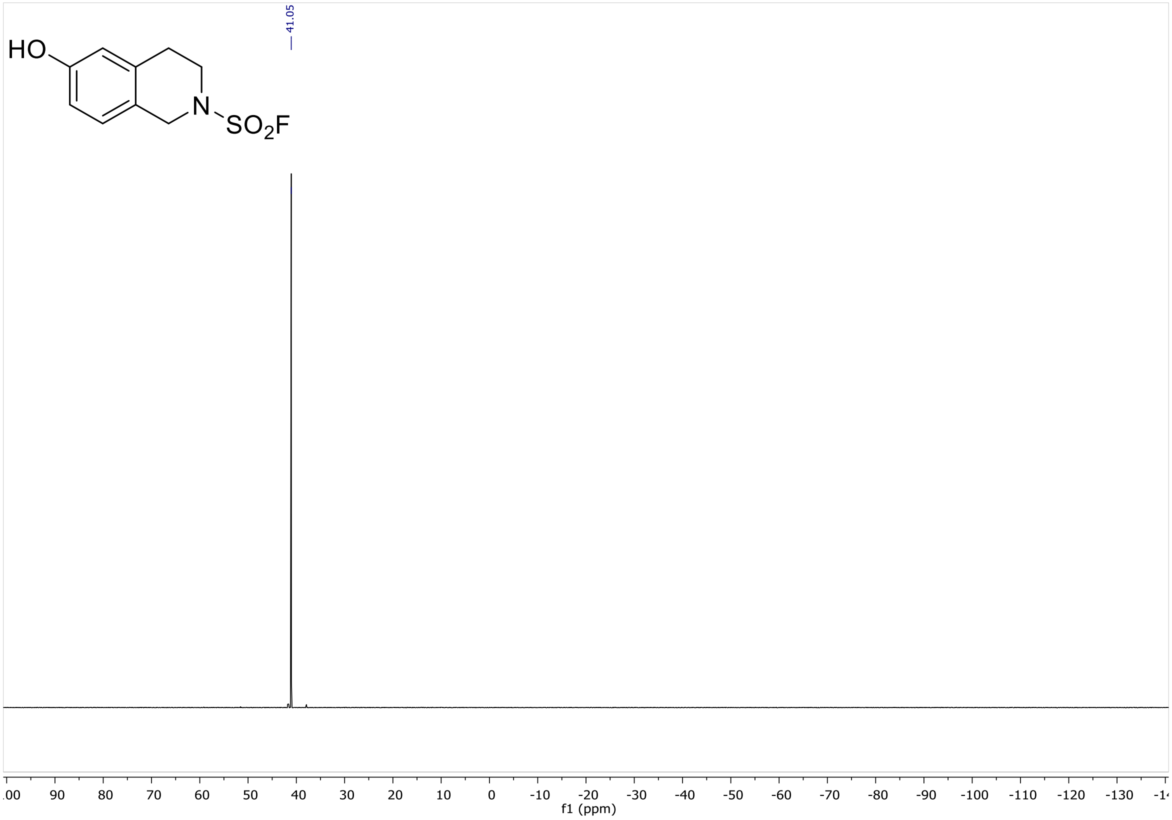

Phenylsulfamoyl fluoride (13h)

^{1}H NMR (400 MHz, $CDCl_3$)

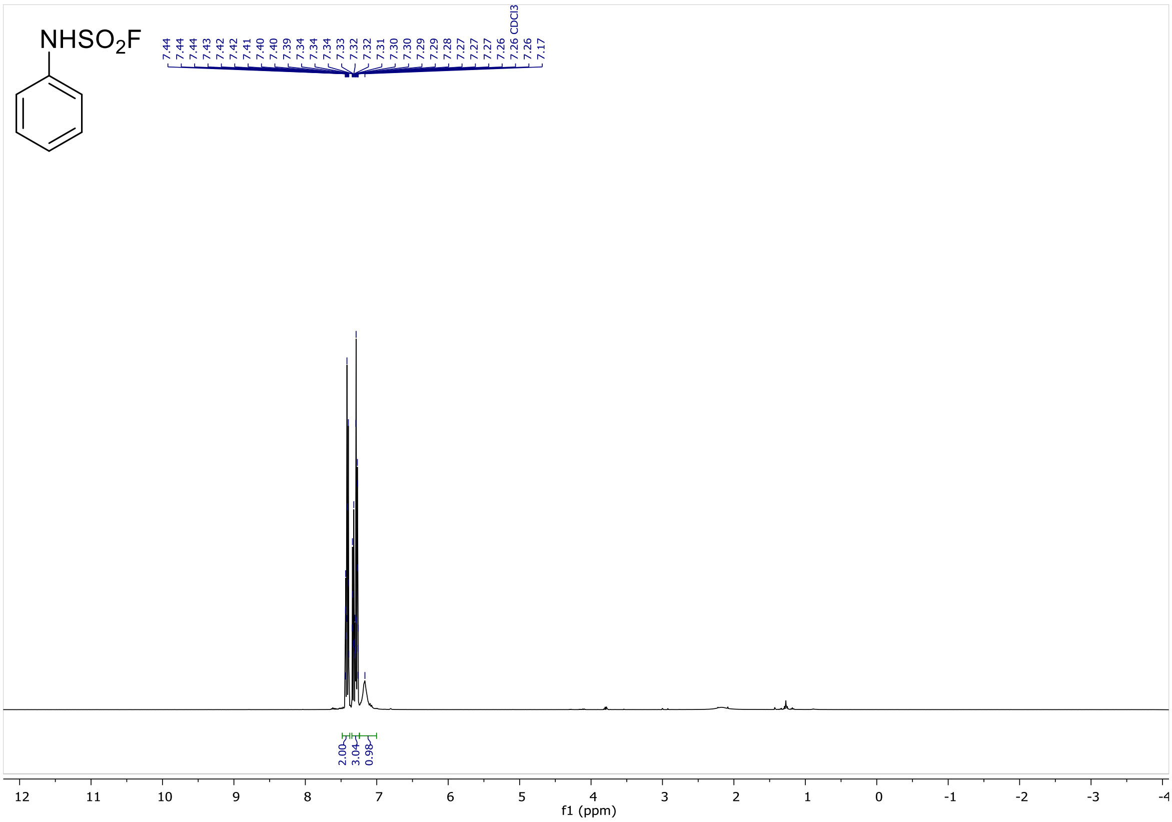

^{13}C{^{1}H} NMR (101 MHz, $CDCl_3$)

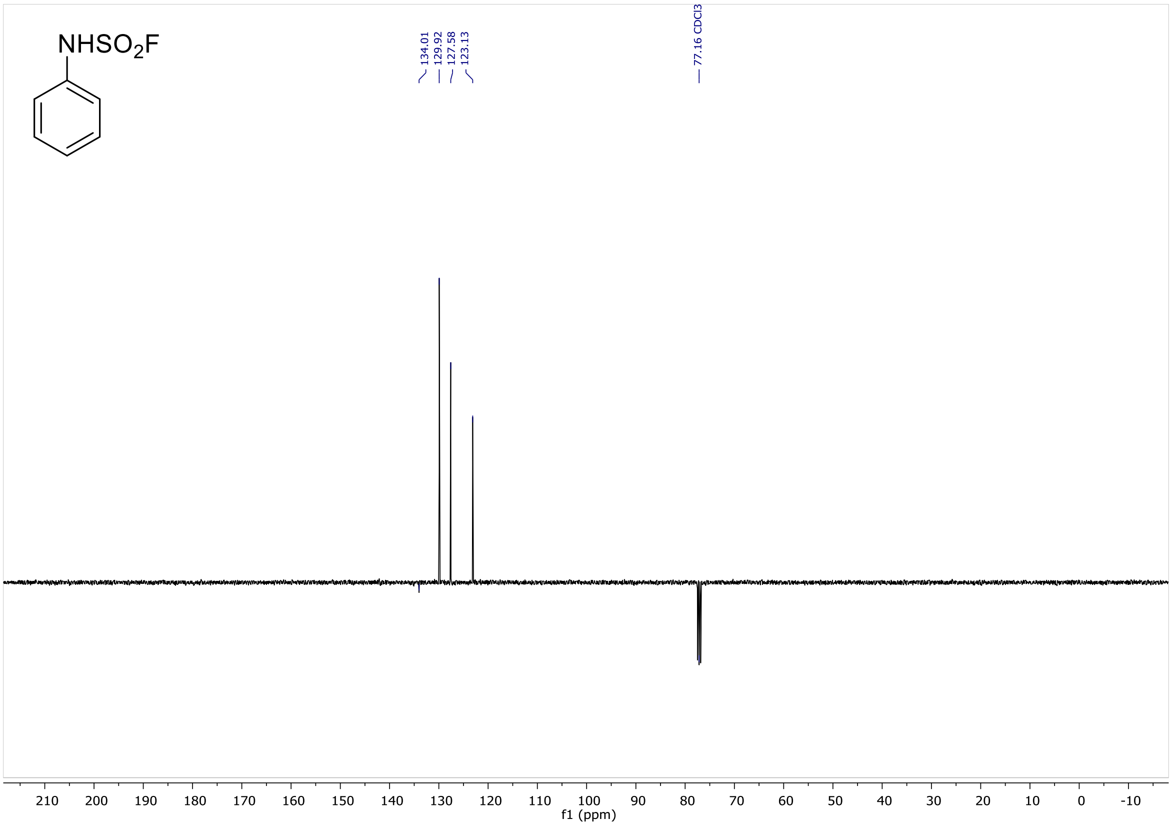

^{19}F NMR (376 MHz, $CDCl_3$)

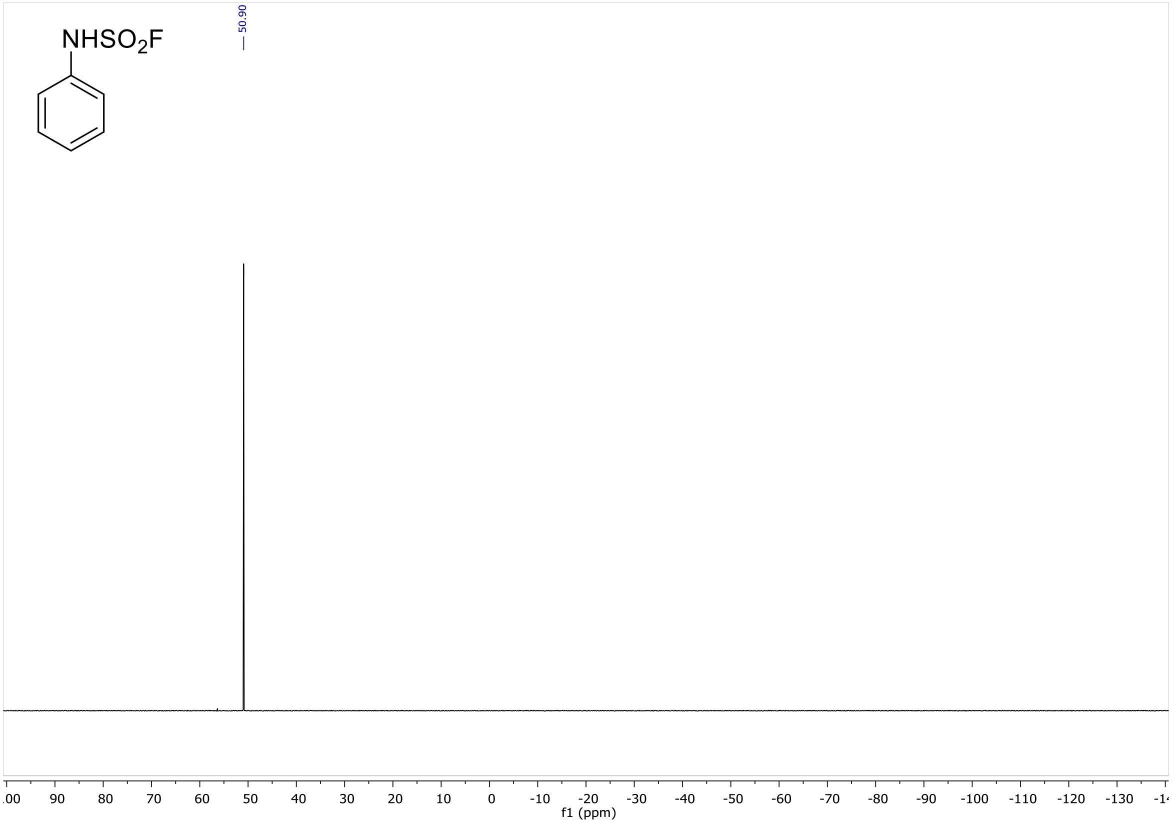

Phenyliminodisulfonyl difluoride (13i)

^1H NMR (400 MHz, $CDCl_3$)

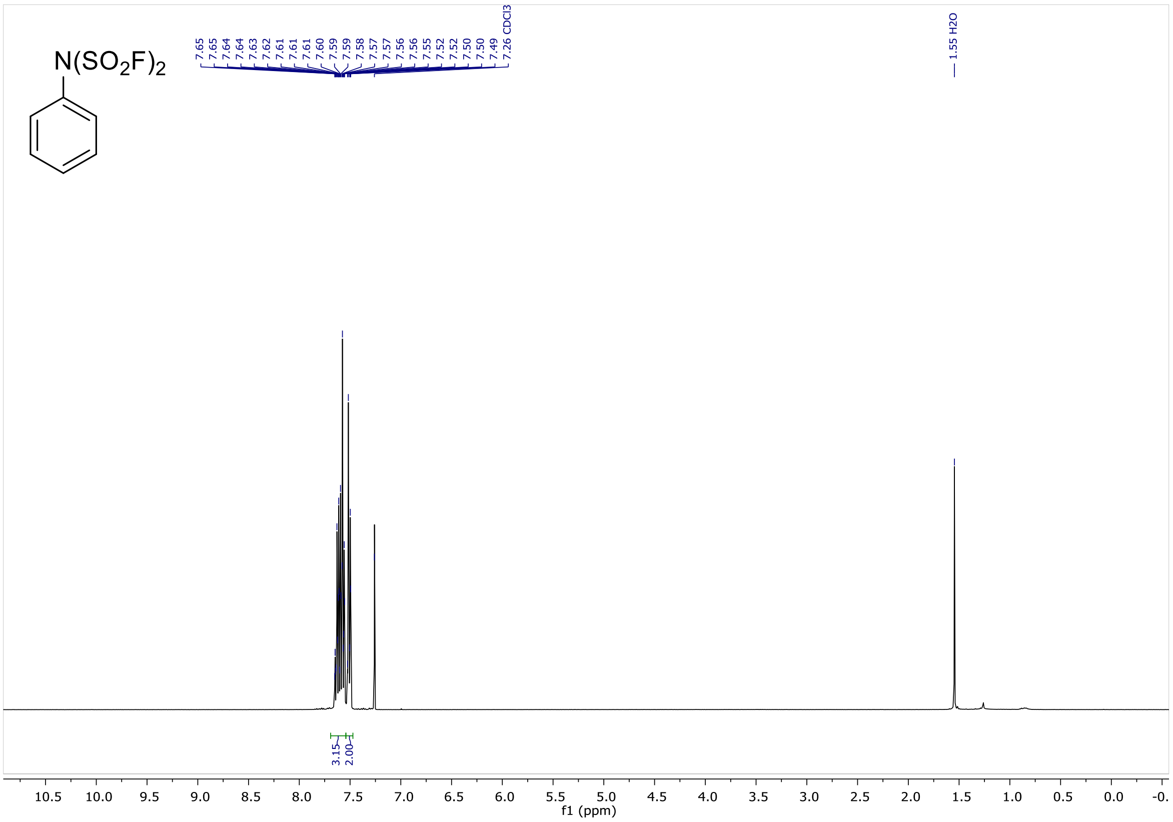

$^{13}C\{^1H\}$ NMR (101 MHz, $CDCl_3$)

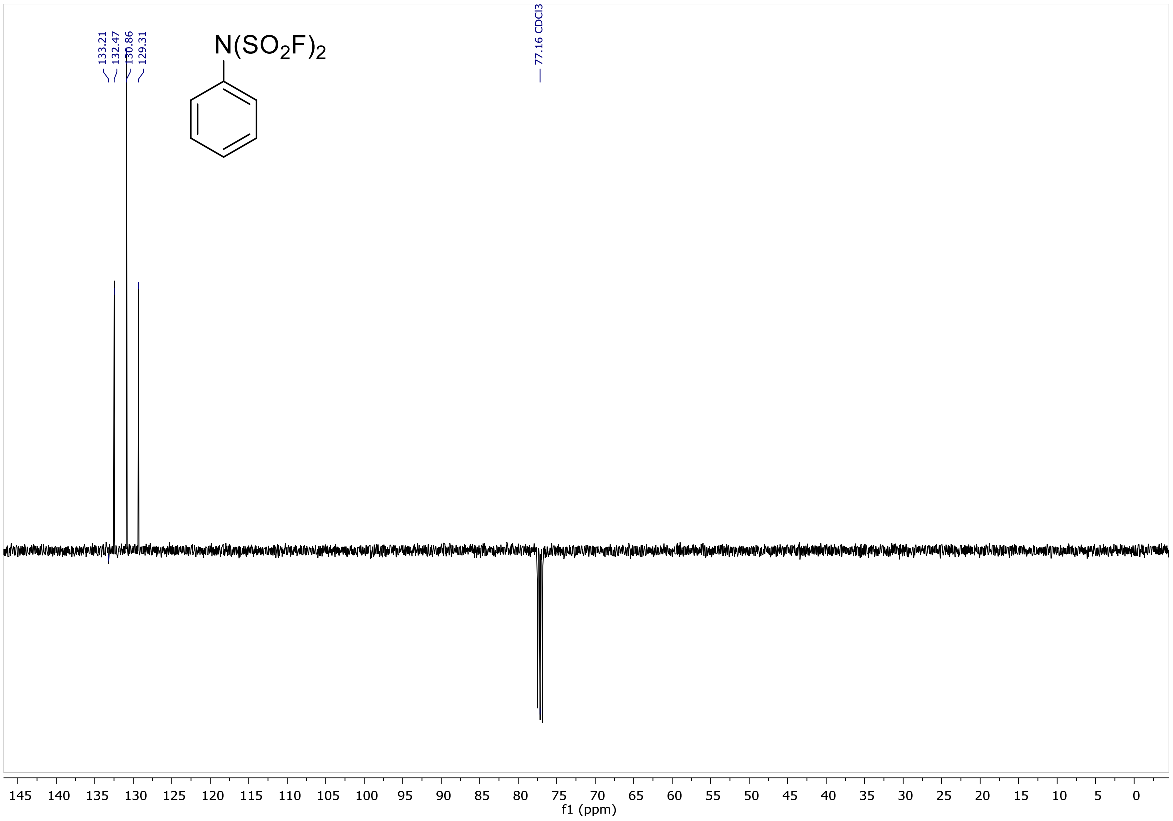

^{19}F NMR (376 MHz, $CDCl_3$)

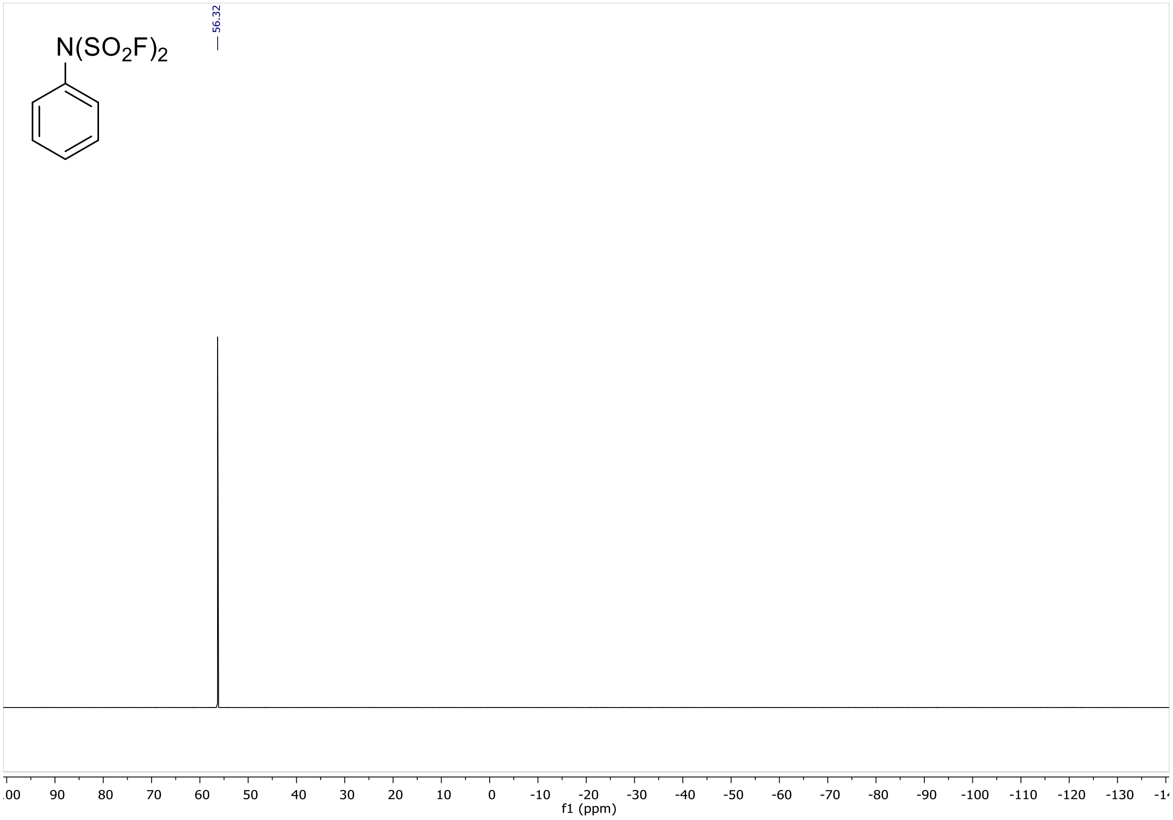

(4-Fluorobenzyl)sulfamoyl fluoride (13j)

^{1}H NMR (400 MHz, $CDCl_3$)

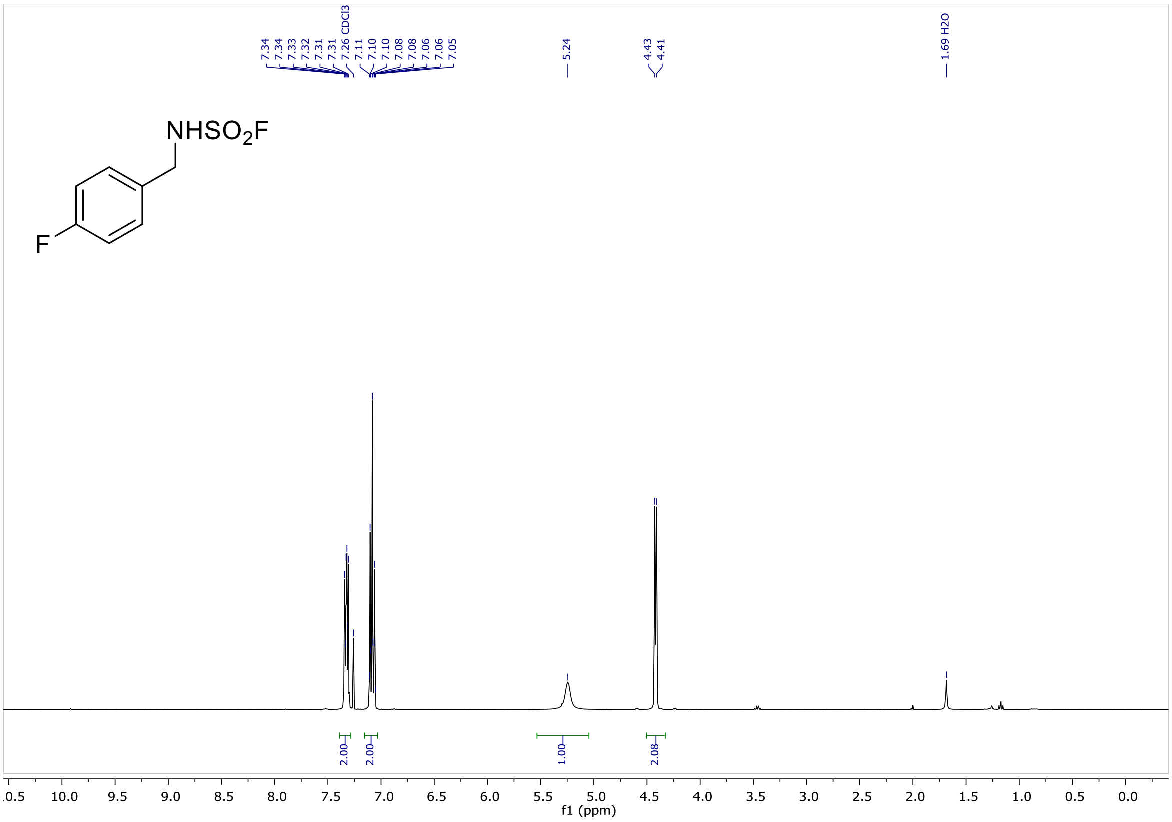

$^{13}C\{^{1}H\}$ NMR (101 MHz, $CDCl_3$)

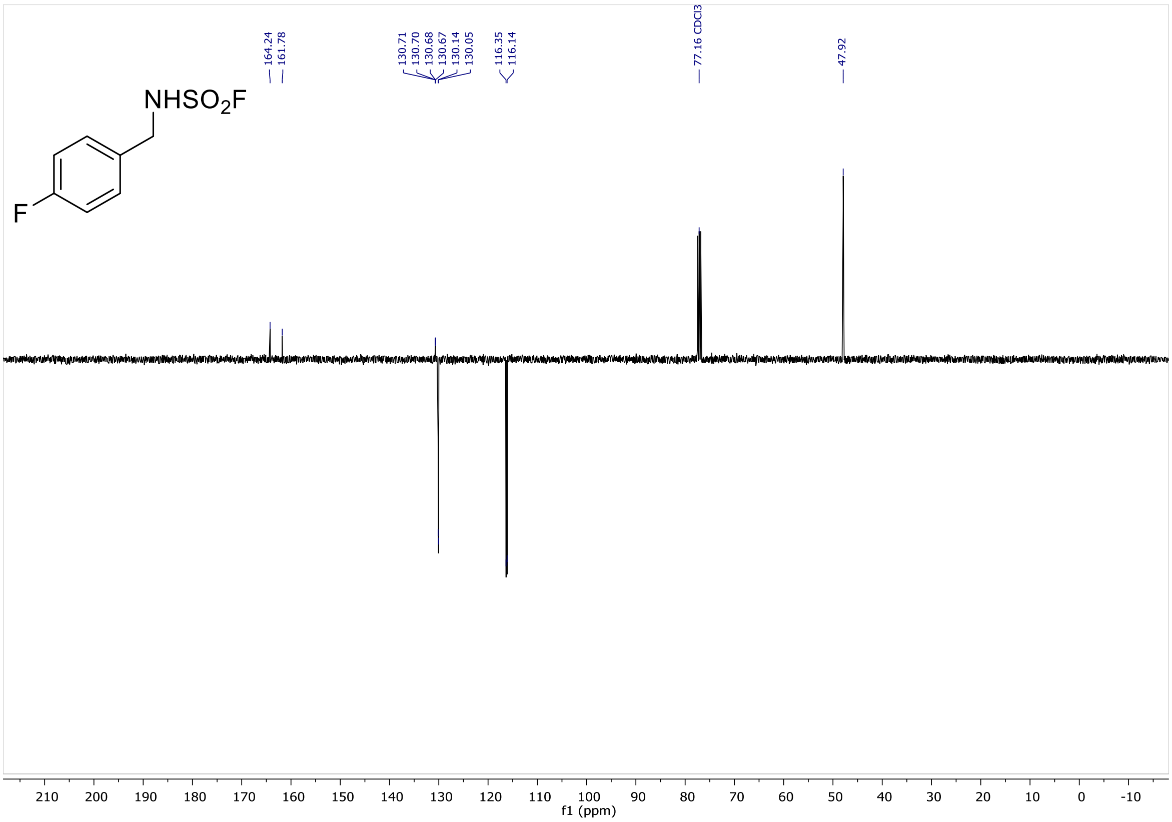

^{19}F NMR (376 MHz, $CDCl_3$)

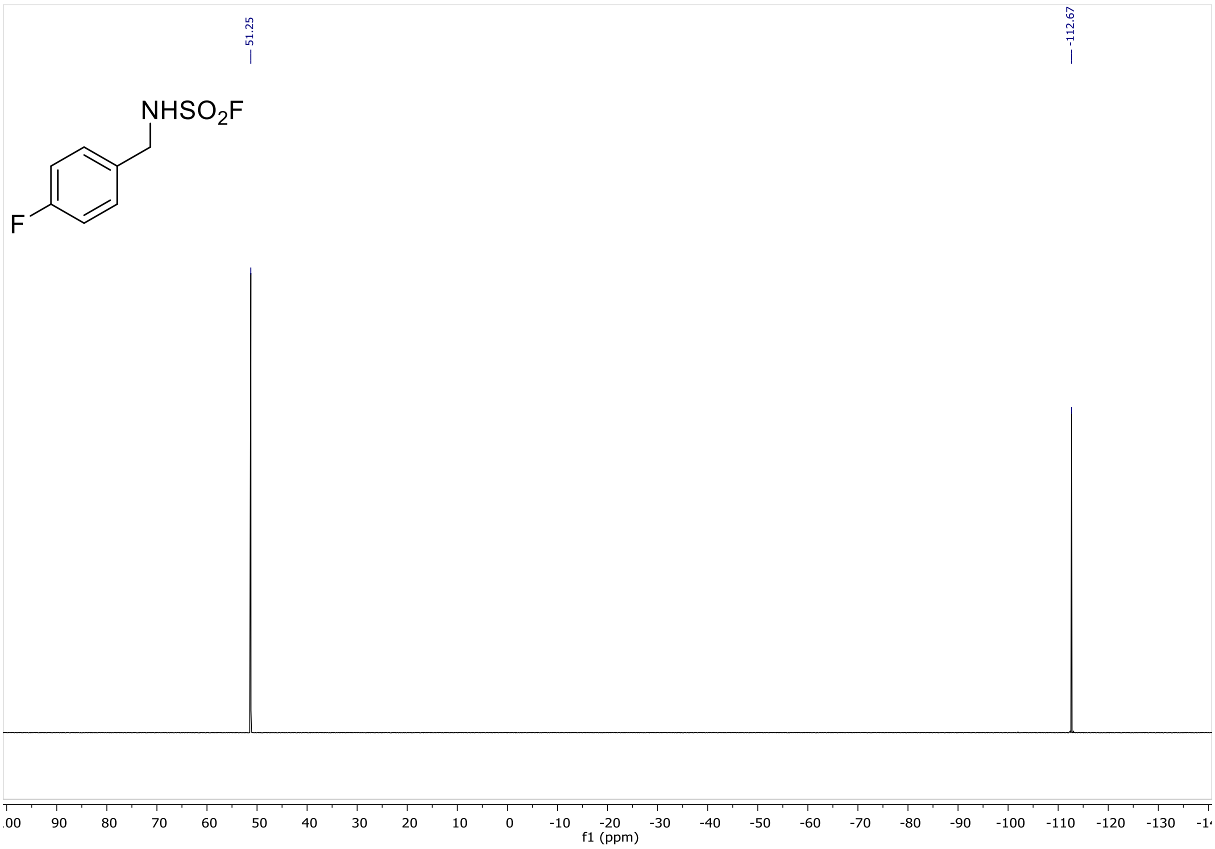

4-(2-Chlorodibenzo[b,f][1,4]oxazepin-11-yl)piperazine-1-sulfonyl fluoride (13k)

^{1}H NMR (400 MHz, $CDCl_3$)

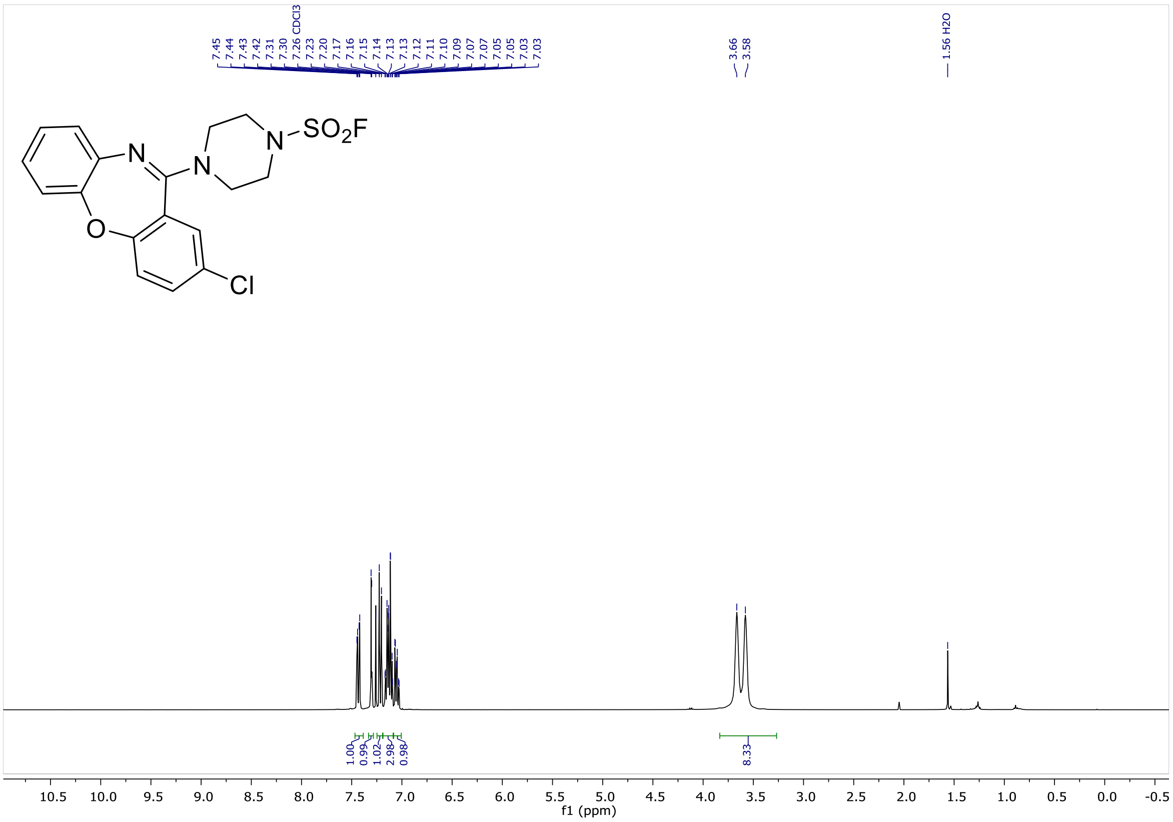

$^{13}C\{^{1}H\}$ NMR (101 MHz, $CDCl_3$)

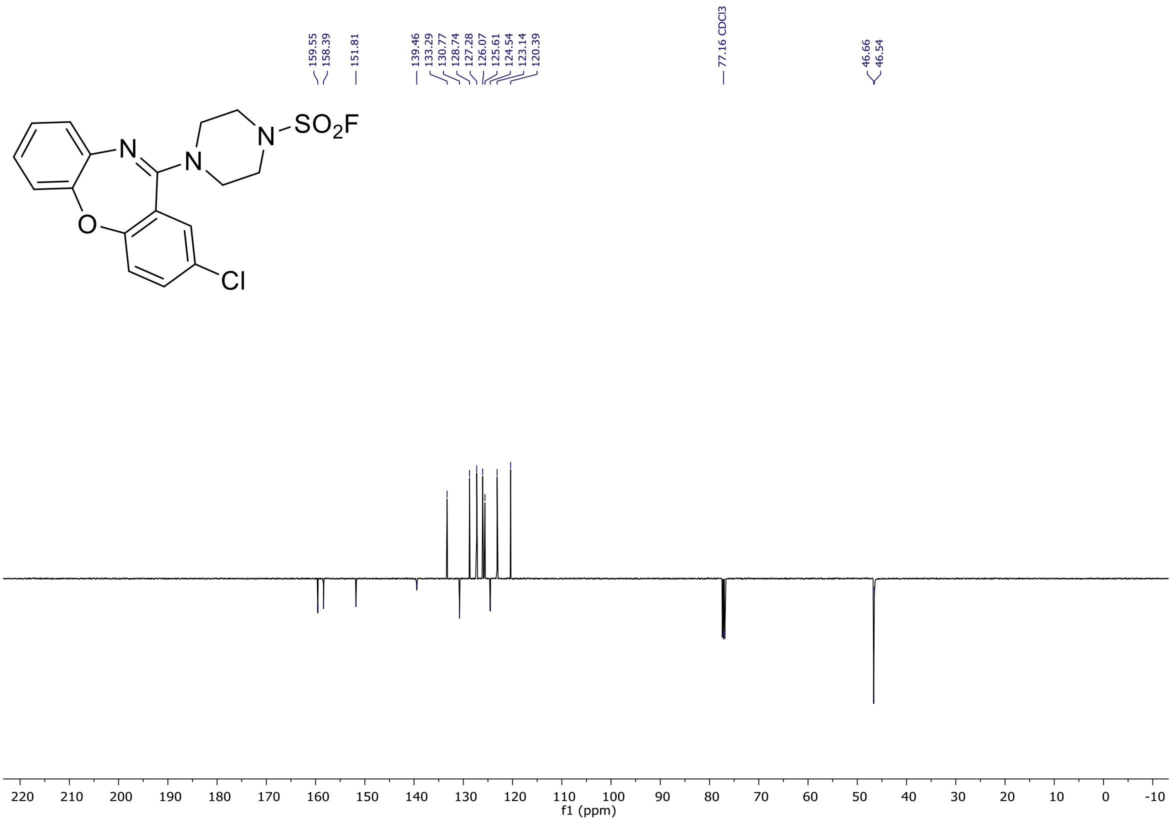

^{19}F NMR (376 MHz, $CDCl_3$)

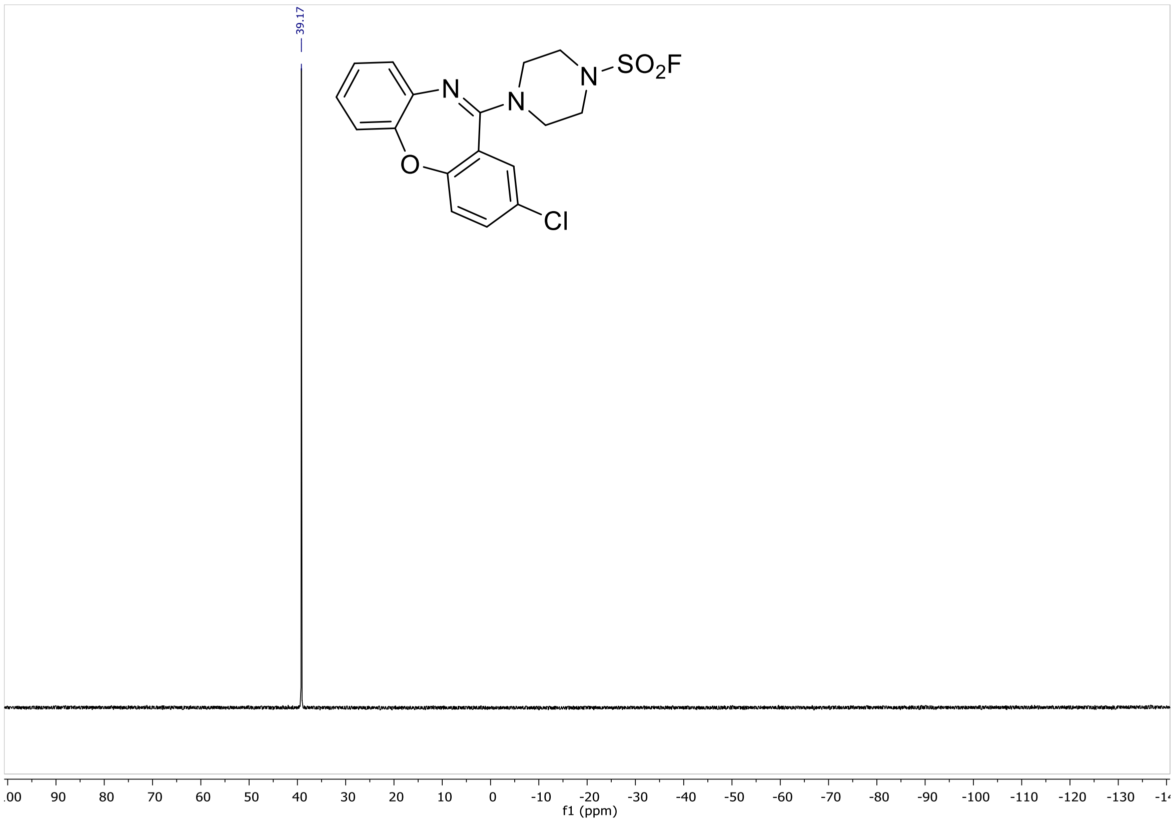

***tert*-Butyl {2-[1-(fluorosulfonyl)-1*H*-indol-3-yl]ethyl}carbamate (13l)**

^{1}H NMR (400 MHz, $CDCl_3$)

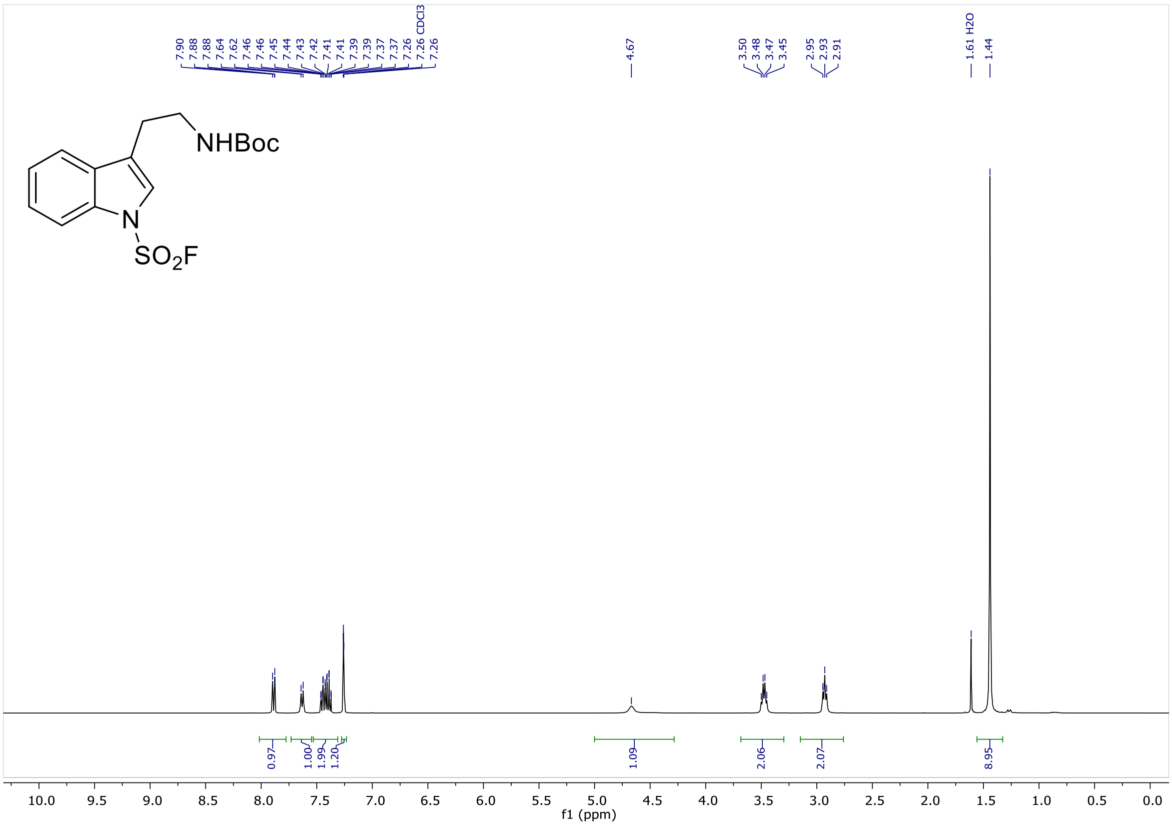

$^{13}C\{^{1}H\}$ NMR (101 MHz, $CDCl_3$)

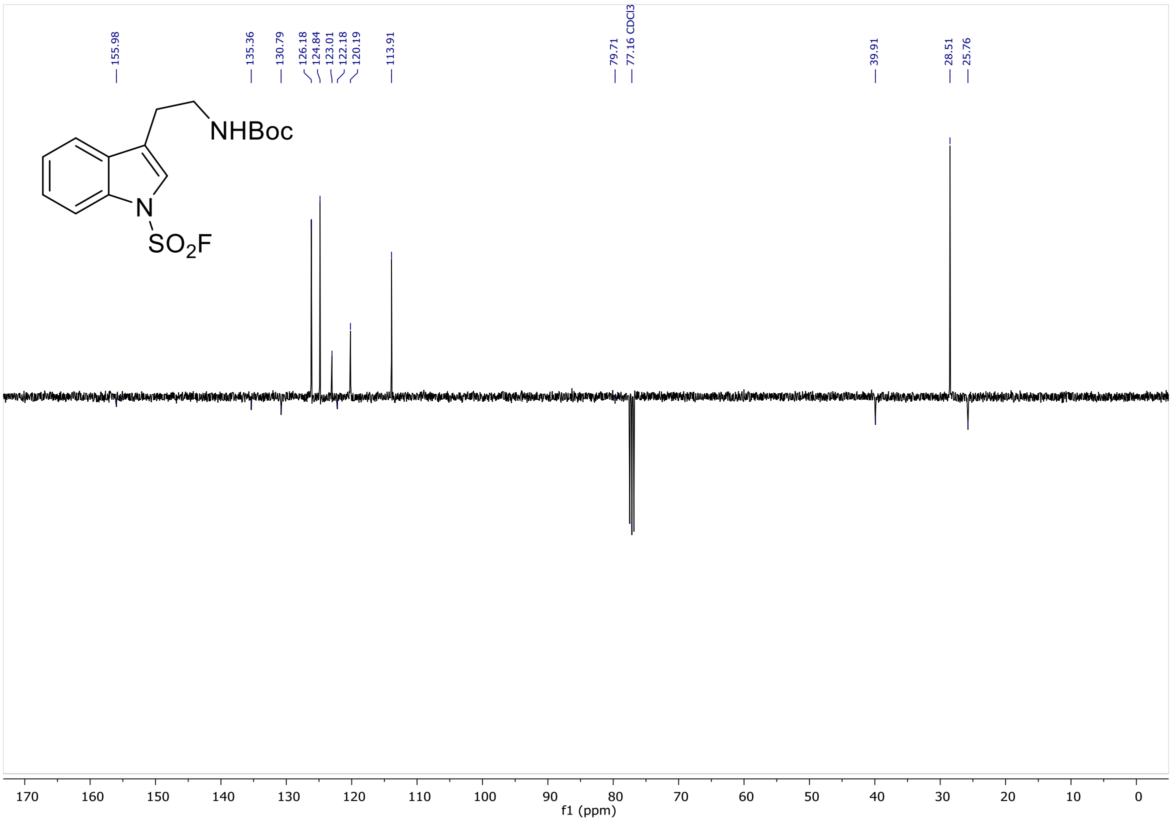

^{19}F NMR (376 MHz, $CDCl_3$)

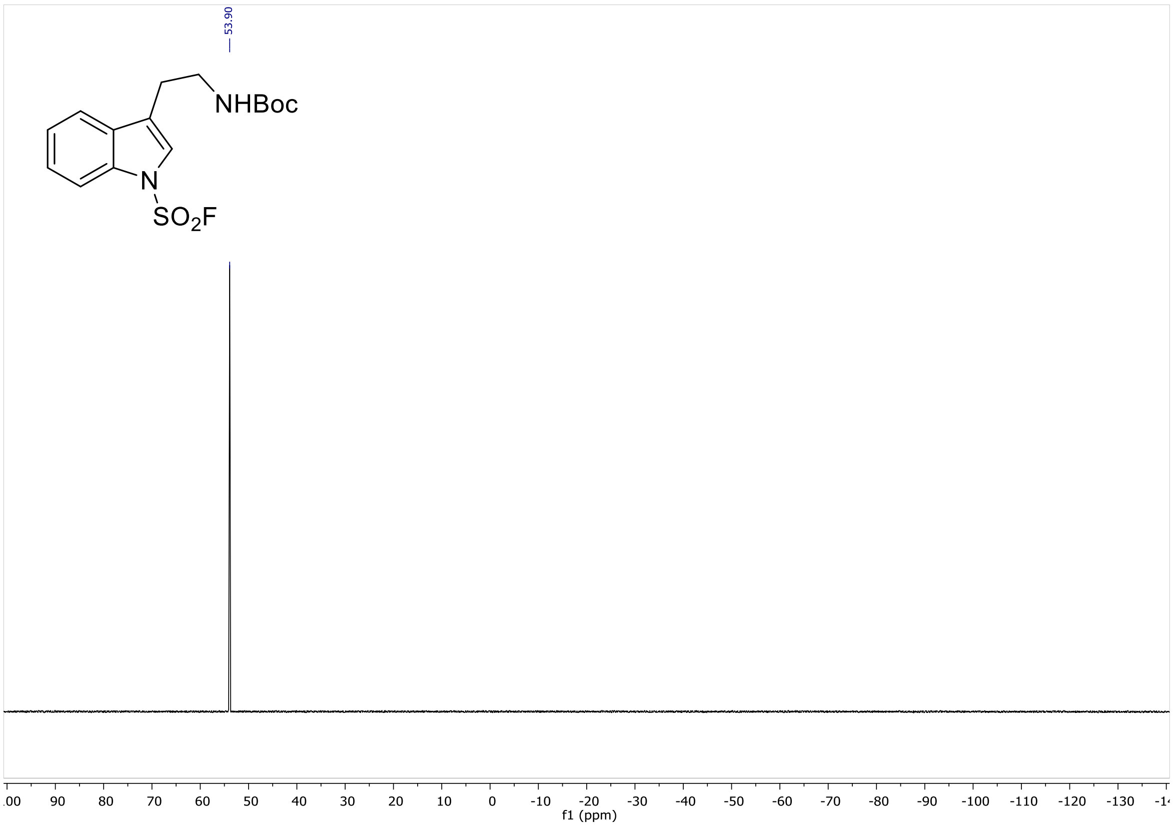

1-(Fluorosulfonyl)piperidine-4-carboxylic acid (13m)

^{1}H NMR (400 MHz, CD_3OD)

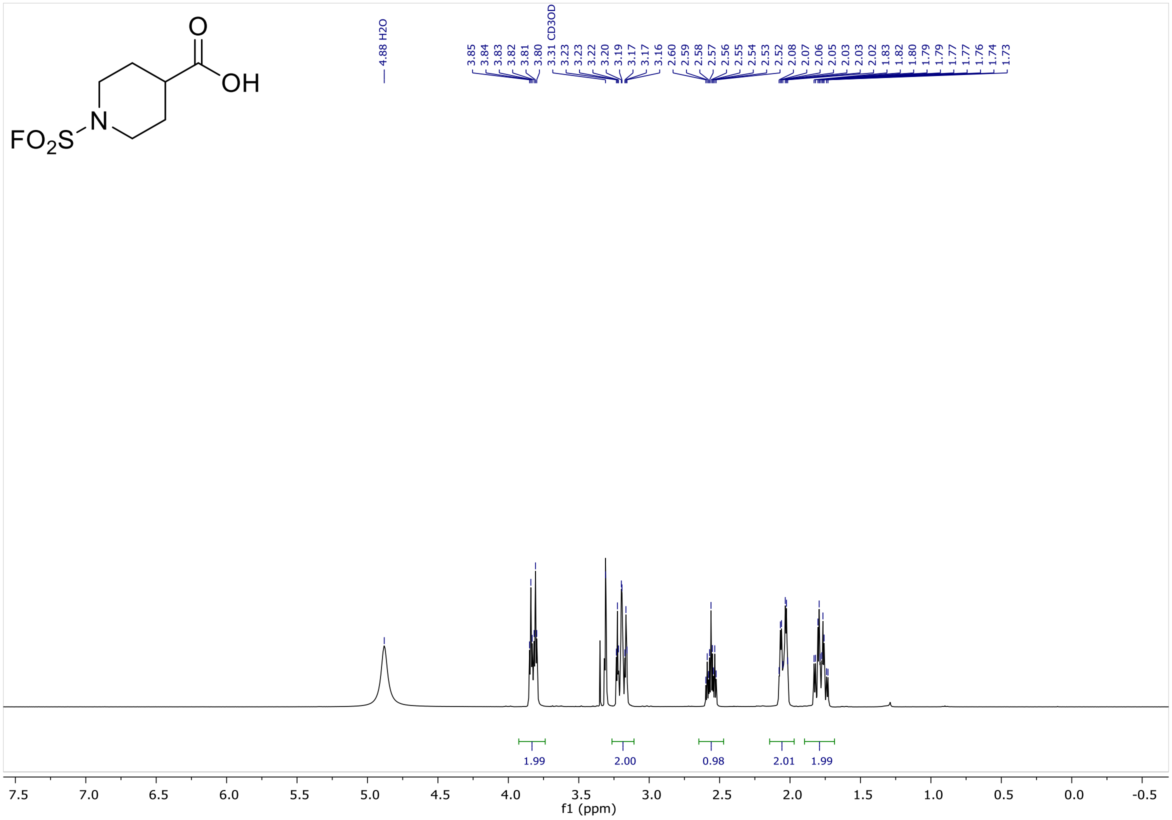

^{13}C{^{1}H} NMR (101 MHz, CD_3OD)

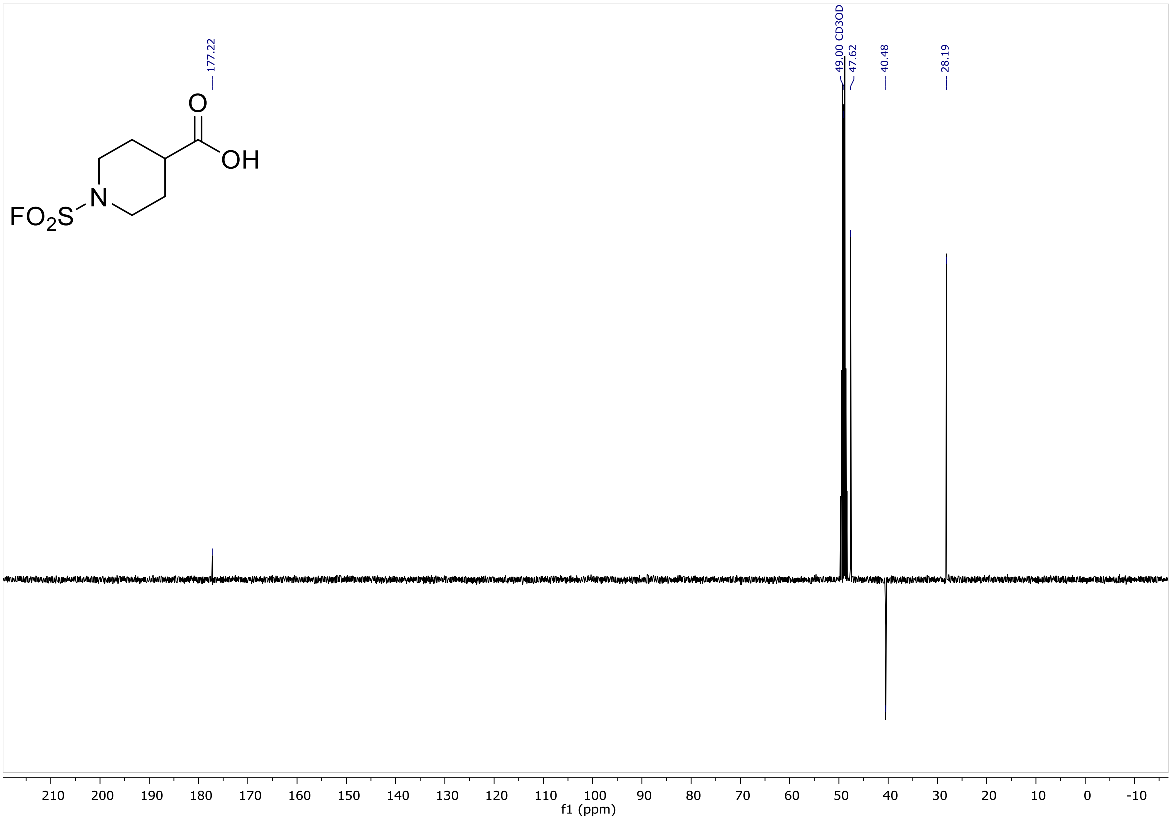

^{19}F NMR (376 MHz, CD_3OD)

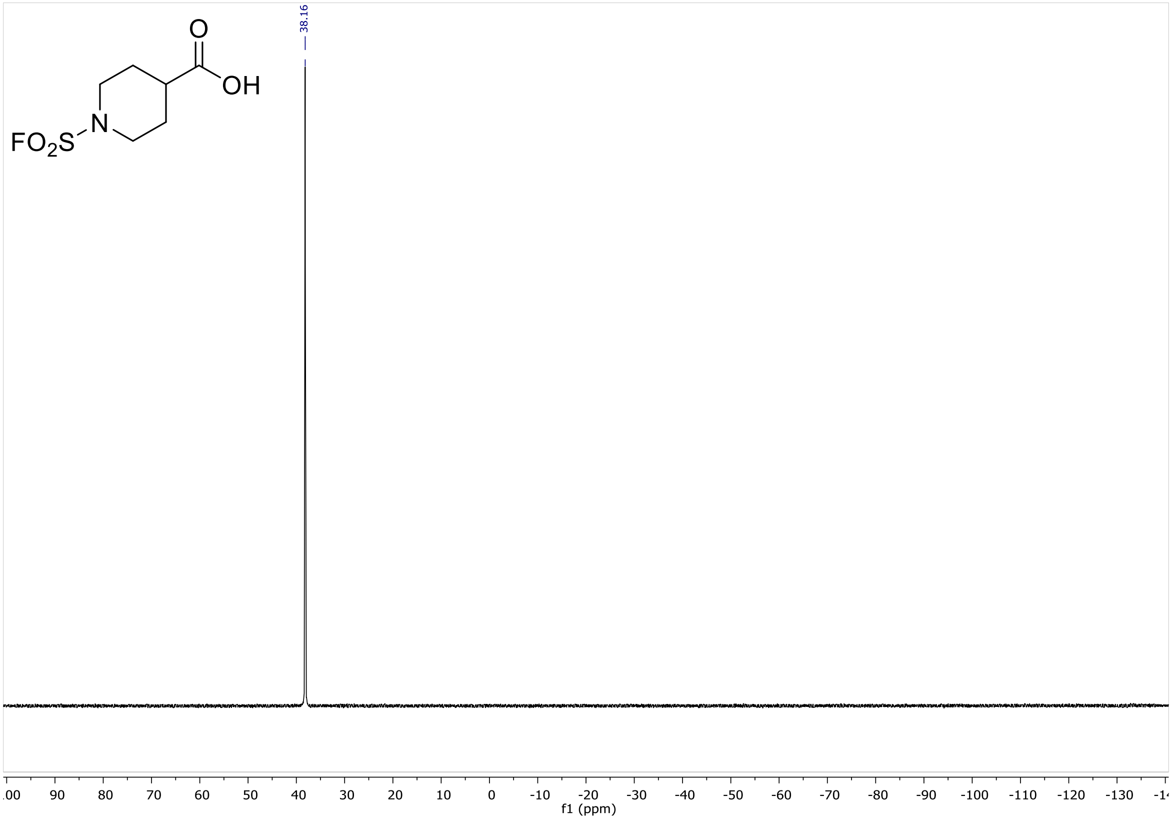

Supplement: Supplementary file 1 — jo3c02643_si_001.pdf [file jo3c02643_si_001.pdf]
